# A multicentre, patient- and assessor-blinded, non-inferiority, randomised and controlled phase II trial to compare standard and torque teno virus-guided immunosuppression in kidney transplant recipients in the first year after transplantation: TTVguideIT

**DOI:** 10.1186/s13063-023-07216-0

**Published:** 2023-03-22

**Authors:** Frederik Haupenthal, Jette Rahn, Fabrizio Maggi, Fanny Gelas, Philippe Bourgeois, Christian Hugo, Bernd Jilma, Georg A. Böhmig, Harald Herkner, Michael Wolzt, Konstantin Doberer, Matthias Vossen, Daniele Focosi, Hannes Neuwirt, Miriam Banas, Bernhard Banas, Klemens Budde, Ondrej Viklicky, Paolo Malvezzi, Lionel Rostaing, Joris I. Rotmans, Stephan J. L. Bakker, Kathrin Eller, Daniel Cejka, Alberto Molina Pérez, David Rodriguez-Arias, Franz König, Gregor Bond, the TTVguideTX consortium partners, Georg Melzer, Martha del Alamo, Isabel Beneyto, David Navarro, Sophie Ohlmann

**Affiliations:** 1 Division of Nephrology and Dialysis, Department of Medicine III, Medical University of Vienna, Vienna, Austria; 2 Coordination Center for Clinical Trials, Faculty of Medicine Carl Gustav Carus, Technische Universität Dresden, Dresden, Germany; 3 Laboratory of Virology, National Institute for Infectious Diseases L. Spallanzani, Rome, Italy; 4 bioMérieux SA, Centre Christophe Merieux, Grenoble, France; 5 Universitätsklinikum Carl Gustav Carus an der Technischen Universität Dresden, Dresden, Germany; 6 Department of Clinical Pharmacology, Medical University of Vienna, Vienna, Austria; 7 Department of Emergency Medicine, Medical University of Vienna, Vienna, Austria; 8 Clinical Trials Coordination Centre, Medical University of Vienna, Vienna, Austria; 9 Division of Infectious diseases and Tropical Medicine, Department of Medicine I, Medical University of Vienna, Vienna, Austria; 10 North-Western Tuscany Blood Bank, Pisa, Italy; 11 Department of Internal Medicine IV, Nephrology and Hypertension, Medical University Innsbruck, Innsbruck, Austria; 12 Department of Nephrology, University Hospital Regensburg, Regensburg, Germany; 13 Charité – Universitätsmedizin Berlin, Berlin, Germany; 14 Transplant Center, Department of Nephrology, Institute for Clinical and Experimental Medicine, Prague, Czech Republic; 15 Department of Nephrology, Hemodialysis, Apheresis and Kidney Transplantation, CHU-Grenoble-Alpes, Grenoble, France; 16 Department of Internal Medicine, Leiden University Medical Center, Leiden, The Netherlands; 17 Division of Nephrology, Department of Internal Medicine, University Medical Center Groningen, University of Groningen, Groningen, The Netherlands; 18 Division of Nephrology, Department of Internal Medicine, Medical University of Graz, Graz, Austria; 19 Ordensklinikum Linz GmbH Elisabethinen, Linz, Austria; 20 Institute for Advanced Social Studies, Spanish National Research Council, Madrid, Spain; 21 Department of Philosophy I, FiloLab-UGR, University of Granada, Granada, Spain; 22 Center for Medical Statistics, Informatics and Intelligent Systems, Medical University of Vienna, Vienna, Austria

**Keywords:** Kidney transplantation, Torque teno virus, Immunosuppression, Tacrolimus, Immunological monitoring, Personalised medicine, Infection, Graft rejection

## Abstract

**Background:**

Immunosuppression after kidney transplantation is mainly guided via plasma tacrolimus trough level, which cannot sufficiently predict allograft rejection and infection. The plasma load of the non-pathogenic and highly prevalent torque teno virus (TTV) is associated with the immunosuppression of its host. Non-interventional studies suggest the use of TTV load to predict allograft rejection and infection. The primary objective of the current trial is to demonstrate the safety, tolerability and preliminary efficacy of TTV-guided immunosuppression.

**Methods:**

For this purpose, a randomised, controlled, interventional, two-arm, non-inferiority, patient- and assessor-blinded, investigator-driven phase II trial was designed. A total of 260 stable, low-immunological-risk adult recipients of a kidney graft with tacrolimus-based immunosuppression and TTV infection after month 3 post-transplantation will be recruited in 13 academic centres in six European countries. Subjects will be randomised in a 1:1 ratio (allocation concealment) to receive tacrolimus either guided by TTV load or according to the local centre standard for 9 months. The primary composite endpoint includes the occurrence of infections, biopsy-proven allograft rejection, graft loss, or death. The main secondary endpoints include estimated glomerular filtration rate, graft rejection detected by protocol biopsy at month 12 post-transplantation (including molecular microscopy), development of de novo donor-specific antibodies, health-related quality of life, and drug adherence. In parallel, a comprehensive biobank will be established including plasma, serum, urine and whole blood. The date of the first enrolment was August 2022 and the planned end is April 2025.

**Discussion:**

The assessment of individual kidney transplant recipient immune function might enable clinicians to personalise immunosuppression, thereby reducing infection and rejection. Moreover, the trial might act as a proof of principle for TTV-guided immunosuppression and thus pave the way for broader clinical applications, including as guidance for immune modulators or disease-modifying agents.

**Trial registration:**

EU CT-Number: 2022-500024-30-00

**Supplementary Information:**

The online version contains supplementary material available at 10.1186/s13063-023-07216-0.

## Administrative information

**Table:** 

**Title {2a}**	A MULTICENTRE, PATIENT- AND ASSESSOR-BLINDED, NON-INFERIORITY, RANDOMISED AND CONTROLLED PHASE II TRIAL TO COMPARE STANDARD AND TORQUE TENO VIRUS-GUIDED IMMUNOSUPPRESSION IN KIDNEY TRANSPLANT RECIPIENTS IN THE FIRST YEAR AFTER TRANSPLANTATION: TTV GUIDE IT
**Trial registration {2a and 2b}**	EU CT-Number: 2022-500024-30-00. For details concerning the trial registration dataset, see Table 1.
**Protocol version (1)**	Version 4.0 submitted for approval 7.3.2022. Version 5.0. approved: Austria, 01.07.2022; the Czech Republic, 01.07.2022; France, 28.06.2022; Germany, 01.07.2022; the Netherlands, 30.06.2022; Spain, 08.07.2022. Version 6.0 submitted for amendment 15.07.2022 (primary reason: exclusion of SARS-CoV-2 infection and COVID-19 disease from the primary endpoint) and approved: Austria, 12.10.2022; the Czech Republic, 17.10.2022; France, 14.10.2022; Germany, 13.10.022; the Netherlands, 17.10.2022; Spain, 13.10.2022.
**Funding {4}**	The trial is fully funded by a Research and Innovation Action within the Horizon 2020 framework; project name: TTVguideTX; grant agreement number: 896932; project coordinator: Gregor Bond, MD, PhD. This trial is investigator-initiated. The funding source had no role in the design of this trial and will not have any role in its execution, analyses, interpretation of the data, or decision to submit results.
**Author details {5a}**	Frederik Haupenthal1, Jette Rahn2, Fabrizio Maggi3, Fanny Gelas4, Philippe Bourgeois4, Christian Hugo5, Bernd Jilma6, Georg A. Böhmig1, Harald Herkner7, Michael Wolzt8, Konstantin Doberer1, Matthias Vossen9, Daniele Focosi10, Hannes Neuwirt11, Miriam Banas12, Bernhard Banas12, Klemens Budde13, Ondrej Viklicky14, Paolo Malvezzi15, Lionel, Rostaing15, Joris I. Rotmans16, Stephan J.L. Bakker17, Kathrin Eller18, Daniel Cejka19, Alberto Molina Pérez20, David Rodriguez-Arias21, Franz König22, Gregor Bond1 1 Division of Nephrology and Dialysis, Department of Medicine III, Medical University of Vienna, Vienna, Austria frederik.haupenthal@meduniwien.ac.at, gregor.bond@meduniwien.ac.at, georg.boehmig@meduniwien.ac.at, konstantin.doberer@meduniwien.ac.at 2 Coordination Center for Clinical Trials, Faculty of Medicine Carl Gustav Carus, Technische Universität Dresden, Dresden, Germany Jette.Rahn@uniklinikum-dresden.de 3 Laboratory of Virology, National Institute for Infectious Diseases L. Spallanzani, Rome, Italy fabrizio.maggi63@gmail.com 4 bioMérieux SA, Centre Christophe Merieux, Grenoble, France philippe.bourgeois@biomerieux.com fanny.gelas@biomerieux.com 5 Universitätsklinikum Carl Gustav Carus an der Technischen Universität Dresden, Dresden, Germany Christian.Hugo@uniklinikum-dresden.de 6 Department of Clinical Pharmacology, Medical University of Vienna, Vienna, Austria bernd.jilma@meduniwien.ac.at 7 Department of Emergency Medicine, Medical University of Vienna, Vienna, Austria harald.herkner@meduniwien.ac.at 8 Clinical Trials Coordination Centre, Medical University of Vienna, Vienna, Austria michael.wolzt@meduniwien.ac.at 9 Division of Infectious diseases and Tropical Medicine, Department of Medicine I, Medical University of Vienna, Vienna, Austria matthias.vossen@meduniwien.ac.at 10 North-Western Tuscany Blood Bank, Azienda Ospedaliero-Universitaria Pisana, Pisa, Italy daniele.focosi@gmail.com 11 Department of Internal Medicine IV, Nephrology and Hypertension, Medical University Innsbruck, Innsbruck, Austria hannes.neuwirt@i-med.ac.at 12 Department of Nephrology, University Hospital Regensburg, Regensburg, Germany Bernhard.Banas@klinik.uni-regensburg.de Miriam.Banas@klinik.uni-regensburg.de 13 Charité – Universitätsmedizin Berlin, Berlin, Germany klemens.budde@charite.de 14 Transplant Center, Deptartment of Nephrology, Institute for Clinical and Experimental Medicine, Prague, The Czech Republic ondrej.viklicky@ikem.cz 15 Department of Nephrology, Hemodialysis, Apheresis and Kidney Transplantation, CHU-Grenoble-Alpes, Grenoble, France pmalvezzi@chu-grenoble.fr lrostaing@chu-grenoble.fr 16 Department of Internal Medicine, Leiden University Medical Center, Leiden, The Netherlands j.i.rotmans@lumc.nl 17 Division of Nephrology, Department of Internal Medicine, University Medical Center Groningen, University of Groningen, Groningen, The Netherlands s.j.l.bakker@umcg.nl 18 Division of Nephrology, Department of Internal Medicine, Medical University of Graz, Graz, Austria kathrin.eller@medunigraz.at 19 Ordensklinikum Linz GmbH Elisabethinen, Linz, Austria Daniel.Cejka@ordensklinikum.at 20 Institute for Advanced Social Studies, Spanish National Research Council, Spain albertononi@gmail.com 21 Department of Philosophy I, FiloLab-UGR, University of Granada, Granada, Spain dra@ugr.es 22 Center for Medical Statistics, Informatics and Intelligent Systems, Medical University of Vienna, Vienna, Austria franz.koenig@meduniwien.ac.at
**Name and contact information for the trial sponsor {5b}**	Medical University of Vienna, Spitalgasse 23, 1090 Vienna, Austria Sponsor-investigator: Gregor Bond, MD, PhD Telephone: +43 (0)1 40400-43910; Email: gregor.bond@meduniwien.ac.at
**Role of sponsor {5c}**	The sponsor is the institution of the principal coordinating investigator. The sponsor has the overall responsibility for the initiation and management of the trial.

## Introduction

### Background and rationale {6a}

#### Scientific problem

Kidney transplantation is the gold standard of treatment for patients with end-stage renal disease. After transplantation, immunosuppressive drugs are crucial for reducing the risk of organ rejection. Despite this desired effect, the compromised immunity of the recipient leads to an increased risk of infectious disease. Moreover, current immunosuppression regimens are unable to sufficiently control allorecognition, which leads to graft rejection [1]. Thus, the optimal management of immunosuppressive drug dosing requires a delicate balance between inadequate and excessive immunosuppression. At present, there is no diagnostic test or algorithm for the optimal guidance of immunosuppressive drug administration in clinical routine. Monitoring relies on the quantification of calcineurin inhibitor trough levels, mostly tacrolimus (TAC), in the peripheral blood, which correlate more closely with the risk of drug-related toxicity than with the effectiveness of immunosuppression [2, 3]. There is an urgent need for tools to personalise immunosuppression to reduce the risk of infectious disease and, at the same time, graft rejection.

#### Status of the research

While most of the proposed assays for the guidance of immunosuppression focus on graft rejection [4, 5], a useful approach would ideally predict both graft rejection and infectious disease [6]. To address this issue, two assays were proposed: (i) A test of leucocyte function, the QuantiFERON Monitor (Qiagen), was prognostic for infectious events but not for graft rejection in a cohort study including solid organ transplant patients [7]. (ii) In a randomised controlled setting, tailoring immunosuppression after liver transplantation via assessment of lymphocyte function using ImmuKnow® (Eurofins Viracor) resulted in fewer infectious events but had no influence on graft rejection [8]. Of note, the trial design precluded reliable analysis concerning the safety and efficacy of the ImmuKnow® assay. Currently, no further interventional trials in solid organ transplantation are registered for either of these products. Recently, no major safety signal was observed in a randomised controlled trial in paediatric kidney recipients testing the steering of immunosuppressive therapy by levels of virus-specific T-cells [9]. No difference was observed in rejection and infection, which were part of the secondary analysis. Of note, the complexity of virus-specific T-cell monitoring might pose an obstacle to further efficacy trials and introduction in the clinical routine.

#### Torque teno virus

Monitoring the torque teno virus (TTV) load in the peripheral blood is a promising new strategy for quantifying immune function [10–12]. TTV can be detected in up to 90% of healthy individuals and has not been described to cause any human disease. Peripheral blood copy numbers of TTV are associated with the function of the immune system of the host. The prevalence of TTV in patients after transplantation is around 99%, and the virus is unaffected by the conventional antiviral drug therapy used in the post-transplantation setting. TTV copy number is directly associated with factors determining immune function, including age, sex and the amount and type of immunosuppressive drugs administered to transplant recipients, and is thus indirectly associated with graft rejection and infectious disease.

#### Preliminary data

Sufficient evidence exists for a linear, robust and independent association between TTV load and all types of clinically overt and subclinical rejection, including T-cell-mediated rejection (TCMR), antibody-mediated rejection (ABMR) [13–19] and infectious events [14, 15, 17, 18, 20–25], including all common post-transplant pathogens in adult kidney transplant recipients (opportunistic infections, cytomegalovirus (CMV), BK virus (BKV) and bacterial infections). Applying the Vienna in-house PCR, an increased risk of rejection and infection was described for a TTV load outside of 6 to 8 log_10_ copies per millilitre (c/ml) in months 4 to 12 after kidney transplantation [14, 16, 19, 24].

Based on this background knowledge, we defined the TTV range targeted in the interventional group within the TTVguideIT study. First, we applied the ‘optimal’ TTV load of 6 to 8 log_10_ c/ml—as suggested by the literature—to all plasma samples from the Vienna kidney transplant cohort obtained in months 4 to 12 after transplantation; 41% showed a TTV load below this range and 29% above, thus exhibiting an uneven distribution. In contrast, an upper TTV load cut-off of 7.6 log_10_ c/ml scored 38% of the samples above the range, leading to a more equal distribution (unpublished data). Second, we re-quantified TTV in the Vienna kidney transplant cohort using the Vienna in-house PCR and the commercial PCR applied during the TTVguideIT study (the TTV R-GENE® assay) in parallel [26]. The two PCRs were highly associated, and the Vienna PCR was found to quantify TTV load as higher compared to the commercial PCR, with a mean difference of 1.4 log_10_ c/ml. Applying this difference to the range defined within step 1 (6 to 7.6 log_10_ c/ml), the optimal TTV range for the TTVguideIT study was set at 4.6–6.2 log_10_ c/ml.

### Objectives {7}

The primary objective is to demonstrate the non-inferiority of TTV-guided immunosuppression (arm T) compared to standard dosing (arm S) with respect to safety, tolerability and preliminary efficacy in stable adult kidney transplant recipients with low immunological risk in the first year after transplantation.

Non-inferiority can be concluded if the upper limit of a two-sided 95% confidence interval for the difference in the proportion of patients at month 9 after randomisation (V7; post-transplant month 12) between the two treatment arms is less than 10% points.

If non-inferiority is reached, the study is designed to test for superiority of TTV-guided immunosuppression (arm T) compared to standard dosing (arm S) in the same endpoint.

#### Initial null hypothesis

TTV-guided immunosuppression is not safe compared to standard dosing in stable adult kidney transplant recipients with low immunological risk in the first year after transplantation.

### Trial design {8}

Randomised, controlled, interventional, two-arm, non-inferiority, patient- and assessor-blinded, multinational, investigator-driven, phase II. Trial registry data are summarized in Table 1.

**Table 1:** Trial registry data

**Primary registry and trial identifying number**	EU CT-Number 2022-500024-30-00
**Date of registration in primary registry**	07.03.2022
**Source of monetary or material support**	Research and Innovation Action within the Horizon 2020 framework; project name: TTVguideTX; grant agreement number: 896932; project coordinator: Dr Gregor Bond.
**Sponsor**	Medical University of Vienna, Spitalgasse 23, 1090 Vienna, Austria
**Contact for public and scientific queries**	Assoc. Prof. PD. Dr Gregor Bond, PhD Nephrology and Dialysis, General Hospital Vienna Währinger Gürtel 18-20, 1090 Vienna, Austria email: gregor.bond@meduniwien.ac.at phone/fax: +43 (0)1 40400-43910/-43920
**Public title**	Personalised dosing of immunosuppression after kidney transplantation by measuring immune system functionality
**Scientific title**	A non-inferiority, randomised and controlled trial to compare the safety, tolerability and preliminary efficacy of standard and Torque Teno virus-guided immunosuppression in stable adult kidney transplant recipients with low immunological risk in the first year after transplantation
**Countries of recruitment**	Austria, the Czech Republic, France, Germany, the Netherlands, and Spain; 13 academic centres.
**Short title**	TTV GUIDE IT
**Health condition studied**	Kidney transplantation
**Intervention**	Active group: tacrolimus target set according to plasma Torque Teno virus load (target 4.6 to 6.2 log_10_ copies/mL) quantified by real-time PCR (TTV R-GENE) every 6 weeks Control: tacrolimus target set according to the local centre standard
**Trial schedule**	Screening: week 1 to month 3 post-transplantation; Randomisation: month 4 post-transplantation; Intervention: 9 months (last follow-up: last 6 weeks)
**Key inclusion and exclusion criteria**	Key inclusion criteria: recipient of a kidney allograft, adult (≥18 years of age), tacrolimus-based immunosuppression, TTV infection Key exclusion criteria: high immunological risk, no standard tacrolimus target immunosuppression according to the local centre definition
**Trial type**	Randomised, controlled, interventional, two-arm, non-inferiority, patient- and assessor-blinded, multinational, investigator-driven, phase II
**Date of first enrolment**	25^th^ of August 2022
**Planned end (last patient last visit)**	April 2025
**Target sample size**	260
**Randomisation and concealment**	1:1 randomisation; allocation concealment
**Primary endpoint**	A composite of one of the following: 1. Infectious disease event (diagnosis based on the Infectious Diseases Guidelines 2019 published by the American Society of Transplantation) requiring one of the following: - Application of anti-bacterial, -fungal, -viral and -protozoal drugs. - Reduction of immunosuppression. - Inpatient treatment SARS-CoV-2 infection with or without COVID-19 is excluded. 2. Allograft rejection detected upon indication biopsy, based on the Banff 2019 Kidney Meeting Report, including borderline rejection suspicious for T-cell-mediated rejection (BL TCMR). 3. Graft loss 4. Death
**Key secondary outcomes**	• Episodes of infection and graft rejection defined by the treating medical personnel • Estimated glomerular filtration rate (eGFR; current CKD-EPI and MDRD abbreviated) • Rejection detected by protocol biopsy at month 12 post-transplantation according to the Banff 2019 meeting report and molecular microscopy • *de novo* donor-specific antibodies • Health-related quality of life: SF-36 and MTSOSD-59R questionnaires • Drug adherence assessed according to paper-based assessment, MEMS® Buttons (AARDEX Group, Switzerland), BAASIS questionnaire, claimed prescriptions, psychological evaluation and tacrolimus trough level variability

## Methods: participants, interventions and outcomes

### Study setting {9}

Patients are being recruited at 13 academic tertiary care hospitals with high-volume kidney transplant units in Austria, the Czech Republic, France, Germany, the Netherlands and Spain. At each centre, appropriately trained medical staff will be available to provide the necessary standard of care for trial participants and to perform medical care specific to the trial and beyond. The trial sites have a local laboratory or cooperate contractually with external service providers to be able to perform the laboratory diagnostics required for the trial in a qualified manner (Table 1).

### Eligibility criteria {10}

#### Inclusion criteria

1. Recipient of a kidney allograft
2. Adult (≥18 years of age)
3. Post day 93 following transplantation
4. TAC-based immunosuppression
5. Standard target TAC trough level (as defined by the local centre)
6. Written informed consent

#### Exclusion criteria

1. High-risk HLA-incompatible transplantation (as defined by the local centre; e.g. pre-formed DSA and/or crossmatch conversion).
2. High-risk ABO-incompatible transplantation (as defined by the local centre).
3. Combined transplantation
4. History of HIV or active Hep B/C infection
5. Donor with a history of HIV or active Hep B/C infection
6. TTV load always below 4.6 log10 c/mL during the screening phase
7. No stable TAC trough levels achieved during the screening phase (as defined by the local centre).
8. Hypersensitivity to TAC or other macrolides and hypersensitivity to any excipients
9. Cyclosporine-, mTor inhibitor- or co-stimulation blocker-based immunosuppression
10. No standard immunosuppression (according to the local centre definition)
11. Treatment with T-cell-depleting drugs within 2 months before the randomisation (e.g. anti-thymocyte globulin. This intervention has been shown to reduce the TTV replication pool and thus produces false low values [27]. Additionally, immunological high-risk patients (e.g. with a history of recent severe TCMR) will be excluded.
12. Current infection or allograft rejection as defined by the primary endpoint
13. Biopsy-proven ABMR or BKV PCR ≥10^4^ c/ml (or corresponding U/mL) in the blood until randomisation
14. Unstable graft function: estimated glomerular filtration rate (eGFR) <25 mL/min/1.73m2 (this limit might be ignored if creatinine clearance is >25 mL/min/1.73m^2^) or rapid and relevant eGFR decline (as defined by the local centre); urinary protein/creatinine ratio >2000 mg/g or rapid and relevant increase (as defined by the local centre)
15. Advanced liver failure (Child–Pugh score C)
16. History of malignancy other than squamous cell carcinoma or basal cell carcinoma of the skin or carcinoma in situ or adenoma of the colon within the last 5 years unless in complete remission for at least 3 years
17. Leukopenia <2000/mm^3^ or neutropenia <1000/mm^3^
18. Unstable angina, cardiac decompensation with the necessity for inpatient treatment
19. Severe tremor (as defined by the local centre) due to TAC
20. Inability to complete study visits at the trial centre
21. Any state that excludes adherence to the trial protocol, such as serious medical or psychiatric illness, language barrier, alcohol or illicit substance abuse or non-adherence
22. Addictions or other illnesses that do not allow the person concerned to assess the nature and extent of the clinical trial and its possible consequences
23. Simultaneous participation in another interventional clinical trial
24. Pregnant or breastfeeding women
25. Women of childbearing potential, except women who meet one of the following criteria:
  - Post-menopausal (12 months’ natural amenorrhoea)
  - Postoperative (6 weeks after bilateral ovariectomy with or without hysterectomy, bilateral salpingectomy)
  - Regular and correct use of a contraceptive method with a Pearl Index <1% per year
  - Sexual abstinence
  - Vasectomy of the partner

#### Centres/investigators

All recruiting centres are university-based tertiary care centres with high-volume kidney transplant units. The local PIs at the participating trial sites are internationally recognised scientists in the field of kidney transplantation with a track record of the successful conduction of interventional clinical trials. The personnel involved in the clinical trial are qualified in accordance with the quality standards of ‘good clinical practice’. At each centre, appropriately trained medical staff will be available to provide the necessary standard of care for trial participants and provide medical care specific to the trial and beyond. The trial sites have a local laboratory or cooperate contractually with external service providers to be able to perform the laboratory diagnostics required for the trial in a qualified manner. The trial sites have Internet access to use web-based randomisation and complete data entry via an electronic case report form (eCRF).

### Who will take informed consent? {26a}

Potential study patients will be informed about the trial by the investigators and written informed consent will be obtained prior to inclusion.

### Additional consent provisions for the collection and use of participant data and biological specimens {26b}

Additional informed consent will be obtained for the sub-study ‘biobank’.

## Intervention

### Explanation for the choice of comparators {6b}

The primary objective of the study is to demonstrate the non-inferiority with respect to safety, tolerability and preliminary efficacy of TTV-guided immunosuppression compared to standard TAC dosing. Current evidence suggests an optimal TAC trough level target of 7 ng/ml for standard-risk kidney transplant recipients in the first year post-transplantation [2, 28]. However, great diversity exists in the centre-specific standards of care. In centres participating in the TTVguideIT study, the standard TAC target ranges from 3 to 7 ng/ml. Reluctance towards a standardised TAC target might reduce protocol adherence and recruitment rates. Thus, no study-specific TAC target was defined for the control group. A TAC target defined by local centres may reflect current practices more precisely than a fixed target.

### Intervention description {11a}

#### Active/interventional group

TAC trough level target range will be adapted by TTV load. TAC is approved for its intended use in this clinical study by all national competent regulatory authorities of the participating countries, and TTV load will be quantified in peripheral blood EDTA plasma by a CE-certified kit (TTV R-GENE®, bioMérieux, France) [12]. bioMérieux has set up TTV R-GENE® at all participating sites with a customised quality assessment programme [29]. The standard TAC packaging/labelling/dosage form as provided by the vendors will be used. There will be no restrictions pertaining to a certain TAC formulation or vendor/distributor. The TAC-containing drug will be obtained as usual by the subjects themselves from their local pharmacies.

**Fig. 1:**
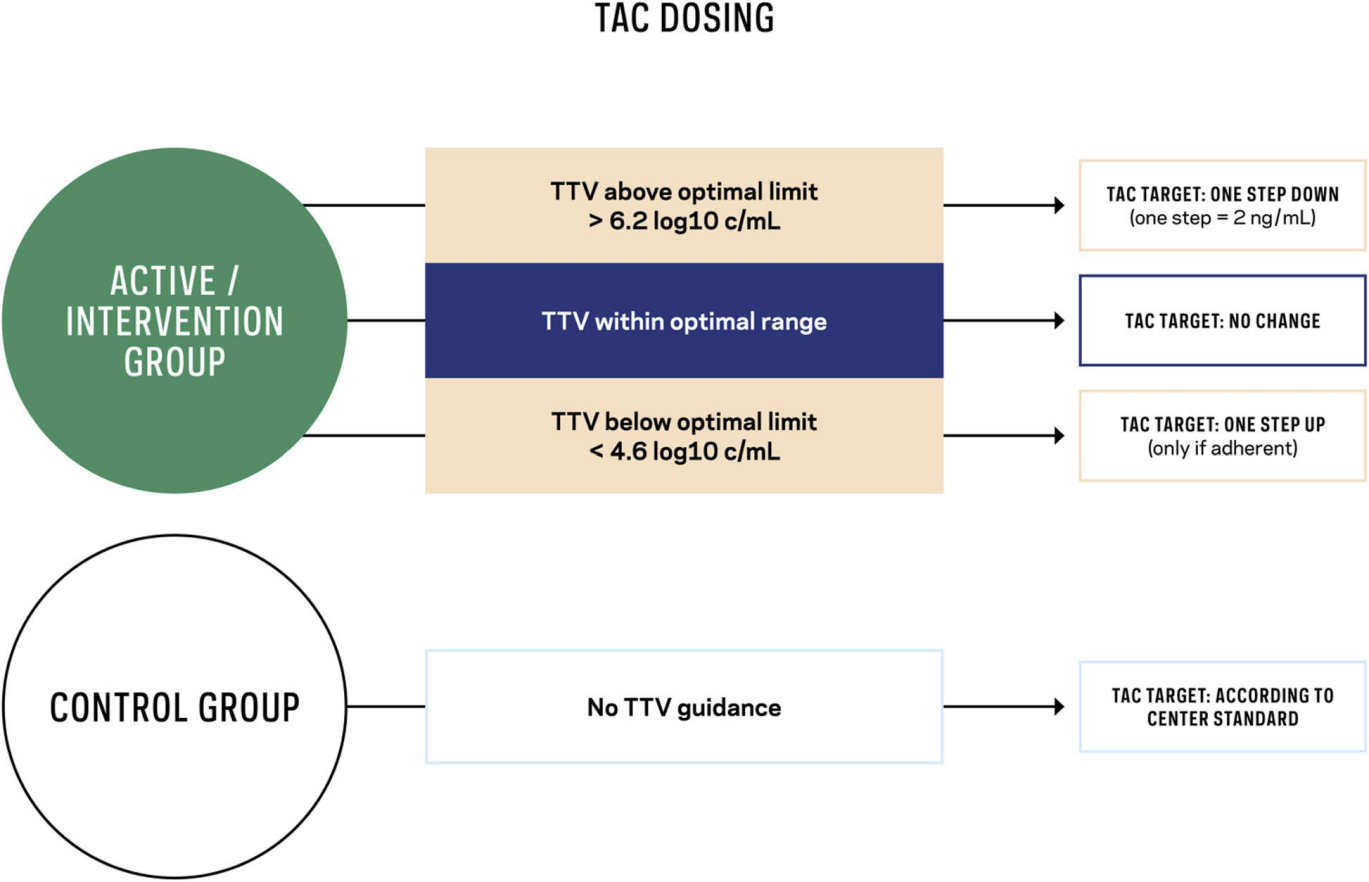
Tacrolimus (TAC) dosing during the interventional phase of the TTVguideIT study: In the active/interventional group, the tacrolimus trough level range will be adapted by torque teno virus (TTV) load. The target TTV load ranges from 4.6 log_10_ copies per millilitre (c/mL) to 6.2 log_10_ c/mL as the optimal range. If TTV is not within the optimal range, the TAC trough level target has to be adapted by one step up (only if the patient is adherent to TAC intake) or down compared to the current TAC trough level. One TAC trough level adaption step is defined as 2±1 ng/mL. Additional rules are detailed in the trial protocol. In the control group, TAC will be dosed according to TAC trough levels defined by the local centre standard

The intervention itself is restricted to a novel dosing strategy for TAC according to TTV load. TAC dosing according to the TTV target load is detailed in Fig. 1; the target TTV load has an optimal range of 4.6 to 6.2 log_10_ c/mL. If TTV is not within the optimal range, the TAC trough level target has to be adapted by one step up (only if the patient is adherent to TAC intake) or down compared to the current TAC trough level. One TAC trough level adaption step is defined as 2±1 ng/mL. Additional rules are detailed in the trial protocol.

#### Control group

TAC will be dosed according to TAC trough levels defined by the local centre standard. The TTV load will be accessed but not revealed to the investigator.

### Criteria for discontinuing or modifying allocated interventions {11b}

In addition to the standard criteria, the trial therapy has to be terminated prematurely for the following reasons: introduction of mTOR inhibitors, co-stimulation blockers or cyclosporine; necessity for significant additional long-term immunosuppression or immune modulation; necessity for long-term termination of TAC; and any condition that is not covered by the additional rules and needs a TAC target significantly higher or lower than the standard of care.

The trial therapy might be terminated prematurely for the following reasons: rejection as defined by the primary endpoint and severe infection (life- or organ-threatening). In these circumstances, the PI decides if TTV-guided TAC dosing will be stopped and the patient will continue the study following the protocol of the control arm.

### Strategies to improve adherence to interventions {11c}

Participant adherence to the trial protocol is expected to be high because the investigational medicinal product is used for routine clinical post-kidney-transplant care, study visits are implemented within routine outpatient care and no additional invasive procedures are necessary. Adherence will be improved by concise study information leaflets and calls to participants every 2 weeks performed by the study team. Medical adherence will be monitored prospectively using a patient diary. Additional electronic drug monitoring using MEMS® Buttons (AARDEX Group, Switzerland) on TAC blisters, the BAASIS© questionnaire (see Supplementary materials), claimed prescriptions, psychological evaluation (see Supplementary materials) and TAC trough level variability will be accessed retrospectively.

### Relevant concomitant care permitted or prohibited during the trial {11d}

At each centre, appropriately trained medical staff will be available to provide the necessary standard of care for trial participants and to provide medical care specific to the trial and beyond. Subjects are not allowed to receive cyclosporine-, mTor inhibitor- or co-stimulation blocker-based immunosuppression or significant additional long-term immunosuppression or immune modulation. This does not include therapy for organ rejection, polyoma virus-associated nephropathy or thrombotic microangiopathy.

### Provisions for post-trial care {30}

In any clinical situation, including emergencies, appropriate medical care will be provided. After the study, medical follow-up will be provided for all participants at the study centre or suitable facilities according to the local standard. In case of adverse events (AEs), all necessary medical care will be provided for all participants at the study centre or suitable facilities even after data collection for the research study is completed. On behalf of the sponsor, mandatory subject insurance according to national and EU regulations has been implemented for all trial subjects. This insurance covers all possible damages that the subject suffers directly or indirectly as a result of the investigational medicinal product or interventions in connection with the clinical trial.

### Outcomes {12}

#### Primary endpoint

The primary endpoint is designed to target the most relevant, frequent and significant consequences of too-intense or insufficient immunosuppression—infection and graft rejection—that might be reduced by personalised TTV-guided immunosuppression, along with the two main safety outcomes in kidney transplantation—graft loss and death.

The occurrence of a composite of one of the following during the interventional part of the study will be noted (V1 to V7; absolute numbers and percentages):

1. Infectious disease event (diagnosis based on the Infectious Diseases Guidelines 2019 published by the American Society of Transplantation) requiring one of the following:
  - Application of anti-bacterial, -fungal, -viral and -protozoal drugs.
  - Reduction of immunosuppression.
  - Inpatient treatment.

SARS-CoV-2 infection with or without COVID-19 is excluded.

(2) Allograft rejection detected upon indication biopsy, based on the Banff 2019 Kidney Meeting Report, including borderline rejection suspicious for T-cell-mediated rejection (BL TCMR).
(3) Graft loss
(4) Death

Routine calls every 2 weeks will be performed to detect infections treated outside the study centre. For the primary endpoint analysis, all episodes of infection and allograft biopsy will be re-assessed by personnel blinded to the randomisation code.

#### Secondary endpoints

- Single components of the primary outcome
- Episodes of infection and graft rejection scored by the treating medical personnel according to the primary endpoint (=main secondary endpoint).
- Severe infection (necessitating treatment in the inpatient or day-care ward) and severe rejection (excluding BL TCMR)
- All of the three abovementioned secondary endpoints including COVID-19
- Episodes of infection due to COVID-19
- eGFR (current CKD-EPI and MDRD abbreviated)
- Rejection detected by protocol biopsy at month 12 post-transplantation according to the Banff 2019 meeting report (including/excluding BL TCMR) and molecular microscopy

Protocol biopsies will not be performed as part of the study protocol but according to local centre standards.

- De novo donor-specific antibodies (DSA)
- Plasma TTV load
- TAC trough level and dose
- Unchanged, increased and decreased TAC trough target levels
- Health-related quality of life: Study Short Form 36 and the Modified Transplant Symptom Occurrence and Symptom Distress Scale-59 Items Revised (see Supplementary materials)
- Drug adherence according to paper-based assessment, MEMS® Buttons on TAC blisters, BAASIS questionnaire, number of actually claimed prescriptions by the patients, psychological evaluation and TAC trough level variability.
- AEs and serious adverse events (SAEs)
- Development of malignoma

The timing of the assessment of endpoints is detailed in Table 2.

**Table 2:** Trial-specific and routine procedures performed during the interventional trial

Post-TX month		1	2	3	4	5		8		11	12 to 13
Study week		−12 to −11	−8	−4	0	6	12	18	24	30	36
Study visit		−3	−2	−1	1	2	3	4	5	6	7 (FUP)
**Informed consent**		X									
**Inclusion/ exclusion criteria**		X			X						
**Intervention**					X	X	X	X	X	X	
**Medical history**		X			X						
**Vital signs**					X	X	X	X	X	X	X
**Physical examination, body weight**					X						X
**Pregnancy test**					X						X
**Medication**					X	X	X	X	X	X	X
**Adverse events**^**a**^						X	X	X	X	X	X
**Primary endpoint**^**a**^						X	X	X	X	X	X
**Laboratory workup**	**TTV R-GENE®**	X	X	X	X	X	X	X	X	X	X
	**TAC trough level**		X	X	X	X	X	X	X	X	X
	**Chemistry, CBC, vBGA, urine analysis**^**b**^		X	X	X	X	X	X	X	X	X
	**BKV, CMV**^**b**^				X	X	X	X	X	X	X
	**DSA**				X						X
**Biobanking**^**c**^	**Whole blood, serum, plasma, urine**	X	X	X	X	X	X	X	X	X	X
**Drug adherence**^**d**^	**MEMS® BUTTON**				X	X	X	X	X	X	X
	**BAASIS**				X	X	X	X	X	X	X
	**Patient diary**				X	X	X	X	X	X	X
	**Claimed prescription check**										X
	**Psychological evaluation**				X						X
**Quality of life**	**SF-36, MTSOSD-59R**				X						X
**Protocol biopsy**^**e**^					X						X

*BKV* BK virus, *CBC* Complete blood count, *CMV* Cytomegalovirus, *DSA* Donor-specific antibodies, *HLA* Human leucocyte antigen, *FUP* Follow-up, *MTSOSD-59R* Modified Transplant Symptom Occurrence and Symptom Distress Scale 59R, *rt-PCR* Real-time polymerase chain reaction, *SF-36* Medical Outcomes Study Short Form 36, *TAC* Tacrolimus, *TTV* Torque teno virus, *TX* Transplantation, *vBGA* Venous blood gas analysis

^a^ Check-ups concerning infections and other adverse events will be performed additionally via telephone calls every 2 weeks

^b^ Laboratory workup and virology screening will be performed not as part of the study protocol but according to local centre standards. However, according to the study protocol, at least the leucocyte count, creatinine, and urinary protein- and albumin-to-creatinine ratio will have to be assessed. Other major laboratory parameters and findings concerning CMV and BKV screening, including plasma CMV and BKV PCR performed routinely at the centres, will be noted

^c^ Biological material will be stored for additional immunological monitoring according to a sub-study

^d^ Adherence will be monitored during the trial by a patient diary and evaluated at every visit; other assessments of adherence will be evaluated retrospectively

^e^ Protocol biopsies (including molecular microscopy) will not be performed as part of the study protocol but according to local centre standards. However, the results of protocol biopsies obtained as part of the routine local centre standard will be noted. If part of the centre standard, the month 3 protocol biopsy has to be performed before V1

### Participant timeline {13}

**Fig. 2:**
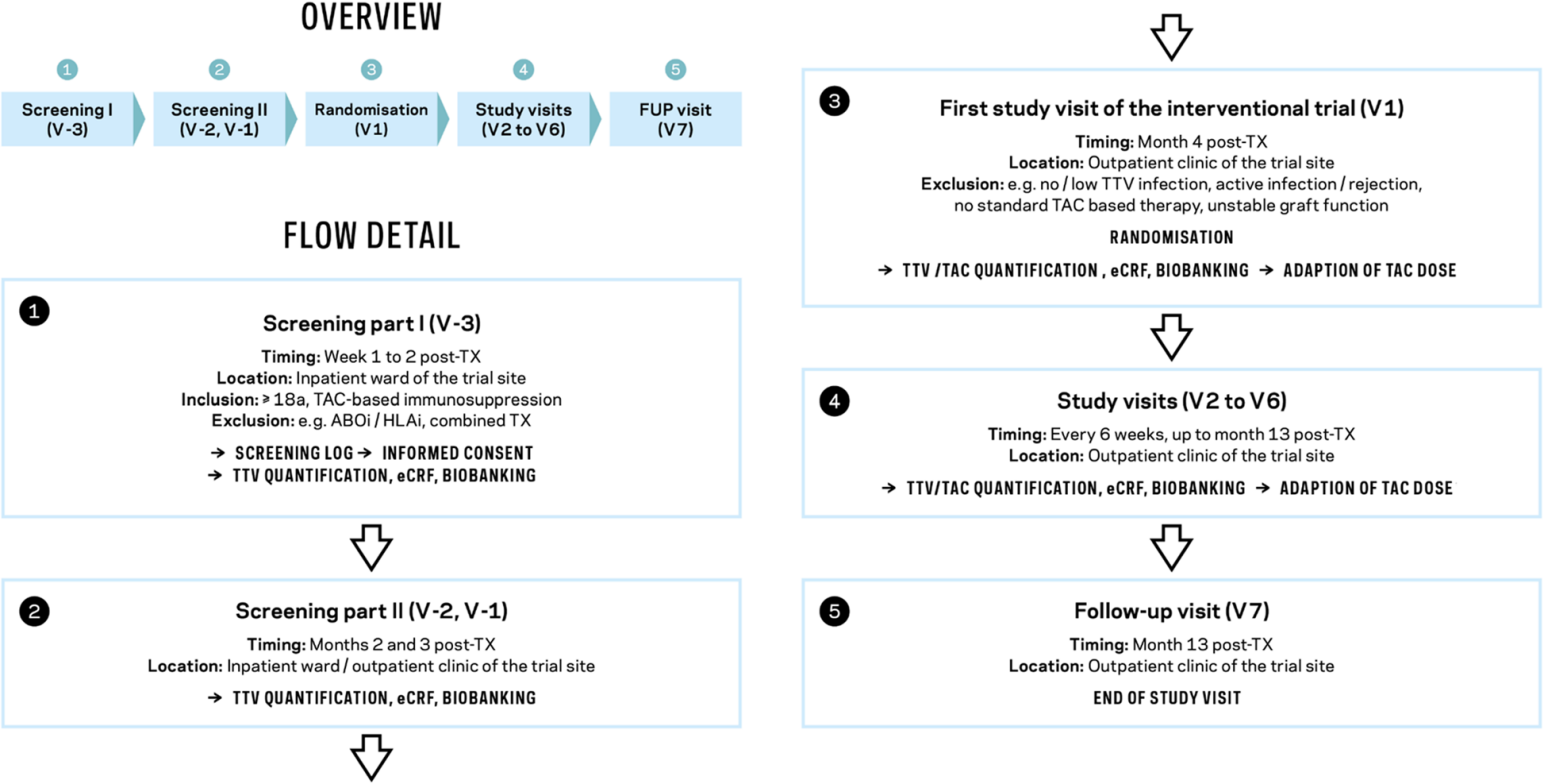
Study flow of the TTVguideIT study: Informed consent will be obtained from all consecutive adult recipients of a kidney allograft within the first 2 weeks after transplantation at the inpatient ward, and patients will be enrolled in the screening phase of the study (V**−**3). Subsequent screening visits will be in months 2 (V**−**2) and 3 (V**−**1) after transplantation. If all inclusion criteria are met and no exclusion criteria are present, participants will be enrolled in the clinical trial and randomised in month 4 after transplantation (V1). The TAC dose will be adjusted according to the TTV-guided TAC trough level target or the routine centre TAC trough level target. Participants will visit the outpatient clinic every 6 weeks, following the same procedure up to and including month 12 after transplantation (V2–V6). Follow-up will be performed until month 13 post-transplantation (V7)

**Fig. 3:**
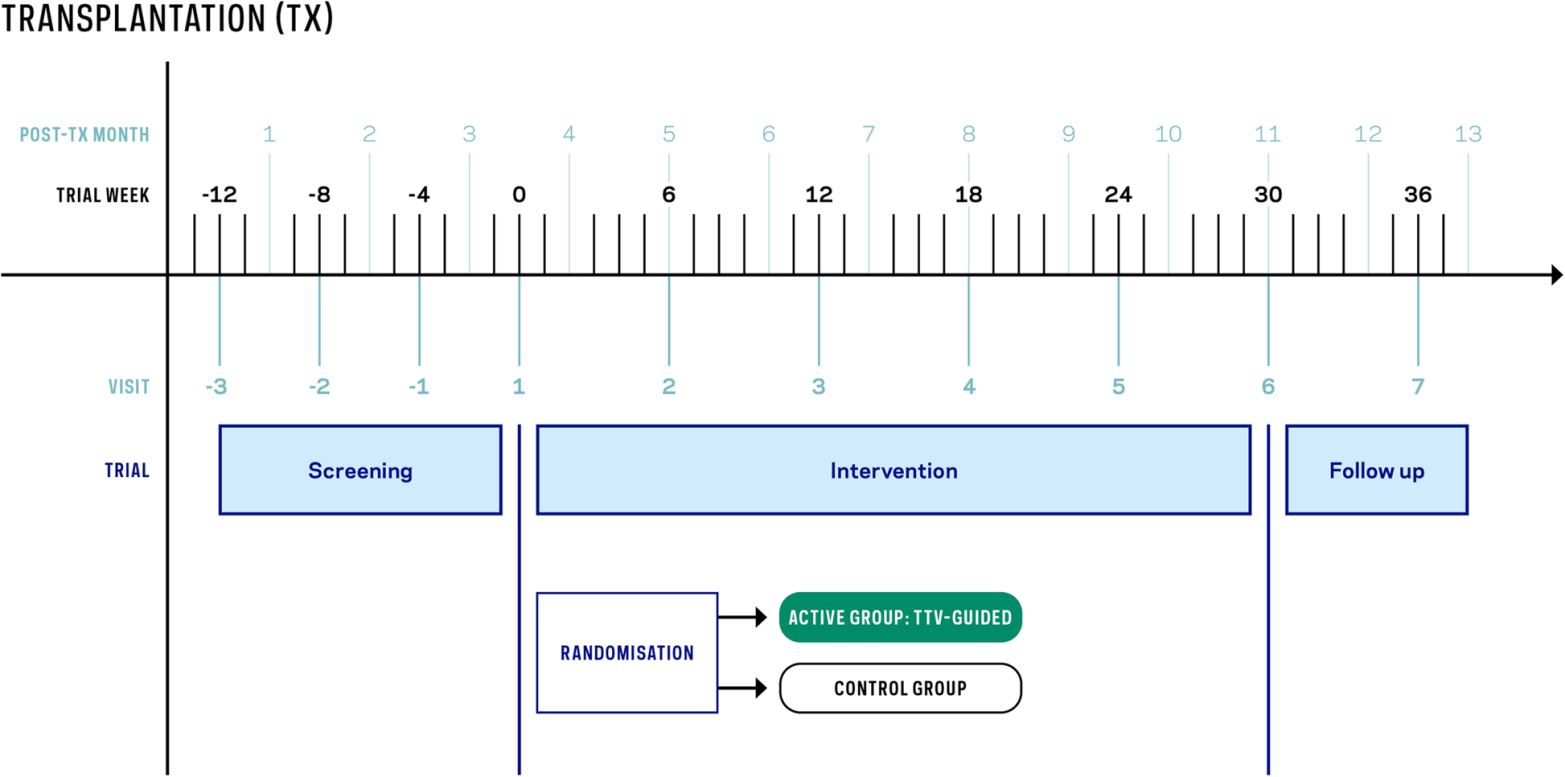
Trial design of the TTVguideIT study: Screening will start within the first 2 weeks after transplantation (V**−**3). Subsequent screening visits will be in months 2 (V**−**2) and 3 (V**−**1) after transplantation. Enrolment and randomisation will be in month 4 after transplantation (V1). During the interventional phase, visits will be every 6 weeks (V2–V6). Follow-up will be performed until month 13 post-transplantation (V7)

The participant timeline is detailed in Figs. 2 and 3. Informed consent will be obtained from all consecutive adult recipients of a kidney allograft within the first 2 weeks after transplantation at the inpatient ward, and the patient will be enrolled in the screening phase of the study (V−3). Subsequent screening visits will be in months 2 (V−2) and 3 (V−1) after transplantation. If all inclusion criteria are met and no exclusion criteria are present, participants will be enrolled in the clinical trial and randomised in month 4 after transplantation (V1). The TAC dose will be adjusted according to the TTV-guided TAC trough level target or the routine centre TAC trough level target. Participants will visit the outpatient clinic every 6 weeks, following the same procedure up to and including month 12 after transplantation (V2–V6). Follow-up will be performed until month 13 post-transplantation (V7). Trial-specific procedures are displayed in Table 2.

### Sample size {14}

A total of 260 patients will be included in the trial, 130 in the active group and 130 in the control group. For the sample size calculation, we analysed the occurrence of the primary endpoint in recipients of a kidney allograft transplanted between 1.1.2012 and 31.12.2018 at the Medical University of Vienna, applying the trial-specific inclusion and exclusion criteria. Overall, 40% of patients experienced the primary endpoint between months 3 and 12 after transplantation (unpublished data). Therefore, we assume a composite rate of around 40% in the standard dosing (arm S). It is expected that the TTV-guided immunosuppression (arm T) reduces the occurrence of the primary endpoint, and therefore rates between 20 and 30% are assumed to be reasonable.

The non-inferiority .margin has been fixed at 10% points. Thus when the sample size in each group is 120, a two-group large-sample normal approximation test of proportions with a one-sided 2.5% significance level will have 90.42% power to reject the null hypothesis that arm T is inferior to standard arm S in favour of the alternative hypothesis arm T is not inferior to arm S. Due to the planned observation period of 9 months and the special patient population, a rather low drop-out rate is expected of about 2–5%. Adjusting for potential drop-outs, the sample size is fixed with 130 patients per group, i.e., 260 in total. The sample size calculation was performed using N-Query Version 8.6.1.

### Recruitment {15}

Based on a feasibility study using a questionnaire and personal contact with the PIs, the potential participant recruitment rate was assessed for each centre. These rates were then reduced by 50%. Applying these re-calculated rates, a recruitment period of 12 months was calculated. We expect a high adherence to the anticipated recruitment rates in this investigator-driven study. All PIs were included in the trial design at an early stage and intensive training on the trial protocol has been performed. Recruitment rates will be continuously monitored by the Data and Safety Monitoring Board (DSMB). The funding allows for 12 additional months of recruitment. Moreover, potential alternative recruiting sites have been identified.

## Assignment of intervention: allocation

### Sequence generation {16a}

Participants will be allocated in a ratio of 1:1 using random permuted blocks with variable block sizes, stratified by recruiting centre.

### Concealment mechanism {16b}

The allocation sequence is implemented in the eCRF. No study personnel involved in the implementation of the allocation, patient care and endpoint assessment have access to the treatment randomisation code.

### Implementation {16c}

The allocation sequence will be created via the programme nQuery-Advisor® by the lead clinical trial unit (KKS TU Dresden). Patients will be enrolled and assigned to the intervention by the local study personnel.

## Assignment of intervention: blinding

### Who will be blinded {17a}

The study will be conducted using a single-blind design (participants blinded). The study design precludes blinding of the investigators. Thus, episodes of infection and allograft rejection will be re-assessed in a centralised manner by personnel blinded to the allocation to reduce assessment bias.

## Data collection and management

### Procedure for unblinding if needed {17b}

Unblinding is not necessary due to the study design (investigator not blinded to allocation).

### Plans for the assessment and collection of outcomes {18a}

Data relevant to the trial protocol are documented in the eCRF specially created for this trial (MACRO 4.0; Ennov, Paris, France). The data are checked for completeness, plausibility and consistency by means of programmed checks directly in the eCRF and by additional manual checks outside the eCRF. The data collection forms can be found in the Supplementary materials.

The following data will be obtained:

A detailed medical history including relevant baseline and FUP data until randomisation will be obtained. The following data will be noted: current medication, SARS-CoV-2 and influenza vaccination status, recipient sex, ethnicity and date of birth, type of renal disease, history of renal replacement therapy, history of prior transplantation, history of diabetes, history of major cardiovascular, immunological and oncological diseases and diseases currently requiring concomitant medication, CMV/ Epstein-Barr virus (EBV) serology, Hepatitis B and C serology, transplant date, donor type/age/sex, donor CMV/EBV serology, donor and recipient HLA (ideally 4-digit typing, but sufficient to define DSA), HLA mismatch, CMV/*Pneumocystis jirovecii* prophylaxis, initial immunosuppression, induction therapy, graft rejection, rejection therapy, de novo DSA, infections and diabetes mellitus post-transplant until randomisation. Blood pressure, heart rate, respiratory rate and body temperature will be assessed. A physical examination including the assessment of body size and weight, auscultation of the lung and heart and palpation of the abdomen will be performed.

A urinary dip β-HCG-based test will be performed. All immunosuppressive and antimicrobial prophylactic medication, medication for concomitant disease and medications for the treatment of AEs and the endpoint will be noted, including name, dose and schedule. All vaccinations will be noted. To detect TAC-related AEs and toxicity, complete blood count, blood chemistry and venous blood gas analyses will be performed according to routine clinical care. In addition, neuropsychiatric, sensorial, cardiovascular, bronchial, gastrointestinal, dermatological, musculoskeletal and urogenital alterations will be noted; specifically, any nausea, vomiting, constipation, diarrhoea, abdominal pain, tremor, paresthesia, headache, mental status change, and changes in motor and sensory functions, insomnia, asthenia, pain, oedema, shortness of breath, chest pain and palpitation will be assessed. AEs will also be assessed using patient diaries.

All infections have to be documented as AEs. In general, quality, onset and duration of symptoms, details on diagnostics, treatment and inpatient stay, and response to treatment have to be noted. Signs of infections that have to be documented as an AE include pain, night sweat, fever, chills, malaise, fatigue, diarrhoea (frequency and consistency of the stool), abdominal pain/cramps, dysuria, pollakisuria, alguria, urinary urgency/frequency, suprapubic pain, flank/allograft pain (on palpitation), cough, sputum (purulent, with blood), adventitious breath sounds on auscultation/palpation, shortness of breath and rapid/shallow breathing. Infectious disease workup will be performed according to local standards. However, some diagnostic workup should be performed to obtain comparable findings. In case of suspected infection, the investigators should perform a minimum diagnostic set including complete blood count, C-reactive protein, blood pressure, heart rate, respiratory rate and body temperature and document any altered mental status.

In addition, the following diagnostic sets should be applied:

- In patients with fever: blood and urine cultures, urinary dipstick and CMV PCR from the blood.
- In patients with suspected urinary tract infection: urinary dipstick and urine cultures; if available, ultrasound of the urinary track system.
- In patients with suspected respiratory infection: chest X-ray; if available, *Legionella/Haemophilus* and pneumococcus urinary antigen and sputum or nasopharyngeal swab multiplex PCR.
- In patients with suspected diarrhoea: stool cultures and CMV PCR from stool samples; if available, multiplex PCR from stool samples.

In general, all performed supporting diagnostic tests related to infections should be documented.

In addition to the trial visits, patients will be contacted at 2-week intervals via phone by the trial team, which will ask about episodes of infection (duration and onset of symptoms, diagnosis, treatment, and inpatient stay) and signs of infection (pain, fever, night sweats, cough, dysuria and diarrhoea).

Indication biopsies will be evaluated by applying standard centre methodology (including HE, PAS, Trichrome, S/AFOG, silver stain and immunohistochemistry). For protocol biopsies at month 12, an additional molecular evaluation will be performed by the Molecular Microscope Diagnostic System (MMDx) at the Alberta Transplant Applied Genomics Centre (ATAGC, University of Alberta, Edmonton, AB, Canada).

TTV will be quantified in peripheral blood EDTA plasma using the TTV R-GENE® (bioMérieux). bioMérieux has set up TTV R-GENE® at all participating sites with a customised quality assessment programme Quality Control for Molecular Diagnostics (QCMD; UK).

Tacrolimus trough level will be quantified according to the local centre standard.

If not part of the routine post-transplant care, DSA monitoring on the basis of a single fluorescence bead assay has to be done.

Tumour screening will be performed, not as part of the study protocol but according to local centre standards. Any oncological disease will be documented (type of tumour and date of diagnosis). Laboratory workup and virology screening will be performed according to local centre standards. However, according to the study protocol, at least leucocyte count, creatinine and urinary protein- and albumin-to-creatinine ratio will have to be assessed. Findings concerning CMV and BKV screening, including CMV and BKV PCR performed routinely at the centres, will be noted. For the assessment of health-related quality of life, the Medical Outcomes Study Short Form 36 and the Modified Transplant Symptom Occurrence and Symptom Distress Scale-59 Items Revised will be used. A questionnaire including sociological status, education, employment, addictions, critical life events and history of psychiatric illness will be administered. Diaries will be filled by the patients, including TAC intake and occurrence of AEs. Data collection forms can be found in the Supplementary materials.

### Plans to promote participant retention and complete follow-up {18b}

Participant retention is expected to be high because the study visits are incorporated within routine outpatient care and no additional invasive procedures are necessary. Retention will be improved by participant calls every 2 weeks performed by the study team. For participants who discontinue or deviate from the intervention, the study team will try to continue the study following the protocol of the control arm. The documentation of all subsequent visits should still be pursued.

### Data management {19}

In addition to the investigators, only persons authorised by the investigators are granted access to the eCRF. The accessed data may not be passed on to third parties. The scope of authorisation and the associated rights in the eCRF are controlled by data management via appropriately defined user roles. The data relevant to the trial protocol (including the data of trial subjects who were prematurely excluded from the trial) must be documented in the eCRF pseudonymously, promptly, legibly (without the use of abbreviations), completely and in accordance with the source data. Implausible values, which are displayed during data entry by programmed checks, must be checked by the trial site and corrected if necessary. If a correction is made in the eCRF, the reasons for it must be given. By means of the audit trail, all data and corrections are automatically logged with the date, time and username of the person entering the data. All old entries are retained and can be retrieved at any time.

A paper-based interim CRF is provided to the trial site as part of the investigator site file. This enables timely documentation in accordance with the protocol if the eCRF is not available (e.g. due to a system malfunction). The authorised persons will transfer the data immediately from the interim CRF to the eCRF as soon as the fault has been rectified.

The correctness and completeness of the documentation are confirmed by the authorised persons after each visit to the eCRF. Once the documentation for a subject has been completed, the PI finally confirms the documentation for this subject in the eCRF.

Queries by the sponsor or its representatives must be checked by authorised persons using the source data and answered directly in the eCRF. Any resulting corrections must be made in the eCRF. The data management plan describes the trial-specific approach of the individual processes for traceability and completeness of the relevant data (see Supplementary materials). The persons responsible for data management are responsible for data administration and processing. This is done by using electronic data capture software for clinical trials that meets the requirements of the applicable laws and guidelines (especially good scientific practice). The scope of database access and the associated authorisations are regulated by appropriate user roles. The data is checked for completeness, plausibility and consistency by means of programmed checks directly in the eCRF and by additional manual checks outside the eCRF. Any queries arising in the process are sent to the respective trial site directly in the eCRF. Queries as well as responses or corrections are made directly in the eCRF. Changes to the data are reproduced in the audit trail. The data is backed up on a daily basis. The data storage facilities are located in a locked room of the Medical Faculty of TU Dresden, to which only the responsible system administrators have access. At the end of the trial, the database will be closed after all data relevant to the trial protocol have been entered and all queries have been clarified. Subsequent changes to the data can only be made with the consent of the principal coordinating investigator. Records and documents related to the trial or distribution of investigational medicinal products (e.g. data collection forms, informed consent forms, drug accountability log and other relevant documents) must be retained at the trial site in accordance with the regulatory requirements but for at least 25 years. Subject records and other original data must be kept for the longest possible period permitted by the hospital, institution or private practice but for at least 25 years.

### Confidentiality {27}

The collection, transfer, storage and evaluation of personal data within this clinical trial is carried out in accordance with the applicable legal regulations (e.g. EU General Data Protection Regulation, EU reg. 2016/679 GDPR). Data collected during this clinical trial will be recorded on electronic data carriers, treated in strict confidence and only passed on to the sponsor of the trial for scientific evaluation and the assessment of AEs and the responsible supervisory authorities, the ethics commissions and the European database to verify the proper conduct of the trial and to evaluate trial results and AEs. To the extent necessary for the review of the clinical trial, authorised representatives of the sponsor (monitoring and auditing) and/or the regulatory authority, who are bound to secrecy, may inspect the personal data available at the trial site.

### Plans for the collection, laboratory evaluation and storage of biological specimens for genetic or molecular analysis in this trial/for future use {33}

An additional informed consent will be obtained for the sub-study ‘biobank’. Participation in the sub-study is not a prerequisite for participation in the TTVguideIT study. Within this sub-study, biological material (serum, plasma, whole blood and urine) will be sampled and stored centrally at the Medical University of Vienna for further immunological monitoring, including but not restricted to further DSA assessment, donor-derived cell-free DNA, gene expression, and urinary TTV and chemokines.

## Statistical methods

A general description of the statistical methods to be used to analyse this study is given below. More details will be provided in the statistical analysis plan (unpublished data).

### Statistical methods for the primary and secondary outcomes {20a}

#### Study objective

The main goal of this study is to demonstrate that the TTV-guided immunosuppression (arm T) is non-inferior with respect to safety compared to standard dosing (arm S) in stable adult kidney transplant recipients with low immunological risk in the first year after transplantation.

#### Non-inferiority hypothesis

The primary objective is to demonstrate the non-inferiority of TTV-guided immunosuppression (arm T) compared to standard dosing (arm S) in stable adult kidney transplant recipients with low immunological risk in the first year after transplantation. Non-inferiority can be concluded if the upper limit of a two-sided 95% CI for the difference in proportion of patients at month 9 after randomisation (= Visit 7; post-transplant month 12) between the two treatment arms is less than 10% points. Let pT and pS be the proportion (in %) of participants in the arm T and arm S, respectively, then the primary statistical hypothesis can be formulated as follows:

$$\textrm{H}0:\textrm{pT}-\textrm{pS}\ge 10\%\textrm{versus}\ \textrm{H}1:\textrm{pT}-\textrm{pS}<10\%$$

#### Superiority hypothesis

If non-inferiority is reached, the study will demonstrate superiority of TTV-guided immunosuppression (arm T) compared to standard dosing (arm S) in the same endpoint by testing the hypothesis:

$$\textrm{H}0:\textrm{pT}-\textrm{pS}\ge 0\ \textrm{versus}\ \textrm{H}1:\textrm{pT}-\textrm{pS}<0$$

#### Definition of evaluation populations

Different analysis sets are defined as follows:

### Modified intention to treat

The modified intention-to-treat analysis set includes subjects who were randomised. According to the intent to treat principle, subjects will be analysed according to the treatment they have been assigned to during the randomisation procedure. The modified intention includes all patients who are eligible for the study and with at least one TAC evaluation (and potential adaption) during visit 1 will be included in the analysis.

### Per protocol

The per protocol analysis set comprises all subjects who received study intervention and did not critically or majorly violate the protocol in a way that might affect the evaluation of the effect on the primary objective. A list of potential protocol deviations is given in the study protocol in section 6.14.

### Adherent set

Medical adherence will be defined by patient diary; see main protocol section 5.10.

#### Analysis of baseline parameters and concomitant medications

Baseline parameters, medical history and concomitant medication will be documented during screening and throughout the trial until the last follow-up visit. Enrolment, protocol deviations and discontinuations from the study drug and the study will be summarised. Demographics (e.g. age, race, ethnicity and sex) and medical history and concomitant medication will also be summarised by treatment group. For qualitative variables (e.g. sex), absolute (*n*=*x*) and relative frequencies will be calculated per treatment group. Data will be visualised by bar plots. For quantitative data (e.g. age), the number of valid observations (*n*=*x*), mean, standard deviation, standard error, median, minimum and maximum will be calculated for each treatment group and each time point separately. Data will be visualised by spaghetti plots (showing individual patient profiles over time), boxplots and histograms.

#### Primary endpoint

The occurrence of the primary composite endpoint will be presented per treatment group as absolute numbers and percentages. The difference of the occurrence of the primary endpoint between the two treatment groups and a two-sided 95% confidence interval will be calculated. Non-inferiority will be concluded if the upper limit of a two-sided 95% confidence interval for the difference in proportion of patients at month 9 after randomisation between the two treatment arms is less than 10% points. Superiority will be concluded if the confidence interval excludes 0. As additional sensitivity analyses confidence intervals will be calculated being adjusted for study stratifications (such as centres) using Cochrane-Mantel-Haenszel weights. Also logistic regression models will be used using additional factors (such as sex) and covariate (such as age at randomisation). To explore the composite endpoint further, time-to-event endpoint considering the time from randomisation till time of first event will be analysed. We will visualise these data by presenting Kaplan-Meier curves for each treatment arm. Furthermore, Cox-regressions models will be performed adjusting for the same factors and covariates as used in the logistic regression models. To address the repetitive nature of the events used in the definition of the primary endpoint, we will also perform supportive analyses for recurrent event data. We will fit a negative binomial regression model for the events of the composite endpoints accounting for the time a patient is under risk. Furthermore, we will perform the counting process model of Andersen-Gill and frailty models. To assess the impact of the individual components of the composite endpoints, each component will be analysed descriptively.

#### Secondary endpoints

For binary secondary endpoints (such as rejection), absolute (*n*=*x*) and frequencies in percentage (%) will be calculated per treatment group. 95% confidence intervals will be calculated for rates, if appropriate. Such data will be visualised with bar charts. If appropriate, logistic regression models will be applied using treatment as independent factor. Furthermore, the model will be adjusted for sex and age. For time-to-event endpoints (such as overall survival), Kaplan-Meier plots will be provided. The two-group will be compared with log-rank tests. Additionally, Cox-regressions models will be performed adjusting for additional factors as described for the primary analysis. For recurrent events (such as infections), the appropriate (survival) methods will be applied. This includes the negative binomial regression models, Anderson and Gill models and Frailty models. Hazard ratios and corresponding two-sided 95% confidence intervals will be reported. Continuous secondary endpoints such as laboratory values or ‘quality of life’ will be summarised by mean, standard deviation, median, first and third quartiles, minimum and maximum for each treatment arm separately. If a continuous endpoint is measured only at one-time point after randomisation, it will be analysed using an analysis of covariance using the factor treatment group adjusting for the factors sex and the covariate age (in years). Mean estimates will be provided, together with their corresponding two-sided 95% confidence intervals. If repeated measurements are available for several visits after randomisation, a mixed model for repeated measurements will be performed using patient as random factor and treatment arm as fixed factor. If baseline values are collected, they will be included as covariate in the analysis of covariance and mixed model for repeated measurements.

#### Multiple testing

For the primary endpoint, we will use a hierarchical testing procedure. This means first we will test the non-inferiority hypothesis at a one-sided alpha of 2.5%. After non-inferiority can be demonstrated, superiority will be tested also using a one-sided alpha of 2.5%. The secondary endpoints include the components of the primary outcome and are needed to support the interpretation of the potential effects in the primary endpoint. The tests for these comparisons would require larger sample sizes to achieve the required power. This was the reason why a composite endpoint has been chosen and these analyses are considered supportive only without further multiplicity correction. For the analyses of all secondary endpoints, two-sided *p*-values and two-sided 95% confidence intervals will be reported.

### Interim analyses {21b}

There will be no interim analysis for efficacy. The DSMB will monitor the trial for safety purposes. To detect safety signals in a timely manner, the board members of the DSMB will be instructed to perform safety analyses after 65 and 130 included patients (if there are safety issues, the DSMB may ask for a higher frequency). SAEs and the primary outcome will be analysed according to the randomisation sequence. The DSMB may suggest stopping the trial if the overall pattern of related SAEs supports a major safety signal. For this trial, no statistical stopping rules will be used. The clinical trial is embedded in an EU Horizon2020-sponsored project (TTVguideTX). Thus, premature termination of the clinical trial will first be discussed by the project’s core team (project steering committee including the sponsor of the TTVguideIT study) and has to be decided on in the general assembly including all project partners. In case of premature termination of trial therapy according to the active group for a patient, the patient will continue the study following the protocol of the control arm. Further visits and trial-specific procedures will be continued. The documentation of all subsequent visits should still be pursued. Should the study be discontinued prematurely for a patient, all study materials will be retained. Data will be collected to the point of withdrawal and used for the intention-to-treat analysis if the subject consents. There will be no replacement of withdrawn subjects. For subjects withdrawing prematurely from the clinical trial, a final visit should be sought.

### Methods for additional analyses (e.g. subgroup analyses) {20b}

The primary analysis will be performed on a modified intention-to-treat principle. Sensitivity analyses will be performed according to intention-to-treat and per protocol analysis, and the dataset will be restricted to adherent patients. Subgroup analysis will be performed in patients at risk for immunological (re-transplantation) and infectious (diabetes mellitus) events and according to age group (>55 years of age), gender and study centre. If appropriate, these factors will be included as additional covariates in the regression models as described above.

### Methods in analysis to handle protocol non-adherence and any statistical methods to handle missing data {20c}

Medical adherence will be defined by patient diary (see main protocol section 5.10). Adherent patients will be analysed within the adherent set. For the primary analysis, missing values will be considered treatment failures in the composite primary endpoint. Additionally, meaningful missing values will be imputed by statistical models for the data according to the underlying mechanism of missing data. Sensitivity analyses will be performed for imputed data.

### Plans to give access to the full protocol, participant-level data and statistical code {31c}

The full trial database and analysis dataset will be reported to the competent regulatory authorities and, as far as possible, made available to the Open Research Data Pilot (https://ec.europa.eu/research/participants/docs/h2020-funding-guide/cross-cutting-issues/open-access-dissemination_en.htm).

## Oversight and monitoring

### Composition of the coordinating centre and trial steering committee {5d}

#### Principal Coordinating Investigator

Assoc. Prof. PD. Dr Gregor Bond, PhD

Nephrology and Dialysis, General Hospital Vienna

#### Biometrics

Assoc. Prof. PD. Dr Franz König

Medical University of Vienna

Center for Medical Statistics, Informatics, and Intelligent Systems

Section for Medical Statistics

#### Trial coordination

Dr. rer. nat. Roland Pfeiffer

Medizinische Fakultät der TU Dresden

KKS Dresden

#### Pharmacovigilance

Barbara Djawid

Medizinische Fakultät der TU Dresden

KKS Dresden

#### Monitoring

Dr. rer. nat. Roland Pfeiffer

Medizinische Fakultät der TU Dresden

KKS Dresden

#### Data management

Sandra König

Medizinische Fakultät der TU Dresden

KKS Dresden

#### Biobank

Mag. Dr. med. univ. & scient. med. Helmuth Haslacher, BSc, BA

MedUni Wien Biobank

General Hospital Vienna

The trial management team providing day-to-day support for the trial consists of the project coordinator, the trial manager and the project administrators. They will meet on a regular basis every month or ad hoc if necessary.

#### Steering committee

The TTVguideIT trial is part of the TTVguideTX project. For the TTVguideIT trial, no trial-specific Steering Committee and Management Committee has been defined. Instead, the trial will be managed by the TTVguideTX project management structures. The project Steering Committee includes the Project Management Team (Project Coordinator and Administrative Manager) and the Work Package Leaders of the TTVguideTX project and meets every 6 months.

#### Patient Advisory Board

The Patient Advisory Board is made up of patients’ representatives and will be consulted to make the developments in the healthcare sector patient-friendly and to take the views and needs of patients into account as much as possible throughout the trial.

#### Scientific Advisory Board

The Scientific Advisory Board includes independent experts in transplant nephrology, clinical virology, statistics and research study design. The Scientific Advisory Board will receive regular project reports and will be consulted by the Steering Committee on scientific problems to discuss solutions to problems and the further progress of the project.

#### Ethics and Governance Council

The independent Ethics and Governance Council includes experts in bioethics and law. The role of the Ethics and Governance Council is to provide independent external supervision and advice regarding the ethical and legal aspects of the trial. The Ethics and Governance Council will monitor the procedures in place for the trial to ensure the application of the highest ethical standards, thus safeguarding patients’ interests and rights.

### Composition of the data monitoring committee and its role and reporting structure {21a}

The external DSMB is independent of the sponsor and competing interests. The DSMB will monitor and protect patient safety throughout the clinical trial, with an emphasis on progress, safety data and critical efficacy endpoints according to the DAMOCLES group. Members of the DSMB include two transplant nephrologists and one biostatistician. The DSMB will report to the steering committee, management team and sponsor. Details concerning the DSMB are outlined in the DSMB charter (not finalised yet).

### Adverse event reporting and harms {22}

All AEs must be documented in the subject record, and appropriate medical treatment must be provided. The investigator will assess the causal relationship between the study drug and the AE. The investigator shall notify the sponsor immediately of the occurrence of an SAE.

The sponsor must document all AEs reported to him in detail and, upon request, submit these to the member states concerned. The sponsor must inform the member states concerned and the investigators involved in the clinical trial about every suspected unexpected serious adverse reaction that comes to his attention immediately. For the duration of the clinical trial, the sponsor must submit to the member states concerned, once a year or upon request, a list of all suspected cases of serious adverse drug reactions that have occurred during the trial as well as a report on the safety of the subjects (Development Safety Update Report). Any pregnancy that occurs during study participation must be reported to the sponsor immediately. Pregnancy complications and elective terminations for medical reasons must be reported as an AE or SAE. Any SAE occurring in association with a pregnancy brought to the investigator’s attention after the subject has completed the study and considered by the investigator as possibly related to the investigational product must be promptly reported to the sponsor. In addition, the investigator must attempt to collect pregnancy information on any female partners of male study subjects who become pregnant while the subject is enrolled in the study.

### Frequency and plans for auditing trial conduct {23}

There are no planned audits on behalf of the sponsor. Inspections can be carried out by the responsible member state concerned in accordance with the regulations (EU No. 536/2014). In case of an announcement of an inspection, the trial site must inform the sponsor immediately.

### Plans for communicating important protocol amendments to relevant parties {25}

Changes in the test conditions shall only be made after mutual agreement between the PIs and the sponsor. Any change to the procedure laid down in the trial protocol must be made in writing, stating the reasons for the change, and signed by all persons responsible for the trial. The changes are then considered to be part of the protocol. Where necessary (e.g. in the case of a change in the investigational medicinal product dosing scheme or other significant changes that indicate a direct impact on the safety of the trial subjects), the consent of the ethics committees and/or regulatory authorities responsible as well as the investigator to the protocol amendments shall be obtained, and the amendment shall be submitted to the regulatory authority.

### Dissemination plans {31a}

All essential results of the clinical trial will be submitted to the responsible higher federal authority and the ethics committee within 1 year after the end of the clinical trial. In addition, the results and, if necessary, other necessary information will be published in the European database. Links to publications will be provided to the trial registry. The trial results will be reported on the trial webpage and to the institutional review boards, the funding agency and the consortium partners of the Horizon2020 project. The full trial database and analysis dataset will be reported to the competent regulatory authorities and, as far as possible, made available to the Open Research Data Pilot. All results will be published open access. Wherever possible, the ‘gold’ open access route will be preferred. If the gold route is not feasible, the green route with self-archiving will be selected.

A dedicated work package within the TTVguideTX project will cover the dissemination and communication of the study results according to a dedicated roadmap targeting a diverse group of stakeholders by target-specific channels.

## Discussion

### Blinding

Blinding of the investigators would necessitate blinding towards the TTV load in the active group and towards the TAC trough levels in both groups. TAC dosing would have to be done centralised. It is unlikely that such trial design would be accepted by the investigator. Thus, we decided against blinding of the investigators.

### Infectious events as primary and secondary endpoints

The primary endpoint is designed to detect consequences of too-intense immunosuppression that might be reduced by personalised TTV-guided immunosuppression. Infectious disease events are scored by assessors blinded to the allocation sequence. Diagnosis is based on the Infectious Diseases Guidelines 2019 published by the American Society according to the data provided in the eCRF. Assessors have the possibility to post queries to the investigator in case of missing information. Investigators are advised to not unblind investigators within their correspondence. Any insignificant treatment, e.g. topical antifungal treatment of minor localised cutaneous disease, or supportive treatment during viral respiratory infections, e.g. antiseptic lozenge, is excluded. Non-indicated therapy, e.g. antibiotic treatment in viral respiratory or gastrointestinal infection, is excluded. Increase of prophylactic treatment dose, e.g. in CMV syndrome, is included.

SARS-CoV-2 infection with or without COVID-19 is excluded. SARS-CoV-2-positive test results still have a high incidence, with only minor differences between healthy subjects and recipients of a kidney transplant. Thus, it is unlikely that TTV-guided immunosuppression will alter the rate of infection and likely that many trial subjects in both the active and the control group will hit the primary endpoint due to COVID-19, potentially substantially reducing the power to discriminate the effect of TTV-guided immunosuppression. A high rate of COVID-19 in both groups would likely lead to non-inferiority of TTV-guided immunosuppression, but the safety of TTV-guided immunosuppression would still be unknown, rendering the trial futile.

Episodes of infection scored by the treating medical personnel according to the primary endpoint are the main secondary outcome. In the routine care of kidney transplant recipients, the diagnosis and treatment of infection and graft rejection sometimes do not follow current guidelines. However, such assessments might outperform guideline-directed management in some cases. Thus, this endpoint might reflect the true rate of clinically significant infection and graft loss more accurately. However, due to the fact that investigators are not blinded towards the treatment allocation, this endpoint has a risk of assessment bias and cannot be used as primary endpoint.

Infectious disease workup will be performed according to local standards. However, the investigators are asked to perform a standardised workup. For the expected main infectious events, the following parameters are of special interest: (a) for urinary tract infections, results on urinary dip stick and cultures and imaging; (b) for respiratory infections, results on imaging, microbiological and virologic workup including light microscopy, cultures, cytokine release assays, antigen tests and PCRs from blood, urine and respiratory material, albumin, respiratory rate, heart rate, peripheral oxygen saturation, arterial Ph and neurological status; (c) for diarrhoea, results on imaging and microbiological and virologic workup including cultures, antigen tests and PCRs; (d) for polyma virus infection, results on blood and urine PCR, decoy cells and kidney graft biopsy results and (e) for CMV infection differential blood count, liver enzymes (to differentiate CMV viremia from CMV syndrome), results on imaging and PCRs from liquor, urine, blood, stole and respiratory material.

### Tacrolimus dosing in the active group

If TTV is not within the optimal range, the TAC trough level target has to be adapted by one step up or down compared to the current TAC trough level. If TTV is below the optimal TTV range, the TAC trough level target has to be increased by one step compared to the current TAC trough level, and if TTV is above the optimal TTV range, the TAC trough level target has to be decreased by one step compared to the current TAC trough level. One TAC trough level adaption step is defined as 2 ng/mL (investigators are allowed to target a range of ±1 ng/mL; thus one step might be within a minimum of 1 ng/mL and a maximum of 3 ng/mL).

#### Dosing example

The patient has a current TAC trough level of 7.2 ng/mL. The TAC dose has to be adapted to target 5.2 ng/mL TAC trough level (= 4.2 to 6.2 ng/mL range) if the current TTV load is above the optimal range limit of 6.2 log10 TTV c/mL and adapted to 9.2 ng/mL TAC trough level (=8.2 to 10.2 ng/mL range) if the current TTV load is below the optimal range limit of 4.6 log10 TTV c/ml, respectively.

#### Additional rules

All changes in TAC dose (including all non-study visits) must be performed according to the study protocol and the current TAC trough level target. TAC adaptions have to be made within 48 h after the receipt of the TTV load. The lowest TAC trough level target is 3.5 ng/mL, and the highest is 10 ng/mL. The treating physician can request additional TAC trough levels at any time (also on non-study visits) but TTV load will only be assessed and TAC trough level target changes only performed at the study visits. If non-adherence is suspected, no increase in TAC trough level target will be performed: e.g. a failure of TAC intake more than once per week on average for b.d. and once every 2 weeks on average for o.d. formulation detected by the patient diary. In this case, the patient should be educated about medical adherence (e.g. psychological counseling) and the target TAC trough level will not be changed according to TTV load until the next study visit.

#### Hints for individualization of TAC dosage

One TAC trough level adaption step is defined as 2 ng/mL (investigators are allowed to target a range of ± 1 ng/mL; thus one step might be within a minimum of 1 ng/mL and a maximum of 3 ng/mL). This allows for additional individualization and accounts for limited granularity of TAC dosage formulation. Additional individualization might be necessary in the following clinical scenarios: Absolute changes in TAC dose might have different effect sizes on the TTV load. A change >20% in TAC dose is expected to show significant changes in the TTV load. Thus the upper range of 3 ng/mL might be targeted in case of high TAC intake and the lower range of 1 ng/ml in case of low TAC intake. Significant changes in TTV load are expected 6 weeks (= 1 visit) after a significant change in immunosuppression has been performed. However, the full effect might take up to 12 weeks (= 2 visits). If a change in the TAC target has been performed at the visit before and another TAC change in the same direction is suggested at the current visit, the lower TAC step range of 1 ng/mL might be targeted. Simultaneous relevant changes in immunosuppression other than TAC might influence the subsequent TTV load and the risk of infection/rejection, e.g. if mycophenolic acid has to be stopped and the TTV load indicates a TAC decrease, a TAC target change of 1 ng/mL might be performed. The peak TTV level is at month 3 to 4 post-transplantation; thereafter, the TTV load shows a gentle decline towards month 12 post-transplantation. If the TTV load indicates a reduction in the TAC target at visit 1, but the TAC level is within the local centre range, a TAC target reduction of 1 ng/mL might be performed.

#### General rules

No change in TAC dosing should be performed if the TAC trough level does not reflect a steady state, e.g. due to the following: low TAC level due to a missed dose or earlier intake than usual the day before the blood draw. High TAC level due to an intake at the morning before the blood was drawn or later than usual at the day before the blood draw. Suspected drug or dietary interaction with TAC. Suspected high TAC levels due to diarrhoea. In these cases, the TAC trough level has to be repeated within a week. If TAC level is back to a steady state, TAC dosing has to performed according to the allocated treatment group. Corticosteroids and antimetabolites will not be dosed according to the TTV level. Centres are encouraged to keep the antimetabolite dose stable.

## Eligibility criteria

Randomisation starts after day 93 following transplantation, because TTV load is not stable until month 4 and optimal TTV range has not been defined for the early post-transplant period. Patients on cyclosporine-, mTor inhibitor- or co-stimulation blocker-based immunosuppression cannot be included, because no sufficient data are available for optimal TTV load in these patients. An optimal TTV target is also not defined for patients with significant additional long-term immunosuppression or immune modulation (e.g. disease-modifying agents used in autoimmune disease or immune modulators used in oncologic disease) and thus such patients have to be excluded. Only patients with standard TAC target trough level are suitable for the trial and this is defined by the local centre. This might also exclude patients with, e.g. a lung transplantation, de novo DSA or thrombotic microangiopathy if the centre targets non-standard TAC trough levels in these circumstances. Patients with a TTV load always below 4.6 log10 c/mL during the screening phase cannot be randomised because TTV load might not reflect immunosuppression sufficiently in such setting.

## Trial status

Protocol version number 4.0 was submitted for approval on 07.03.2022 and approved as version 5.0 in Austria on 01.07.2022, in the Czech Republic on 01.07.2022, in France on 28.06.2022, in Germany on 01.07.2022, in the Netherlands on 30.06.2022 and in Spain on 08.07.2022. Version 6.0 was submitted as the first amendment on 15.07.2022 and approved in Austria on 12.10.2022, in the Czech Republic on 17.10.2022, in France on 14.10.2022, in Germany on 13.10.022, in the Netherlands on 17.10.2022 and in Spain on 13.10.2022. The first patient was recruited on 25.08.2022, and recruitment is expected to be completed in April 2024.

## Supplementary Information

**Additional file 1.**

**Additional file 2.**

**Additional file 3.**

**Additional file 4.**

**Additional file 5.**

**Additional file 6.**

**Additional file 7.**

**Additional file 8.**

**Additional file 9.**

**Additional file 10.**

## Data Availability

The full trial database and analysis dataset will be made available as far as possible according to the Open Research Data Pilot.

## Abbreviations

ABMR: Antibody-mediated rejection
AE: Adverse event
BKV: BK virus
BL TCMR: Borderline rejection suspicious for T-cell-mediated rejection
CMV: Cytomegalovirus
DSA: Donor-specific antibodies
DSMB: Data and Safety Monitoring Board
EBV: Epstein-Barr virus
eCRF: Electronic case report form
eGFR: Estimated glomerular filtration rate
TAC: Tacrolimus
TCMR: T-cell-mediated rejection
TTV: Torque Teno virus
