# Supplementary material for: A multicentre, patient- and assessor-blinded, non-inferiority, randomised and controlled phase II trial to compare standard and torque teno virus-guided immunosuppression in kidney transplant recipients in the first year after transplantation: TTVguideIT
Source: Trials. 2023 Mar 22;24:213. doi: 10.1186/s13063-023-07216-0 (PMC10032258; doi:10.1186/s13063-023-07216-0)
Supplement: Supplementary file 1 — Additional file 1. [file 13063_2023_7216_MOESM1_ESM.pdf]

TRIAL PROTOCOL

A NON-INFERIORITY, RANDOMISED AND CONTROLLED TRIAL TO COMPARE THE SAFETY, TOLERABILITY AND PRELIMINARY EFFICACY BETWEEN STANDARD AND TORQUE TENO VIRUS-GUIDED IMMUNOSUPPRESSION IN STABLE ADULT KIDNEY TRANSPLANT RECIPIENTS WITH LOW IMMUNOLOGICAL RISK IN THE FIRST YEAR AFTER TRANSPLANTATION

TTV GUIDE IT

Randomised and controlled, patient/assessor-blinded, two-arm, non-inferiority, interventional, multicentre, multinational investigator driven phase II trial

Version 6.0F, 15.07.2022

Author(s): Associate Professor PD Dr. Gregor Bond, PhD

Sponsor:

Medical University of Vienna, Spitalgasse 23, 1090 Vienna, Austria

Sponsor Code: *TTV GUIDE IT*

EU CT-Number: 2022-500024-30-00

EudraCT-Number: *2021-002525-24*

Principal Coordinating Investigator (PCI):

Assoc. Prof. PD Dr. Gregor Bond, PhD

General Hospital Vienna

Nephrology and Dialysis

Währinger Gürtel 18-20

1090 Vienna, Austria

CONFIDENTIALITY

The information in this protocol must be kept strictly confidential until the results of the clinical trial are published. It is intended only for the information of the investigators, other persons involved in the trial, the ethics committees and the authorities. This protocol may not be disclosed to uninvolved parties without the consent of the principal coordinating investigator mentioned above.

TABLE OF CONTENTS

Involved Persons/Institutions **6**
Synopsis **8**
Visit schedule **14**
1 Introduction **16**
1.1 Background of the clinical trial 16
1.1.1 Name and description of the investigational products 18
1.2 Rationale of the clinical trial 18
1.3 Benefit-risk assessment 19
1.3.1 Summary of the known and potential risks and benefits, if any, to human subjects. 19
2 Objectives of the clinical trial **22**
2.1 Primary objective 22
2.2 Secondary objectives 22
3 Description of the clinical trial **23**
3.1 Trial design 23
3.2 Primary end point 23
3.3 Secondary end points 23
3.4 Investigations accompayning the trial 24
3.5 Sample size 24
3.6 Subject recruitment 24
3.7 Time schedule 24
3.8 Requirements for trial sites and investigators 27
4 Population of trial subjects **28**
4.1 Description of the trial population 28
4.2 Inclusion criteria 28
4.3 Exclusion criteria 29
5 Investigational medicinal products **31**
5.1 Identification and description of investigational medicinal products (IMP(s)) 31
5.2 Side effects and interactions 31
5.3 Manufacturing and labelling 32
5.4 Storage, dispense, return and documentation of investigational drug (drug accountability) 32
5.5 Assignment of the treatment arm 32
5.6 Treatment plan 32
5.7 Instructions for dose adjustment 32
5.7.1 Active/Intervention Group 33
5.7.2 Main Principles 33
5.7.3 Additonal Rules 35
5.7.4 Hints for individualization of TAC dosage 35
5.7.5 Endpoint related treatments 35
5.7.6 Control Group 36
5.7.7 General Rule 36
5.8 Emergency measures 37
5.9 Concomitant medication 37
5.10 Adherence 37
5.11 Blinding and emergency envelopes 37

5.11.1 Blinding 37
5.11.2 Emergency envelopes 37
5.11.3 Premature unblinding in an emergency 38
5.11.4 Regular unblinding 38

6 Course of the clinical trial 39
6.1 Implementation of informed consent 39
6.2 Registration/Randomization 40
6.3 Screening V-3 (at the ward in the first week post-transplantation; latest until discharge) 41
6.4 Screening V-2 and v-1 (at routine visit in month 2 and 3 post-transplantation) 41
6.5 Visit 1 (Day 0; after day 93 post-transplantation, within month 4 post-transplantation) 41
6.6 Visit 2 (Week 6; ± 14 days) 42
6.7 Visit 3 (Week 12; ± 14 days) 42
6.8 Visit 4 (Week 18 ± 14 days) 42
6.9 VisIt 5 (Week 24; ± 14 days) 42
6.10 Visit 6 (Week 30; ± 14 days) 42
6.11 Visit 7 (Final visit: week 36 ± 14 days) 42
6.12 Follow-Up 43
6.13 Routine Calls 43
6.14 Deviations from the protocol 43
6.15 Assessment of efficacy 44
6.16 Assessment of safety 44
6.16.1 Specification of safety parameters 44
6.16.2 The methods and timing for assessing, recording, and analysing safety parameters 45
6.17 Trial-specific investigations 45
6.18 Biomaterials 49
6.19 Rescue Therapy 49
6.20 Further treatment after completion of the trial 49
6.21 Premature termination of trial therapy for a subject 50
6.22 Premature termination of the clinical trial for a subject 50
6.23 Premature termination of the clinical trial 50

7 Safety/Pharmacovigilance 52
7.1 Definitions 52
7.1.1 Pregnancy 52
7.2 Documentation of (S)AEs 53
7.2.1 Severity of adverse events 53
7.2.2 Relationship to study drug 54
7.2.3 Special features with SAEs 54
7.2.4 Deaths 55
7.3 Reporting obligations 55
7.3.1 Reporting obligations of the investigator 55
7.3.2 Reporting obligations of the sponsor 55
7.4 Data Safety Monitoring Board (DSMB) 56
7.5 Contraception 57
7.6 Dealing with pregnancies 57
7.6.1 Pregnancial complications and abortions 58
7.6.2 Storage of documentation 58

7.7 In-vitro diagnostic medical device (IVD) ... 58
8 Documentation and Data management ... 59
8.1 Delegation log of the trial site ... 59
8.2 Subject identification log ... 59
8.3 Investigator site file (ISF) ... 59
8.4 Case report form ... 59
8.5 Data management ... 60
8.6 Data storage ... 60
8.6.1 Storage obligations of the trial site ... 60
8.6.2 Storage obligations of the sponsor ... 60
9 Monitoring and Audit ... 62
9.1 Access to source data ... 62
9.2 Monitoring ... 62
9.3 Audit/Inspection ... 63
10 Statistics ... 64
10.1 Statistical hypotheses ... 64
10.2 Sample size calculation ... 64
10.3 Statistical Analysis ... 65
10.3.1 Definition of evalution populations ... 65
10.3.2 Baseline parameters and concomitant medications ... 65
10.3.3 Statistical Analysis of the primary end point ... 66
10.3.4 Statistical Analysis of the secondary end points ... 66
10.3.5 Multiple testing ... 67
10.3.6 Interim analysis ... 67
10.3.7 Missing values ... 67
10.3.8 Exploratory subgroup analysis ... 67
10.3.9 Statistical software ... 67
11 Ethical, legal and administrative aspects ... 68
11.1 Responsibilities of sponsor and investigator ... 68
11.2 Approving evaluation of the ethics commission and notification to the authorities ... 69
11.3 Subject insurance ... 69
11.4 Data protection and confidentiality ... 70
11.5 Ethics and Governance Council ... 70
12 Amendments to the trial protocol ... 72
13 Publication ... 73
13.1 Final report ... 73
13.2 Publications ... 73
13.3 Data sharing ... 73
14 Signatures ... 74
15 List of abbreviations ... 76
16 List of literature ... 78

INVOLVED PERSONS/INSTITUTIONS

**Sponsor**	**Medical University of Vienna**<br>Spitalgasse 23<br>1090 Vienna, Austria<br>(represented by the Principal coordinating Investigator)
**Principal coordinating Investigator**<br>(PCI)	**Assoc. Prof. PD. Dr. Gregor Bond, PhD**<br>Nephrology and Dialysis, General Hospital Vienna<br>Währinger Gürtel 18-20, 1090 Vienna, Austria<br>email: gregor.bond@meduniwien.ac.at<br>phone/ fax: +43 (0)1 40400-43910/ -43920
**Biometrics**	**Assoc. Prof. PD. Dr. Franz König**<br>Medical University of Vienna<br>Center for Medical Statistics, Informatics, and Intelligent Systems<br>Section for Medical Statistics<br>Spitalgasse 23, 1090 Vienna, Austria<br>email: franz.koenig@meduniwien.ac.at<br>phone: +43 (0)1 40400-74800
**Trial Coordination**	**Dr. rer. nat. Roland Pfeiffer**<br>Medizinische Fakultät der TU Dresden<br>KKS Dresden<br>Fetscherstraße 74, 01307 Dresden<br>email: ttvguidetx.kks@mailbox.tu-dresden.de<br>phone/ fax: +49(0)351458-15169/ -5799
**Pharmacovigilance**	**Barbara Djawid**<br>Medizinische Fakultät der TU Dresden<br>KKS Dresden<br>Fetscherstraße 74, 01307 Dresden<br>email: pharmakovigilanz@uniklinikum-dresden.de<br>phone/ fax: +49(0)351458-13246/-5799
**Monitoring**	**Dr. rer. nat. Roland Pfeiffer**<br>Medizinische Fakultät der TU Dresden<br>KKS Dresden<br>Fetscherstraße 74, 01307 Dresden<br>email: ttvguidetx.kks@mailbox.tu-dresden.de<br>phone/ fax: +49(0)351458-15169/ -5799
**Data Management**	**Sandra König**<br>Medizinische Fakultät der TU Dresden<br>KKS Dresden<br>Fetscherstraße 74, 01307 Dresden<br>email: sandra.koenig@uniklinikum-dresden.de<br>phone/ fax: +49 351 458 – 15132/ -5799

**Biobank**	**Mag. Dr. med. univ. & scient. med. Helmuth Haslacher, BSc BA**<br>MedUni Wien Biobank, General Hospital Vienna; Währinger Gürtel 18-20, 1090 Vienna, Austria<br>email: helmuth.haslacher@meduniwien.ac.at<br>phone/ fax: +43 (0)1 40400 53190/ 40495 15547

SYNOPSIS

**Sponsor**	**Medical University of Vienna**<br>Spitalgasse 23, 1090 Vienna, Austria
**Principal Coordinating Investigator** (PCI)	**Assoc. Prof. PD. Dr. Gregor Bond, PhD**
**Full Title**	A non-inferiority, randomised and controlled trial to compare the safety, tolerability and preliminary efficacy between standard and Torque Teno virus-guided immunosuppression in stable adult kidney transplant recipients with low immunological risk in the first year after transplantation
**Public Title**	Personalized dosing of immunosuppression after kidney transplantation by measuring the immune system functionality
**Short Title**	TTV GUIDE IT
**EU CT-Number**	2022-500024-30-00
**Target Population**<br>(or indication)	260 adult stable immunological low risk recipients of a kidney transplant with tacrolimus based immunosuppression and Torque Teno virus (TTV) infection in month 4 after transplantation.
**Phase**	Clinical trial phase II
**Trial Design**	Randomised, controlled, interventional, two-arm, non-inferiority, patient and assessor-blinded, multinational and investigator driven
**Objectives of the Clinical Trial**	Immunosuppression after kidney transplantation is mainly guided via calcineurin inhibitor (CNI) trough level, which is not able to predict allograft rejection or infection sufficiently. The peripheral blood level of the apathogenic and highly prevalent TTV is associated with the immunosuppression of its host. Non-interventional studies suggest TTV load to predict allograft rejection and infection in the first year after kidney transplantation.<br>Primary objective:<br>To demonstrate non-inferiority with respect to safety, tolerability and preliminary efficacy of TTV-guided immunosuppression compared to standard TAC dosing in stable adult kidney transplant patients with low immunological risk in the first year after transplantation<br><br>Secondary objective:<br>Assessment of TTV-guided immunosuppression in stable adult kidney transplant patients with low immunological risk in the first year after transplantation according to secondary endpoints.
**End points of the Clinical Trial**	Primary end point:<br>A composite of one of the following:<br>1. Infectious disease event (diagnosed on the basis of the Infectious Diseases Guidelines 2019 published by the American Society of Transplantation) requiring one of the following:<br>a) Inpatient treatment (including day-care)<br>b) Application of anti-bacteria, fungal, viral and protozoal drugs (including increase of prophylactic treatment dose; therapy restricted to topical treatment of minor localised cutaneous disease is excluded)<br>c) Reduction of immunosuppression<br>SARS-CoV-2 positive antigen test or PCR with or without COVID-19 is excluded<br>2. Allograft rejection detected upon indication biopsy, based on the

	Banff 2019 Kidney Meeting Report including borderline rejection suspicious for T-cellular mediated rejection (BL TCMR)<br>3. Death<br>4. Graft loss<br>Secondary end points:<br>• Single components of the primary outcome<br>• Episodes of infection and graft rejection defined by the treating medical personnel<br>• Severe infection (necessitating treatment in the inpatient or day-care ward) and severe rejection (excluding BL TCMR)<br>• All of the three mentioned secondary end-points including COVID-19<br>• Episodes of infection due to COVID-19<br>• Estimated glomerular filtration rate (eGFR; current CKD EPI and MDRD abbreviated)<br>• Rejection detected by protocol biopsy at month 12 post-transplantation: according to BANFF 2019 meeting report (including/excluding BL TCMR) and according to molecular microscope (MMDX)<br>• *de novo* donor specific antibodies (DSA)<br>• Plasma TTV load<br>• TAC trough level and dose<br>• Unchanged, increased, and decreased TAC trough target levels<br>• Health related quality of live: SF-36 and MTSOSD-59R questionnaires<br>• Drug adherence assessed according to paper-based assessment, MEMS® Buttons (AARDEX Group, Switzerland) on TAC blisters, BAASIS questionnaire, claimed prescriptions, psychological evaluation and TAC trough level variability<br>• Adverse Events and Serious Adverse Events (AEs/SAEs)<br>• Development of malignoma
**Number of Subjects**	130 per group (260 in total)
**Randomization, Concealment**	1:1 randomization; allocation concealment
**Trial Schedule**	The overall trial:<br>• Recruitment time: 12 months (expendable to a maximum of 18 to 24 months depending on roll out timing)<br>• Planned beginning (FPFV): May 2022 (maximum 6 months roll out)<br>• Planned end (LPLV): April 2025<br>Regarding the trial subject:<br>• Screening: 3 months<br>• Duration of Intervention: 9 months (last 6 weeks follow-up; FUP)<br>End of Clinical Trial:<br>• LPLV: April 2025
**Inclusion Criteria**	1. Recipient of a kidney allograft<br>2. Adult (≥18 years of age)<br>3. Post day 93 following transplantation<br>4. TAC-based immunosuppression<br>5. Standard target TAC trough level (as defined by local centre; might exclude patients with e.g. a lung transplantation or *de novo* DSA or thrombotic microangiopathy [TMA] if the centre applies non-standard TAC trough levels in these circumstances)

	6. Written informed consent
**Exclusion Criteria**	1. HLA incompatible transplantation (as defined by local centre; e.g. preformed DSA and/or crossmatch conversion)<br>2. ABO incompatible transplantation (as defined by local centre; e.g. relevant ABO incompatible blood group combination)<br>3. Combined transplantation<br>4. History of HIV or active Hep B/C infection<br>5. Donor history of HIV or Hep B/C infection<br>6. TTV load always below 4.6 log_{10} c/mL during screening phase<br>7. No stable TAC trough levels achieved during screening phase (as defined by local centre)<br>8. Hypersensitivity to TAC or other macrolides and hypersensitivity to any excipients.<br>9. Cyclosporine, mTor inhibitor or Co-stimulation blocker based immunosuppression<br>10. No standard immunosuppression according to local centre definition; e.g. necessity of significant additional long term immunosuppression or immune modulation (e.g. disease modifying agents in autoimmune disease or immune modulators for cancer)<br>11. Treatment with T-cell depleting drugs within 2 months before the randomization (e.g. anti-thymocyte globulin)<br>12. Current infection or allograft rejection as defined by the primary end-point<br>13. Biopsy proven antibody mediated rejection (ABMR) or BK virus PCR $\geq 10^4$ c/ml (or corresponding U/mL) in the blood until randomisation<br>14. Unstable graft function: eGFR <25 mL/min/1.73m^2 (this limit might be ignored if creatinine clearance is >25 mL/min/1.73m^2) or rapid and relevant eGFR decline (as defined by local centre), urinary protein/creatinine ratio >2000 mg/g, or rapid and relevant increase (as defined by the local centre).<br>15. Advanced liver failure (CHILD-Pugh score C)<br>16. History of malignancy other than squamous cell carcinoma or basal cell carcinoma of the skin or carcinoma in situ or adenoma of the colon within the last 5 years unless in complete remission since at least 3 years<br>17. Leukopenia <2000/mm^3 or neutropenia <1000/mm^3<br>18. Unstable angina, cardiac decompensation with the necessity of inpatient treatment<br>19. Severe tremor (as defined by local centre) due to TAC<br>20. Inability to perform study visits at the trial centre<br>21. Any state that excludes adherence with the trial protocol, such as serious medical or psychiatric illness, language barrier, alcohol or illicit substance abuse or non-adherence<br>22. Addictions or other illnesses that do not allow the person concerned to assess the nature and extent of the clinical trial and its possible consequences<br>23. Simultaneous participation in another interventional clinical trial<br>24. Pregnant or breastfeeding women<br>25. Women of childbearing potential, except women who meet one of the following criteria:<br>a) post-menopausal (12 months natural amenorrhoea)<br>b) postoperative (6 weeks after bilateral ovarectomy with or without hysterectomy, bilateral salpingectomy)

	c) regular and correct use of a contraceptive method with an Pearl Index < 1% per year<br>d) sexual abstinence<br>e) vasectomy of the partner
**Course of the Clinical Trial**	<u>Screening (V-3; week 1 post-transplantation at the ward; latest until discharge)</u><br>• Informed consent<br>• Preliminary inclusion / exclusion criteria check<br>• Medical history<br>• Laboratory workup (TTV R-GENE®)<br>• Biobanking<br><u>Screening (V-2, V-1; at routine visits in month 2 and 3 post-transplantation)</u><br>• Laboratory workup (TTV R-GENE®; TAC-trough level; chemistry; complete blood count [CBC]; urine analysis)<br>• Biobanking<br><u>Visit 1 (day 0; after day 93 post-transplantation within month 4 post-transplantation)</u><br>• Inclusion/ exclusion criteria<br>• Medical history<br>• Medication<br>• Vital signs<br>• Physical examination, body weight<br>• Pregnancy test<br>• Laboratory workup (TTV R-GENE®; TAC-trough level; chemistry; CBC; venous blood gas analysis [vBGA]; urine analysis)<br>• Virology (BKV; cytomegalovirus [CMV])<br>• Biobanking<br>• DSA<br>• Quality of life (SF-36; MTSOSD-59R)<br>• Drug adherence (MEMS® BUTTON; patient diary; BAASIS; psychological evaluation)<br>• Data sampling concerning protocol biopsy performed according to local centre standard; protocol biopsy has to be performed before V1<br>• Intervention: adaption of TAC dose<br><u>Visit 2 (week 6; ± 14 days)</u><br>• Vital signs<br>• Medication<br>• AE<br>• Primary endpoint<br>• Laboratory workup (TTV R-GENE®; TAC-trough level; chemistry, CBC, vBGA; urine analysis)<br>• Virology (BKV; CMV)<br>• Biobanking<br>• Drug adherence (MEMS® BUTTON; BAASIS; patient diary)<br>• Intervention: adaption of TAC dose<br><u>Visit 3 (week 12; ± 14 days)</u><br>Like Visit 2<br><u>Visit 4 (week 18 ± 14 days)</u><br>Like Visit 2

	Visit 5 (week 24; ± 14 days)<br>Like Visit 2<br>Visit 6 (week 30; ± 14 days)<br>Like Visit 2<br>Visit 7 (final visit: week 36 ± 14 days)<br>• Vital signs<br>• Physical examination, body weight<br>• Medication<br>• AE<br>• Primary endpoint<br>• Pregnancy test<br>• Laboratory workup (TTV R-GENE®; TAC-trough level; chemistry, CBC; vBGA; urine analysis)<br>• Virology (BKV; CMV)<br>• Biobanking<br>• DSA<br>• Drug adherence (MEMS® BUTTON; BAASIS; patient diary; claimed prescription check; psychological evaluation)<br>• Quality of life (SF-36; MTSOSD-59R)<br>• Data sampling concerning protocol biopsy performed according to local centre standard<br>Routine calls every second week will help to detect AE and infections treated outside the study centre.
**Trial Specific Procedures**	• TTV quantification (no additional vein-puncture)<br>• Health-related quality of life questionnaires: SF-36, MTSOSD-59R<br>• Pregnancy test, physical examination, body weight and vital signs<br>• Patient diary<br>• DSA assessment (if not part of the routine post-transplant care; no additional vein-puncture)<br>• Assessment of month 12 protocol biopsy with 'molecular microscope' if performed as part of routine post-transplant care<br>• Additional adherence monitoring: MEMS® Buttons, BAASIS questionnaires, psychological evaluation and claimed prescription check<br>• Sub-study: biobanking of urine, whole blood (PAXgene), serum and plasma (no additional vein-puncture)
**Investigational Medicinal Product**	Active substance: TAC<br>Trade name: All products containing TAC and authorized in participating countries<br>Manufacturer: All manufactures of authorized products
**Treatment Schedule**	Active group<br>Product TAC<br>Dose: Dependent on TTV level (target 4.6 to 6.2 $\log_{10}$ c/mL) quantified by real-time PCR kit TTV R-GENE® in the plasma<br>Duration: 9 months<br>Control group<br>Product: TAC<br>Dose: According to local centre standard<br>Duration: 9 months

**Including Centres**	Berlin, Dresden, Graz, Grenoble, Groningen, Innsbruck, Leiden, Linz, Prague, Regensburg, Strasbourg, Valencia and Vienna
**Grant Support**	H2020 RIA, 'TTV GUIDE TX', #896932, project coordinator Assoc. Prof. PD. Dr. Gregor Bond, PhD

VISIT SCHEDULE

Table 1 Trial-specific and routine procedures performed during the interventional trial.

Post-TX month		1	2	3	4	5		8		11	12 to 13
**Study week**		-12 to -11	-8	-4	0	6	12	18	24	30	36
**Study visit**		-3	-2	-1	1	2	3	4	5	6	7 (FUP)
**Informed consent**		X									
**Inclusion/ exclusion criteria**		X			X						
**Intervention**					X	X	X	X	X	X	
**Medical History**		X			X						
**Vital signs**					X	X	X	X	X	X	X
**Physical examination, body weight**					X						X
**Pregnancy test**					X						X
**Medication**					X	X	X	X	X	X	X
**Adverse events[a]**						X	X	X	X	X	X
**Primary end-point[a]**						X	X	X	X	X	X
**Laboratory workup**	**TTV R-GENE®**	X	X	X	X	X	X	X	X	X	X
	**TAC-trough level**		X	X	X	X	X	X	X	X	X
	**Chemistry, CBC, vBGA, Urine analysis[b]**		X	X	X	X	X	X	X	X	X
	**BKV, CMV[b]**				X	X	X	X	X	X	X
	**DSA**				X						X
**Biobanking[c]**	**Whole blood, Serum, plasma, urine**	X	X	X	X	X	X	X	X	X	X
**Drug adherence[d]**	**MEMS® BUTTON**				X	X	X	X	X	X	X
	**BAASIS**				X	X	X	X	X	X	X
	**Patient diary**				X	X	X	X	X	X	X
	**Claimed prescription check**										X
	**Psychological evaluation**				X						X
**Quality of life**	**SF-36, MTSOSD-59R**				X						X
**Protocol biopsy[e]**					X						X

Note: BKV, BK virus; CBC, Complete blood count, CMV, Cytomegalovirus; DSA, Donor-specific antibodies; HLA, Human leucocyte antigen; FUP, follow-up; MTSOSD-59R, Modified Transplant Symptom Occurrence and Symptom Distress Scale 59R; rt-PCR, Real-time polymerase chain reaction; SF-36, Medical Outcomes Study Short Form 36; TAC, Tacrolimus; TTV, Torque Teno virus; TX Transplantation; vBGA, Venous blood gas analysis.

[a] Check-ups concerning infections and other adverse events will be performed additionally via telephone calls by study personnel every 2 weeks.

[b] Laboratory workup and virology screening will be performed not as part of the study protocol, but according to local centre standards. However, according to study protocol at least the leukocyte count, creatinine, and urinary protein- and albumin to creatinine ratio will have to be assessed. Other major laboratory parameters and findings concerning CMV and BKV screening including plasma CMV and BKV PCR performed routinely at the centres will be noted.

[c] Biological material will be stored for additional immunologic monitoring according to a sub-study.

[d] Adherence will be monitored during the trial by patient diary and evaluated at every visit; other assessment of adherence will be evaluated retrospectively.

[e] Protocol biopsies (including molecular microscope; MMDx) will not be performed as part of the study protocol, but according to local centre standards. However results on protocol biopsies obtained as part of the routine local centre standard will be noted. If part of the centre standard the month 3 protocol biopsy has to be performed before V1.

1 INTRODUCTION

1.1 BACKGROUND OF THE CLINICAL TRIAL

Scientific problem

Kidney transplantation is the gold standard of treatment for patients with ESRD (End stage renal disease). After transplantation, immunosuppressive drugs are crucial for reducing the risk of organ rejection. Despite this desired effect, the compromised immunity of the recipient leads to an increased risk for infectious disease. Moreover, current immunosuppression regimens are unable to sufficiently control allo-recognition of the graft, which leads to chronic rejection. Thus, optimal management of immunosuppressive drug dosing requires a delicate balance between inadequate and excessive immunosuppression. At present, **there is no diagnostic test or algorithm for optimal guidance of immunosuppressive drugs in clinical routine**. Monitoring relies on the quantification of calcineurin inhibitors trough levels, mostly tacrolimus (TAC), in the peripheral blood, which correlate more closely with the risk of drug-related toxicity than with the effectiveness of immunosuppression. There is an urgent need for tools to personalize immunosuppression in order to reduce the risk of infectious disease and, at the same time, graft rejection.

Status of the research

While most of the proposed assays focus on graft rejection, a useful approach for guidance of immunosuppression would ideally predict both graft rejection and infectious disease. To address this, two assays were proposed. A test of leucocyte function, the QuantiFERON Monitor (Qiagen), was prognostic for infectious events, but not for graft rejection in kidney, liver and lung transplant patients (Mian et al. Clin Infect Dis. 2018). In a randomized controlled trial, tailoring immunosuppression after liver transplantation via assessment of $CD4^+$ lymphocyte function using ImmuKnow® (Erofins Viracor) resulted in fewer infectious events, but had no influence on graft rejection (Ravaioli et al. Transplantation. 2015). However, the trial design precluded reliable analysis concerning safety and efficacy. Currently, no further interventional trials in solid organ transplantation are registered for either of these products or for other tools capable of quantifying immune function in a clinically useful manner.

Recently, no major safety signal was observed in a German multicenter, open-label randomized, controlled trial in pediatric kidney recipients testing the steering of immunosuppressive therapy by levels of virus-specific T cells (adenovirus, cytomegalovirus, and herpes simplex virus quantified by cytokine flow cytometry; Ahlenstiel-Grunow et al. JASN. 2021). No difference was observed in rejection and infection, which were part of the secondary analysis. Of note, the complexity of virus-specific T cell monitoring poses a major obstacle for further efficacy trials and introduction in the clinical routine.

Torque Teno virus

Monitoring TTV in peripheral blood is a promising new strategy for quantifying immune function (De Vlaminck et al. Cell. 2013; Focosi et al. Clinical microbiology and infection. 2016). TTV can be detected in up to 90% of healthy individuals and has not been linked to any human disease. Peripheral blood copy numbers of **TTV are associated with the function of the immune system of the host.** The prevalence of TTV in patients after transplantation is around 99% and the virus is unaffected by conventional anti-viral drug therapy used in the post-transplantation setting. TTV copy number is directly associated with factors determining the immune function including age, sex and the amount and type of immunosuppressive drugs

administered to transplant recipients and is thus indirectly associated with graft rejection and infectious disease.

Quantification of TTV represents a novel approach for the assessment of the human immune system. Instead of characterizing single compartments of the immune system, TTV copy number might be able to mirror the function of the host's immune system in a more holistic aspect. Besides T cells, other major components of the immune system are involved in TTV control: NK cells, antigen-presenting cells, B cells, the complement system, pattern recognition receptors and antibodies.

We and others have demonstrated the predictive value of TTV copy number for infectious disease and organ rejection in kidney allograft recipients.

Preliminary data

In a prospective study, we analysed 169 consecutive kidney transplant recipients and found that the risk for infectious disease could already be stratified by TTV 3 months before the event (median $4x10^8$ copies/mL [c/mL], interquartile range [IQR] $3x10^7$-$2x10^9$ for infections vs. $3x10^7$, IQR $1x10^6$-$7x10^8$ for non-infections), whereas routine clinical markers, including level of immunosuppressive drugs, were not predictive (Strassl et al. The Journal of Infectious Diseases. 2018). Colleagues from Spain have confirmed our findings (Fernandez-Ruiz et al. American Journal of Transplantation. 2019). In a cohort study, we analysed 113 kidney transplant patients and described the predictive value of TTV for clinically overt acute rejection (Strassl R et al. The Journal of Infectious Diseases. 2019). Conventional markers were non-predictive, but TTV copies were predictive for rejection more than one month before diagnosis was made by indication biopsy (rejection: median $3x10^7$ c/mL, IQR $5x10^5$-$2x10^8$ vs. no rejection: $2x10^8$, IQR $1x10^7$-$4x10^9$).

In direct preparation for this trial, we re-analysed TTV in all consecutive kidney graft recipients transplanted at the MUV between 2016 and 2018 and confirmed the predictive value of TTV for infectious disease and clinically overt rejection in 386 patients (Doberer K et al. American Journal of Transplantation. 2020). Each log increase in TTV load decreased the odds for rejection by 22% (odds ratio [OR] 0.78, 95% confidence interval [CI] 0.62-0.97; p=0.027) and increased the odds for infection by 11% (OR 1.11, 95% CI 1.06-1.15; p<0.001). TTV was quantified at a median of 14 days before rejection was diagnosed and 27 days before onset of infection. Patients with allograft rejection had lower levels of TTV compared to patients without rejection (median $3.5x10^6$ c/mL, IQR $1.7x10^5$-$1.3x10^8$ c/mL vs. median $2.5x10^8$ c/mL, IQR $5.8x10^6$-$9.3x10^8$ c/mL, respectively; p=0.028) in subsequent biopsies. Applying receiver operating characteristic curve, an area under the curve of 0.73 (IQR, 0.54-0.92; p=0.028) was calculated to classify rejection by TTV level. A TTV level cut-off of $1.5x10^6$ c/mL corresponded to a specificity of 89% and a sensitivity of 36%, a negative predictive value (NPV) of 77% and a positive predictive value (PPV) of 50%. For TTV levels above 10^7 c/mL, high NPVs (range 84% to 87%) were calculated, and PPVs were high below a TTV level of 10^5 c/mL (range 57% to 85%). Higher levels of TTV were detected in the plasma drawn from individuals experiencing an infectious event in the subsequent observation period compared to patients without infection (median $3.9x10^8$ c/mL, IQR $7.9x10^6$-$3.3x10^9$ c/mL vs. median $2.6x10^7$ c/mL, IQR $1.3x10^6$-$9.2x10^8$ c/mL, respectively; p<0.001). Further subgroup analysis showed a comparable effect size for the association between TTV level and infections that did not require hospitalisation (OR 1.10, 95% CI 1.04-1.16, p<.001). The largest effect size was calculated for BK infections (presumptive polyomavirus-associated nephropathy [PVAN] and PVN; OR 1.21, 95% CI 1.06-1.39, p=0.005), followed by cytomegalovirus (CMV) disease (CMV syndrome and end-organ disease; OR 1.16, 95% CI

1.05-1.27, p=0.005) and infections restricted to opportunistic pathogens (OR 1.16, 95% CI 1.07-1.26, p<0.001). A smaller effect size was found for infections with extracellular bacteria (OR 1.06, 95% CI 1.00-1.12, p=0.05). Applying receiver operating characteristic curve, an area under the curve of 0.62 (IQR, 0.58-0.67; p<0.001) was calculated to classify infection by TTV level. A TTV level >5.8x10^9 c/mL corresponded to a specificity of 90%, a sensitivity of 18%, an NPV of 77% and a PPV of 37% to detect infection. For TTV level up to 10^9 c/mL, high NPVs were calculated for the detection of infection (range 79% to 100%), and the highest PPV was calculated for TTV loads above 10^{10} c/mL (range 44% to 67%).

In an addition study we analysed 82 protocol biopsies from month 12 after transplantation from clinically stable kidney transplant recipients transplanted consecutively in 2017 and 2018 at the MUV and confirmed the predictive value of TTV in this setting (Doberer et al. Transplantation. 2021). Patients with graft rejection had a lower TTV load than patients without rejection (median 2x10^5 c/mL, IQR 3x10^3-2x10^6 vs. 7x10^5 c/mL, IQR 1x10^5-2x10^7). Each log increase in TTV load decreased the risk for rejection by 9% (RR 0.91, 95% CI 0.85-0.97; *P*=.004). Interestingly, the time TTV measurements were below 1x10^6 c/mL between months 3 and 12 post-transplant was associated with the development of chronic lesions in the month 12 biopsies as compared to month 3 protocol biopsies: the longer the TTV c/ml was below 1x10^6, the more likely lesions would be observed in the month 12 biopsy (coefficient 0.07, 95% CI 0.01-0.14; *P*=.02).

1.1.1 NAME AND DESCRIPTION OF THE INVESTIGATIONAL PRODUCTS

General considerations

Within the active/interventional group TAC will be dosed supported by TTV load in the plasma quantified by the real-time PCR detection and quantification kit, TTV R-GENE®. TAC is approved for its intended use in this clinical study by all national competent regulatory authorities of the participating countries, and the TTV R-GENE® is CE certified. The intervention itself is restricted to a novel dosing strategy for TAC according to TTV load quantified by TTV R-GENE®. For details see 5.1.

Tacrolimus

TAC is a macrolide calcineurin inhibitor. TAC is indicated for the prophylaxis of organ rejection, in adult and pediatric patients receiving allogeneic kidney, liver transplants and heart transplants, in combination with other immunosuppressants. TAC is approved for its intended use in this clinical study by all national competent regulatory authorities of the participating countries.

The TTV R-GENE®, using the real-time PCR technology after extraction of the viral DNA, enables to detect and quantify the genome of TTV in whole blood, plasma and serum samples. The kit is intended for in vitro diagnostic use only, in clinical laboratories by laboratory health professionals. The TTV R-GENE® is CE certified.

1.2 RATIONALE OF THE CLINICAL TRIAL

The specific aim of the proposal is to demonstrate the safety and tolerability of TTV-guided immunosuppression in kidney transplant recipients. For the first time, a comprehensive, personalised and functional assessment of the immune system will be tested in a randomised and controlled clinical setting.

Kidney transplantation is the gold standard of treatment for patients with ESRD. After transplantation, immunosuppressive drugs are crucial for reducing the risk of organ rejection.

Despite this desired effect, the compromised immunity of the recipient leads to an increased risk for infectious disease. Moreover, current immunosuppression regimens are unable to sufficiently control allo-recognition of the graft, which leads to chronic rejection. Thus, optimal management of immunosuppressive drug dosing requires a delicate balance between inadequate and excessive immunosuppression. **At present, there is no diagnostic test or algorithm for optimal guidance of immunosuppressive drugs in clinical routine.**

The proposed clinical trial will assess the potential of a novel healthcare intervention that would generate meaningful advances in clinical practice and care for patients with ESRD. Notably, the trial would enable clinicians to personalise and optimise immunosuppressive drug dosing and thus reduce infections and graft rejection in kidney transplant recipients. Both outcomes are burdensome for kidney transplant patients and highly clinically relevant. Infections are the second-most common cause of death following kidney transplantation and rejection is the leading cause of graft loss.

1.3 BENEFIT-RISK ASSESSMENT

1.3.1 SUMMARY OF THE KNOWN AND POTENTIAL RISKS AND BENEFITS, IF ANY, TO HUMAN SUBJECTS.

General considerations

Non-interventional studies have suggested that measurement of TTV load in the peripheral blood of kidney transplant recipients is superior compared to standard TAC trough level-based risk prediction of harmful effects due to insufficient or excessive immunosuppression in the first year post-transplantation. However, no interventional study has tested the safety or efficacy of TTV-guided immunosuppression. **Taken together, non-interventional studies provide sufficient scientific evidence to use TTV-guided immunosuppression, but genuine uncertainty about the safety, tolerability and effectiveness warrant an interventional trial.**

Risk of trial therapy

Within the trial TAC will be dosed supported by TTV quantification. Adverse reactions of TAC are well known and described. We do not expect other adverse reactions. In worse case a higher rate or intensity of these adverse reactions might occur.

The most common adverse reactions observed in TAC-treated kidney transplant patients are: infection, tremor, hypertension, abnormal renal function, constipation, diarrhoea, headache, abdominal pain, nausea, asthenia, pain, insomnia, peripheral oedema, hypomagnesemia, hypophosphatemia, hyperlipidaemia, hyperkalaemia, and anaemia.

- Patients receiving TAC, are at increased risk of developing lymphomas and other malignancies, particularly of the skin. Post-transplant lymphoproliferative disorder has been reported in immunosuppressed organ transplant recipients. Cases of pure red cell aplasia have been reported in patients treated with TAC.
- Patients receiving TAC are at increased risk of developing bacterial, viral, fungal and protozoal infections, including opportunistic infections. These infections may lead to serious, including fatal, outcomes. Serious viral infections reported include PVAN, mostly due to BK virus infection and CMV infections.
- TAC was shown to cause new onset diabetes mellitus.
- TAC can cause acute or chronic nephrotoxicity.

- TAC may cause a spectrum of neurotoxicities. The most severe neurotoxicities include posterior reversible encephalopathy syndrome, delirium, seizure and coma; others include tremors, paresthesias, headache, mental status changes, and changes in motor and sensory functions.
- Hyperkalaemia has been reported with TAC. Hypertension is a common adverse effect of TAC therapy. TAC may prolong the QT/QTc interval and may cause Torsade de Pointes. Myocardial hypertrophy has been reported.

TAC will be administered oral. If oral administration is not possible intravenous application will be used. The TAC dosage regimen is detailed in 5.7

Risk of trial interventions
No additional invasive procedures such as venous puncture or allograft biopsy, will be performed for study purposes only. Protocol biopsies will be performed only where established as part of the clinical routine. Sampling for TTV quantification will be performed at routine blood draws without requiring an additional venous puncture. The amount of additional blood drawn at a single visit is not expected to cause harm to the participant.

Specific measures to reduce participant risk during the trial have been implemented:

- Immunological high-risk patients will be excluded.
- TAC related AE will be assessed rigorously throughout the trial.
- An independent DSMB (Data Safety Monitoring Board) will regularly check whether predefined safety margins, sample size assumptions and rates of recruitment and loss to follow-up are met, as well as whether the level of data quality and protocol compliance is sufficient. For details see 7.4.

Benefit of trial therapy
We and others have demonstrated the predictive value of TTV copy number for infectious disease and organ rejection in kidney allograft recipients (Strassl et al. The Journal of Infectious Diseases. 2018 and 2019; Fernandez-Ruiz et al. American Journal of Transplantation. 2019; Doberer K et al. American Journal of Transplantation. 2020 and Transplantation 2021). **Within the proposed trial we anticipate a reduction from infection and rejection from 40% to 30%.**

The results of the proposed trial have the potential to change clinical practice of immunosuppression dosage after kidney transplantation, thereby reducing infectious and immunologic complications. Effective project management, rigorous quality assurance and control and detailed risk assessment will increase the likelihood of a successful conduction of the trial and scientific integrity of the data.

Lower infection and rejection rates achieved through optimisation of immunosuppression would result in reduced hospitalisation and harm due to the side effects of antimicrobial and anti-rejection therapy. The prolongation of graft survival reduces the need for dialysis, which is a very burdensome procedure for ESRD patients, and thus improves quality of life. Improved graft survival would also reduce the number of patients re-entering the waiting-list for a kidney transplant after terminal graft failure, thereby shortening the waiting-time for ESRD patients on the waiting list for a suitable kidney. These changes would result in significant improvement in health-related quality of life for ESRD patients.

The results of the trial have the potential to contribute to increase patient and graft survival and thus to reduce the socioeconomic burden of ESRD for healthcare systems.

Taken together, non-interventional data support the hypothesis of the trial and equipoise for the suggested intervention is given, the potential benefits outweigh the potential risks for the participants and the trial has the potential to improve clinical practice.

2 OBJECTIVES OF THE CLINICAL TRIAL

2.1 PRIMARY OBJECTIVE

The primary objective of the clinical trial is to demonstrate non-inferiority with respect to safety, tolerability and preliminary efficacy of TTV-guided immunosuppression compared to standard TAC dosing in stable adult kidney transplant patients with low immunological risk in the first year after transplantation.

For the first time, a holistic personalised and functional assessment of the immune system will be tested in a randomised and controlled clinical setting.

2.2 SECONDARY OBJECTIVES

The secondary objective of the clinical trial is the assessment of TTV-guided immunosuppression in stable adult kidney transplant patients with low immunological risk in the first year after transplantation on kidney function, transplant rejection detected by protocol biopsy at month 12 post-transplantation, development of *de novo* DSA, number of unscheduled emergency hospital visits, admissions to the inpatient ward, development of malignoma, health related quality of life and drug adherence.

3 DESCRIPTION OF THE CLINICAL TRIAL

3.1 TRIAL DESIGN

Randomized, controlled, interventional, two arm, non-inferiority, patient and assessor blinded, multinational, and investigator driven phase II trial to compare the safety, tolerability and preliminary efficacy between standard and Torque Teno virus-guided immunosuppression in stable adult kidney transplant recipients with low immunological risk in the first year after transplantation.

3.2 PRIMARY END POINT

A composite of one of the following:

1. **Infectious disease event** (diagnosed on the basis of the Infectious Diseases Guidelines 2019 published by the American Society of Transplantation) requiring one of the following:
   a) Inpatient treatment (including day-care)
   b) Application of anti-bacteria, fungal, viral and protozoal drugs (including increase of prophylactic treatment dose; therapy restricted to topical treatment of minor localised cutaneous disease is excluded)
   c) Reduction of immunosuppression

SARS-CoV-2 positive antigen test or PCR with or without COVID-19 is excluded

2. **Allograft rejection** detected upon indication biopsy, based on the Banff 2019 Kidney Meeting Report including borderline rejection suspicious for T-cellular mediated rejection (BL TCMR)
3. **Death**
4. **Graft loss**

For the primary end point analysis all episodes of infection and allograft biopsy will be reassessed by personnel blinded to the randomization code (infection: two infectious disease specialists; biopsy: two kidney pathologist; the two assessors have to agree on one diagnosis). For this purpose biopsy slides will be made electronically available for the assessor by scanning. Infections will be evaluated according to the data provided in the eCRF, which will be recorded according to the protocol. The monitor will screen for non-reported episodes of infections. Routine calls and patient diaries will help to detect episodes of infections.

3.3 SECONDARY END POINTS

- Single components of the primary outcome
- Episodes of infection and graft rejection defined by the treating medical personnel
- Severe infection (necessitating treatment in the inpatient or day-care ward) and severe rejection (excluding BL TCMR)
- All of the three mentioned secondary end-points including COVID-19
- Episodes of infection due to COVID-19
- Estimated glomerular filtration rate (eGFR; current CKD EPI and MDRD abbreviated)
- Rejection detected by protocol biopsy at month 12 post-transplantation: according to BANFF 2019 meeting report (including/excluding BL TCMR) and according to molecular microscope (MMDX)
- *de novo* donor specific antibodies (DSA)

- Plasma TTV load
- TAC trough level and dose
- Unchanged, increased, and decreased TAC trough target levels
- Health related quality of live: SF-36 and MTSOSD-59R questionnaires
- Drug adherence assessed according to paper-based assessment, MEMS® Buttons (AARDEX Group, Switzerland) on TAC blisters, BAASIS questionnaire, claimed prescriptions, psychological evaluation and TAC trough level variability
- Adverse Events and Serious Adverse Events (AEs/SAEs)
- Development of malignoma

3.4 INVESTIGATIONS ACCOMPAYNING THE TRIAL

Sub-study: Further immunologic monitoring; Biological material (serum, plasma, whole blood and urine) will be sampled for further immunologic monitoring including but not restricted to further DSA assessment, donor derived cell free DNA [ddcfDNA], gene expression and urinary TTV and chemokines.

3.5 SAMPLE SIZE

A total of 260 patients will be included in the trial, 130 of them in the active group and 130 in the control group.

For the sample size calculation, we analysed the occurrence of the primary endpoint (infection, allograft rejection, death and graft loss) in recipients of a kidney allograft transplanted between 1.1.2012 and 31.12.2018 at the Vienna centre, applying the trial-specific inclusion and exclusion criteria (unpublished data). Overall, 40% of patients experienced the primary endpoint between month 3 and 12 after transplantation.

An inclusion of 260 recipients will give a power of >90% to demonstrate non-inferiority with respect to the primary endpoint (assuming a decrease from 40% to 30%) using a one-side significance level of alpha 2.5%, given a 1:1 randomisation ratio. If the differences are larger, there is also sufficient power to demonstrate superiority. More details are given in section 10.

3.6 SUBJECT RECRUITMENT

Recruitment and trial entry is detailed in Figure 1. All consecutive adult recipients of a kidney allograft will be subjected to screening within the first two week after transplantation at the inpatient ward by the study team (study visit -3) and entered in the screening log. If baseline inclusion criteria are met (e.g. tacrolimus-based immunosuppression) and no baseline exclusion criteria are present (e.g. immunologic high risk, active participation in another clinical trial, any state that excludes adherence with the trial protocol, inability to perform study visits at the trial centre) informed consent will be obtained and TTV will be quantified. TTV will then be assessed in month 2 and 3 after transplantation at routine study visits.

3.7 TIME SCHEDULE

Trial Flow and Design

The trial flow and design is detailed in Figure 1 and Figure 2. Patients will meet the study team again at the outpatient clinic in month 4 after transplantation at a routine visit. If all inclusion criteria are met and no exclusion criteria are present participants will be enrolled into the clinical trial and randomized (study visit 1). TAC dose will be adjusted according to TTV-guided TAC trough level target or routine centre TAC trough level target. Participants will visit the outpatient

clinic every 6 weeks following the same procedure up to and including month 12 after transplantation (study visit 6). Follow-up will be performed until month 13 post-transplantation (study visit 7).

End of trial
The end of trial is last visit of last patient (LPLV).

Figure 1 Trial Flow.

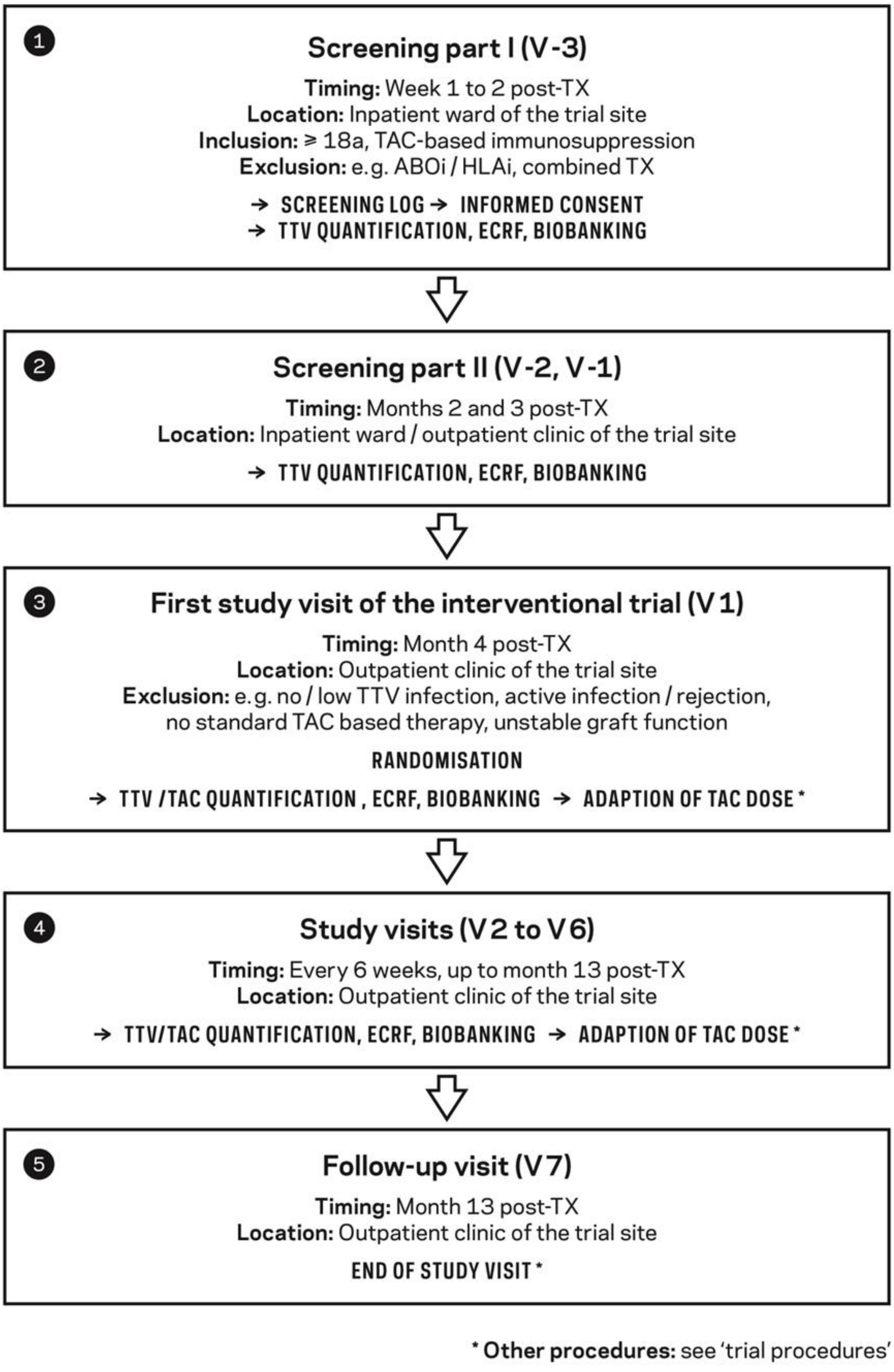

Figure 2 Trial Design.

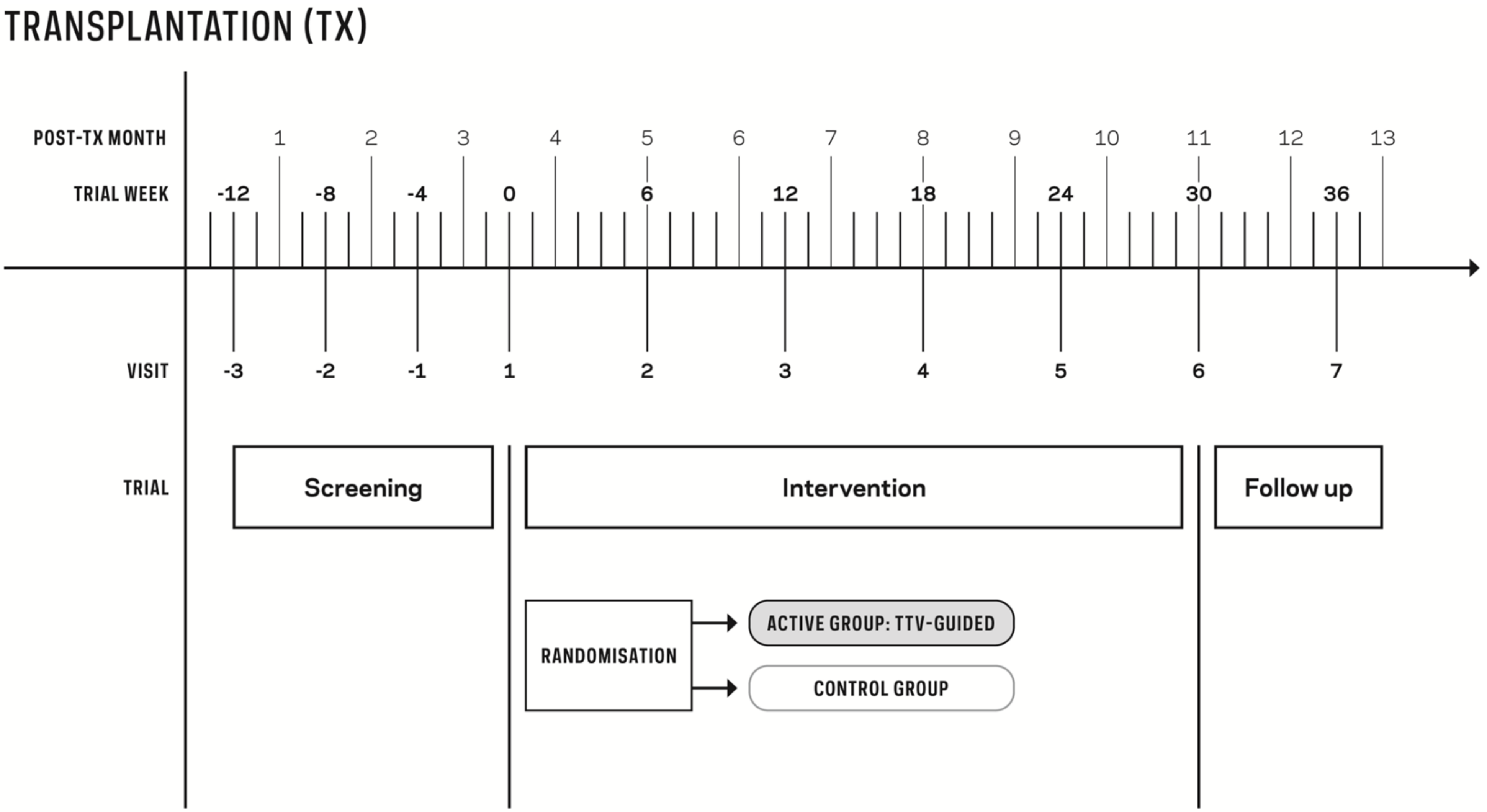

3.8 REQUIREMENTS FOR TRIAL SITES AND INVESTIGATORS

All clinical/recruitment centres are university-based tertiary care centres with a high-volume kidney transplant unit. The local PIs at the participating trial sites are internationally recognised scientists in the field of kidney transplantation with a track record of successful conduction of interventional clinical trials. At each centre, appropriately trained medical staff will be available to provide the necessary standard of care for trial participants and to perform medical care specific to the trial and beyond. The trial sites have a local laboratory or cooperate contractually with external service providers in order to be able to perform the laboratory diagnostics required for the trial in a qualified manner. Current quality certificates or adequate certification of other established quality control measures are provided to the sponsor to demonstrate that the investigations are properly performed during the trial period. The trial sites have Internet access in order to use web-based randomization and data entry via eCRF.

Due to their combination of fundamental virological research, applied medical-virological research, viral diagnostics and university and postgraduate teaching, the participating laboratories are centres of excellence for all current aspects of medically oriented virology. The participating trial sites have adequate human resources throughout the trial. The personnel involved in the clinical trial are qualified in accordance with the quality standards of "good clinical practice".

The investigator is responsible for selecting and assembling the members of the trial team (especially the medical staff) in accordance with the requirements of this protocol. In addition, the investigator is responsible for the training and supervision of the trial team and for providing all necessary information.

4 POPULATION OF TRIAL SUBJECTS

4.1 DESCRIPTION OF THE TRIAL POPULATION

Gender and age distribution

The trial is expected to be safe for both men and women, and differences in treatment effects between the sexes are unlikely. However, gender and sex dimensions are important factors in the proposed project and have been taken into account at various stages of the trial.

- At the stage of trial design, we considered the association between sex and TTV level, organ rejection and medical adherence: female sex is associated with lower TTV levels and lower rates of rejection and non-adherence. Analysis of the DSMB will stratify according to sex for early detection of sex related risks. Baseline variables and follow-up data known to be influenced by sex and gender will be collected. The study is designed as a mixed-sex study with equal sex distribution among the participants in order to reflect the composition of the group of patients likely to be treated with the strategy under investigation. An equal sex distribution within treatment arms will be guaranteed by appropriate randomization allocation and concealment.

- At the stage of analysis, data will be analysed separately for each sex and possible effect modifications will be considered. Likewise, factors that interact with sex/gender (as explored during statistical evaluation of the results) will be included. The study is not powered to analyse the outcome stratified for sex/gender as a subgroup.

- At the stage of dissemination, results will be displayed according to sex/gender and differences between the sexes/genders will be reported. Implications for clinical practice or further research concerning sex/gender will be emphasised.

Dependent persons

Persons dependent on the sponsor, investigator or medical staff of the trial team must not be included.

Special protection

Members of particularly vulnerable groups such as:

- underage persons,
- pregnant and breastfeeding women,
- adults who are not able to recognise the nature, significance and scope of the clinical trial and to orient their will accordingly,
- persons who are unable to give informed consent,
- persons who are accommodated in an institution by court or official order

will not be included.

Immunological high-risk patients will also be excluded.

4.2 INCLUSION CRITERIA

1. Recipient of a kidney allograft
2. Adult (≥18 years of age)
3. Post day 93 following transplantation
4. TAC-based immunosuppression

5. Standard target TAC trough level (as defined by local centre; might exclude patients with e.g. a lung transplantation or *de novo* DSA or thrombotic microangiopathy [TMA] if the centre applies non-standard TAC trough levels in these circumstances)
6. Written informed consent

4.3 EXCLUSION CRITERIA

1. HLA incompatible transplantation (as defined by local centre; e.g. preformed DSA and/or crossmatch conversion)
2. ABO incompatible transplantation (as defined by local centre; e.g. relevant ABO incompatible blood group combination)
3. Combined transplantation
4. History of HIV or active Hep B/C infection
5. Donor with history of HIV or active Hep B/C infection
6. TTV load always below 4.6$\log_{10}$ c/mL during screening phase
7. No stable TAC trough levels achieved during screening phase (as defined by local centre)
8. Hypersensitivity to TAC or other macrolides and hypersensitivity to any excipients
9. Cyclosporine, mTor inhibitor or Co-stimulation blocker based immunosuppression
10. No standard immunosuppression according to local centre definition; e.g. necessity of significant additional long term immunosuppression or immune modulation (e.g. disease modifying agents in autoimmune disease or immune modulators for cancer)
11. Treatment with T-cell depleting drugs within 2 months before the randomization (e.g. anti-thymocyte globulin)
12. Current infection or allograft rejection as defined by the primary end-point
13. Biopsy proven antibody mediated rejection (ABMR) or BK virus PCR $\geq 10^4$ c/ml (or corresponding U/mL) in the blood until randomisation
14. Unstable graft function: eGFR <25 mL/min/1.73m^2 (this limit might be ignored if creatinine clearance is >25 mL/min/1.73m^2) or rapid and relevant eGFR decline (as defined by local centre), urinary protein/ creatinine ratio >2000 mg/g, or rapid and relevant increase (as defined by local centre).
15. Advanced liver failure (CHILD-Pugh score C)
16. History of malignancy other than squamous cell carcinoma or basal cell carcinoma of the skin or carcinoma in situ or adenoma of the colon within the last 5 years unless in complete remission since at least 3 years
17. Leukopenia <2000/mm^3 or neutropenia <1000/mm^3
18. Unstable angina, cardiac decompensation with the necessity of inpatient treatment
19. Severe tremor (as defined by local centre) due to TAC
20. Inability to perform study visits at the trial centre
21. Any state that excludes adherence with the trial protocol, such as serious medical or psychiatric illness, language barrier, alcohol or illicit substance abuse or non-adherence
22. Addictions or other illnesses that do not allow the person concerned to assess the nature and extent of the clinical trial and its possible consequences
23. Simultaneous participation in another interventional clinical trial
24. Pregnant or breastfeeding women
25. Women of childbearing potential, except women who meet one of the following criteria:
    a) post-menopausal (12 months natural amenorrhoea)

b) postoperative (6 weeks after bilateral ovarectomy with or without hysterectomy, bilateral salpingectomy)
c) regular and correct use of a contraceptive method with an Pearl Index < 1% per year
d) sexual abstinence
e) Vasectomy of the partner

5 INVESTIGATIONAL MEDICINAL PRODUCTS

5.1 IDENTIFICATION AND DESCRIPTION OF INVESTIGATIONAL MEDICINAL PRODUCTS (IMP(S))

Tacrolimus

TAC is a macrolide calcineurin inhibitor. TAC is indicated for the prophylaxis of organ rejection, in adult and pediatric patients receiving allogeneic kidney, liver transplants and heart transplants, in combination with other immunosuppressants. TAC is approved for its intended use in this clinical study by all national competent regulatory authorities of the participating countries.

In T-cells, activation of the T-cell receptor normally increases intracellular calcium, which acts via calmodulin to activate calcineurin. Calcineurin then dephosphorylates the transcription factor nuclear factor of activated T-cells (NF-AT), which moves to the nucleus of the T-cell and increases the activity of genes coding for IL-2 and related cytokines. TAC prevents the dephosphorylation of NF-AT. In detail, TAC reduces peptidylprolyl isomerase activity by binding to the immunophilin FKBP12 (FK506 binding protein), creating a new complex. This FKBP12–FK506 complex interacts with and inhibits calcineurin, thus inhibiting both T-lymphocyte signal transduction and IL-2 transcription.

TTV R-GENE®

The TTV R-GENE®, using real-time PCR technology after extraction of the viral DNA, enables to detect and quantify the genome of TTV in whole blood, plasma and serum samples (Kulifaj et al. Journal of clinical virology. 2018; Mcculloch et al. 22nd Annual Meeting of the European Society for Clinical Virology, Copenhagen. 2019). The TTV R-GENE® kit is CE certified.

TTV, classified in the Anelloviridae family, is a non-enveloped, single-stranded, and circular DNA virus. TTV is a member of the Alphatorquevirus genus, which includes at least 20 human species. Human Anelloviridae are also represented by the Torque teno mini virus (TTMV) and the Torque teno midi virus (TTMDV), belonging to the Betatorquevirus and Gammatorquevirus genera, respectively. The highly diverse anellovirus family has been identified as one of the main components of the human blood virome; its prevalence is very high, usually more than 90%. Multiple infections with different TTV species are acquired over time, starting early in infancy, and result in lifelong viremia. This virus is transmitted through many channels: blood, oral, fecal, sexual, mother to child, among others. To date, TTV has not been clearly associated with any clinical manifestation and the clinical utility of TTV relies on its relationship with the functional immune status of the individual (De Vlaminck et al. Cell. 2013; Focosi et al. Clinical microbiology and infection. 2016).

5.2 SIDE EFFECTS AND INTERACTIONS

The reference safety information is listed in the SmPCs and are provided to the trial site with the investigator site file (ISF). The most common adverse reactions observed in TAC-treated kidney transplant patients are: infection, tremor, hypertension, abnormal renal function, constipation, diarrhoea, headache, abdominal pain, insomnia, nausea, hypomagnesemia, urinary tract infection, hypophosphatemia, peripheral oedema, asthenia, pain, hyperlipidaemia, hyperkalaemia, and anaemia.

- Patients receiving TAC, are at increased risk of developing lymphomas and other malignancies, particularly of the skin. Post-transplant lymphoproliferative disorder has been reported in immunosuppressed organ transplant recipients. Cases of pure red cell aplasia have been reported in patients treated with TAC.
- Patients receiving TAC are at increased risk of developing bacterial, viral, fungal and protozoal infections, including opportunistic infections. These infections may lead to serious, including fatal, outcomes. Serious viral infections reported include PVAN, mostly due to BK virus infection and CMV infections.
- TAC was shown to cause new onset diabetes mellitus.
- TAC can cause acute or chronic nephrotoxicity. TAC may cause a spectrum of neurotoxicities. The most severe neurotoxicities include posterior reversible encephalopathy syndrome, delirium, seizure and coma; others include tremors, paresthesias, headache, mental status changes, and changes in motor and sensory functions.
- Hyperkalemia has been reported with TAC. Hypertension is a common adverse effect of TAC therapy. TAC may prolong the QT/QTc interval and may cause Torsade de Pointes. Myocardial hypertrophy has been reported.

5.3 MANUFACTURING AND LABELLING

Standard TAC packaging/labelling/dosage form as provided by the vendors will be used. There will be no restriction on a certain TAC formulation or vendor/distributor. The TAC containing drug will be obtained as usual by the subjects themselves at their local pharmacies.

5.4 STORAGE, DISPENSE, RETURN AND DOCUMENTATION OF INVESTIGATIONAL DRUG (DRUG ACCOUNTABILITY)

Medications are handled according to standard practice. Batch numbers of TAC will be recorded.

5.5 ASSIGNMENT OF THE TREATMENT ARM

The assignment of a subject to a treatment arm is done by randomization, see 6.2.

5.6 TREATMENT PLAN

All patients will receive TAC based triple immunosuppression according to trial site routine standard. TAC in the active group will be guided by TTV as described in 5.7. There is no restriction to brand/formulation/rout of administration within this trial. TAC will be dosed according to TTV at every study visit up to visit 6. Follow up will end at month 13 post-transplant as detailed in 6.12.

5.7 INSTRUCTIONS FOR DOSE ADJUSTMENT

<u>Background:</u>
Applying the Vienna in-house PCR an increased risk for allo-graft rejection and infection was described for a TTV load outside of 6 $\log_{10}$ c/ml to 8 $\log_{10}$ c/ml in month 4 to 12 after kidney transplantation (Doberer K et al, American Journal of Transplantation, 2020). Applying this range to all available samples in the cohort described in the above cited publication in the same period, 41% of the measurements were below and 29% above the range. An upper cut-off of 7.6 $\log_{10}$ c/ml scored 38% of the samples above the range and thus leads to a more equal distribution (unpublished data). In preparation for this trial, we re-analysed TTV in the above

mentioned cohort using the Vienna in-house PCR and the PCR applied during the trial - the TTV R-GENE® assay - in parallel (unpublished data). The two assays were highly associated (slope: estimate 0.91, 95% CI 0.89 to 0.93; R 0.91) and the Bland-Altman plot revealed a mean difference of 1.4 $\log_{10}$ c/ml (95% CI of from -2.9 to 0.2). Applying this number the optimal TTV range for the trial was set at 4.6 $\log_{10}$ c/ml to 6.2 $\log_{10}$ c/ml.

5.7.1 ACTIVE/INTERVENTION GROUP

5.7.2 MAIN PRINCIPLES

TTV-guided TAC dosing: **The TAC trough level target will be adapted according to TTV copies per milliliter (c/mL) in the plasma (quantified by TTV R-GENE®) at study visit 1 to 6. The target TTV load ranges from 4.6 $\log_{10}$ c/mL to 6.2 $\log_{10}$ c/mL as defined by TTV R-GENE® (=optimal range).** TAC dosing according to TTV is detailed in Figure 3

1. If TTV is not within the optimal range, the TAC trough level target has to be adapted by one step up or down compared to the current TAC trough level.

If TTV is below the optimal TTV range, the TAC trough level target has to be increased by one step compared to the current TAC trough level, if TTV is above the optimal TTV range, the TAC trough level target has to be decreased by one step compared to the current TAC trough level.

2. One TAC trough level adaption step is defined as 2 ng/mL (investigators are allowed to target a range of +/-1 ng/mL; thus one step might be within a minimum of 1 ng/mL and a maximum of 3 ng/mL).

Dosing example

The patient has a current TAC trough level of 7.2 ng/mL. The TAC dose has to be adapted to target 5.2 ng/mL TAC trough level (= 4.2 to 6.2 ng/mL range) if the current TTV load is above the optimal range limit of 6.2 $\log_{10}$ TTV c/mL and adapted to 9.2 ng/mL TAC trough level (=8.2 to 10.2 ng/mL range) if the current TTV load is below the optimal range limit of 4.6 $\log_{10}$ TTV c/ml, respectively.

Figure 3 TAC Dosing According to TTV Range.

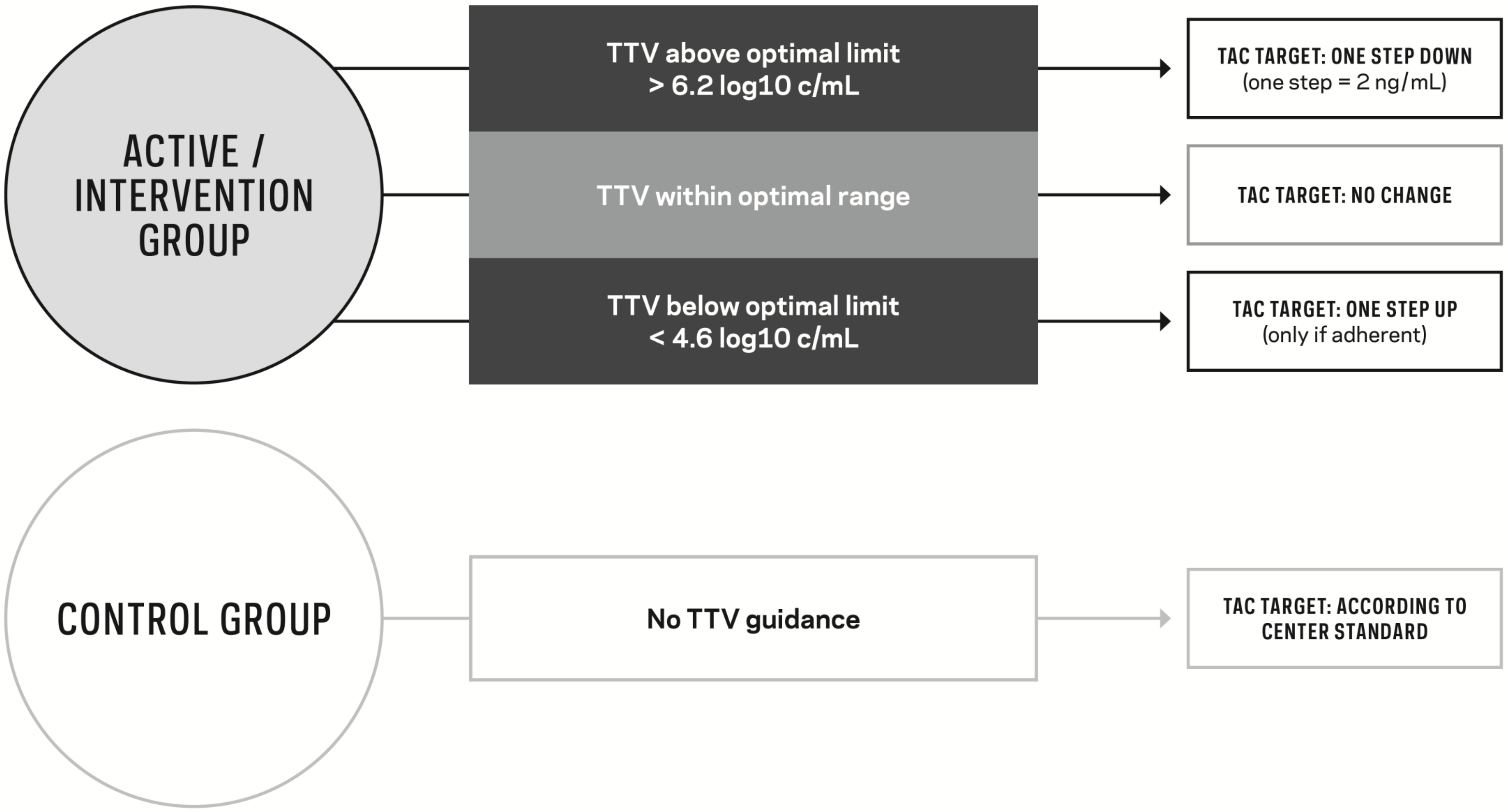

5.7.3 ADDITONAL RULES

- **All changes in TAC dose (including all non-study visits) must be performed according to the study protocol and the current TAC trough level target.**
- **TAC adaptions have to be made within 48 hours after the receipt of the TTV load.**
- **The lowest TAC trough level target is 3.5 ng/mL, the highest is 10 ng/mL.**
- **The treating physician can request additional TAC trough levels at any time (also on non-study visits) but TTV load will only be assessed and TAC trough level target changes only performed at the study visits.**
- **If non-adherence is suspected, no increase in TAC trough level target will be performed:** E.g. a failure of TAC intake more than once per week on average for b.d. and once every two weeks on average for o.d. formulation detected by the patient diary. In this case the patient should be educated about medical adherence (e.g. psychological counseling) and the target TAC trough level will not be changed according to TTV load until the next study visit.

5.7.4 HINTS FOR INDIVIDUALIZATION OF TAC DOSAGE

One TAC trough level adaption step is defined as 2 ng/mL (investigators are allowed to target a range of +/- 1 ng/mL; thus one step might be within a minimum of 1 ng/mL and a maximum of 3 ng/mL). This allows for additional individualization and accounts for limited granularity of TAC dosage formulation. **Additional individualization might be necessary in the following clinical scenarios:**

- **Absolute changes in TAC dose might have different effect sizes on the TTV load.** A change >20% in TAC dose is expected to show significant changes in the TTV load. Thus the upper range of 3 ng/mL might be targeted in case of high TAC intake and the lower range of 1 ng/ml in case of low TAC intake.
- **Significant changes in TTV load are expected 6 weeks (= 1 visit) after a significant change in immunosuppression has been performed. However, the full effect might take up to 12 weeks (= 2 visits).** If a change in the TAC target has been performed at the visit before and another TAC change in the same direction is suggested at the current visit, the lower TAC step range of 1 ng/mL might be targeted.
- **Simultaneous relevant changes in immunosuppression other than TAC might influence the subsequent TTV load** and the risk of infection/rejection. E.g. if mycophenolic acid has to be stopped and the TTV load indicates a TAC decrease, a TAC target change of 1 ng/mL might be performed.
- **The peak TTV level is at month 3 to 4 post-transplantation; thereafter the TTV load shows a gentle decline towards month 12 post-transplantation.** If the TTV load indicates a reduction in the TAC target at Visit 1, but the TAC level is within the local centre range, a TAC target reduction of 1 ng/mL might be performed.

5.7.5 ENDPOINT RELATED TREATMENTS

If subjects experience rejection as defined by the primary end point, severe infection (life or organ threatening defined by investigator (e.g. need for ventilator assistance in CMV pneumonia; but not acute renal impairment during UTI) **or relevant BK infection** (e.g. presumptive PVAN defined by ≥10^4 c/mL BKV [or corresponding U/mL] in the plasma detected by PCR or biopsy proven PVAN), **the investigator may decide for participants of the active group to continue TTV-guided TAC dosing or to stopped and to continue using the protocol of the control arm (see stopping rules).**

If the investigator decides to continue, the following rules apply:

- **Rejection:**
  - if TTV load suggests TAC trough level target step down, this suggestion must not be followed and TAC trough level target will be chosen by the investigator (e.g. stay on TAC trough level target or increase TAC trough level target)
  - if TTV load suggests no change in TAC trough level target this suggestion may be followed or TAC trough level target may be chosen by the investigator (e.g. increase TAC trough level target)
- **Severe infection:**
  - If TTV load suggests TAC trough level target step up, this suggestion must not be followed and TAC trough level target will be chosen by the investigator (e.g. stay on TAC trough level target or decrease TAC trough level target)
  - If TTV load suggests no change in TAC trough level target this suggestion may be followed or TAC trough level target may be chosen by the investigator (e.g. decrease TAC trough level target)
- **Relevant BKV infection:**
  - If a TAC trough level target step down is indicated by TTV load, a 'reduction of TAC as a treatment response to BKV' is the preferred strategy.
  - If no TAC trough level target change is indicated by TTV load, a 'reduction of the antimetabolite as a treatment response to BKV' is the preferred strategy.
  - If a TAC trough level target step up is indicated by TTV load, it has to be ignored and a 'reduction of the antimetabolite as a treatment response to BKV' is the preferred strategy.
  - Investigators are encouraged not to use a 'switch from mycophenolic acid to azathioprine' as treatment response for BKV.
  - Introduction of IVIG is possible independently of the treatment strategy and the TTV load.

5.7.6 CONTROL GROUP

TAC will be dosed according to TAC trough target levels defined by the local centre standard. TAC will be quantified at every study visit. Additional TAC quantification may be ordered by the treating physician at any time.

The TTV load will be accessed but not revealed to the investigator. TTV results must not be entered in the hospital data system used for routine care but have to be entered in the eCRF by the personnel, who performed the TTV assessment. TTV results in the eCRF will only be visible for the treating clinical personnel in the active/interventional group.

5.7.7 GENERAL RULE

- **No change in TAC** dosing should be performed **if the TAC** trough level **does not reflect a steady state** e.g. due to:
  - Low TAC level due to a missed dose or earlier intake than usual the day before the blood draw.
  - High TAC level due to an intake at the morning before the blood was drawn or later than usual at the day before the blood draw.
  - Suspected drug or dietary interaction with TAC.
  - Suspected high TAC levels due to diarrhea.

In these cases, the TAC trough level has to be repeated within a week. If TAC level is back to a steady state, TAC dosing has to performed according to the allocated treatment group.

- **Corticosteroids and antimetabolites will not be dosed according to the TTV level.** Centres are encouraged to keep the antimetabolite dose stable.

5.8 EMERGENCY MEASURES

In the event of a serious adverse reaction (possibly due to the trial drug dosing) or an emergency, subjects have the opportunity to contact the trial site or to introduce themselves personally. After clinical assessment, the appropriate emergency measures are initiated there. In the event of a dosing related problem, a temporary dose adaption or stop of the trial shall be applied in accordance with chapter 5.7 and 6.21. In the event of emergency treatment outside of the trial site, the subject shall present the trial card. Unblinding is not necessary due to the study design (investigator not blinded to allocation).

5.9 CONCOMITANT MEDICATION

Subjects are not allowed to receive cyclosporine, mTor inhibitor or co-stimulation blocker (Belatacept) based immunosuppression or significant additional long term (e.g. weeks) immunosuppression (e.g. cyclophosphamide) or immune modulation (e.g. check-point inhibitors). This does not include therapy for organ rejection, PVAN or TMA. In addition, herbal preparations containing St. John’s Wort (Hypericum perforatum) or other herbal preparations should be avoided when taking TAC due to the risk of interactions that lead to decrease in blood concentrations of tacrolimus and reduced clinical effect of tacrolimus.

5.10 ADHERENCE

Medical adherence will be monitored prospectively by patient diary. Non-adherence is defined as failure of TAC intake more than once per week on average for b.d. and once every two weeks for o.d. formulation. If missing entries of medical intake in the patient diary suggests non-adherence, the trial personnel has to verify if this is due to true non-compliance or due to inaccurate completion of the patient diary. Additional electronic drug monitoring using MEMS® Buttons (AARDEX Group, Switzerland) on TAC blisters, the BAASIS questionnaire, claimed prescriptions, psychological evaluation and TAC trough level variability will be accessed retrospectively.

5.11 BLINDING AND EMERGENCY ENVELOPES

5.11.1 BLINDING

The study will be conducted using a single-blind design (participants blinded). Assessors of the primary outcome will also be blinded to allocation: all episodes of infection and allograft rejection will be assessed by personnel blinded to the randomization code (infection: two infectious disease specialists; rejection: two kidney pathologist; Vienna based). The randomization code will be kept with the sponsor. No clinical staff member has access to the treatment randomization codes. Unblinding is not necessary due to the study design (investigator not blinded to allocation).

5.11.2 EMERGENCY ENVELOPES

Unblinding is not necessary due to the study design (investigator not blinded to allocation).

5.11.3 PREMATURE UNBLINDING IN AN EMERGENCY

Unblinding is not necessary due to the study design (investigator not blinded to allocation).

5.11.4 REGULAR UNBLINDING

Regular unblinding for the assessors and patients is performed after the end of the clinical trial and data evaluation (closing of the database).

6 COURSE OF THE CLINICAL TRIAL

6.1 IMPLEMENTATION OF INFORMED CONSENT

Potential study patients will be informed about the trial (aims, methods, implications, benefits and risks of the trial, voluntary nature of participation, right to refuse participation, samples and data collection and procedures for unexpected events) in terms they can fully understand, and written informed consent will be obtained prior to inclusion.

As a general starting point, patients are entitled to:

- understand that participation is voluntary.
- ask questions and receive understandable answers before making a decision.
- know the degree of risk and burden involved in participation.
- know who will benefit from participation.
- know the procedures that will be implemented in the case of incidental findings.
- receive assurances that appropriate insurance coverage is in place.
- know how their biological samples and data will be collected and protected during the trial and either destroyed or reused at the end of the trial.
- know of any potential commercial exploitation of the research.

All consent forms will be signed following the general rule that in the prior information-giving session, the patients concerned were given written and verbal information on the study by an investigator. At the end of the session, they are specifically asked whether they have understood all the relevant aspects of the study described and whether they are aware of their rights and duties as study participants. The patient is not asked to sign the consent form until he/she confirms that he/she has no further questions and wants to voluntarily participate in the study and to adhere to the protocol and the required long-term follow-up. The patient concerned can choose to do so directly after the information-giving session or later, after an appropriate period for reflection. The consent documentation will be in the original languages of the patients to be recruited for the study. When submitting the application for review by the competent local/national ethical boards/bodies for authorization, detailed information will be provided on the informed consent procedures that will be implemented. Copies of examples of informed consent forms and information materials will be included. These will be in language and terms understandable to the participants.

The informed consent materials will include, inter alia, the following data:

- a statement that the study involves research subjects;
- an explanation of the purpose of the research and the expected duration of the subject's participation;
- a description of the procedures to be followed and identification of any procedures that are experimental;
- a description of any reasonably foreseeable risks or discomforts to the subject;
- a description of any benefits to the subject or to others that may reasonably be expected from the research;
- a disclosure of appropriate alternative procedures or courses of treatment that might be advantageous to the subject;
- a statement describing the extent, if any, to which confidentiality of records identifying the subject will be maintained;

- for research involving more than minimal risk, an explanation of whether there are any treatments or compensation if injury occurs and, if so, what they consist of, or where further information may be obtained;
- an explanation of whom to contact for answers to pertinent questions about the research and research subjects' rights, and whom to contact in the event of a research-related injury to the subject;
- a statement that participation is voluntary, that refusal to participate will involve no penalty or loss of benefits to which the subject is otherwise entitled and that the subject may discontinue participation at any time without penalty or loss of benefits to which the subject is otherwise entitled.

In order to maintain data subject privacy, data will be identified by study number only. The investigator will keep a separate log of subject names and addresses in a secure place. However, in compliance with Good Clinical Practice, the study monitor may review the part of the subject's medical record that is directly related to the study. Prior to implementation, any amendments to the protocol that have subject safety implications must be approved by the appropriate IRBs and other competent regulatory authorities. Measures that are critical for subject safety may be implemented simultaneously with appropriate and timely reporting to the relevant IRBs and the competent regulatory authorities.

Incidental findings:

In principle, patients have the right to decide whether they are informed about incidental findings or not. The majority of the examinations are routine clinical practice and would also be performed independently of the study. Therefore, information about the handling of incidental findings must be provided in accordance with the prevailing clinical standard. No incidental findings are expected as a result of the TTV test. The central reporting (pathology and infection) is only used for endpoint recording and is not communicated back to the centers and patients, thus incidental findings do not exist. For these reasons, explicit study-specific information on incidental findings is not necessary.

All subjects have the right to withdraw at any time during the study without prejudice and this will be communicated clearly and often. There will be continuous safety and efficacy follow-up for each subject. The following circumstances constitute signs for withdrawal of the patient, and participating researchers will be alert to these contexts:

- withdrawal of consent by the patient/legal representative;
- any clinical condition that, in the investigator’s opinion, could constitute a risk to the patient and that would prevent the appropriate conduct of the trial; and
- loss of capacity to consent.

6.2 REGISTRATION/RANDOMIZATION

Participants will be allocated at a ratio of 1:1 using random permuted blocks with variable block size, stratified by recruiting centre. Eligible patients will be randomized 1:1 to either

- Torque Teno virus-guided immunosuppression (arm T) or
- Standard dosing (arm S).

eCRF-based randomisation will be used. Patients will be registered at screening.

Randomization occur at visit 1 (post-transplant month 4) after confirming the inclusion/exclusion criteria. Randomization list will be created via the program nQuery-Advisor® and included in the eCRF. Implementation according to the allocation sequence will be performed by the trial personnel.

Randomization will be conducted only if the patient can be included in the study (checking of inclusion- and exclusion criteria, written consent of the patient).

6.3 SCREENING V-3 (AT THE WARD IN THE FIRST WEEK POST-TRANSPLANTATION; LATEST UNTIL DISCHARGE)

Screening is the process of determining whether a person is suitable for inclusion in a clinical trial. For this purpose, the following information is recorded or examinations are carried out after the consent of the trial subject has been obtained (see 6.1):

Required information:

- Information and written informed consent of the subject,
- Preliminary inclusion / exclusion criteria check

Required investigations:

- Laboratory workup (TTV R-GENE®)
- Biobanking

6.4 SCREENING V-2 AND V-1 (AT ROUTINE VISIT IN MONTH 2 AND 3 POST-TRANSPLANTATION)

Required investigations:

- Laboratory workup (TTV R-GENE®; TAC-trough level; chemistry; CBC; urine analysis)
- Biobanking

6.5 VISIT 1 (DAY 0; AFTER DAY 93 POST-TRANSPLANTATION, WITHIN MONTH 4 POST-TRANSPLANTATION)

After the final assessment of the inclusion and exclusion criteria, the subject is admitted to the trial and randomized if suitable (see 6.2).

The following information is recorded or investigations are performed:

Required information:

- Inclusion/Exclusion criteria
- Medical history
- Medication
- Vital signs
- Physical examination, body weight
- Pregnancy test

Required investigation:

- Laboratory workup (TTV R-GENE®; TAC-trough level; chemistry; CBC; vBGA; urine analysis; BKV and CMV; DSA)
- Biobanking
- Quality of life (SF-36; MTSOSD-59R)
- Drug adherence (MEMS® BUTTON; patient diary; BAASIS, psychological evaluation)
- Data sampling concerning protocol biopsy performed according to local centre standard; protocol biopsy has to be performed before V1

Required Intervention:

- Adaption of TAC dose

6.6 VISIT 2 (WEEK 6; ± 14 DAYS)

Required information:

- Vital signs
- Medication
- Adverse events
- Primary endpoint

Required investigation:

- Laboratory workup (TTV R-GENE®; TAC-trough level; chemistry, CBC, vBGA; urine analysis; BKV and CMV)
- Biobanking
- Drug adherence (MEMS® BUTTON; BAASIS; patient diary)

Required Intervention:

- Adaption of TAC dose

6.7 VISIT 3 (WEEK 12; ± 14 DAYS)

Like Visit 2

6.8 VISIT 4 (WEEK 18 ± 14 DAYS)

Like Visit 2

6.9 VISIT 5 (WEEK 24; ± 14 DAYS)

Like Visit 2.

6.10 VISIT 6 (WEEK 30; ± 14 DAYS)

Like Visit 2.

6.11 VISIT 7 (FINAL VISIT: WEEK 36 ± 14 DAYS)

Required information:

- Vital signs
- Physical examination, body weight
- Pregnancy test
- Medication
- Adverse events
- Primary endpoint

Required investigation:

- Laboratory workup (TTV R-GENE®; TAC-trough level; chemistry, CBC; vBGA; urine analysis; BKV and CMV; DSA)
- Biobanking
- Drug adherence (MEMS® BUTTON; BAASIS; patient diary; claimed prescription check, psychological evaluation)
- Quality of life (SF-36; MTSOSD-59R)
- Data sampling concerning protocol biopsy performed according to local centre standard

6.12 FOLLOW-UP

The final adaption on TAC range will be perfumed on visit 6. FUP will last for 6 weeks until visit 7 (last visit).

6.13 ROUTINE CALLS

Routine calls every two weeks will help to detect infections treated outside the study centre.

6.14 DEVIATIONS FROM THE PROTOCOL

The principal investigator is responsible for ensuring that the clinical trial is conducted at the trial site in accordance with the trial protocol. If there are nevertheless deviations from the valid protocol, specified procedures, GCP and/ or the applicable regulatory requirement(s), these must be documented by the trial site, including a clear description and justification.

Subject-related deviations are documented in the eCRF. Subject-independent deviations are documented on a separate form, which is stored in the ISF.

Deviations from the protocol are e.g.:

	**minor**	**major**	**critical**
Deviations in relation to the inclusion or exclusion criteria			X
Examinations/visits that were missed		X	
Examinations/visits that did not take place within the given time window	X		
Non-performance of laboratory analyses in regard to TTV-level determination		X	
Non-Adherence in regard to IMP intake		X	
Incorrect administration of the IMP (dosing)		X	
Intake of expired IMP		X	

Excluded concomitant medication has been taken by the patient		X	
Treatment not as randomized			X
Incorrect adjustment of the IMP according to TTV-level in the TTV guided group		X	
Deviations that have led to an (S)AE		X	

Management of Serious Breaches

A `serious breach` means any deviation of the Regulation (EU) 536/2014 or of the approved version of the protocol applicable that is likely to affect to a significant degree

(A) the safety and rights of a subject and/or
(B) the reliability and robustness of the data generated in the clinical trial.

The sponsor will notify the Member States concerned about a serious breach via the EU portal not later than seven days after becoming aware of that breach. Serious breaches will be documented in the eCRF.

6.15 ASSESSMENT OF EFFICACY

Components of the primary endpoint will be accessed at every study visit. In addition check-ups on patients concerning infections and AEs will be performed via telephone. Timing of assessment is detailed in chapter 6 and methods of assessment are detailed in chapter 5.

6.16 ASSESSMENT OF SAFETY

6.16.1 SPECIFICATION OF SAFETY PARAMETERS

- Death
- Graft loss
- Infection and rejection according to the primary and secondary endpoints
- Acute kidney injury (>grade I according to the Acute Kidney Injury Network)
- New onset of diabetes mellitus (according to the American Diabetes Association)
- Fluid overload (>5% increase in body weight)
- Hypercholesterolemia (LDL >160mg/dl) and hypertriglyceridemia (>150mg/dl)
- Arterial hypertension (>140/90 mm Hg)
- Cardiovascular event (myocardial infarction, unstable angina, stroke, peripheral artery disease >grade III, hospitalisation due to cardiac decompensation)
- Prolongation of the QTc interval (>500 ms) and Torsade de Pointes tachycardia
- Hyperkalaemia (>6 mmol/L)
- Nausea, vomiting, diarrhoea, constipation
- Posterior reversible encephalopathy syndrome, delirium, seizure and coma, tremors, paraesthesia, headache, mental status change, and changes in motor and sensory function, insomnia, asthenia and pain
- Anaemia (Hb<6mg/dl)
- Malignoma

6.16.2 THE METHODS AND TIMING FOR ASSESSING, RECORDING, AND ANALYSING SAFETY PARAMETERS.

Safety parameters will be assessed at every study visit. To detect TAC-related AEs and toxicity lab work will be performed according to routine clinical care at every study visit. In addition vital signs, neuropsychiatric, sensoria, cardiovascular, bronchial, gastrointestinal, dermatologic, musculoskeletal and urogenital alterations will be assessed at every study visit. Age-adapted tumor screening will be recommended to participants. Check-ups on patients concerning AEs and infections will be performed via telephone.

6.17 TRIAL-SPECIFIC INVESTIGATIONS

Trial Procedures

Trial-specific and routine procedures during the interventional part of the trial period are displayed in Table 1. During the screening phase of the trial, no trial-specific procedures except TTV quantification and sampling of biologic material for the biobank (sub-study) will be performed.

Medical history

A detailed medical history including relevant baseline and FUP data until randomization will be obtained. The following data will be noted: current medication, SARS-CoV 2 and Influenza vaccination status, recipient sex and date of birth, type of renal disease, history of renal replacement therapy, history of prior transplantation, history of diabetes, history of major cardiovascular, immunologic, and oncologic diseases and diseases currently requiring concomitant medication, CMV/EBV status (IgG), transplant date, donor type/age/sex, donor CMV/EBV status (IgG), donor and recipient HLA (ideally 4 digit typing, but sufficient to define DSA), HLA mismatch, CMV/PCP prophylaxis, initial immunosuppression, induction therapy, and graft rejection, rejection therapy, *de novo* DSA, infections and NODAT post-transplant until randomization.

Vital signs

Blood pressure, heart rate, respiratory rate and body temperature will be assessed.

Physical examination

A physical examination including assessment of body size and weight, auscultation of the lung and heart and palpation of the abdomen will be performed.

Pregnancy Test

A urinary dip β-HCG based test will be performed. The test can be omitted in post-menopausal (12 months natural amenorrhoea) women and women in a postoperative status 6 weeks after bilateral ovarectomy with or without hysterectomy, bilateral salpingectomy.

Medication

All immunosuppressive and antimicrobial prophylactic medication, medication for concomitant disease, and medications for treatment of AEs and the end-point, respectively will be noted including name, dose and schedule. All vaccinations will be noted.

Adverse events/ tacrolimus toxicity

To detect TAC-related AEs and toxicity, complete blood count, blood chemistry and venous blood gas analyses will be performed according to routine clinical care. In addition neuropsychiatric, sensoria, cardiovascular, bronchial, gastrointestinal, dermatologic,

musculoskeletal and urogenital alterations will be noted; specifically any nausea, vomiting, constipation, diarrhea, abdominal pain, tremor, paresthesia, headache, mental status change, and changes in motor and sensory functions, insomnia, asthenia, pain, edema, shortness of breath, chest pain and palpitation will be assessed. AEs will also be assessed using patient diaries.

Infection
All infections have to be documented as adverse events.

In general quality, onset and duration of symptoms, details on diagnostic, treatment in-patient stay, and response to treatment have to be noted.

Signs of infections which have to be documented as an adverse event include pain, night sweat, fever, chills, malaise, fatigue, diarrhea (frequency and consistency of the stool) abdominal pain/cramps, dysuria, pollakisuria, alguria, urinary urgency/frequency, suprapubic pain, flank/allograft pain (on palpitation), caught, sputum (purulent, with blood), adventitious breath sounds on auscultation/palpation, shortness of breath, and rapid/shallow breathing.

Infections concerning endpoints (see 3.2 and 3.3)

Infectious disease workup will be performed according to local standards. However, some diagnostic workup should be performed to obtain comparable findings:

- In case of suspected infection, the investigators should perform a minimum diagnostic set including CBC, CRP, blood pressure, heart rate, respiratory rate, and body temperature and document an altered mental status.

In addition the following diagnostic sets should be applied:
- In patients with fever: blood and urine cultures, urinary dip stick and CMV PCR from the blood.
- In patients with suspected urinary tract infection: urinary dip stick and urine cultures; if available ultrasound of the urinary track system.
- In patients with suspected respiratory infection: chest X-ray; if available: *legionella/ haemophilus* and *pneumococcus* urinary antigen and sputum or nasopharyngeal swab multiplex PCR.
- In patients with suspected diarrhea: stool cultures and CMV PCR from stool samples; if available: multiplex PCR from stool samples.

In general, all performed supporting diagnostic tests related to infections should be documented including vital signs (blood pressure, heart rate, respiratory rate, mental state, body temperature), laboratory (e.g. CBC, CRP, PCT, IL-6, creatinine/eGFR, blood urea nitrogen, blood pH, lactate, albumin), microbiologic and virologic results, imaging (e.g. X-ray, CT, MRI, PET, US, endoscopy), and qSOFA/SOFA score.

For the expected main infectious events the following parameters are of special interest:
- Urinary tract infection: results on urinary dip stick and cultures (blood and urine) and imaging (e.g. abdominal US and CT and cystoscopy).
- Respiratory infection: results on imaging (e.g. chest X-ray, CT and bronchoscopy), microbiological and virologic workup including light microscopy (gram- and ZN stains), cultures, cytokine release assays, antigen tests and PCRs from blood, urine and respiratory material; albumin, respiratory rate, heart rate, peripheral oxygen saturation and arterial Ph, and neurological status.

- Diarrhea: results on imaging (e.g. CT abdomen and endoscopy) and microbiological and virologic work up including cultures, antigen tests and PCRs.
- BKV: results on blood and urine BKV PCR, decoy cells and kidney graft biopsy results.
- CMV: results on imaging (e.g. CT/MRT and endoscopy) and CMV PCRs from liquor, urine, blood, stole and respiratory material.

Investigators will be able to submit additional information in form of brief commentaries to give context in complex cases. These commentaries will only be available to the blinded assessor if explicitly unlocked by request. The assessor may query the site if additional information for clarification if needed. Test results of examinations used to identify the focus of an infectious episode (e.g. ultrasound, radiography, endoscopy, explorative surgery) should be submitted in writing.

Timing of data sampling

In addition to the trial visits, **patients will be contacted in 2 week intervals via phone** by the trial team who will ask for episodes of infection (duration and onset of symptoms, diagnosis, treatment and inpatient stay) and signs of infection (pain, fever, night sweats, cough, dysuria, and diarrhea).

Out of centre treatment

Patients should make an appointment at their trial center if they feel they are suffering from an infection. If they seek medical advice concerning an infection or receive treatment for an infection outside the trial centre, the trial centre should contact the treatment center. The local trial team should thrive to acquire all infection relevant documentation. To ensure this, each patient will receive an ID card with a reminder to follow these procedures and contact details of the trial centre.

Allograft biopsy

If patients have an allo-graft biopsy or receive rejection treatment outside the trial centre, the treatment centre should be contacted. The local trial team should thrive to acquire all relevant documentation. To ensure this, each patient will receive an ID card with a reminder to follow these procedures and contact details of the trial centre.

Indication biopsies will be evaluated by applying standard center methodology (including HE, PAS, Trichrom, S/AFOG, silver stain and IHC). For protocol biopsies at month 12 additional molecular evaluation will be performed by the Molecular Microscope Diagnostic System (MMDx) at the Alberta Transplant Applied Genomics Centre (ATAGC, University of Alberta, Edmonton, AB, Canada). For this purpose 5 mm biopsy material will be separated, fixed in RNA-later in RNase-free cryotubes and shipped. Details concerning sampling, preparation, storage and shipping of protocol biopsies is described in a dedicated manual.

A biopsy is only counted as protocol, if, at this time point, no clinical signs of rejection are observed. Protocol biopsies are not mandatory, but data concerning month 12 protocol biopsies will be obtained if performed. Some centers have incorporated protocol biopsies in their clinical standard (at month 12 after transplantation in Vienna, Regensburg and Dresden).

All episodes of allograft biopsy will be assessed in addition by personnel blinded to the randomization code (two pathologists) following predefined protocols based on current guidelines (Banff 2019 Kidney Meeting Report published by the American Society of Transplantation). For this purpose, histological slides of graft biopsies will be scanned and sent to the evaluator via Cy-

tomine. In order to facilitate centralized assessment information concerning DSA, BK viraemia and date of transplantation and biopsy have to be provided.

Tumor Screening
Tumor screening will be performed not as part of the study protocol, but according to local centre standards. Any oncologic disease will be documented (type of tumor and date of diagnosis).

TTV Quantification
TTV will be quantified in peripheral blood EDTA plasma using the TTV R-GENE® (bioMérieux). BioMérieux, has set up TTV R-GENE® at all participating sites with a customized quality assessment program.

TTV results for the clinical trial staff should generally be available within 24 hours after the study visit. Therefore samples have to arrive at the laboratory performing the PCR no later than 11:00 a.m. Monday to Thursday. For samples arriving after 11:00 a.m., TTV results might only be available <48h after the study visit. For samples arriving at Friday, TTV results might only be available on the following Monday. Arrival times and sampling days might be adapted locally to optimize timing of TTV analyses.

EDTA blood (collected in 6mL EDTA tubes) will be drawn and sent immediately to the local virology lab. EDTA plasma will be separated (a minimum of 2ml) immediately and nucleic acids from EDTA plasma (200 µL) will be extracted using various platforms, amplification will be performed on various platforms according to data sheet. Excess EDTA plasma will be stored at the local virology lab at -20C° and, if necessary, shipped for quality control to Spallanzani National Institute for Infectious Diseases, Rome, Italy. Details concerning sampling, transport, preanalytics, analytics, data flow, storage and shipping of EDTA plasma are described in the TTV R-GENE® data sheet.

If unacceptable delays of TTV quantification are anticipated (>72h e.g. due to technical problems) bioMérieux and the sponsor represented by the principal coordinating investigator have to be contacted. The principal coordinating investigator will organize shipment to an alternative study center and data exchange.

TTV results must not be entered in the hospital data system used for routine care but have to be entered in the eCRF by the personnel, who performed the assessment. Source data should be printed out, have to be kept with the TTV assessor and sent to the PI for monitoring purpose when requested or latest at end of the trial.

Additional TTV quality control
Universal real-time PCR for quantitative TTV detection, real-time PCRs for quantitative detection of human TTMV and TTMDV, species-specific real-time PCRs for quali-/quantitative characterization of TTV genetic variability and NGS analysis will performed on selected samples with discrepancies between clinical events and TTV load in Rome, Italy.

Tacrolimus trough level quantification
Tacrolimus trough level will be quantified according to local centre standard.

DSA Monitoring
If not part of the routine post-transplant care a DSA monitoring on the basis of a single fluorescence bead assay has to be done.

Other Laboratory Workup and Virology Screening
Laboratory workup and virology screening will be performed according to local centre standards. However, according to study protocol, at least leukocyte count, creatinine and urinary protein- and albumin to creatinine ratio will have to be assessed. Findings concerning CMV and BKV screening including CMV and BKV PCR performed routinely at the centres will be noted.

Health-related Quality of Life
For the assessment of health-related quality of life, the Medical Outcomes Study Short Form 36 SF-36) and the Modified Transplant Symptom Occurrence and Symptom Distress Scale-59 Items Revised (MTSOSD-59R) will be performed.

Monitoring Subject Adherence
Medical adherence will be monitored by patient diary and with electronic drug monitoring using MEMS® Buttons (AARDEX Group, Switzerland) on TAC blisters, the BAASIS questionnaire, claimed prescriptions, psychological evaluation and TAC trough level variability.

Psychological Evaluation

A questionnaire including sociological status, education, employment, addictions, critical life events, and history of psychiatric illness will be performed.

Calls
Routine calls every 2 weeks will be performed to detect infections treated outside the study center: Calls will be performed according to preferred contact numbers and times with a minimum of 3 calls at two different days at varied times if contact cannot be established. Patient will be asked for episodes of infection (symptoms including fever, night sweats, cough, dysuria, and diarrhea, duration and onset of symptoms, date of diagnosis, treatment, and inpatient stay).

Patient Diary
Diaries will be filled by the patients including TAC intake and occurrence of AEs.

6.18 BIOMATERIALS

Biological material will be stored at each individual centre for the sub-study. Whole blood (2x2.5 mL PAXgene tubes), serum (8mL), plasma (1x8 mL and 2x9mL EDTA tubes) and urine (10mL) will be sampled at all visits including screening for biobanking. In addition, whole blood (4mL) will be sampled at visit 1. Details concerning sampling, storage and shipping of biological material are described in a dedicated manual.

6.19 RESCUE THERAPY

Kidney transplant rejection and infections will be treated in accordance to local centre standard therapy.

6.20 FURTHER TREATMENT AFTER COMPLETION OF THE TRIAL

In any clinical situation, including emergencies, the appropriate medical care will be provided. After the study, medical follow-up will be provided for all participants at the study centre or suitable facilities according to local standard. In case of AE all necessary medical care will be provided for all participants at the study centre or suitable facilities even after data collection for the research study is completed.

6.21 PREMATURE TERMINATION OF TRIAL THERAPY FOR A SUBJECT

The trial therapy has to be terminated prematurely for the following reasons:

- at the subjects own request
- if the Investigator feels it would not be in the best interest of the subject to continue
- pregnancy
- non-adherence with the trial treatment as detailed in 5.10.
- adverse event that requires the termination of the trial treatment (CTCAE grade >3)
- introduction of mTOR inhibitors, co-stimulation blockers or cyclosporine
- necessity of significant additional long term immunosuppression or immune modulation (as defined by the PI; e.g. for autoimmune disease or cancer; this does not include rejection therapy or IVIG for PVAN)
- necessity of long term stopping of TAC (> than one week; E.g. during PTLD therapy)
- HIV infection
- cancer (as defined by the secondary outcome)
- any condition that needs significant higher/lower than usual TAC target range as defined by the PI (e.g. TMA necessitating low dose TAC or lung transplant necessitating higher dose TAC)
- graft loss

The trial therapy **might be** terminated prematurely for the following reasons:

- rejection as defined by the primary end point
- severe infection (life or organ threatening defined by investigator)

In these circumstances, the PI decides if TTV-guided TAC dosing will be stopped and the patient will continue the study following the protocol of the control arm.

Further visits and trial specific procedures will be continued in the event of premature termination of the trial therapy. The documentation of all subsequent visits should still be pursued.

6.22 PREMATURE TERMINATION OF THE CLINICAL TRIAL FOR A SUBJECT

Subjects must be withdrawn from the trial under the following circumstances:

- at their own request
- death

In all cases, the reason why subjects are withdrawn must be recorded in detail in the CRF and in the subject's medical records. Should the study be discontinued prematurely, all study materials will be retained. Data will be collected to the point of withdrawal and used for the intention-to-treat analysis if subject consent. There will be no replacement of withdrawn subjects. For subjects withdrawing prematurely from the clinical trial, a final visit (according to visit 7) should be sought and the results documented in the eCRF. The subjects may request that all biological samples collected in the course of the study will be destroyed. The PIs will take care, that the patient will receive routine post-transplant care after withdrawal.

6.23 PREMATURE TERMINATION OF THE CLINICAL TRIAL

The decision to discontinue the trial is made by the principal coordinating investigator (PCI) and the Sponsor in consultation with the Data Safety Monitoring Board (DSMB). The Sponsor shall report the early termination of the clinical trial to the competent authorities and ethics committees within 15 days, stating the reasons for the termination. The reasons for termination

of the trial are documented in detail. Subjects who were in treatment at the time of termination of the trial must undergo a final examination, which is documented in the eCRF according to visit 7. Should the study be discontinued prematurely, all study materials will be retained. Data will be collected to the point of termination and used for the intention-to-treat analysis. The PIs will take care, that the patient will receive routine post-transplant care after withdrawal.

Reasons for premature termination of the clinical trial may be:

- low recruitment rate
- high dropout rate
- low event rate
- serious, unresolved problems with the quality of the data collected
- unforeseeable circumstances at the respective trial site that do not allow the continuation of the clinical trial
- safety signal according to safety analysis of the DSMB
- unacceptable risks and toxicities (decision after new benefit-risk assessment)
- new scientific evidence during the period of the trial which does not allow it to continue

If an investigator has ethical concerns regarding the continuation of the trial, the PCI must be informed immediately.

The clinical trial is embedded in an EU H2020 sponsored project (TTV GUIDE TX). Premature termination of the clinical trial will be discussed with the projects core team (steering committee; SC) and all project partners within the general assembly of the project.

DSMB safety monitoring

Details concerning the analysis performed by the DSMB are outlined in 7.4. The DSMB will report the conclusions of safety analysis to the PCI and the sponsor. For this trial, no statistical stopping rules will be used.

7 SAFETY/PHARMACOVIGILANCE

7.1 DEFINITIONS

An **Adverse Event (AE)** is any adverse medical occurrence to a subject being administered an IMP and that is not necessarily causally related to that treatment.

- These may be diseases, signs of disease, clinically significant laboratory values or symptoms that occur after the person is included in the trial.
- Also the worsening of a pre-existing disease is to be regarded as AE in this context.

An **adverse reaction (AR)** is any adverse and unintended reaction to an IMP, regardless of its dosage. An adverse reaction is defined as an event that is considered to be at least possibly related to the IMP.

An **Unexpected Adverse Reaction (UAR)** is an adverse reaction that does not match the type or severity of the present reference information (e.g., Investigator's Brochure or SmPC) on the investigational product.

A **Serious Adverse Event (SAE)** or **Serious Adverse Reaction (SAR)** is any adverse event or reaction that:

- results in death, or
- is life-threatening, or
- leads to permanent or serious disability or invalidity, or
- requires inpatient treatment or its prolongation, or
- leads to congenital malformations or birth defects, or
- is medically significant for other reasons (event in which medical intervention became necessary to prevent an outcome considered serious).

A suspected case of an **Unexpected Serious Adverse Reaction** is called **Suspected Unexpected Serious Adverse Reaction (SUSAR)**. A serious adverse reaction is considered unexpected if its type, severity or outcome is not listed in the corresponding reference information. The section 4.8 “Undesirable effects” of the of the respective current valid SmPCs of Advagraf (prolonged release), Prograf (capsules hard) and Prograf (concentrate for solution) serve as reference safety information for purpose of SUSAR reporting.

7.1.1 PREGNANCY

Any pregnancy that occurs during study participation must be reported to the Investigator/sponsor. To ensure subject safety, each pregnancy must be reported to the Sponsor immediately. The pregnancy must be followed up to determine outcome (including premature termination) and status of mother and child. Pregnancy complications and elective terminations for medical reasons must be reported as an AE or SAE. Spontaneous abortions must be reported as an SAE.

Any SAE occurring in association with a pregnancy brought to the Investigator’s attention after the subject has completed the study and considered by the Investigator as possibly related to the investigational product, must be promptly reported to the Investigator/sponsor.

In addition, the Investigator must attempt to collect pregnancy information on any female partners of male study subjects who become pregnant while the subject is enrolled in the study. Pregnancy information must be reported to the Investigator/sponsor as described above.

7.2 DOCUMENTATION OF (S)AES

All adverse events, including intercurrent diseases, must be documented in the subject record and subsequently in the eCRF.

When an adverse event occurs, regardless of the causal relationship between the event and the IMP, the affected subject must always be monitored until symptoms subside or pathological laboratory values return to baseline, or until no further findings are expected in the opinion of the principal coordinating investigator (PCI). If the adverse event develops into a persistent secondary disease, it should be classified as an SAE and documented accordingly at the end of the trial. All findings and results must be documented both in the subject's record and on the corresponding adverse event form in the eCRF.

The following information is required:

- type of adverse event (sign, symptom or disease, if possible medical term),
- assessment of the (worsening of existing) concomitant diseases as an adverse event,
- classification (serious/ non-serious),
- start and end of occurrence,
- CTCAE Grade (Version 5.0, published 27.11.2017, NCI)
- causality to the IMP (not -, unlikely -, possibly -, probably -, certainly related, unknown or not assessable)
- action taken regarding the IMP (dose not changed, dose reduced, dose increased, temporarily interrupted, drug withdrawn, unknown, not applicable),
- action taken in order to restore or improve the well-being of the subject (none, drug treatment, other)
- outcome of the event (recovered, recovering, not recovered, recovered with sequelae, fatal, unknown).

7.2.1 SEVERITY OF ADVERSE EVENTS

The severity of clinical AEs is graded from 1 to 5 in accordance to the Common Terminology Criteria for Adverse Events (CTCAE; Version 5.0, 27.11.2017; NCI) and reported on specific AE pages of the eCRF. If the severity of an AE worsens during study drug administration, only the worst intensity should be reported on the AE page. If the AE lessens in intensity, no change in the severity is required. If an AE occurs during a washout or placebo run-in phase and afterwards worsens during the treatment phase, a new AE page must be filled in with the intensity observed during study drug administration.

Grade 1

Mild; asymptomatic or mild symptoms; clinical or diagnostic observations only; intervention not indicated.

Grade 2

Moderate; minimal, local or noninvasive intervention indicated; limiting age-appropriate instrumental activities of daily living (ADL).

Grade 3

Severe or medically significant but not immediately life-threatening; hospitalization or prolongation of hospitalization indicated; disabling; limiting self-care ADL.

Grade 4

Life-threatening consequences; urgent intervention indicated.

Grade 5

Death related to AE.

AE Grade 1 to 3 may or may not be serious. These terms are used to describe the intensity of a specific event. However, a severe event may be of relatively minor medical significance (such as severe headache) and is not necessarily serious. For example, nausea lasting several hours may be rated as severe, but may not be clinically serious. Fever of 39°C that is not considered severe may become serious if it prolongs hospital discharge by a day. Seriousness rather than severity serves as a guide for defining regulatory reporting obligations.

7.2.2 RELATIONSHIP TO STUDY DRUG

For all AEs, the Investigator will assess the causal relationship between the study drug and the AE using his/her clinical expertise and judgment according to the following algorithm that best fits the circumstances of the AE:

Not related

- May or may not follow a temporal sequence from administration of the study product
- Is biologically implausible and does not follow known response pattern to the suspect study drug (if response pattern is previously known).
- Can be explained by the known characteristics of the subject's clinical state or other modes of therapy administered to the subject.

Unlikely

- There is a reasonable temporal relation between the AE and the intake of the study medication, but there is a plausible other explanation for the occurrence of the AE.

Possibly

- The AE has a reasonable temporal relationship with drug administration.
- The AE may equally be explained by the study subject's clinically state, environmental or toxic factors, or concomitant therapy administered to the study subject.
- The relationship between study drug and AE may also be pharmacologically or clinically plausible.

Probably

- There is a reasonable temporal relation between the AE and the intake of the study medication, and plausible reasons point to a causal relation with the study medication.

Related

- Reasonable temporal relation between the AE and the intake of the study medication and
- There is no other explanation for the AE and
- Subsidence or disappearance of the AE on withdrawal of the study medication and
- Recurrence of the symptoms on restart at previous dose (only applies for re-institution of mediation).

Not assessable

- The causal relationship between the study drug and the AE cannot be judged.

7.2.3 SPECIAL FEATURES WITH SAES

The following points must be observed when documenting SAEs:

- the corresponding AE must be marked as **serious** in the eCRF.
- each SAE must be documented as completely as possible (in the event of death, an autopsy should be performed if possible and the report made available to the sponsor).

7.2.4 DEATHS

Deaths that are not due to AE must also be documented in the eCRF.

Safety parameters will be assessed at every study visit. To detect TAC-related AEs and toxicity lab work will be performed according to routine clinical care at every study visit. In addition vital signs, neuropsychiatric, sensoria, cardiovascular, bronchial, gastrointestinal, dermatologic, musculoskeletal and urogenital alterations will be assessed at every study visit. In addition to the age-adapted tumour screening recommended for the general population, annual skin examinations will be recommended to participants. heck-ups on patients concerning AEs and infections will be performed via telephone according to preferred contact numbers and times every two weeks. Multiple calls will be performed: a minimum of 3 at two different days at varied times. Safety parameters will be recorded in the eCRF.

7.3 REPORTING OBLIGATIONS

7.3.1 REPORTING OBLIGATIONS OF THE INVESTIGATOR

SAEs

The investigator shall notify the sponsor (or his designee(s)) immediately (normally **within 24 hours** of notification) of the occurrence of a SAE. The notification shall be made to pharmacovigilance by the investigator or a medical member of the trial group authorized by the investigator.

Electronic reporting via eCRF: After documentation of the SAE in the eCRF, the data is verified by electronic signature and reported to the pharmacovigilance department by means of an automatically generated e-mail. At the same time, the e-mail is sent to the reporting trial site. This e-mail is to be filed in the ISF as evidence of the SAE notification.

If the electronic system is temporarily unavailable, the SAE notification will be submitted on paper (form in ISF) via fax or e-mail to the Pharmacovigilance Department. As soon as the system is available again, the documentation in the eCRF must be made up for.

Follow Up

If further information on the SAE becomes available at a later date, it must also be reported immediately to the sponsor (or his representative). The trial site's report is checked for completeness and plausibility and, if necessary, further inquiries are made and followed up.

Death of a subject

In the event of the death of a subject, the trial site shall provide the competent ethics committee, the higher federal authority and the sponsor (or its authorised representative) with any additional information required for the performance of their duties (upon request).

7.3.2 REPORTING OBLIGATIONS OF THE SPONSOR

AEs

The Sponsor must document in detail all adverse events reported to him and, upon request, submit these to the Member States concerned.

SUSARs

The sponsor must inform the Member States concerned and the investigators involved in the clinical trial about every suspected case of an unexpected serious adverse drug reaction (SUSAR) that comes to his attention immediately, but at the latest **within 15 days** of its discovery.

For every suspected case of a suspected unexpected serious adverse reaction (SUSAR) reported to the sponsor, that has led to **death** or is **life-threatening**, the sponsor must immediately, but at the latest **within seven day**s after becoming aware of it, provide the Member States concerned and the investigators involved in the clinical trial with all information relevant to the evaluation and within a maximum of **eight further days** with the other relevant information.

Re-evaluation of the benefit-risk assessment

The Sponsor shall immediately, but **at the latest within 15 days** of its notification, inform the Member States concerned of any factual situation that requires a re-examination of the benefit-risk assessment of the IMP. This includes in particular:

- subject case reports of expected serious adverse reactions (SARs) with an unexpected outcome,
- increase of the frequency of expected serious adverse reactions (SARs) that are considered clinically relevant,
- suspected cases of serious unexpected adverse reactions (SUSAR) that occurred after the subject had already terminated the clinical trial,
- events related to the conduct of the trial or the development of the IMP that may potentially affect the safety of the subjects.

List of all SARs and safety report

For the duration of the clinical trial, the sponsor must submit to the Member States concerned **once a year or upon request**, a list of all suspected cases of serious adverse drug reactions (SARs) that have occurred during the trial as well as a report on the safety of the subjects (**D**evelopment **S**afety **U**pdate **R**eport, **DSUR**).

Measures to protect against an imminent danger

Where the safety of subjects is compromised and measures are taken by the Sponsor and the investigator to protect them from immediate danger, the Sponsor immediately informs the Member States concerned about these measures and the circumstances that trigger them.

7.4 DATA SAFETY MONITORING BOARD (DSMB)

An external and independent DSMB will be established to monitor and protect patient safety throughout the clinical trial, with an emphasis on progress, safety data and critical efficacy endpoints of the clinical study according to the DAMOCLES group.

To detect safety signals in a timely manner, the board members of the DSMB will be instructed to perform safety analyses after 65 and 130 included patients (if there are safety issues, the DSMB may ask for a higher frequency). They will evaluate dropout rate, inclusion rate, rate of

primary out-come and SAEs. SAEs and primary outcome will be analyzed according to the randomization sequence.

The DSMB may suggest stopping the trial if the overall pattern of related SAEs supports a major safety signal, such as:

- >80% of all the participants experience SAEs (CTCAE >Grade 3 or severe/medically significant) and >80% are in the intervention group.
- >80% of all the participants experience a clinically significant infectious disease as defined by the primary endpoint and >80% are in the intervention group.
- >30% of all the participants experience a clinically significant allograft rejection as defined by the primary endpoint and >80% are in the intervention group.
- >70% of all the participants experience an allograft rejection in the 12-month protocol biopsy as defined by the secondary outcome, and >80% are in the intervention group.

Analysis of the DSMB will stratify according to sex for early detection of sex related risks.

For this trial, no statistical stopping rules will be used.

7.5 CONTRACEPTION

Human data show that tacrolimus crosses the placenta. Nevertheless, limited data from organ transplant recipients show no evidence of an increased risk of adverse reactions on the course and outcome of pregnancy under tacrolimus treatment compared with other immunosuppressive medicinal products. However, cases of spontaneous abortion have been reported. Therefore, specific measures of contraception need to be taken in this clinical trial.

Women of childbearing potential (WOCBP) are generally excluded from participation in this clinical trial except if they fulfil one of the criteria mentioned in the exclusion criteria section (4.3 Exclusion criteria, p. 29). WOCBP can be included in this clinical trial if a highly effective contraceptive measure (Pearl Index < 1% per year) is used regularly and correctly. Such methods include combined (oestrogen and progestogen containing) hormonal contraception and progestogen-only hormonal contraception associated with inhibition of ovulation, intrauterine device (IUD), intrauterine hormone-releasing system (IUS), bilateral tubal occlusion, vasectomised partner, or sexual abstinence. For male participants with a pregnant or non-pregnant WOCBP partner contraception (condom) is recommended in order to avoid exposure of an existing embryo/foetus. Contraception should be continued until the end of the clinical trial (visit 7) in WOCBP.

7.6 DEALING WITH PREGNANCIES

Pregnant and breastfeeding women are generally excluded from participation in this clinical trial.

The occurrence of pregnancy during the trial itself (including partners of male study subjects who become pregnant) should not be treated as an adverse event, but must be reported by the trial site to the sponsor (or its designee(s)) immediately upon becoming known. The pregnancy must be monitored and documented throughout its course. The Sponsor provides a form for this purpose. The **trial therapy has to be terminated prematurely** (see 6.21).

Further treatment as well as follow-up observation must be carried out in consultation between the investigator and sponsor, if necessary with the involvement of the treating gynaecologist. The consent of the subject is required for the involvement of the gynaecologist. The investigator shall coordinate this.

Pregnancy complications and elective terminations for medical reasons must be reported as an AE or SAE. Spontaneous abortions must be reported as an SAE.

All study materials (completed, partially completed and empty CRFs) will be retained. Data will be collected to the point of withdrawal and used for the intention-to-treat analysis if subject consent. Further visits and trial specific procedures will be continued if subject consent: in the event of premature termination of the trial therapy, the documentation of all subsequent visits should still be pursued. There will be no replacement of withdrawn subjects. The subjects may request that from the time point of withdrawal no more data will be recorded and that all biological samples collected in the course of the study will be destroyed. The PIs will take care, that the patient will receive routine post-transplant care after withdrawal.

7.6.1 PREGNANCIAL COMPLICATIONS AND ABORTIONS

In contrast to the onset of pregnancy itself, serious complications occurring during pregnancy while taking an IMP as well as congenital anomalies or birth defects must be documented as serious adverse events (SAEs) and reported to the sponsor in accordance with the protocol.

Any SAE occurring in association with a pregnancy brought to the Investigator's attention after the subject has completed the study and considered by the Investigator as possibly related to the investigational product, must be promptly reported to the Investigator/sponsor including the first 12 months after birth.

The sponsor is responsible for fulfilling the legal reporting obligations if an AE with a suspected causal relationship to the investigational product occurs.

AEs include:

- premature termination of pregnancy, induced or spontaneous (note: termination of pregnancy for social reasons is not subject to reporting)
- complications during pregnancy
- an unhealthy or dead child and/or
- abnormal development of the child in the first 12 months after birth

If the evaluation shows that this is a suspected case of an unexpected serious adverse reaction (SUSAR), this case must be reported in accordance with the legal requirements.

The annual Development Safety Update Report should also report positive and negative experiences during pregnancy or lactation, if relevant.

7.6.2 STORAGE OF DOCUMENTATION

The original reports on the occurrence and outcome of pregnancy under exposure to an IMP are stored in the ISF and copies are stored in the TMF.

7.7 IN-VITRO DIAGNOSTIC MEDICAL DEVICE (IVD)

The used IVD TTV R-GENE® (bioMérieux) is CE certified and will be applied according to the intended use. The laboratories will measure the TTV-Level exactly as it is described in the corresponding data sheet. The manufacturer is responsible for the legally required market surveillance and the associated vigilance monitoring and reporting.

8 DOCUMENTATION AND DATA MANAGEMENT

8.1 DELEGATION LOG OF THE TRIAL SITE

The investigator is responsible for conducting the clinical trial in accordance with the GCP guidelines, the national and EU regulatories, and the trial protocol and that the data are properly documented. It must be ensured that all data relevant to the protocol are collected only by appropriately authorized persons. The authorization of these persons and their scope of authorization is carried out by the investigator at the trial site via an authorization list (Delegation Log/ Site Signature Log), which is stored as an original in the ISF and as a copy in the TMF. It must also be ensured that the signatures and abbreviations can be clearly assigned to the authorized persons.

8.2 SUBJECT IDENTIFICATION LOG

Subjects included in the trial are noted in a special subject identification list at the trial site. This list must guarantee a clear assignment of the subject to the subject trial number.

The subject identification list must be kept confidential and remains at the trial site after completion of the trial. In addition, the participation of the subject in this clinical trial must be recorded in the subject record (including IMP, subject number/randomization number, start and end of the trial, contact person within the trial site).

8.3 INVESTIGATOR SITE FILE (ISF)

An Investigator Site File (ISF) is provided to the trial site by the sponsor. All documents required for the clinical trial are kept at the ISF, in particular the essential documents according to ICH-GCP, such as the protocol, a sample of the subject informed consent form, the authorization of the clinical trial according to the Regulation (EU) No 536/2014, and other forms must be used. Changes or the use of own templates can be made in consultation with the sponsor.

The trial site is responsible for the actuality and completeness of the ISF. This will be checked during monitoring in accordance with the regulations.

After completion or discontinuation of the trial, the ISF must be retained for at least 25 years.

8.4 CASE REPORT FORM

Data relevant to the trial protocol is documented in the electronic Case Report Form (eCRF) specially created for this trial.

In addition to the investigator, only persons authorized by the investigator are granted access to the eCRF. The access data may not be passed on to third parties. The scope of authorization and the associated rights in the eCRF are controlled by data management via appropriately defined user roles.

The data relevant to the trial protocol (including the data of trial subjects who were prematurely excluded from the trial) must be documented in the eCRF pseudonymously, promptly, legibly (without the use of abbreviations), completely and in accordance with the source data.

Implausible values, which are displayed during data entry by programmed checks, must be checked by the trial site and corrected if necessary. If a correction is made in the eCRF, the reasons for it must be given. By means of the audit trail, all data and corrections are

automatically logged with date, time and the user name of the person entering the data. All old entries are retained and can be retrieved at any time.

A paper-based interim CRF is provided to the trial site as part of the ISF. This enables timely documentation in accordance with the protocol if the eCRF is not available (e.g. due to a system malfunction). The authorized persons will transfer the data immediately from the interim CRF to the eCRF as soon as the fault has been rectified.

The correctness and completeness of the documentation is confirmed by the authorized persons after each visit to the eCRF. Once the documentation for a subject has been completed, the principal investigator finally confirms the documentation for this subject in the eCRF.

Queries by the sponsor or its representatives must be checked by authorized persons using the source data and answered directly in the eCRF. Any resulting corrections must be made in the eCRF.

8.5 DATA MANAGEMENT

The data management plan describes the trial-specific approach of the individual processes for traceability and completeness of the relevant data.

The persons responsible for data management are responsible for data administration and processing. This is done by using an electronic data capture software for clinical trials that meets the requirements of the applicable laws and guidelines (especially GCP). The scope of database access and the associated authorizations are regulated by appropriate user roles.

The data is checked for completeness, plausibility and consistency by means of programmed checks directly in the eCRF and by additional manual checks outside the eCRF. Any queries arising in the process are sent to the respective trial site directly in the eCRF. Queries as well as responses or corrections are made directly in the eCRF. Changes to the data are reproduced in the audit trail.

The data is backed up on a daily basis. The data storage facilities are located in a locked room of the Medical Faculty of the TU Dresden, to which only the responsible system administrators have access.

At the end of the trial, the database is closed after all data relevant to the trial protocol has been entered and all queries have been clarified. Subsequent changes to the data can only be made with the consent of the principal coordinating investigator (PCI).

8.6 DATA STORAGE

8.6.1 STORAGE OBLIGATIONS OF THE TRIAL SITE

Records and documents related to the trial or distribution of IMPs (e.g., data collection forms, informed consent forms, drug accountability log, and other relevant documents) must be retained at the trial site in accordance with the regulatory requirement, but for at least 25 years.

Subject records and other original data must be kept for the longest possible period of time permitted by the hospital, institution or private practice, but at least 25 years.

8.6.2 STORAGE OBLIGATIONS OF THE SPONSOR

The sponsor is responsible for creating and maintaining a central trial master file (TMF) in accordance with ICH-GCP and for keeping it access-protected during the trial. It contains all

essential documents of the clinical trial. After termination or discontinuation of the trial, the TMF must be retained by the sponsor in accordance with the legal requirements, but at least for a period of 25 years.

9 MONITORING AND AUDIT

9.1 ACCESS TO SOURCE DATA

The investigator must allow all authorized third parties access to the trial site and to the subject's records (including direct access to source data and texts) in accordance with applicable legal requirements. Authorized third parties include monitors, auditors, and other persons authorized by the sponsor as well as the Member States concerned. All these persons are bound to secrecy.

9.2 MONITORING

The continuous monitoring of a clinical trial is an indispensable instrument of quality assurance and is risk-based according to ICH-GCP.

The monitoring includes initiation and monitoring visits for source data verification as well as final visits for the proper closure of the trial site. During the conduct of the trial, remote and central monitoring procedures are combined with on-site visits to achieve high protocol compliance and data quality, and to ensure subject's safety and rights.

Detailed information on the scope, procedure and contents of the monitoring as well as procedures to ensure data quality and necessary measures in case of protocol deviations are described in the monitoring plan.

Source data verification (SDV)

In addition to providing access to all documents related to the trial, including the original trial-relevant subject records, the trial site must maintain the subject record as complete as possible, i.e., record information on medical history, concomitant diseases, trial enrolment, visit dates, trial results, IMP dispensation, and (serious) adverse events.

The monitor is also enabled to perform data review and comparison with the relevant subject records in accordance with the Standard-Operation-Procedures (SOPs) and ICH-GCP guidelines at pre-determined intervals to ensure compliance with the protocol and continuous data recording. All original medical records that are required as the source of the information in the eCRF are reviewed. The trial subject has agreed to such verification by signing the trial-specific informed consent form.

Monitoring tasks are e.g.:

- verifying that the trial site meets the requirements of the clinical trial according to the trial protocol (subject population, equipment, etc.) and GCP guidelines,
- initiation of the trial site,
- verification of informed consent forms,
- checking the ISF for completeness and actuality,
- source data verification,
- verification of proper reporting of SAEs,
- verification of compliance.

The monitor is obliged to treat all information confidentially and to uphold the fundamental right of the subjects to integrity and protection of their privacy.

9.3 AUDIT/INSPECTION

In order to guarantee that the trial is conducted in accordance with GCP guidelines, audits can be carried out on behalf of the sponsor. The auditor is independent of the persons involved in the trial.

During the audit, the following points are checked, among others:

- quality of the trial according to GCP guidelines,
- performance of the trial according to the trial protocol,
- validity of the data.

After each audit, the trial site receives an audit confirmation from the person responsible for the audit. This confirmation must be kept in the ISF so that it is available in case of an inspection by the Member States concerned. A corresponding audit report is also sent to the trial site and the Sponsor.

In addition, inspections can be carried out by the responsible Member State concerned in accordance with the Regulation (EU) No 536/2014. In case of an announcement of an inspection, the trial site must inform the sponsor immediately.

10 STATISTICS

A general description of the sample size calculation and the statistical methods to be used to analyze this study are outlined below. More details will be provided in the statistical analysis plan (SAP).

10.1 STATISTICAL HYPOTHESES

Study objective

The main goal of this study is to demonstrate that the TTV-guided immunosuppression (arm T) is non-inferior with respect to safety compared to standard dosing (arm S) in stable adult kidney transplant recipients with low immunological risk in the first year after transplantation.

Non-Inferiority hypothesis:

The primary objective is to demonstrate the non-inferiority of TTV-guided immunosuppression (arm T) compared to standard dosing (arm S) in stable adult kidney transplant recipients with low immunological risk in the first year after transplantation.

Non-inferiority can be concluded if the upper limit of a two-sided 95% CI for the difference in proportion of patients at month 9 after randomisation (= Visit 7; post-transplant month 12) between the two treatment arms is less than 10% points.

Let p_T and p_S be the proportion (in %) of participants in the arm T and arm S, respectively, then the primary statistical hypothesis can be formulated as follows:

$$H_0\text{:}\ p_T\text{-}p_S \geq 10\ \% \quad \text{versus} \quad H_1\text{:}\ p_T\text{-}p_S < 10\ \%$$

Superiority hypothesis:

If non-inferiority is reached, the study will demonstrate superiority of TTV-guided immunosuppression (arm T) compared to standard dosing (arm S) in the same endpoint by testing the hypothesis:

$$H_0\text{:}\ p_T\text{-}p_S \geq 0 \quad \text{versus} \quad H_1\text{:}\ p_T\text{-}p_S < 0$$

Null hypothesis

TTV-guided immunosuppression is not safe compared to standard dosing in stable adult kidney transplant recipients with low immunological risk in the first year after transplantation.

10.2 SAMPLE SIZE CALCULATION

For the sample size calculation, we analysed the occurrence of the primary endpoint (infection, allograft rejection, death and graft loss) in recipients of a kidney allograft transplanted between 1.1.2012 and 31.12.2018 at the Vienna centre, applying the trial-specific inclusion and exclusion criteria (unpublished data). Overall, 40% of patients experienced the primary endpoint between month 3 and 12 after transplantation. Therefore, we assume a composite rate of around 40% in the standard dosing (arm S).

It is expected that the TTV-guided immunosuppression (arm T) reduces the occurrence of the primary endpoint, and therefore rates between 20% and 30% are assumed to be reasonable.

The non-inferiority margin has been fixed 10% points (see also above).Thus when the sample size in each group is 120, a two-group large-sample normal approximation test of proportions with a one-sided 2.5% significance level will have 90.42% power to reject the null hypothesis

that arm T is inferior to standard arm S (the difference in proportions, π_1 - π_0, is 0.1 or farther from zero in the same direction) in favour of the alternative hypothesis arm T is not inferior to arm S, assuming that the expected proportions are 0.3 and 0.4 in arm T and S, respectively (i.e. assuming a better outcome in arm T).

Due to the planned observation period of 9 months and the special patient population, a rather low drop-out rate is expected of about 2-5%. Adjusting for potential drop-outs, the sample size is fixed with 130 patients per group, i.e., 260 in total.

The sample size calculation was performed using N-Query Version 8.6.1.

10.3 STATISTICAL ANALYSIS

10.3.1 DEFINITION OF EVALUTION POPULATIONS

The primary analysis will be performed on a modified intention to treat (mITT) principle. Sensitivity analyses will be performed according to intention to treat and per protocol (PP) analysis, and the dataset restricted to adherent patients. Further sensitivity analyses are described below.

Different analysis sets are defined as follow:

mITT set

This analysis set includes subjects who were randomized. According to the intent to treat principle, subjects will be analyzed according to the treatment they have been assigned to during the randomization procedure. The miTT includes all patients who are eligible for the study and with at least one TAC evaluation (and potential adaption) during visit 1 will be included in the analysis.

PP set

This analysis set comprises all subjects who received study intervention and did not critically or majorly violate the protocol in a way that might affect the evaluation of the effect on the primary objective. A list of potential protocol deviations is given in section 6.14.

Adherent set
Medical adherence will be defined by patient diary; see 5.10

10.3.2 BASELINE PARAMETERS AND CONCOMITANT MEDICATIONS

Baseline parameters, medical history and concomitant medication will be documented during screening and throughout the trial until the last follow-up visit. Enrolment, protocol deviations and discontinuations from the study drug and the study will be summarized. Demographics (e.g. age, race, ethnicity and sex) and medical history and concomitant medication will also be summarized by treatment group. For qualitative variables (e.g. sex), absolute (n=x) and relative frequencies will be calculated per treatment group. Data will be visualized by bar plots. For quantitative data (e.g. age), the number of valid observations (n=x), mean, standard deviation, standard error, median, minimum and maximum will be calculated for each treatment group and each time point separately. Data will be visualized by spaghetti plots (showing individual patient profiles over time), boxplots and histograms.

10.3.3 STATISTICAL ANALYSIS OF THE PRIMARY END POINT

The occurrence of the primary composite endpoint will be presented per treatment group as absolute numbers and percentages. The difference of the occurrence of the primary endpoint between the two treatment groups and a two-sided 95% confidence interval will be calculated.

Non-inferiority will be concluded if the upper limit of a two-sided 95% confidence interval for the difference in proportion of patients at month 9 after randomization (= Visit 7; post-transplant month 12) between the two treatment arms is less than 10% points. Superiority will be concluded if the confidence interval excludes 0.

As additional sensitivity analyses confidence intervals will be calculated being adjusted for study stratifications (such as centers) using Cochrane-Mantel-Haenszel weights. Also logistic regression models will be used using additional factors (such as sex) and covariate (such as age at randomization).

To explore the composite endpoint further, it will be also be analyzed as time-to-event endpoint considering the time from randomization till time of first event. We will visualize these data by presenting Kaplan-Meier curves for each treatment arm. Furthermore, Cox-regressions models will be performed adjusting for the same factors and covariates as used in the logistic regression models.

To address the repetitive nature of the events used in the definition of the primary endpoint, we will also perform supportive analyses for recurrent event data. We will fit a negative Binomial regression model for the events of the composite endpoints accounting for the time a patient is under risk. Furthermore, we will perform the counting process model of Andersen-Gill and frailty models.

To assess the impact of the individual components of the composite endpoints, each component will be analyzed descriptively (see description for secondary endpoints).

10.3.4 STATISTICAL ANALYSIS OF THE SECONDARY END POINTS

For **binary secondary endpoints** (such as rejection) absolute (n=x) and frequencies in percentage (%) will be calculated per treatment group. 95%-confidence intervals will be calculated for rates, if appropriate. Such data will be visualized with bar charts. If appropriate, logistic regression models will be applied using treatment as independent factor. Furthermore, the model will be adjusted for sex and age.

For **time-to-event endpoints** (such as overall survival) Kaplan-Meier plots will be provided. The two-group will be compared with log-rank tests. Additionally Cox-Regressions models will be performed adjusting for additional factors as described for the primary analysis.

For recurrent events (such as infections) the appropriate survival methods will be applied. This includes the Negative Binomial Regression models, Anderson and Gill models and Frailty models. Hazard ratios and corresponding two-sided 95% confidence intervals will be reported.

Continuous secondary endpoints such as laboratory values or “SF36” will be summarized by mean, standard deviation, median, first and third quartiles, minimum and maximum for each treatment arm separately. If a continuous endpoint is measured only at one time point after randomization, it will be analysed using an analysis of covariance using the factor treatment group adjusting for the factors sex and the covariate age (in years). Mean estimates will be provided, together with their corresponding two-sided 95% confidence intervals. If repeated measurements are available for several visits after randomisation, a mixed model for repeated measurements will be performed using patient as random factor and treatment arm as fixed

factor. If baseline values are collected, they will be included as covariate in the analysis of covariance and mixed model for repeated measurements.

10.3.5 MULTIPLE TESTING

For the primary endpoint we will use a hierarchical testing procedure. This means first we will test the non-inferiority hypothesis at a one-sided alpha of 2.5%. After non-inferiority can be demonstrated, superiority will be tested also using a one-sided alpha of 2.5%.

The secondary endpoints include the components of the primary outcome and are needed to support the interpretation of the potential effects in the primary endpoint. The tests for this comparisons would require larger sample sizes to achieve the required power. This was the reason why a composite endpoint has been chosen and these analyses are considered supportive only without further multiplicity correction.

For the analyses of all secondary endpoints two-sided p-values and two-sided 95%-confidence intervals will be reported.

10.3.6 INTERIM ANALYSIS

There will be no interim analysis for efficacy. The DSMB will monitor the trial for safety purposes.

10.3.7 MISSING VALUES

For the primary analysis missing values will be considered as treatment failures in the composite primary endpoint. Additionally meaningful missing values will be imputed by statistical models for the data according to underlining mechanism of missing data. Sensitivity analyses will be performed for imputed data.

10.3.8 EXPLORATORY SUBGROUP ANALYSIS

Subgroup analysis will be performed in patients at risk for immunological (re-transplantation) and infectious (diabetes mellitus) events and according to age group (>55 years of age), gender and study centre. If appropriate this factors will be included as additional covariates in the regression models as described above.

10.3.9 STATISTICAL SOFTWARE

All statistical analyses will be conducted with statistical software like SAS 9.4. (or higher), R 3.6.3. (or higher) or IBM SPSS Statistics (SPSS Inc., Chicago, IL, USA), STATA (StataCorp, College Station, TX, USA), MS EXCEL (Microsoft, USA). Any deviation from the original statistical plan will be described and justified in the protocol and in the final report.

11 ETHICAL, LEGAL AND ADMINISTRATIVE ASPECTS

11.1 RESPONSIBILITIES OF SPONSOR AND INVESTIGATOR

The sponsor of the clinical trial (Medical University of Vienna) assumes responsibility for the initiation, organization and funding of the clinical trial to be conducted in accordance to national laws. To this end, the clinical trial must be registered in the EudraCT database before the application for approval by the Member states concerned.

The sponsor and the investigator ensure that the clinical trial is conducted in accordance with existing laws and regulations, in accordance with the ICH-GCP guidelines, the Declaration of Helsinki (2013), the Regulation (EU) No 536/2014 as well as the provisions of the national laws.

The investigator accepts the requirements of the signed trial protocol.

His or her responsibilities include:

- understanding the characteristics of the IMP described in the Investigator's Brochure or the Summary of Product Characteristics,
- understanding and implementing the treatment plan,
- ensure that sufficient time and capacity are available to conduct the trial,
- correct collection and documentation of data, reporting,
- provision of all data for sponsor, monitoring or corresponding authorities for audits and/ or inspections,
- ensure that the information on trial subjects and all information received from the sponsor is kept confidential by all persons involved in the trial,
- statement on the involvement of persons possibly dependent on the sponsor or the investigator,
- information on possible economic and other interests of the investigators in connection with the IMP.

According to EU regulation No 536/2014, the respective investigator assumes responsibility for conducting the clinical trial at the trial site.

We confirm that the proposed trial will comply with established ethical principles, EU regulation No 536/2014 and applicable national laws. We will ensure respect for people and for human dignity and fair distribution of research benefits, and will protect the values, rights and interests of our research participants.

It is important to note that the proposed research does not involve:

- research activity aimed at human cloning for reproductive purposes;
- research activity intended to modify the genetic heritage of human beings, which could make such changes heritable;
- research activity intended to create human embryos solely for the purpose of research or for the purpose of stem cell procurement, including by means of somatic cell nuclear transfer;
- research involving the use of human embryos or embryonic stem cells;
- processing of genetic information;
- animals;
- non-EU countries;

- components that will raise any environmental and health and safety concerns.

11.2 APPROVING EVALUATION OF THE ETHICS COMMISSION AND NOTIFICATION TO THE AUTHORITIES

The clinical trial may only be started if the approving evaluation of the ethics committee responsible for the principal coordinating investigator (PCI) and the approval of the responsible higher federal authorities have been received.

Prior to the start of the trial, notification of the start of the clinical trial is made to the responsible higher federal authorities in accordance with EU regulation No 536/2014 and national laws.

11.3 SUBJECT INSURANCE

On behalf of the Sponsor, the mandatory subject insurance according to national and EU regulations for all trial subjects was concluded with the following insurer:

Table 2: Details of the country-specific subject insurance

	Austria	Czech Republic	France	Germany	Netherlands	Spain
Company	Zürich Versicherungs-Aktiengesellschaft	HDI Versicherung AG, organizační složka	HDI GLOBAL SE, Direction pour la France	HDI Global SE, Zentrale - Standort Hannover	HDI Global SE, the Netherlands	HDI Global SE<br>Sucursal en España
Policy No.	07229622-2	2102121	01005345140 58 210129	76458782 03035 390	V-074-969-270-5	76494709-30014
Address	Schwarzenberg-platz 15, 1010 Wien	Jugoslávská 620/29, 120 00 Praha 2	Tour Opus 12 -Défense 9<br>77 Esplanade du Général de Gaulle, 92914 Paris	HDI-Platz 1, 30659 Hannover	Postbus 925, 3000 AX Rotterdam	C/ Luchana, 23, 5ª Planta<br>28010 Madrid

As soon as you experience:

- a claim,

- a writ of summons

For countries except Austria please contact the following without delay:

Mr Yves GARCIN
Phone: 033.1.58.86.82.35
Fax: 033.2.98.76.46.21
Email: yves.garcin@verlingue.fr with a copy

to maryvonne.sevestre@biomedic-insure.com

For Austria please contact:

Zürich Versicherungs-Aktiengesellschaft,
Leopold-Ungar-Platz 2
1190 Wien
Tel.: 0043 (01) 50125-0

This insurance covers all possible damages that the subject suffers directly or indirectly as a result of the IMP or interventions in connection with the clinical trial.

In order not to jeopardize the insurance coverage, the trial subjects must strictly follow the instructions of the trial site personnel. Furthermore, they may not undergo any other medical treatment during the trial without the investigator's consent (except in emergencies). They must immediately inform the investigator of any emergency treatment. Any damage to health that may have occurred as a result of the trial must be reported immediately by the subjects to the investigator and the insurance company. In addition, the subjects must take all appropriate measures to clarify the cause and extent of the damage.

The subject receives assurances that appropriate insurance coverage is in place or the insurance conditions is handed out together with his or her copy of the informed consent form.

11.4 DATA PROTECTION AND CONFIDENTIALITY

The collection, transfer, storage and evaluation of personal data within this clinical trial is carried out in accordance with the applicable legal regulations (e.g. EU General Data Protection Regulation (EU reg. 2016/679 GDPR)). The prerequisite for this is the voluntary consent of the subjects in the informed consent form prior to participation in the clinical trial. During the informed consent procedure, the subjects are informed about the following:

1. data collected in the course of this clinical trial will be recorded on electronic data carriers, treated in strict confidence and only passed on to:
   a) the sponsor of the trial (MUV) for scientific evaluation and assessment of adverse events
   b) the responsible supervisory authorities (the regional councils or the higher federal authorities), the ethics commissions and the European database to verify the proper conduct of the trial and to evaluate trial results and adverse events.
2. To the extent necessary for the review of the clinical trial, authorized representatives of the sponsor (monitoring, auditing) and/ or the regulatory authority, who are bound to secrecy, may inspect the personal data available at the trial site. For this measure, the investigator is released from medical confidentiality.
3. Consent to the collection and processing of personal data within the scope of this clinical trial is revocable. The subject is informed that he/ she may terminate his/ her participation in the clinical trial at any time - without giving reasons and without any consecutive disadvantages. Just like the consent to participate in the clinical trial, the consent to process the collected data can be revoked at any time. According to national and EU regulations, however, in the event of revocation, the stored data may be used further to the extent necessary to
   a) to determine the effects of the medicinal product under investigation
   b) to ensure that the interests of the participants worthy of protection are not impaired,
   c) to comply with the obligation to submit complete registration documents.
   In the event of revocation of the declaration of consent, the responsible bodies will immediately check to what extent the stored data is still required. Data that is no longer required will be deleted immediately, unless legal and/or official documentation and reporting obligations conflict with this. However, the data processing carried out up to the point of revocation remains lawful.

11.5 ETHICS AND GOVERNANCE COUNCIL

The management of this trial will be assisted and advised by an independent Ethics and Governance Council (EGC). The role of the EGC is to provide independent external supervision

and advice regarding the ethical and legal aspects of the clinical trial. The EGC will monitor the procedures in place for the clinical trial to ensure the application of the highest ethical standards, thus safeguarding patients' interests and rights. The EGC will convene regularly as an independent forum and will also be invited to take part in TSC meetings and will receive the progress and ethics reports. Questions with ethical relevance concerning the trial will also be communicated in a suitable and understandable manner for the general public on the project website, helping to inform the public debate on the new technology.

The central objectives are:

- To ensure that the study protocol and measures are in agreement with ICH-GCP and the Declaration of Helsinki.

- To ensure continuous ethical scrutiny of activities.

- To provide advice for patient consenting and inclusion regarding the randomised approach.

- To ensure – via the external EGC – independent external supervision and advice regarding the ethical, legal and safety aspects.

- To monitor the procedures in place for the study to ensure safeguard of patients' interests and entitlements.

- To review data safety measures and ensure compliance with the current EU data protection legislation.

- To provide a framework that enables trial centres to address ad hoc ethical and legal issues competently and swiftly

12 AMENDMENTS TO THE TRIAL PROTOCOL

In order to ensure largely comparable conditions in all trial sites and in the interest of proper data evaluation, there are no plans to change the test conditions agreed upon and laid down in the trial protocol.

In exceptional cases, however, the examination conditions may be changed. These shall only be made after mutual agreement between the investigator and the sponsor. Any change to the procedure laid down in the trial protocol must be made in writing, stating the reasons for the change, and signed by all persons responsible for the trial. The changes are then considered to be part of the protocol.

Where necessary (e.g., in the case of a change in the IMP dosing scheme or other significant changes that indicate a direct impact on the safety of the trial subjects), the consent of the ethics committees and/ or regulatory authorities responsible as well as the investigator to the protocol amendments shall be obtained, and the amendment shall be submitted to the regulatory authority.

13 PUBLICATION

13.1 FINAL REPORT

After the end of the clinical trial, the statistical evaluation is performed first and then the results report is prepared, which contains all essential results of the clinical trial. This report is submitted to the responsible higher federal authority and the ethics committee within one year after the end of the clinical trial. In addition, the results and, if necessary, other necessary information will be published in the European database.

13.2 PUBLICATIONS

Links to publications will be provided to the trial registry. The trial results will be reported on the trial webpage and to the IRBs, the funding agency (EU; the study is 100% funded by a H2020 RIA action, project number: 896932, project name: TTV GUIDE TX, project coordinator: Dr. Gregor Bond) and the consortium partners of the H2020 project. The full trial database and analysis dataset will be reported to the competent regulatory authorities and as far as possible made available to the H2020 Open Data Pilot. All results will be published open access. Wherever possible, the "gold" open access route will be preferred. If the gold route is not feasible, the green route with self-archiving will be selected.

13.3 DATA SHARING

After the results of the main objective of the clinical trial have been published, previously published primary data can be passed on to interested scientists in anonymized form on request, provided that the principal coordinating investigator (PCI), sponsor and trial subject agree to the transfer.

14 SIGNATURES

The following responsible persons, according to the sponsor's delegation, agree to the content of the clinical trial and confirm this with their signatures.

Sponsor

Prof. Dr. Gregor Bond		
Name in block letters	Place, Date	Signature

Signiert von: Gregor Bond

Datum: 15.07.2022 17:52:50

Dieses mit einer qualifizierten elektronischen Signatur versehene Dokument hat gemäß Art. 25 Abs. 2 der Verordnung (EU) Nr 910/2014 vom 23. Juli 2014 ("eIDAS-VO") die gleiche Rechtswirkung wie ein handschriftlich unterschriebenes Dokument.

www.a-trust.at

Dieses Dokument ist digital signiert!

Prüfinformation:
Informationen zur Prüfung der elektronischen Signatur finden Sie unter:
www.handy-signatur.at

TRUST einfach sicher

Biostatistics

Prof. Dr. Franz König		
Name in block letters	Place, Date	Signature

14 SIGNATURES

The following responsible persons, according to the sponsor's delegation, agree to the content of the clinical trial and confirm this with their signatures.

Sponsor

Prof. Dr. Gregor Bond		
Name in block letters	Place, Date	Signature

Biostatistics

Prof. Dr. Franz König	Wien, 15.7.2022	[signature]
Name in block letters	Place, Date	Signature

Investigator at the trial site:

I hereby confirm that I have read and understood the present trial protocol and accept it in all parts.

I undertake to ensure that the persons included in the clinical trial at my site are treated, observed and documented in accordance with the provisions of this trial protocol.

Name in block letters	Place, Date	Signature

15 LIST OF ABBREVIATIONS

**ABMR**	Antibody mediated rejection
**AE**	Adverse event
**AR**	Adverse reaction
**b. d.**	Twice a day
**BKV**	BK virus
**BL TCMR**	Borderline rejection suspicious for T-cellular mediated rejection
**CBC**	Complete blood count
**CI**	Confidents interval
**CIT**	Cold ischemia time
**CKD EPI**	Chronic Kidney Disease Epidemiology Collaboration
**CMV**	Cytomegalovirus
**CRF**	Case report form
**CRP**	C-reactive protein
**CT**	Computer tomography
**CTCAE**	Common terminology criteria for adverse events
**DSA**	Donor-specific antibodies
**eCRF**	Electronic case report form
**eGFR**	Estimated glomerular filtration rate
**ESRD**	End stage renal disease
**EudraCT**	European Union Drug Regulating Authorities Clinical Trials
**FAS**	Full analysis set
**FPFV**	First patient first visit
**GCP**	Good clinical cractice
**HLA**	Human leucocyte antigen
**IHC**	Imunhistochemistry
**ICH**	International Conference on Harmonization
**ICMJE**	International Committee of Medical Journal Editors
**IL**	Interleukin
**IQR**	Interquartile range
**ISF**	Investigator site file
**ITT**	Intention to treat
**IVIG**	Intra venous immunoglobulin G
**KKS**	Koordinierungszentrum für Klinische Studien
**LPLV**	Last patient last visit
**MDRD**	Modification of Diet in Renal Disease
**MMDX**	Molecular microscope
**MUV**	Medical University of Vienna
**MRI**	Magnetic resonance Imaging

**NA**	Not applicable
**ND**	Not done
**NF-AAT**	Nuclear factor activated T-cells
**NPV**	Negative predictive value
**NODAT**	New onset diabetes after transplantation
**o.d.**	Once a day
**OR**	Odds ratio
**PCT**	Procalcitone
**PEI**	Paul-Ehrlich-Institut
**PET**	Positron emission tomography
**pH**	Pondus hydrogenii
**PPS**	Per protocol set
**PPV**	Positive predictive value
**PVAN**	Presumptive polyomavirus-associated nephropathy
**rt-PCR**	Real-time polymerase chain reaction
**SAB MFI**	Single antigen bead median fluorescence intensity
**SAE**	Serious adverse event
**SAFB**	Single antigen fluorescence beads
**SAR**	Serious adverse reaction
**SAS**	Safety analysis set
**SDV**	Source data verification
**S/AFOG**	Acid Fuchsin Orange G
**SF-36**	Medical Outcomes study Short Form 36
**SOP**	Standard operating procedure
**SOFA**	Sequential organ failure assessment
**SUSAR**	Suspected unexpected serious adverse reaction
**TAC**	Tacrolimus
**TMA**	Thrombotic micro angiopathy
**TMF**	Trial master file
**TTV**	Torque Teno virus
**TX**	Transplantation
**UAR**	Unexpected adverse reaction
**US**	Ultra sound
**vBGA**	Venous blood gas analysis
**WOCBP**	Women of childbearing potential
**ZN stain**	Ziehl-Neelsen stain

16 LIST OF LITERATURE

- De Vlaminck I, Khush KK, Strehl C et al. Temporal response of the human virome to immunosuppression and antiviral therapy. Cell. 2013.

- Doberer K, Haupenthal F, Nackenhorst M et al. Torque Teno virus load is associated with sub-clinical alloreactivity in kidney transplant recipients - a prospective observational trial. Transplantation. 2021.

- Doberer K, Schiemann M, Strassl R et al. Torque teno virus for risk stratification of graft rejection and infection in kidney transplant recipients - a prospective observational trial. American Journal of Transplantation. 2020.

- Fernandez-Ruiz M, Albert E, Gimenez E, Ruiz-Merlo T et al. Monitoring of alphatorquevirus DNA levels for the prediction of immunosuppression-related complications after kidney transplantation. American Journal of Transplantation. 2019.

- Focosi D, Antonelli G, Pistello M et al. Torquetenovirus: the human virome from bench to bedside. Clinical microbiology and infection. 2016.

- Ahlenstiel-Grunow T, Liu X, Schild R et al. Steering Transplant Immunosuppression by Measuring Virus-Specific T Cell Levels: The Randomized, Controlled IVIST Trial. JASN. 2021.

- Kulifaj D, Durgueil-Lariviere B, Meynier F et al. Development of a standardized real time PCR for Torque teno viruses (TTV) viral load detection and quantification: A new tool for immune monitoring. Journal of clinical virology. 2018.

- Maggi F, Pifferi M, Fornai C et al. TT virus in the nasal secretions of children with acute respiratory diseases: relations to viremia and disease severity. Journal of clinical virology. 2003.

- Mcculloch E, Montgomery D, Maggi F et al. External Quality Assessment (EQA) Pilot Study for Molecular Diagnostics of Torque Teno Virus (TTV). 22nd Annual Meeting of the European Society for Clinical Virology, Copenhagen. 2019.

- Mian M, Natori Y, Ferreira V et al. Evaluation of a Novel Global Immunity Assay to Predict Infection in Organ Transplant Recipients. Clin Infect Dis. 2018.

- Ravaioli M, Neri F, Lazzarotto T et al. Immunosuppression Modifications Based on an Immune Response Assay: Results of a Randomized, Controlled Trial. Transplantation. 2015.

- Solis M, Velay A, Gantner P et al. Torquetenovirus viremia for early prediction of graft rejection after kidney transplantation. The Journal of infection. 2019.

- Strassl R, Doberer K, Rasoul-Rockenschaub S et al. Torque Teno Virus for Risk Stratification of Acute Biopsy-proven Alloreactivity in Kidney Transplant Recipients. The Journal of infectious diseases. 2019.

- Strassl R, Schiemann M, Doberer K et al. Quantification of Torque Teno Virus Viremia as a Prospective Biomarker for Infectious Disease in Kidney Allograft Recipients. The Journal of infectious diseases. 2018.
