# Supplementary material for: A multicentre, patient- and assessor-blinded, non-inferiority, randomised and controlled phase II trial to compare standard and torque teno virus-guided immunosuppression in kidney transplant recipients in the first year after transplantation: TTVguideIT
Source: Trials. 2023 Mar 22;24:213. doi: 10.1186/s13063-023-07216-0 (PMC10032258; doi:10.1186/s13063-023-07216-0)
Supplement: Supplementary file 2 — Additional file 2. [file 13063_2023_7216_MOESM2_ESM.pdf]

DATA MANAGEMENT PLAN

Project deliverable number:	D4.3
Work Package:	WP4
Lead Beneficiary:	TUD
Dissemination level:	CO
Official delivery date:	M6
Status:	FINAL

TTV GUIDE TX Coordinator
Medical University Vienna
Spitalgasse 23, 1090 Vienna, Austria
coordinator@ttv-guide.eu

This project has received funding from the European Union's Horizon 2020 research and innovation programme under grant agreement no. ID 896932

The content of this report reflects only the authors' view. The Research Executive Agency (REA) is not responsible for any use that may be made of the information it contains.

Project[1] Number: [896932]

Project Acronym: [TTV GUIDE TX]

Project title: [PERSONALISATION OF IMMUNOSUPPRESSION BY MONITORING VIRAL LOAD POST KIDNEY TRANSPLANTATION - A RANDOMISED CONTROLLED PHASE II TRIAL]

DATA MANAGEMENT PLAN

Version 1.0, 26.10.2021

[1] The term 'project' used in this template equates to an 'action' in certain other Horizon 2020 documentation

1. Data Summary

Purpose of the data collection/generation:

See Chapter 1.2 of the protocol:

"The specific aim of the proposal is to demonstrate the safety and tolerability of TTV-guided immunosuppression in kidney transplant recipients. For the first time, a holistic personalised and functional assessment of the immune system will be tested in a randomised and controlled clinical setting.

Kidney transplantation is the gold standard of treatment for patients with End Stage Renal Disease (ESRD). After transplantation, immunosuppressive drugs are crucial for reducing the risk of organ rejection. Despite this desired effect, the compromised immunity of the recipient leads to an increased risk for infectious disease. Moreover, current immunosuppression regimens are unable to sufficiently control allo-recognition of the graft, which leads to chronic rejection. Thus, optimal management of immunosuppressive drug dosing requires a delicate balance between inadequate and excessive immunosuppression. At present, there is no diagnostic test or algorithm for optimal guidance of immunosuppressive drugs in clinical routine.

The proposed clinical trial will assess the potential of a novel healthcare intervention that would generate meaningful advances in clinical practice and care for patients with ESRD. Notably, the trial would enable clinicians to personalise, optimise immunosuppressive drug dosing, thus reduce infections, and graft rejection in kidney transplant recipients. Both outcomes are burdensome for kidney transplant patients and highly clinically relevant. Infections are the second-most common cause of death following kidney transplantation and rejection is the leading cause of graft loss."

Disclaimer: The process described below relates to the management of the clinical data by the KKS Dresden in the context of the clinical trial, including the preparation and publication of the results report and archiving in accordance with the requirements of EU Regulation 536/2014.

Types and formats of data: See annotated CRF and system reports on Data Base structure in current version. KKS Data Management provides these documents.

Re-use of existing data: KKS Dresden does not use data from previous projects for this project, nor does it re-use data from this project.

Origin of the data: Source data are the Patient files; lab results or study files.

Expected size of the data: A statement about the file size can only be made for the final export.

Data utility:

See chapter 1.31. of the protocol:

"Benefit of trial therapy"

We and others have demonstrated the predictive value of TTV copy number for infectious disease and organ rejection in kidney allograft recipients. Within the proposed trial, we anticipate a reduction from infection and rejection from 40% to 30%.

The results of the proposed trial have the potential to change clinical practice of immunosuppression dosage after kidney transplantation, thereby reducing infectious and immunologic complications. Effective project management, rigorous quality assurance, control, and detailed risk assessment will increase the likelihood of a successful conduction of the trial and scientific integrity of the data.

The anticipated 25% reduction in rejection and infection rates by TTV-guided immunosuppression would prevent 5k infections, 150 deaths due to infection, 400 rejections and 300 graft losses in Europe per year. Lower infection and rejection rates achieved through optimisation of immunosuppression would result in reduced hospitalisation and harm due to the side effects of antimicrobial and anti-rejection therapy. The prolongation of graft survival reduces the need for dialysis, which is a very burdensome procedure for ESRD patients, and thus improves quality of life. Improved graft survival would also reduce the number of patients re-entering the waiting list for a kidney transplant after terminal graft failure, thereby shortening the waiting-time for ESRD patients on the waiting list for a suitable kidney. These changes would result in significant improvement in health-related quality of life for ESRD patients.

The results of the trial have the potential to contribute to increase patient and graft survival and thus to reduce the socioeconomic burden of ESRD for healthcare systems.

Taken together, non-interventional data support the hypothesis of the trial and equipoise for the suggested intervention is given, the potential benefits outweigh the potential risks for the participants and the trial has the potential to improve clinical practice."

2. FAIR data

2. 1. Making data findable, including provisions for metadata

Use of unique identifiers: Yes.

Naming conventions: The creation of the database is based on CDISC (Clinical Data Interchange Standards Consortium), unless the client has its own standards/guidelines.

Provision of keywords to optimize possibilities for re-use: By following CDISC and the associated standardization, further use is supported. However, there is no indexing in the naming of the data fields in the database.

Provision of clear version numbers: Yes.

Type of metadata:

Following .txt files will be created for archiving:

- SAS_Data.txt: Contains the data (separator tab stop).
- SAS_Label.txt: Contains the coding and the corresponding name of the questions.
  - SAS_Category.txt: Contains the coding of the categories and the designation to the respective coding.
  - SAS_QLU.txt: Contains the formats of all fields, e.g. Text, Category, Date/Time.
- SAS_Format.txt: Specifies the format of Date/Time-questions, e.g. ddmmyy or time.
  - SAS_Type.txt: Specifies the format of all fields, e.g. $50 means text field with max. 50 characters. Here also the specifications for the formats of the Date/Time-Questions appear redundantly.

Other possible export formats: .xml and .csv

An annotated CRF and system reports (containing further information on the database structure) are also provided.

2.2. Making data openly accessible

In the trial, personal data are collected and stored in the database in a pseudonymized form. The evaluation is carried out with pseudonymized data. For legal reasons, the data are stored by the sponsor for 25 years. Results of the clinical trial will be published

The results are published as required by EU regulation 536/2014. In addition, publication in scientific journals is planned.

For data protection reasons, only anonymized data can be provided for general publication. This means that the data must be processed in such a way that it is no longer possible to draw conclusions about the patients.

2.3. Making data interoperable

To be described later.

2.4. Increase data re-use (through clarifying licences)

At the earliest after completion and publication of the final trial report, which by law must be published within one year after the end of the study (last visit of the last patient, LPLV).

Restriction of data re-use: Data that may lead to identification of the patient shall not be further used.

Duration of the reusability of the data: 25 years after end of trial.

Data quality assurance processes:

Data quality is monitored in accordance with the SOPs applicable to the KKS Dresden. A monitoring manual is prepared for the monitoring activities. The implementation of data verification is described in the KKS internal Data Management Plan Module 4.

3. Allocation of resources

In the course of the study the KKS Dresden as representative of the Sponsor is responsible for data management.

4. Data security

At KKS Dresden, the archive files are stored in a data management system, in a separate partition with exclusive read authorization (no electronic archive). The TMF is transferred to the sponsor at the end of the project by the KKS (after uploading the final trial report). In the TMF, the data are stored on CD for archiving at the sponsor. The sponsor will ensure the readability of the data for the duration of the archiving.

5. Ethical aspects

In the study, personal data are collected and stored in the database in a pseudonymized form. The evaluation is carried out with pseudonymized data. For legal reasons, the data are stored by the sponsor for 25 years.

For data protection reasons, only anonymized data can be provided for general publication. This means that the data must be processed in such a way that it is no longer possible to draw conclusions about the patients.

Participation in the clinical trial is only possible with the patient's consent to the collection and processing of the data. Patients will be informed about the collection and processing of personal data in the patient information (informed consent form) and verbally by the investigator.

Ethics committees review patient information as part of the clinical trial approval process with regard to the requirements of EU Regulation 536/2014. The trial sites may only start the clinical trial after the ethics committees have given their approval.

Patients are informed, give their consent that the data will be stored in pseudonymized form for 25 years, and then in anonymized form, and that publication of the data will only take place in a way that does not allow conclusions to be made about the person (anonymized).

6. Other issues

Data management in the clinical trial is performed in accordance with the following regulations:

EU regulation 536/2014; EU regulation (EU) 2016/679; National laws on clinical trials (e.g. AMG); Grand Agreement of the project; , ICH-GCP E6 (R2), EMA/INS/GCP/454280/2010, FDA CFR Part 11 and the SOPs of the Lead CTU (KKS Dresden).

SUMMARY TABLE 1

FAIR Data Management at a glance: issues to cover in your Horizon 2020 DMP

This table provides a summary of the Data Management Plan (DMP) issues to be addressed, as outlined above.

DMP component	Issues to be addressed
**1. Data summary**	• Purpose of the data collection/generation: basis for statistical evaluation, overview patient safety and trial procedure<br>• Types and formats of data generated/collected: annotated case report form (CRF), system reports on database structure<br>• no existing data will be reused<br>• Origin of the data: patient records (including lab results), study files<br>• Expected size of the data: not known until the final data export<br>• Data utility: results of the clinical trial have the potential to change clinical practice of immunosuppressive dosage after kidney transplantation, thereby reducing infections and immunologic complications
**2. FAIR Data**<br>2.1. Making data findable, including provisions for metadata	• Metadata provision: data ware produced in with a standard identification mechanism (unique patient identifiers)<br>• Naming conventions: creation of the database is based on CDISC (Clinical Data Interchange Standards Consortium)<br>• Approach towards search keyword: By following CDISC and the associated standardization, further use is supported. However, there is no indexing in the naming of the data fields in the database<br>• Approach for clear versioning: a versioning is performed<br>• Creation of following .txt files:<br>○ SAS_Data.txt: Contains the data (separator tab stop).<br>○ SAS_Label.txt: Contains the coding and the corresponding name of the questions.<br>○ SAS_Category.txt: Contains the coding of the categories and the designation to the respective coding.<br>○ SAS_QLU.txt: Contains the formats of all fields, e.g. Text, Category, Date/Time.<br>○ SAS_Format.txt: Specifies the format of Date/Time-questions, e.g. ddmmyy or time.<br>○ SAS_Type.txt: Specifies the format of all fields, e.g. $50 means text field with max. 50 characters. Here also the specifications for the formats of the Date/Time-Questions appear redundantly.<br>○ Other possible export formats: .xml and .csv

2.2 Making data openly accessible	• Personal data are collected, stored and evaluated in the database in a pseudonymized form<br>• Publication of results as required by the EU Regulation 536/2014 and in scientific journals<br>• For general publication only anonymized can be provided due to data protection reasons
2.3. Making data interoperable	• To be described later
2.4. Increase data re-use (through clarifying licences)	• Data will be made available for re-use earliest after completion and publication of the final trial report (must be published by law within one year after the end of the study)<br>• personal identifying data is not made available for use<br>• duration of data storage: 25 years<br>• Monitoring of data quality: According to SOPs of the lead CTU (KKS Dresden), preparing of a Monitoring Manual as well as description of implementation of data verification in the KKS Dresden Data Management Plan Module 4.
**3. Allocation of resources**	• Responsibility for data management: KKS Dresden as representative of the Sponsor of the clinical trial<br>• Resources for long-term preservation: not applicable
**4. Data security**	• At KKS Dresden: files are stored in a data management system, in a separate partition with exclusive read authorization<br>• Archiving of the TMF: data are stored on CD and will be archived at the Sponsor<br>• Sponsor is responsible to ensure the readability of the data during archiving
**5. Ethical aspects**	• Collection, storage and evaluation of data in a pseudonymized way<br>• Participation in the clinical trial only possible after patient informed consent, which includes the consent for collection and processing of data<br>• Ethics committees review the informed consent form in the course of the clinical trial approval process
**6. Other**	• Data management is performed according to following regulations: EU regulation 536/2014; EU regulation (EU) 2016/679; National laws on clinical trials (e.g. AMG); Grand Agreement of the project; , ICH-GCP E6 (R2), EMA/INS/GCP/454280/2010, FDA CFR Part 11 and the SOPs of the Lead CTU (KKS Dresden)

HISTORY OF CHANGES		
**Version**	**Publication date**	**Change**
1.0	26.10.2021	▪ Initial version
