# Supplementary material for: A multicentre, patient- and assessor-blinded, non-inferiority, randomised and controlled phase II trial to compare standard and torque teno virus-guided immunosuppression in kidney transplant recipients in the first year after transplantation: TTVguideIT
Source: Trials. 2023 Mar 22;24:213. doi: 10.1186/s13063-023-07216-0 (PMC10032258; doi:10.1186/s13063-023-07216-0)
Supplement: Supplementary file 3 — Additional file 3. [file 13063_2023_7216_MOESM3_ESM.pdf]

A randomised and controlled trial to compare the safety, tolerability and preliminary efficacy between standard and Torque Teno virus -guided immunosuppression in stable adult kidney transplantet recipients with low immunological risk in the first year after transplantation

Randomised and controlled, interventional, two-arm, patient and assessor-blinded, multinational, investigator driven phase II

EudraCT-Number: 2021-002525-24

Sponsor Code: TTV GUIDE IT

Database:

Site-No.:

Patient-No.: *Please note! A change of the patient number is not possible after successful registration in the database.*

Informed consent has been signed?
- ◎ no
- ◎ yes

Consent to sampling biomaterials for biobank:
- ◎ no
- ◎ yes

Date of informed consent:
dd/mm/yyyy

Have any protocol or GCP deviations occurred on this eForm?
- ◎ no
- ◎ yes

If yes, please document all protocol or GCP deviations for this eForm here (max. 3 entries):

Description:	(Possible) Reason:
...	...

Sponsor Code: TTV GUIDE II
EudraCT-Number: 2021-002525-24

Visit:

Site-No.: **Patient-No.:** **Arm:** **Database:** *Only applicable in eCRF!*

TTV analysis

Tabel: TTV anaylsis, max. 10 rows

Visit cycle:	Sample receipt:	Date of receipt: dd/mm/yyyy	Date of result: dd/mm/yyyy	TTV result: (log10 c/mL)

Have any protocol or GCP deviations occurred on this eForm?

◎ **no**

◎ **yes**

If yes, please document all protocol or GCP deviations for this eForm here (max. 3 entries):

Description:	(Possible) Reason:

Sponsor Code: TTV GUIDE IT
EudraCT-Number: 2021-002525-24

Visit:

Site-No.: Patient-No.: Arm: Database: *Only applicable in eCRF!*

Visit date

Was the visit done?

◎ no
◎ yes

If no, reason:

If other reason, please specify:

If yes, date: dd/mm/yyyy

If the visit was not done or not within timeframe according to study protocol, please document the reason as protocol deviation (see table below).

Have any protocol or GCP deviations occurred on this eForm?

◎ no
◎ yes

If yes, please document all protocol or GCP deviations for this eForm here (max. 3 entries):

Description:	(Possible) Reason:
...	...

TTV GUIDE

Sponsor Code: TTV GUIDE IT
EudraCT-Number: 2021-002525-24

Visit: ____

Site-No.: ____ Patient-No.: ____ Arm: ____ Database: ____ *Only applicable in eCRF!*

Routine phone calls between the visits

(every second week)

Has a routine phone call been made since the last visit?

◎ no
◎ yes
◎ Patient was not available

☞ **If yes, date of phone call:** ____ dd/mm/yyyy

☞ **Has the patient reported any adverse events (e.g., infections or rejections) or been hospitalized since the last visit?**

◎ no
◎ yes

☞ ***If yes, please document this as an adverse event and if necessary on the appropriate continuing eForms for SAE Reporting, Infection and Rejection.***

Go to eForm "Adverse events"

Go to eForm "SAE report"

Has another routine phone call occurred since the last visit?

◎ no
◎ yes
◎ Patient was not available

☞ **If yes, date of phone call:** ____ dd/mm/yyyy

☞ **Has the patient reported any adverse events (e.g., infections or rejections) or been hospitalized since the last visit?**

◎ no
◎ yes

☞ ***If yes, please document this as an adverse event and if necessary on the appropriate continuing eForms for SAE Reporting, Infection and Rejection.***

Go to eForm "Adverse events"

Go to eForm "SAE report"

Have any protocol or GCP deviations occurred on this eForm?

◎ no
◎ yes

If yes, please document all protocol or GCP deviations for this eForm here (max. 3 entries):

Description:	(Possible) Reason:
...	...

Sponsor Code: TTV GUIDE IT
EudraCT-Number: 2021-002525-24

Visit:

Site-No.: Patient-No.: Database:

Demographic data

Year of birth: ☐ **Age (years):** ☐

Sex:
- ◎ male
- ◎ female

Ethnicity:
- ◎ White or Caucasian
- ◎ Black or African Descent
- ◎ Asian
- ◎ Other

☞ **If other, please specify:**

Have any protocol or GCP deviations occurred on this eForm?
- ◎ no
- ◎ yes

If yes, please document all protocol or GCP deviations for this eForm here (max. 3 entries):

Description:	(Possible) Reason:
...	...

Sponsor Code: TTV GUIDE IT
EudraCT-Number: 2021-002525-24

Visit:

Site-No.: Patient-No.: Database: Only applicable in eC

Recipient details

Cause of end-stage renal disease:

In case of multiple causes for chronic kidney failure, please select the leading cause.

- ◎ Glomerulonephritis
- ◎ Kidney disease due to diabetes mellitus
- ◎ Hypertensive kidney disease
- ◎ Interstitial nephritis
- ◎ Polycystic kidney disease
- ◎ Urologic disease
- ◎ Uncertain
- ◎ Other

If glomerulonephritis, please specify:

If interstitial nephritis, please specify:

If polycystic kidney disease, please specify:

If urologic disease, please specify:

If uncertain or other cause of end-stage renal disease, please specify:

The diagnosis was proven by:

If other, please specify:

Previous kidney transplantation and replacement therapies:

Number of previous kidney transplants:

Last kidney replacement therapy before current transplant:

Start of last kidney replacement therapy before current transplant: dd/mm/yyyy

Blood group:

Blood group: **Rhesus factor:**

HLA typing:

Please enter the 4-digit HLA typing as follows:
Allele 1: A1*01*01
Allele 2: A3*03*01

	Allele 1:	*Allele 2:*
**HLA-A:**	*HLA-A allele 1:*	*HLA-A allele 2:*
**HLA-B:**	*HLA-B allele 1:*	*HLA-B allele 2:*
**HLA-C:**	*HLA-C allele 1:*	*HLA-C allele 2:*
**HLA-DRB1:**	*HLA-DRB1 allele 1:*	*HLA-DRB1 allele 2:*
**HLA-DRB3:**	*HLA-DRB3 allele 1:*	*HLA-DRB3 allele 2:*
**HLA-DRB4:**	*HLA-DRB4 allele 1:*	*HLA-DRB4 allele 2:*
**HLA-DRB5:**	*HLA-DRB5 allele 1:*	*HLA-DRB5 allele 2:*
**HLA-DQA1:**	*HLA-DQA1 allele 1:*	*HLA-DQA1 allele 2:*
**HLA-DQB1:**	*HLA-DQB1 allele 1:*	*HLA-DQB1 allele 2:*

HLA-DPA1: *HLA-DPA1 allele 1:* ______ *HLA-DPA1 allele 2:* ______

HLA-DPB1: *HLA-DPB1 allele 1:* ______ *HLA-DPB1 allele 2:* ______

Virus serology before current transplant

CMV IgG:

◎ negative
◎ positive
◎ unknown

EBV IgG:

◎ negative
◎ positive
◎ unknown

HCV antibody:

◎ negative
◎ positive
◎ unknown

HBs antibody:

◎ negative
◎ positive
◎ unknown

HBc antibody:

◎ negative
◎ positive
◎ unknown

Have any protocol or GCP deviations occurred on this eForm?

◎ no
◎ yes

If yes, please document all protocol or GCP deviations for this eForm here (max. 3 entries):

Description:	(Possible) Reason:
...	...

TTV GUIDE

Sponsor Code: TTV GUIDE IT
EudraCT-Number: 2021-002525-24

Visit:

Site-No.: Patient-No.: Database: *Only applicable in eCRF!*

Donor details

Age (years):

Sex:

◎ male
◎ female

Type of donation:

◎ living donation
◎ diseased donor
◎ donation after cardiac death

If living donation, relationship:

◎ related
◎ unrelated

Blood group:

Blood group: **Rhesus factor:**

HLA typing:

Please enter the 4-digit HLA typing as follows:
Allele 1: A1*01*01
Allele 2: A3*03*01

	Allele 1:	*Allele 2:*
**HLA-A:**	*HLA-A allele 1:*	*HLA-A allele 2:*
**HLA-B:**	*HLA-B allele 1:*	*HLA-B allele 2:*
**HLA-C:**	*HLA-C allele 1:*	*HLA-C allele 2:*
**HLA-DRB1:**	*HLA-DRB1 allele 1:*	*HLA-DRB1 allele 2:*
**HLA-DRB3:**	*HLA-DRB3 allele 1:*	*HLA-DRB3 allele 2:*
**HLA-DRB4:**	*HLA-DRB4 allele 1:*	*HLA-DRB4 allele 2:*
**HLA-DRB5:**	*HLA-DRB5 allele 1:*	*HLA-DRB5 allele 2:*
**HLA-DQA1:**	*HLA-DQA1 allele 1:*	*HLA-DQA1 allele 2:*
**HLA-DQB1:**	*HLA-DQB1 allele 1:*	*HLA-DQB1 allele 2:*
**HLA-DPA1:**	*HLA-DPA1 allele 1:*	*HLA-DPA1 allele 2:*
**HLA-DPB1:**	*HLA-DPB1 allele 1:*	*HLA-DPB1 allele 2:*

Virus serology

CMV IgG:

◎ negative
◎ positive
◎ unknown

EBV IgG:

◎ negative
◎ positive
◎ unknown

Have any protocol or GCP deviations occurred on this eForm?

◎ no
◎ yes

If yes, please document all protocol or GCP deviations for this eForm here (max. 3 entries):

Description: **(Possible) Reason:**

Sponsor Code: TTV GUIDE IT
EudraCT-Number: 2021-002525-24

Visit:

Site-No.: Patient-No.: Database:

Only applicable in eCRF!

Transplant details

Date of current transplantation: dd/mm/yyyy

HLA mismatch: HLA-A: HLA-B: HLA-DR:

Has induction therapy been administered?

◎ no
◎ yes

If yes, please specify the induction therapy:

If other induction therapy, please specify:

Initial Immunosppression

Tabel: Initial Immunosuppression, max. 4 rows

Active substance:	Other initial therapy (only active substances):	Further medication?

Prophylaxis

Has CMV prophylaxis been administered?

◎ no
◎ yes

If yes, please document it on the eForm "Concomitant medication"

Has PCP prophylaxis been administered?

◎ no
◎ yes

If yes, please document is on the eForm "Concomitant medication"

Go to eForm "Concomitant medication"

Have any protocol or GCP deviations occurred on this eForm?

◎ no
◎ yes

If yes, please document all protocol or GCP deviations for this eForm here (max. 3 entries):

Description:	(Possible) Reason:
...	...

Sponsor Code: TTV GUIDE IT
EudraCT-Number: 2021-002525-24

Visit:

Site-No.: Patient-No.: Database:

Only applicable in eCRF

Inclusion / Exclusion criteria at screening

Inclusion criteria

1. **Recipient of a kidney allograft**
   automatic entry by the database from the eForm "Transplant details"
   Go to eForm "Transplant details"
   ◎ no ◎ yes

2. **Adult (>= 18 years of age)**
   automatic entry by the database from the eForm "Demography"
   Go to eForm "Demography"
   ◎ no ◎ yes

3. **TAC-based immunosuppression**
   ◎ no ◎ yes

4. **Written informed consent**
   automatic entry by the database from the eForm "Registration"
   Go to eForm "Registration"
   ◎ no ◎ yes

Exclusion criteria

1. **HLA incompatible transplantation (as defined by local centre; e.g. performed DSA and/or crossmatch conversion)**
   ◎ no ◎ yes

2. **AB0 incompatible transplantation (as defined by local centre; e.g. relevant AB0 incompatible blood group combination)**
   ◎ no ◎ yes

3. **Combined transplantation**
   ◎ no ◎ yes

4. **History of HIV or active Hep B/C infection**
   ◎ no ◎ yes

5. **Donor with history of HIV or Hep B/C**
   ◎ no ◎ yes

6. **Hypersensitivity to TAC or other macrolides and hypersensitivity to any excipients**
   ◎ no ◎ yes

7. **Cyclosporine, mTor inhibitor or Co-stimulation blocker based immunosuppression**
   ◎ no ◎ yes

8. **Inability to perform study visits at the trial centre**
   ◎ no ◎ yes

9. **Any state that excludes adherence with the trial protocol, such as serious medical or psychiatric illness, language barrier, alcohol or illicit substance abuse or non-adherence**
   ◎ no ◎ yes

10. **Simultaneous participation in another interventional clinical trial**
    ◎ no ◎ yes

All inclusion an none of the exclusion criteria at screening met?
◎ no ◎ yes

Have any protocol or GCP deviations occurred on this eForm?

◎ **no**

◎ **yes**

If yes, please document all protocol or GCP deviations for this eForm here (max. 3 entries):

**Description:**	**(Possible) Reason:**
...	...

Sponsor Code: TTV GUIDE IT
EudraCT-Number: 2021-002525-24

Visit:

Site-No.: Patient-No.: Arm: Database: *Only applicable in eCRF!*

Concomitant diseases

(One more) pre-existing or concomitant disease?

◎ no
◎ yes ☞ **If yes, please provide further information**

No. eCRF:
automatic entry

Selection of concomitant disease:
◎ **History of diabetes**
◎ **History of major cadiovascular diseases**
◎ **History of immunologic diseases**
◎ **History of oncologic diseases**
◎ **History of other significant diseases**

Description (Medical term):

Is this concomitant disease still current at the start of the study?

◎ no
◎ yes

Is this concomitant disease currently being treated?

◎ no
◎ yes ☞ ***If yes, please document all necessary medication for the treatment of this concomitant disease at eCRF section "Concomitant medication".***

Go to eForm "Concomitant medication"

Have any protocol or GCP deviations occurred on this eForm?

◎ no
◎ yes

If yes, please document all protocol or GCP deviations for this eForm here (max. 3 entries):

Description: ... **(Possible) Reason:** ...

Sponsor Code: TTV GUIDE IT
EudraCT-Number: 2021-002525-24

Visit:

Site-No.: Patient-No.: Arm: Database: *Only applicable in eCRF!*

Complications after transplantation

(One more) complication after current transplant?

◎ no
◎ yes

☞ **If yes, please provide further information**

No. eCRF:

automatic entry

Selection of complication after NTX:

◎ **Graft rejection**
◎ **de novo DSA**
◎ **Relevant infections**
◎ **New onset diabetes after transplant**
◎ **Other**

Description (Medical term):

Start date:

dd/mm/yyyy

Is this complication still current at the start of the study?

◎ no
◎ yes

Is this complication currently being treated?

◎ no
◎ yes

☞ ***If yes, please document all necessary medication for the treatment of this complication at eCRF section "Concomitant medication".***

Go to eForm "Concomitant medication"

Was a kidney biopsy performed?
Please document on this eForm only kidney biopsies that have performed up to visit 1.

◎ no
◎ yes

☞ **If yes, date of biopsy:**

dd/mm/yyyy

Is this graft rejection being treated (incl. additional immunosuppression)?

◎ no
◎ yes

☞ ***If yes, please document all necessary medication for the treatment of this graft rejection at eCRF section "Concomitant medication".***

Go to eForm "Concomitant medication"

Postop. day:

automatic entry

BANFF Classification:
based on the Banff 2019 Kidney Meeting Report

Normal biopsy or nonspecific changes:

◎ no
◎ yes

Active antibody-mediated rejection:

◎ no
◎ yes

Chronic active antibody-mediated rejection:

◎ no
◎ yes

Chronic (inactive) antibody-mediated rejection:

◎ no
◎ yes

C4d staining without evidence of rejection:

◎ no
◎ yes

Borderline (suspicious) for acute T-cell-mediated rejection:

◎ no

◎ yes

Acute T-cell-mediated rejection:

Chronic active T-cell-mediated rejection:

Have any protocol or GCP deviations occurred on this eForm?

◎ no

◎ yes

If yes, please document all protocol or GCP deviations for this eForm here (max. 3 entries):

Description:	(Possible) Reason:
...	...

Sponsor Code: TTV GUIDE IT
EudraCT-Number: 2021-002525-24

Visit:

Site-No.: Patient-No.: Arm: Database: *Only applicable in eCRF!*

Physical examination and Vital signs

Was a physical examination performed?

◎ no
◎ yes

If no, reason:

If yes, date: dd/mm/yyyy

Please specify abnormal findings:

Were the vital signs obtained?

◎ no
◎ yes

If no, reason:

If yes, date: dd/mm/yyyy

Parameter:	Value:
Height: (m)	
Weight: (kg)	
BMI:	*automatic entry*
Blood pressure: (mmHg)	systolic / diastolic
Pulse: (1/min)	
Respiratory rate: (1/min)	
Temperature: (°C)	

Have any protocol or GCP deviations occurred on this eForm?

◎ no
◎ yes

If yes, please document all protocol or GCP deviations for this eForm here (max. 3 entries):

Description:	(Possible) Reason:
...	...

Sponsor Code: TTV GUIDE IT
EudraCT-Number: 2021-002525-24

Visit: ____

Site-No.: ____ Patient-No.: ____ Arm: ____ Database: ____ *Only applicable in eCRF!*

TTV level

Was the sample shipped to the laboratory?

◎ no ☞ **If no, reason:** ____

◎ yes ☞ **Date of shippment:** ____ dd/mm/yyyy

Please note that this section of the eForm is automatically filled in by the database.

Sample receipt in the laboratory:

◎ no

◎ yes ☞ **Date of receipt:** ____ dd/mm/yyyy

TTV result available:

◎ no

◎ yes ☞ **Date of result:** ____ dd/mm/yyyy

☞ **TTV result:** ____ **log10 c/mL**

Please note that onwards Visit 1 the TTV result is only displayed for patients randomised to the TTV guided arm.

Have any protocol or GCP deviations occurred on this eForm?

◎ no

◎ yes

If yes, please document all protocol or GCP deviations for this eForm here (max. 3 entries):

Description:	(Possible) Reason:
...	...

Sponsor Code: TTV GUIDE IT
EudraCT-Number: 2021-002525-24

Visit:

Site-No.: Patient-No.: Arm: Database: *Only applicable in eCRF!*

Differential blood count

Has the differential blood count been done?

◎ no — If no, reason:

◎ yes — If yes, date: dd/mm/yyyy

Parameter:	Value:	Unit:	Other unit:
Leukocytes:		*Leukocytes unit: Gpt/l (10^9/l, G/L), /µl, /nl (10^3/µl, 1000/µl)*	*Leukocytes other unit:*
Neutrophiles:		*Neutrophiles unit: Gpt/l (10^9/l, G/L), /µl, /nl (10^3/µl, 1000/µl), %, ratio*	*Neutrophiles other unit:*
Erythrocytes:		*Erythrocytes unit: Tpt/l (10^6/µl), /pl*	*Erythrocytes other unit:*
Haemoglobin:		*Haemoglobin unit: g/l, g/dl, mg/dl, mmol/l*	*Haemoglobin other unit:*
Haematocrit:		*Haematocrit unit: %, ratio, l/l*	*Haematocrit other unit:*
Platelets:		*Platelets unit: Gpt/l (10^9/l, G/L), /nl (10^3/µl, 1000/µl), /µl*	*Platelets other unit:*

Have any protocol or GCP deviations occurred on this eForm?

◎ no

◎ yes

If yes, please document all protocol or GCP deviations for this eForm here (max. 3 entries):

Description: **(Possible) Reason:**

Sponsor Code: TTV GUIDE IT
EudraCT-Number: 2021-002525-24

Visit:

Site-No.: Patient-No.: Arm: Database:

Only applicable in eCRF!

Clinical chemistry - Part 1

Has the clinical chemistry been done?

◎ no

☞ **If no, reason:**

◎ yes

☞ **If yes, date:**

dd/mm/yyyy

Parameter:	Value:	Unit:	Other unit:
**Tacrolimus:**		*TAC unit:* *ng/ml*	
**CRP:**		*CRP unit:* *g/l, g/dl, mg/l, mg/dl, µg/dl*	
Sodium stand.:			
**Sodium:**		*Sodium unit:* *g/l, g/dl, mg/dl, mmol/l, mEq/l, mval/l*	*Sodium other unit:*
**Potassium:**		*Potassium unit:* *g/l, g/dl, mg/l, mg/dl, mmol/l, mEq/l, mval/l*	*Potassium other unit:*
**Calcium:**		*Calcium unit:* *g/l, g/dl, mg/dl, mmol/l, mEq/l, mval/l*	*Calcium other unit:*
**Magnesium:**		*Magnesium unit:* *g/l, g/dl, mg/dl, mmol/l, mEq/l, mval/l*	*Magnesium other unit:*
**Phosphate:**		*Phosphate unit:* *g/l, g/dl, mg/dl, mmol/l, mEq/l, mval/l*	*Phosphate other unit:*
**LDH:**		*LDH unit:* *µkat/l (µkatal/l, µmol/s*l), U/l, nkat/l*	*LDH other unit:*
**HbA1c:**		*HbA1c unit:* *mmol/mol, %*	*HbA1c other unit:*
**Blood Glucose:**		*Glucose unit:* *g/l, g/dl, mg/dl, mmol/l*	*Glucose other unit:*
**Total Cholesterol:**		*Total Cholesterol unit:* *g/l, g/dl, mg/dl, mmol/l*	*Total Cholesterol other unit:*
**Triglycerides:**		*Triglycerides unit:* *g/l, g/dl, mg/dl, mmol/l*	*Triglycerides other unit:*

Have any protocol or GCP deviations occurred on this eForm?

◎ **no**

◎ **yes**

If yes, please document all protocol or GCP deviations for this eForm here (max. 3 entries):

**Description:**	**(Possible) Reason:**
...	...

TTV GUIDE
Torque Teno Virus
Based Immune Monitoring

Sponsor Code: TTV GUIDE IT
EudraCT-Number: 2021-002525-24

Visit:

Site-No.: Patient-No.: Arm: Database:

Only applicable in eCRF!

Clinical chemistry - Part 2

Parameter:	Value:	Unit:	Other unit:
Creatinine stand.: / **Creatinine:**		*Creatinine unit: g/l, g/dl, mg/dl, mmol/l, µmol/l*	*Creatinine other unit:* ...
**Albumin:**		*Albumin unit: g/l, g/dl, mg/dl, g%, mmol/l, µmol/l*	*Albumin other unit:* ...
**Total protein:**		*Total protein unit: g/l, g/dl, mg/dl*	*Total protein other unit:* ...
**ALAT (SGPT):**		*ALAT (SGPT) unit: µkat/l (µkatal/l, µmol/s*l), U/l, nkat/l*	*ALAT (SGPT) other unit:* ...
**ASAT (SGOT):**		*ASAT (SGOT) unit: µkat/l (µkatal/l, µmol/s*l), U/l, nkat/l*	*ASAT (SGOT) other unit:* ...
**GGT:**		*GGT unit: µkat/l (µkatal/l, µmol/s*l), U/l, nkat/l*	*GGT other unit:* ...
**Blood Urea:**		*Blood Urea unit: g/l, g/dl, mg/l, mg/dl, µg/ml, mmol/l, µmol/l*	*Blood Urea other unit:* ...
**Blood Urea nitrogen:**		*Blood Urea nitrogen unit: mg/dl, µg/ml, mmol/l, µmol/l*	*Blood Urea nitrogen other unit:* ...

Has the blood gas analysis been done?

◎ no ☞ **If no, reason:**

◎ yes ☞ **If yes, date:** dd/mm/yyyy

Parameter:	Value:	Unit:	Other unit:
**pH Value:**			
**Base excess:**	*Base excess prefix:* ◎ - ◎ +	*Base excess unit: mmol/l*	
**Bicarbonate:**		*Bicarbonate unit: g/l, g/dl, mg/l, mg/dl, µg/ml, mmol/l, µmol/l, mEq/l*	*Bicarbonate other unit:* ...

Have any protocol or GCP deviations occurred on this eForm?

◎ **no**

◎ **yes**

If yes, please document all protocol or GCP deviations for this eForm here (max. 3 entries):

**Description:**	**(Possible) Reason:**
...	...

Sponsor Code: TTV GUIDE IT
EudraCT-Number: 2021-002525-24

Visit:

Site-No.: Patient-No.: Arm: Database: *Only applicable in eCRF!*

Urinalysis

Was the urinalysis been done?

◎ no — If no, reason:

◎ yes — If yes, date: dd/mm/yyyy

Parameter:	Value:	Unit:
Protein/Creatinine Ratio:		*Protein/Creatinine Ratio unit: mg/g, mg/mmol*
Albumin/Creatinine Ratio:		*Albumin/Creatinine Ratio unit: mg/g, mg/mmol*

Have any protocol or GCP deviations occurred on this eForm?

◎ no

◎ yes

If yes, please document all protocol or GCP deviations for this eForm here (max. 3 entries):

Description:	(Possible) Reason:

Sponsor Code: TTV GUIDE IT
EudraCT-Number: 2021-002525-24

Visit:

Site-No.: Patient-No.: Arm: Database: *Only applicable in eCRF!*

Virology

Has the virological examination been done?

◎ no
◎ yes

If yes, date: dd/mm/yyyy

Parameter:	Test done?	Value:	Unit:	Other unit:
**Cytomegaly virus:** *(blood)*	*CMV test done:*		*CMV unit: IU/ml, Copies/µl*	*CMV other unit:*
**Polyomavirus:** *(blood)*	*BKV (blood) test done:*		*BKV (blood) unit: IU/ml, Copies/µl*	*BKV other unit:*
**Polyomavirus:** *(urine)*	*BKV (urine) test done:*		*BKV (urine) unit: IU/ml, Copies/µl*	*BKV (urine) other unit:*
**Decoy cells:** *(urine)*	*Decoy cells test done:*		*Decoy cells unit: %*	

Have any protocol or GCP deviations occurred on this eForm?

◎ no
◎ yes

If applicable, please document all protocol or GCP deviations for this eForm here (max. 3 entries):

Description:	(Possible) Reason:

Sponsor Code: TTV GUIDE IT
EudraCT-Number: 2021-002525-24

Visit:

Site-No.: Patient-No.: Arm: Database: *Only applicable in eCRF!*

Donor-specific antibodies

Was the HLA serology been done?

◎ no
◎ yes

☞ **If no, reason:**

☞ **If yes, date:** dd/mm/yyyy

Were HLA antibodies detectable?

◎ no
◎ yes

☞ **If yes, please specify:**
◎ de novo
◎ pre-existing before NTX

Donor specific:
◎ no
◎ yes

Specificity:

MFI result:

Test method:
◎ single antigen bead
◎ other

☞ **If other, please specify:**

Have any protocol or GCP deviations occurred on this eForm?

◎ no
◎ yes

If yes, please document all protocol or GCP deviations for this eForm here (max. 3 entries):

Description: ...

(Possible) Reason: ...

Sponsor Code: TTV GUIDE IT
EudraCT-Number: 2021-002525-24

Visit:

Site-No.: Patient-No.: Arm: Database: *Only applicable in eCRF!*

Pregnancy test

Was a pregnancy test performed?

◎ no
◎ yes

☞ **If no, main reason:**
◎ Menopause
◎ Sterilization
◎ Other reason

☞ **If other reason, please specify:**

☞ **If yes, Date:** dd/mm/yyyy

Result:
◎ negative
◎ positive

Have any protocol or GCP deviations occurred on this eForm?

◎ no
◎ yes

If yes, please document all protocol or GCP deviations for this eForm here (max. 3 entries):

Description:	(Possible) Reason:
...	...

Sponsor Code: TTV GUIDE II
EudraCT-Number: 2021-002525-24

Visit:

Site-No.: Patient-No.: Database: *Only applicable in eCRF!*

Inclusion criteria at Visit 1

1. Recipient of a kidney allograft
automatic entry by the database from the eForm "Transplant details"
Go to eForm "Transplant details"
◎ no
◎ yes

2. Adult (>= 18 years of age)
automatic entry by the database from the eForm "Demography"
Go to eForm "Demography"
◎ no
◎ yes

3. Post day 93 following transplantation
◎ no
◎ yes

4. TAC-based immunosuppression
◎ no
◎ yes

5. Standard target TAC trough level (as defined by local centre; might exclude patients with e.g. a lung transplantation or de novo DSA or thrombotic microangiopathy (TMA) if the centre applies non-standard TAC trough levels in these circumstances)
◎ no
◎ yes

6. Written informed consent
automatic entry by the database from the eForm "Registration"
Go to eForm "Registration"
◎ no
◎ yes

Have any protocol or GCP deviations occurred on this eForm?

◎ no
◎ yes

If yes, please document all protocol or GCP deviations for this eForm here (max. 3 entries):

Description:	(Possible) Reason:
...	...

Sponsor Code: TTV GUIDE IT
EudraCT-Number: 2021-002525-24

Visit:

Site-No.: Patient-No.: Database: *Only applicable in eCRF!*

Exclusion criteria at Visit 1

1.	**HLA incompatible transplantation (as defined by local centre; e.g. performed DSA and/or crossmatch conversion)**<br>*automatic entry by the database from the eForm "Inclusion/Exclusion criteria at screening"*<br>Go to eForm "Inclusion/Exclusion criteria at screening"	◎ no<br>◎ yes
2.	**AB0 incompatible transplantation (as defined by local centre; e.g. relevant AB0 incompatible blood group combination)**<br>*automatic entry by the database from the eForm "Inclusion/Exclusion criteria at screening"*<br>Go to eForm "Inclusion/Exclusion criteria at screening"	◎ no<br>◎ yes
3.	**Combined transplantation**<br>*automatic entry by the database from the eForm "Inclusion/Exclusion criteria at screening"*<br>Go to eForm "Inclusion/Exclusion criteria at screening"	◎ no<br>◎ yes
4.	**History of HIV or active Hep B/C infection**	◎ no<br>◎ yes
5.	**Donor with history of HIV or Hep B/c**<br>*automatic entry by the database from the eForm "Inclusion/Exclusion criteria at screening"*<br>Go to eForm "Inclusion/Exclusion criteria at screening"	◎ no<br>◎ yes
6.	**TTV load always below 4.6 log10 c/mL during screeing phase**	◎ no<br>◎ yes
7.	**No stable TAC through levels achieved during screening phase (as defined by local centre)**	◎ no<br>◎ yes
8.	**Hypersensitivity to TAC or other macrolides and hypersensitivity to any excipients**	◎ no<br>◎ yes
9.	**Cyclosporine, mTor inhibitor or co-stimulation blocker based immunosuppression**	◎ no<br>◎ yes
10.	**No standard immunosuppression according to local centre definition; e.g. necessity of significant additional long term immunosuppression or immune modultion (e.g. disease modifying agents in autoimmune disease or immune modulators for cancer)**	◎ no<br>◎ yes
11.	**Treatment with T-cell depleting drugs within 2 months before the randomisation (e.g. anti-thymocyte globulin)**	◎ no<br>◎ yes
12.	**Current infection or allograft rejection as defined by the primary end-point**	◎ no<br>◎ yes
13.	**Biopsy proven antibody mediated rejection (ABMR) or BK virus PCR >= 10^4 c/ml (or corresponding U/mL) in the blood until randomisation**	◎ no<br>◎ yes
14.	**Unstable graft function:**<br>*eGFR <25 mL/min/1.73m²*<br>*(this limit might be ignored if creatinine clearance is >25 mL/min/1.73m²)*<br>***or***<br>*rapid and relevant eGRF decline (as definied by local centre)*<br>***or***<br>*urinary protein / creatinine ration > 2000 mg/g*<br>***or***<br>*rapid and relevant increase (as defined by local centre)*	◎ no<br>◎ yes
15.	**Advanced liver failure (CHILD-Pugh Score C)**	◎ no<br>◎ yes

**16.**	**History of malignancy other than squamous cell carcinoma or basal cell carcinoma of the skin or carcinoma in situ or adenoma of the colon within the last 5 years unless in complete remission since at least 3 years**	◎ **no**<br>◎ **yes**
**17.**	**Leukopenia <2000/mm³ or neutropenia <1000/mm³**	◎ **no**<br>◎ **yes**
**18.**	**Unstable angina, cardiac decompensation with the necessity of inpatient treatment**	◎ **no**<br>◎ **yes**
**19.**	**Severe tremor (as defined by local centre) due to TAC**	◎ **no**<br>◎ **yes**
**20.**	**Inability to perform study visits at the trial centre**	◎ **no**<br>◎ **yes**
**21.**	**Any state that excludes adherence with the trial protocol, such as serious medical or psychiatric illness, language barrier, alcohol or illicit substance abuse or non-adherence**	◎ **no**<br>◎ **yes**
**22.**	**Addictions or other illnesses that do not allow the person concerned to assess the nature and extent of the clinical trial and its possible consequences**	◎ **no**<br>◎ **yes**
**23.**	**Simultaneous participation in another interventional clinical trial**<br>***automatic entry by the database from the eForm "Inclusion/Exclusion criteria at screening"***<br><u>**Go to eForm "Inclusion/Exclusion criteria at screening"**</u>	◎ **no**<br>◎ **yes**
**24.**	**Pregnant of breastfeeding woman**	◎ **no**<br>◎ **yes**
**25.**	**Women of childbearing potential, except women who meet (one of) the following criteria:**<br>*a) post-menopausal (12 months natural amenorrhoea)*<br>*b) postoperative (6 weeks after bilateral ovarectomy with or without hysterectomy, bilateral salpingectomy)*<br>*c) regular and correct use of a contraceptive methode with an Pearl Index < 1% per year*<br>*d) sexual abstinence*<br>*e) Vasectomy of the partner*	◎ **no**<br>◎ **yes**

**All inclusion and none of the exclusion criteria met?**	◎ **no**<br>◎ **yes**
**Will the patient be enrolled?**	◎ **no, Screening failure**<br>◎ **yes**

Have any protocol or GCP deviations occurred on this eForm?

◎ **no**
◎ **yes**

If yes, please document all protocol or GCP deviations for this eForm here (max. 3 entries):

**Description:**	**(Possible) Reason:**
...	...

Sponsor Code: TTV GUIDE TT
EudraCT-Number: 2021-002525-24

Visit:

Site-No.: Patient-No.: Database: *Only applicable in eCRF!*

Randomisation

Will the patient be randomised?

◎ no
◎ yes

☞ ***If No: Please save this eForm with the disk symbol in the top menu bar and then use the link below to go to the eForm "End of study" to document the reason.***

Go to eForm "End of study"

☞ ***If Yes: Please save this eForm with the disk symbol in the top of menu bar before you going to the next eForm "Randomisation - result".***

Have any protocol or GCP deviations occurred on this eForm?

◎ no
◎ yes

If yes, please document all protocol or GCP deviations for this eForm here (max. 3 entries):

Description:	(Possible) Reason:
...	...

Sponsor Code: TTV GUIDE II
EudraCT-Number: 2021-002525-24

Visit:

Site-No.: **Patient-No.:** **Database:** *Only applicable in eCRF!*

Randomisation result

If no results are displayed, please reopen this eForm after about 10 seconds.

Date of randomisation: ______ ***automatic entry***
dd/mm/yyyy

Randomisation result: ______

Please save this eForm when leaving.

Sponsor Code: TTV GUIDE II
EudraCT-Number: 2021-002525-24

Visit: [ ]

Site-No.: [ ] Patient-No.: [ ] Arm: [ ] Database: [ ] *Only applicable in eCRF!*

Intervention

Actual TAC level: [ ] **ng/ml**
automatic entry

Actual TAC dose [ ] **mg**

Frequency:
◎ **once daily**
◎ **twice daily**

Actual TTV level: [ ] **log10 c/mL**
automatic entry

☞ ***PLEASE NOTE! The TTV result is only displayed for patients randomised to the TTV arm.***

Has Tacrolimus been taken as prescribed since the last visit?

◎ **no**
◎ **yes**

☞ **If Tacrolimus not taken as prescribed, please specify:**

◎ **Failure of Tacrolimus intake more than once per week on average for twice daily.**
◎ **Failure of Tacrolimus intake more than once every two weeks on average for once daily.**

Has the study intervention been terminated prematurely since the last visit up to the current visit?
To be answered only on visits 2 to 5.

◎ **no**
◎ **yes**

☞ ***If yes, please continue to document all subsequent visits until visit 6 is reached according to the specification for the control group.***

☞ ***Please also document the date and reason for the end of intervention on eForm "End of intervention" in "Visit 6 / End of intervention".***

Go to eForm "End of intervention"

Active / Intervention group:

TTV range:
[ ]

TTV-guided TAC dosing:
If TTV is not within the optimal range, the TAC trough level target has to be adapted by one step up or down compared to the current TAC trough level.
One TAC trough level adaption step is defined as 2 ng/mL (investigators are allowed to target a range of +/-1 ng/mL; thus one step might be within a minimum of 1 ng/mL and a maximum of 3 ng/mL).

☞ **New TAC target:** [ ] **ng/ml**

☞ **New TAC dose:** [ ] **mg**

New TAC frequency:
◎ **once daily**
◎ **twice daily**

Control group:

Has a dose adjustment been done at this visit?

◎ **no**
◎ **yes**

☞ **If yes, new TAC dose:** [ ] **mg**

New TAC frequency:
◎ **once daily**
◎ **twice daily**

Have any protocol or GCP deviations occurred on this eForm?

◎ **no**

◎ **yes**

If yes, please document all protocol or GCP deviations for this eForm here (max. 3 entries):

**Description:**	**(Possible) Reason:**
...	...

TTV GUIDE

Sponsor Code: TTV GUIDE IT
EudraCT-Number: 2021-002525-24

Visit:

Site-No.: Patient-No.: Arm: Database:

Only applicable in eCRF!

MEMS® Button - Visit assessement

Only at Visit 1:

1. Has the MEMS® Button been initiated and dispensed?

◎ no ☞ **If no, reason:**

◎ yes ☞ **If yes, date:** dd/mm/yyyy **MEMS® Button ID:**

For Visit 2 to Visit 7:

2. Was the MEMS® Button brought to this visit?

◎ no

◎ yes ☞ **If no, reason:**

3. Has the MEMS® Button been read out?

◎ no

◎ yes ☞ **If yes, date:** ddmm/yyyy

4. If remembered, has the medication been taken without pushing the MEMS® Button?

◎ no

◎ yes ☞ **If yes, frequency:**

☞ **If yes, reason:**

5. Has the use of the MEMS® Button been interrupted for a period of time?

◎ no

◎ yes ☞ **If yes, from date:** dd/mm/yyyy **to date:** dd/mm/yyyy

☞ **If yes, reason:**

☞ **If other reason, please specify:**

6. Has the use of the MEMS® Button been terminated prematurely?

◎ no

◎ yes ☞ **If yes, reason:**

☞ **If other reason, please specify:**

7. Has the device been replaced by a newly initiated MEMS® Button?

◎ no

◎ yes ☞ **If yes, date of initiation:** dd/mm/yyyy **New MEMS® Button ID:**

Only at visit 7:

8.

Has the MEMS® Button been returned?

◎ no
◎ yes

☞ **If no, reason:** [ ]

☞ **Date of return:** [ ] dd/mm/yyyy

Have any protocol or GCP deviations occurred on this eForm?

◎ no
◎ yes

If yes, please document all protocol or GCP deviations for this eForm here (max. 3 entries):

Description:	(Possible) Reason:
...	...

Sponsor Code: TTV GUIDE II
EudraCT-Number: 2021-002525-24

Visit:

Site-No.: Patient-No.: Arm: Database: *Only applicable in eCRF!*

BAASIS© Questionnaire

The Basel Assessment of Adherence to immonoSuppressive medications Scale©

Has the BAASIS questionnaire been done?

☞ **If no, reason:**

◎ no
◎ yes

☞ **If yes, Date:**

dd/mm/yyyy

1. Can you first tell me which anti-rejection medications you have been prescribed, what dose you take of each and what time(s) you take them?

Table 1: max. 8 rows

What is the name of the anti-rejection medication(s) you take? (one medication per line)<br>Example: Prograf (tacrolimus)	Can you tell me what your dose is for that?<br>Example: 2 mg and 1,5 mg	And what time do you take that? Do you take it once a day or more often? And what time do you take the second dose? And the third?<br>Example: 8:00 and 20:00

1A. Did you miss any doses, even one, of any of your anti-rejection medications in the past weeks?

◎ no
◎ yes ☞ **If yes, could you tell me how often this happened in the past 4 weeks?**

◎ Once
◎ Twice
◎ Three times
◎ Four times
◎ More than four times

1B. Did you ever skip two or more doses in a row in the past 4 weeks?

Only to be completed if the answer to question 1A was 'yes'.

◎ no
◎ yes ☞ **If yes, could you tell me how often this happened in the past 4 weeks?**

◎ Once
◎ Twice
◎ Three times
◎ Four times
◎ More than four times

**2. You take your anti-rejection medications at exact time points described in Table 1.
Did you take any of your anti-rejection medication more than 2 hours before or after the(se) dosing time(s) in the past 4 weeks?**

◎ no
◎ yes ☞

If yes, could you tell me how often this happened in the past 4 weeks?

- ◎ **Once**
- ◎ **Two to three times**
- ◎ **About once weekly**
- ◎ **A couple of times per week**
- ◎ **Almost every day**

3. Have you changed the prescribed amount of any of your anti-rejection medications during the past 4 weeks, on your own initative without your doctor telling you to do that?

◎ **no** *For example, have you taken more or fewer pills or change the dose, maybe by cutting a pill in half?*

◎ **yes**

4. Have you completely stopped taking any of your anti-rejection medications within the past year, on your own initiative without your doctor telling you to do that?

◎ **no**

◎ **yes**

5. Did your family doctor or a specialist give you any prescriptions for any new medications in the past year? (Maybe high blood pressure medication, cholesterol/lipid lowering drugs?)

◎ **no**

◎ **yes** ☞ **If yes, did you fill the prescription at the pharmacy and start taking this new medication?**

◎ **no**

◎ **yes** ☞ *If yes, please document it on the eForm "Concomitant treatment"*

Go to eForm 'Concomitant treatment'

© University of Basel, Leuven-Basel Research Group, Institute of Nursing Science, Department Public Health, University of Basel, Switzerland, 2005. Permission & conditions to use the BAASIS© can be obtained from:

http://baasis.nursing.unibas.ch/ or sabina.degeest@unibas.ch

Have any protocol or GCP deviations occurred on this eForm?

◎ **no**

◎ **yes**

If yes, please document all protocol or GCP deviations for this eForm here (max. 3 entries):

Description:	(Possible) Reason:
...	...

TTV GUIDE

Sponsor Code: TTV GUIDE IT
EudraCT-Number: 2021-002525-24

Visit:

Site-No.: **Patient-No.:** **Arm:** **Database:** *Only applicable in eCRF!*

Drug account

Was the patient diary available at this visit?

☞ **If no, reason:**

◎ **no**
◎ **yes**

☞ **If yes, please transfer the entries up to the day of this visit.**

Have any new packages of Tacrolimus been opened since the last visit?

◎ **no**
◎ **yes**

☞ **If yes, please document each newly started package of Tacrolimus with the respective batch number.**

PLEASE NOTE! If it should be necessary to delete lines in this table, e.g. due to a query by the monitor, please select the answer BLANK ROW in the last column of the relevant line.

Table: New medication packages, max. 10 rows per eForm, max. 2 eForms

Date new package: (dd/mm/yyyy)	Medication name and dosage: (e.g. Advagrafe 1 mg)	Batch number:	New row?

Have any protocol or GCP deviations occurred on this eForm?

◎ **no**
◎ **yes**

If yes, please document all protocol or GCP deviations for this eForm here (max. 3 entries):

Description: **(Possible) Reason:**

Sponsor Code: TTV GUIDE IT
EudraCT-Number: 2021-002525-24

Visit:

Site-No.: Patient-No.: Arm: Database: *Only applicable in eCRF!*

Tacrolimus

Please document tacrolimus intake retrospectively from the date of the last visit to the date of the current visit.

For each interruption or change in intake, please document the reason for the interruption in the CURRENT ROW.
If the reason is an adverse event, please document it in the eCRF section "Adverse events" as well.

Go to eForm "Adverse events"

After an interruption or change in intake, please document the continuation in a NEW ROW.

Table: Tacrolimus exposure, max. 7 rows per eForm, max. 6 eForms

Start date: (dd/mm/yyyy)	End date: (dd/mm/yyyy)	Total daily dose: (mg)	Complaints / Infections occurred?	If complaints or infections occurred, please describe:	New row?

Further row = no means that all days of taken medication are fully documented according to the patient diary.

If it should be necessary to delete rows, e.g. due to a query by the monitor, please select the answer BLANK ROW in the last column of the relevant row.

Have any protocol or GCP deviations occurred on this eForm?

◎ **no**

◎ **yes**

If yes, please document all protocol or GCP deviations for this eForm here (max. 3 entries):

**Description:**	**(Possible) Reason:**

Sponsor Code: TTV GUIDE IT
EudraCT-Number: 2021-002525-24

Visit:

Site-No.: Patient-No.: Arm: Database: *Only applicable in eCRF!*

Psychological evaluation

Has the psychological evaluation been done?

◎ no
◎ yes

If no, reason:

If yes, Date: dd/mm/yyyy

1. **Social Status:**
   - ◎ Single
   - ◎ Married
   - ◎ Divorced
   - ◎ Separated
   - ◎ Widowed
   - ◎ In a stable relationship

2. **Children:**
   - ◎ no
   - ◎ yes

   If yes, how many:

3. **Level of education:**
   - ◎ No school degree
   - ◎ Primary education
   - ◎ Secondary education
   - ◎ High school degree
   - ◎ Vocational school degree
   - ◎ University degree

4. **Employment status:**
   - ◎ Employed
   - ◎ Self-employed
   - ◎ Sick leave
   - ◎ Pension/rehab
   - ◎ Pension/age
   - ◎ Unemployed

5. **Social support/resources:**

   Emotional and practical support from family
   - ◎ no
   - ◎ yes

   Emotional and practical support from friends
   - ◎ no
   - ◎ yes

6. **Addiction:**
   - ◎ Alcohol
   - ◎ Illegal substance abuse
   - ◎ Nicotine abuse
   - ◎ Medication abuse
   - ◎ None

   Accorting to WHO:
   for women: >12 mg/day = 0,3L beer, 0,13L wine or 4cL 38% alcohol;
   for men: >24 mg/day = 0,6L beer, 0,26L wine or 8cL 38% alcohol

7. **Critical life events within the last year:**
   (Loss, seperation, experience of violence, abuse, traumatic experiences, previous illnesses)

   - ◎ no
   - ◎ yes

   If yes, Date: dd/mm/yyyy

   Which event:

8. Psychatric History:
- ◎ none
- ◎ Post-traumatic stress disorder
- ◎ Depression
- ◎ Anxiety disorder
- ◎ Personality disorder
- ◎ Others

☞ **If others, please specify:** [ ]

Treatment:
- ◎ no
- ◎ yes

9. Transplantation and Treatment

**I received the best possible treatment.**	◎ no<br>◎ yes
**I receive information about the procedures and examinations that are important to me.**	◎ no<br>◎ yes
**I have the opportunity to participate in medical decisions concerning my care.**	◎ no<br>◎ yes
**The team gives me the feeling that they are "taking care of me".**	◎ no<br>◎ yes
**The team treats me with respect.**	◎ no<br>◎ yes
**There is an overall positive atmosphere in the team.**	◎ no<br>◎ yes

10. Future perspective and resources
Goals, motives, resources can be named (family, job, leisure time)
- ◎ no
- ◎ yes

11. Additional important information:

[ ]

Have any protocol or GCP deviations occurred on this eForm?
- ◎ no
- ◎ yes

If yes, please document all protocol or GCP deviations for this eForm here (max. 3 entries):

**Description:**	**(Possible) Reason:**
...	...

Sponsor Code: TTV GUIDE IT
EudraCT-Number: 2021-002525-24

Visit:

Site-No.: Patient-No.: Arm: Database:

Only applicable in eCRF!

SF-36 (English) for Health Related Quality of Life Assessment - TTV-GUIDE-IT

Has the SF-36 questionnaire been done?

◎ no — If no, reason:
◎ yes — If yes, Date: dd/mm/yyyy

1. In general, would you say your health is:

◎ 1 - Excellent
◎ 2 - Very good
◎ 3 - Good
◎ 4 - Fair
◎ 5 - Poor

2. Compared to one year ago, how would you rate your health in general now?

◎ 1 - Much better now than one year ago
◎ 2 - Somewhat better now than one year ago
◎ 3 - About the same
◎ 4 - Somewhat worse now than one year ago
◎ 5 - Much worse now than one year ago

The following items are about activities you might do during a typical day. Does your health now limit you in these activities? If so, how much?

answer options for question 3. to 12:
1 - Yes, limited a lot , 2 - Yes, limited a little, 3 - No, not limited at all

3. Vigorous activities, such as running, lifting heavy objects, participating in strenuous sports
4. Moderate activities, such as moving a table, pushing a vacuum cleaner, bowling, or playing golf
5. Lifting or carrying groceries
6. Climbing several flights of stairs
7. Climbing one flight of stairs
8. Bending, kneeling, or stooping
9. Walking more than a mile
10. Walking several blocks
11. Walking one block
12. Bathing or dressing yourself

During the past 4 weeks, have you had any of the following problems with your work or other regular daily activities as a result of your physical health?

13. Cut down the amount of time you spent on work or other activities
◎ 1 - yes
◎ 2 - no

14. Accomplished less than you would like
◎ 1 - yes
◎ 2 - no

15. Were limited in the kind of work or other activities
- ◎ 1 - yes
- ◎ 2 - no

16. Has difficulty performing the work or other activities (for example, it took extra effort)
- ◎ 1 - yes
- ◎ 2 - no

During the past 4 weeks, have you had any of the following problems with your work or other regular daily activities as a result of any emotional problems (such as feeling depressed or anxious)?

17. Cut down the amount of time you spent on work or other activities
- ◎ 1 - yes
- ◎ 2 - no

18. Accomplished less than you would like
- ◎ 1 - yes
- ◎ 2 - no

19. Didn't do work or other activities as carefully as usual
- ◎ 1 - yes
- ◎ 2 - no

20. During the past 4 weeks, to what extent has your physical health or emotional problems interfered with your normal social activities with family, friends, neighbors or groups?
- ◎ 1 - Not at all
- ◎ 2 - Slightly
- ◎ 3 - Moderately
- ◎ 4 - Quite a bit
- ◎ 5 - Extremely

21. How much bodily pain have you had during the past 4 weeks?
- ◎ 1 - None
- ◎ 2 - Very mild
- ◎ 3 - Mild
- ◎ 4 - Moderately
- ◎ 5 - Severe
- ◎ 6 - Very severe

22. During the past 4 weeks, how much did pain interfere with your normal work (including both work outside the home and housework)?
- ◎ 1 - Not at all
- ◎ 2 - A little bit
- ◎ 3 - Moderately
- ◎ 4 - Quite a bit
- ◎ 5 - Extremely

These questions are about how you feel and how things have been with you during the past 4 weeks. For each question, please give the on answer that comes closest to the way you have been feeling.

How much of the time during the past 4 weeks...

answer options for question 23. to 31.:
1 - All of the time, 2 - Most of the time, 3 - A good bit of the time
4 - Some of the time, 5 - A little of the time, 6 - None of the time

23. Did you feel full of pep?

24. Have you been a very nervous person?

25. Have you felt so down in the dumps that nothing could cheer you up?

26. Have you felt calm and peaceful?

27. Did you have a lot of energy?

28. Have you felt downhearted and blue?

29. Did you feel worn out?

30. Have you been a happy person?

31. Did you feel tired?

32. During the past 4 weeks, how much of the time has your physical health or emotional problems interfered with your social activities (like visiting with friends, relatives, etc.)?
- ◎ 1 - All of the time
- ◎ 2 - Most of the time
- ◎ 3 - Some of the time
- ◎ 4 - A little of the time
- ◎ 5 - None of the time

How TRUE or FALSE is each of the following statements for you.

answer options for question 33. to 36:
1 - Definitely true, 2 - Mostly true, 3 - Don't know
4 - Mostly false, 5 - Definitely false

33. I seem to get sick a little easier than other people

34. I am as healthy as anybody I know

35. I expect my health to get worse

36. My health is excellent

Have any protocol or GCP deviations occurred on this eForm?

◎ **no**

◎ **yes**

If yes, please document all protocol or GCP deviations for this eForm here (max. 3 entries):

**Description:**	**(Possible) Reason:**
...	...

Sponsor Code: TTV GUIDE II
EudraCT-Number: 2021-002525-24

Visit:

Site-No.: Patient-No.: Arm: Database: *Only applicable in eCRF!*

MTSOSD

Modified Transplant Symptom Occurrence and Symptom Distress Scale

Has the MTSOSD questionnaire been done?

◎ no — If no, reason:

◎ yes — If yes, Date: dd/mm/yyyy

During the past 4 weeks (including today):

1.	I have had itching		My itching was:	
2.	I have had chest pain		My chest pain was:	
3.	I have had wind		My wind was:	
4.	I have had increased thirst		My increased thirst was:	
5.	I have felt restless or nervous		My restlessness or nervousness was:	
6.	I have had hearing loss		My hearing loss was:	
7.	I have had an abnormal skin color		My abnormal skin color was:	
8.	I have had increased sweating		My increased sweating was:	
9.	My face and neck have been red		The redness in my face and neck was:	
10.	I have had brittle fingernails		My brittle fingernails were:	
11.	My breasts habe been larger		My breast enlargement was:	
12.	I have had sores on my lips and/or in my mouth		My sores on lips and/or in mouth were:	
13.	I have had an altered voice		My altered voice was:	
14.	I have had oily skin		My oily skin was:	
15.	I have felt dizzy		My dizziness was:	
16.	My hands have trembled		My trembling hands were:	
17.	I have had an increased urge to urinate		My increased urge to urinate was:	
18.	I have had a feeling of warmth in my hands and feet		The feeling of warmth in my hands and feet was:	
19.	I have had bruises more easily		My bruises were:	
20.	I have had sores or warts around my genitals		My sores or warts around genitals were:	
21.	I have had spots on my face and/or my back		My spots on my face and/or back were:	

**22.**	**I have had an excessive appetite**	**My excessive appetite was:**	
**23.**	**I have felt depressed**	**My feelings of depression were:**	
**24.**	**My gums have swollen**	**My swollen gums were:**	
**25.**	**I have had swollen glands in my neck, armpit or groin**	**My swollen glands were:**	
**26.**	**I have had thinning of hair or hair loss**	**My hair thinning or hair loss was:**	
**27a.**	**I have had menstrual problems** ***(for females only)***	**My menstual problems were:**	
**27b.**	**I have had erectile problems** ***(for males only)***	**My erectile problems were:**	
**28.**	**I have had a puffy face (moon face)**	**My puffy face was:**	
**29.**	**I have had swollen ankles or feet**	**My swollen ankles or feet were:**	

☞ ***To continue, please go to the next page.***

f:\institut\cultadap\project\bms2259\etude2259\finalversions\original\mtsosd-f-orig.doc-11/03/2005

Have any protocol or GCP deviations occurred on this eForm?

◎ **no**

◎ **yes**

If yes, please document all protocol or GCP deviations for this eForm here (max. 3 entries):

**Description:**	**(Possible) Reason:**
...	...

TTV GUIDE

Sponsor Code: TTV GUIDE II
EudraCT-Number: 2021-002525-24

Visit:

Site-No.: Patient-No.: Arm: Database:

Only applicable in eCRF!

MTSOSD

Modified Transplant Symptom Occurrence and Symptom Distress Scale

During the past 4 weeks (including today):

30.	I have had diarrhea		My diarrhea was:	
31.	I have had tingling or numbness in my hands or feet		My tingling or numbness in my hands or feet was:	
32.	I have had back pain		My back pain was:	
33.	I have had a brittle skin		My brittle skin was:	
34.	I have felt anxious		My feelings of anxiety were:	
35.	I have been experiencing mood swings		My mood swings were:	
36.	I have had headaches		My headaches were:	
37.	My facial features have changed		My changed facial features were:	
38.	I have had fat deposits on my neck and back ("buffalo hump")		My fat deposits on neck and back were:	
39.	I have had difficulty concentrating and/or memory problems		My concentration difficulties and/or memory problems were:	
40.	I have had warts on hands and feet		My warts on hands and feet were:	
41.	I have had increased hair growth on face and body		My increased hair growth on face and body were:	
42.	I have had sleep difficulties		My sleep difficulties were:	
43.	I have had muscle weakness		My muscle weakness was:	
44.	My sense of taste has changed		The change in my sense of taste was:	
45.	I have had a poor appetite		My poor appetite was:	
46.	I have felt tired		My tiredness was:	
47.	I have had lack of energy		My lack of energy was:	
48.	I have had stomach complaints, I have felt nauseous and/or I had to vomit		My stomach complaints, nausea or vomiting were:	
49.	I have had pain in my joints		My joint pain was:	
50.	I have had a rash on my skin		My skin rash was:	
51.	I have had muscle cramps		My muscle cramps were:	
52.	I have had nightmares		My nightmares were:	
53.	I have been short of breath		My shortness of breath was:	
54.	I have had a dry skin		My dry skin was:	

**55.**	**I have had palpitations**	**My palpitations were:**	
**56.**	**I have had constipation**	**My constipation was:**	
**57.**	**I have had difficulty seeing well**	**My seeing difficulties were:**	
**58.**	**I have had a reduced interest in sex**	**My reduced interest in sex was:**	
**59.**	**My eyes have been sensitive to light**	**My sensitivity to light was:**	

☞ ***Please check if you have completed all items and all pages.***
Please also be sure that you have completed both the right and left column of the questionnaire.

f:\institut\cultadap\project\bms2259\etude2259\finalversions\original\mtsosd-f-orig.doc-11/03/2005

Have any protocol or GCP deviations occurred on this eForm?

◎ **no**

◎ **yes**

If yes, please document all protocol or GCP deviations for this eForm here (max. 3 entries):

**Description:**	**(Possible) Reason:**
...	...

Sponsor Code: TTV GUIDE IT
EudraCT-Number: 2021-002525-24

Visit:

Site-No.: Patient-No.: Arm: Database: *Only applicable in eCRF!*

Sampling of additional Biological Material

Have the additional biological samples been collected?

☞ If no, reason:

◎ no
◎ yes

☞ If yes, Date:
dd/mm/yyyy

Have any protocol or GCP deviations occurred on this eForm?

◎ no
◎ yes

If yes, please document all protocol or GCP deviations for this eForm here (max. 3 entries):

Description:	(Possible) Reason:
...	...

Sponsor Code: TTV GUIDE IT
EudraCT-Number: 2021-002525-24

Visit:

Site-No.: Patient-No.: Arm: Database: *Only applicable in eCRF!*

Protocol biopsy

Has a protocol biopsy been done?

The protocol biopsy is an examination within the framework of clinical routine.

◎ no
◎ yes

If yes, date: dd/mm/yyyy

Diagnosis / Medical Term:

Date of KTX: dd/mm/yyyy *automatic entry*

Donor-specific antibodies currently detectable:

◎ no
◎ yes

If yes, please specify:

BKV PCR: ***(blood)***

BKV (blood) unit: IU/ml, Copies/µl

◎ IU/ml
◎ Copies/µl

Has material been shipped for Molecular Microscope Diagnostic for Kidney (MMDx-Kidney)?

◎ no
◎ yes

If yes, date: dd/mm/yyyy

SV40 stain:

BANFF Classification:

based on the Banff 2019 Kidney Meeting Report

Normal biopsy or nonspecific changes:

◎ no
◎ yes

Active antibody-mediated rejection:

◎ no
◎ yes

Chronic active antibody-mediated rejection:

◎ no
◎ yes

Chronic (inactive) antibody-mediated rejection:

◎ no
◎ yes

C4d staining without evidence of rejection:

◎ no
◎ yes

Borderline (suspicious) for acute T-cell-mediated rejection:

◎ no
◎ yes

Acute T-cell-mediated rejection:

Chronic active T-cell-mediated rejection:

Polyomavirus nephropathy:

BANFF single scores:

i: **t:** **v:** **g:** **ptc:** **C4d:**

ci: **ct:** **cv:** **cg:** **ptcml:**

ti: **i-IFTA:** **t-IFTA:** **pvl:**

Have any protocol or GCP deviations occurred on this eForm?

◎ no

◎ yes

If yes, please document all protocol or GCP deviations for this eForm here (max. 3 entries):

Description: ...

(Possible) Reason: ...

Sponsor Code: TTV GUIDE IT
EudraCT-Number: 2021-002525-24

Visit:

Site-No.: Patient-No.: Arm: Database: *Only applicable in eCRF!*

End of intervention

Please specify the reason for end of intervention:

◎ Regular end of intervention
◎ Patient wants no further study participation
◎ Investigator decision that it is not in the best interest of the subject to continue
◎ Graft rejection
◎ Graft loss
◎ Severe infection (life or organ threatening defined by investigator)
◎ Other (Serious) Adverse event that requires the termination of the trial treatment
◎ Pregnancy
◎ Non-adherence with the trial treatment
◎ Introduction of mTOR inhibitors, co-stimulation blockers or cyclosporine
◎ Necessity of significant additional long term immunosuppression or immune modulation
◎ Any condition that needs significant higher/lower than usual TAC target range
◎ Necessity of long term stopping of TAC
◎ HIV infection
◎ Cancer
◎ Death
◎ Other reasons

Date End of intervention: dd/mm/yyyy

If the reason for termination is graft rejection, severe infection, or (serious) adverse event, please provide the corresponding eCRF number.

(S)AE No.:

Specify other reason:

In case of end of intervention due to death, please also complete the eform "End of study".

In case of end of intervention due to graft loss, please complete the eforms "Graft loss".

Go to eForm "Graft loss"

Go to eForm "End of study"

Have any protocol or GCP deviations occurred on this eForm?

◎ no
◎ yes

If yes, please document all protocol or GCP deviations for this eForm here (max. 3 entries):

Description: **(Possible) Reason:**

Sponsor Code: TTV GUIDE IT
EudraCT-Number: 2021-002525-24

Visit:

Site-No.: Patient-No.: Arm: Database: *Only applicable in eCRF!*

Graft loss

Date of graft loss: dd/mm/yyyy **days after transplantation** *automatic entry*

☞ **Primary reason:**
- ◎ acute graft rejection
- ◎ chronic graft rejection
- ◎ Recurrence of underlying disease
- ◎ technical complications
- ◎ BKV Nephropathy
- ◎ Other

☞ **If other, please specify:**

Have any protocol or GCP deviations occurred on this eForm?

- ◎ no
- ◎ yes

If yes, please document all protocol or GCP deviations for this eForm here (max. 3 entries):

Description: ... **(Possible) Reason:** ...

Sponsor Code: TTV GUIDE IT
EudraCT-Number: 2021-002525-24

Visit:

Site-No.: **Patient-No.:** **Arm:** **Database:** *Only applicable in eCRF!*

Adverse events

(Further) Adverse event (AE) occurred?

◎ no
◎ yes ☞ **If yes, please provide further information.**

Please document all adverse events that have been observed from visit 1 to visit 7.

No. eCRF:
(automatic entry)

AE categorisation:

◎ Infectious disease*
◎ Graft rejection**
◎ Other Adverse Event

☞ ****In the case of an infectious disease please document the additional eForms 'Infection diseases part 1' to 'Infection diseases part 3'.***
*****In case of graft rejection, please document the additional eForm 'Graft rejection'.***

Medical term / CTCAE term:

Abbreviations are not allowed!

Start:
dd/mm/yyyy

Outcome:

◎ recovered/resolved
◎ recovering/resolving
◎ not recovered/not resolved
◎ recovered/resolved with sequelae
◎ fatal
◎ unknown

☞ **End of adverse event:**
(when recovered/resolved or death)
dd/mm/yyyy

☞ **Does the adverse event is ongoing beyond the end of study?**
(when recovering/resolving or not recovered/not resolved)

◎ no
◎ yes

CTCAE-Grade (1-5):
CTCAE 5.0 – English Version, November 2017, National Cancer Institute

Inpatient or daycare treatment?

◎ no
◎ inpatient treatment
◎ daycare treatment
◎ unknown

☞ **from:** dd/mm/yyyy **to:** dd/mm/yyyy

Serious?

◎ no
◎ yes

☞ ***If this is a serious adverse event or inpatient treatment is necessary, please fill in the eCRF Page "SAE Report" immediately within 24 hours.***

Causality:

◎ certain
◎ probable, likely
◎ possible
◎ unlikely
◎ not related
◎ not assessable, unclassificable

Action taken regarding with Tacrolimus:

◎ **dose not changed**
◎ **dose reduced**
◎ **dose increased**
◎ **drug interrupted**
◎ **drug withdrawn**
◎ **unknown**
◎ **not applicable**

Has this AE been treated?

◎ **no**
◎ **yes**

☞ ***If yes, please document all necessary therapies for the treatment of Adverse Events at eCRF section 'Concomitant medication'.***

Go to eCRF section 'Concomitant medication'

Have any protocol or GCP deviations occurred on this eForm?

◎ **no**
◎ **yes**

If yes, please document all protocol or GCP deviations for this eForm here (max. 3 entries):

**Description:**	**(Possible) Reason:**
...	...

Sponsor Code: TTV GUIDE II
EudraCT-Number: 2021-002525-24

Visit:

Site-No.: Patient-No.: Arm: Database: *Only applicable in eCRF!*

Infectious diseases part 1 - Symptoms

No. eCRF:
automatic entry

Description of corresponding AE (see eCRF page Adverse event)

General symptoms:	Gastrointestinal tract symptoms:	Renal and urinary tract symtoms:	Respiratory tract symtoms:
Pain: ◎ Pain	*Diarrhea:* ◎ Diarrhea	*Dysuria:* ◎ Dysuria	*Cough* ◎ Cough
Night sweat: ◎ Night sweat	*Abdominal pain/cramps:* ◎ Abdominal pain/cramps	*Pollakisuria:* ◎ Pollakisuria	*Sputum (purulent, with blood):* ◎ Sputum (purulent, with blood)
Fever: ◎ Fever		*Alguria:* ◎ Alguria	☞ *Sputum purulent:* ◎ purulent
Chills: ◎ Chills		*Urinary urgency/frequency:* ◎ Urinary urgency/frequency	*Sputum with blood:* ◎ with blood
Malaise: ◎ Malaise		*Suprapubic pain:* ◎ Suprapubic pain	*Adventitious breath sounds on auscultation/palpation:* ◎ Adventitious breath sounds on auscultation/palpation
Fatigue: ◎ Fatigue		*Flank/allograft pain (on palpitation):* ◎ Flank/allograft pain (on palpitation)	*Shortness of breath:* ◎ Shortness of breath
			Rapid/shallow breathing: ◎ Rapid/shallow breathing

If other symptoms have occurred, please specify:

☞ **If diarrhea is a symptom, please indicate the stool frequency:** ____ **during the day**

☞ **If diarrhea is a symptom, please indicate the consistency of stool:**

Vital signs

Blood pressure: (mmHg) ____ / ____
systolic *diastolic*

Pulse: (1/min)

Respiratory rate: (1/min)

Temperature: (°C)

Altered Mental status:
◎ no
◎ yes

qSOFA-Score: *automatic entry*

Treatment

Has this infectious disease been treated?

◎ **no**
◎ **yes**

☞ **If yes, please specify:**
(multiple choice possible)

anti-bacterial treatment:
◎ **antibiotics**
anti-viral treatment:
◎ **virustatics**
anti-fungal treatment:
◎ **antimycotics**
anti-protozoal treatment:
◎ **anti-protozoal**
other treatment:
◎ **other**

☞ ***Please also document the exact medication used to treat this infectious disease in the eCRF section "Concomitant medication".***

Go to eCRF section 'Concomitant medication'

Have any protocol or GCP deviations occurred on this eForm?

◎ **no**
◎ **yes**

If yes, please document all protocol or GCP deviations for this eForm here (max. 3 entries):

**Description:**	**(Possible) Reason:**
...	...

Sponsor Code: TTV GUIDE IT
EudraCT-Number: 2021-002525-24

Visit:

Site-No.: Patient-No.: Arm: Database: *Only applicable in eCRF!*

Infectious diseases part 2 - Diagnostics

Was laboratory test done at the start of this infection?

◎ no → **If no, reason:**

◎ yes → **If yes, date:** dd/mm/yyyy

Parameter:	Value:	Unit:	Other unit:
Leukocytes stand.: **Leukocytes:**		*Leukocytes unit: Gpt/l (10^9/l), /µl, /nl (10^3/µl, 1000/µl)*	*Leukocytes other unit:*
Neutrophiles stand.: **Neutrophiles:**		*Neutrophiles unit: Gpt/l (10^9/l), /µl, /nl, 1000/µl (10^3/µl), %, ratio*	*Neutrophiles other unit:*
Lymphocytes stand.: **Lymphocytes:**		*Lymphocytes unit: Gpt/l (10^9/l), /µl, /nl, 1000/µl (10^3/µl), %, ratio*	*Lymphocytes other unit:*
Erythrocytes stand.: **Erythrocytes:**		*Erythrocytes unit: Tpt/l (10^6/µl), /pl*	*Erythrocytes other unit:*
Haemoglobin stand.: **Haemoglobin:**		*Haemoglobin unit: g/l, g/dl, mg/dl, mmol/l*	*Haemoglobin other unit:*
Haematocrit stand.: **Haematocrit:**		*Haematocrit unit: %, ratio, l/l*	*Haematocrit other unit:*
Platelets stand.: **Platelets:**		*Platelets unit: Gpt/l (10^9/l), /nl, 1000/µl (10^3/µl), /µl*	*Platelets other unit:*
CRP stand.: **CRP:**		*CRP unit: g/l, g/dl, mg/l, mg/dl, µg/dl*	*CRP other unit:*
Procalcitonine stand.: **Procalcitonine:**		*Procalcitonine unit: µg/l, ng/l, ng/dl, ng/ml, ng%*	*Procalcitonine other unit:*
Interleukin-6 stand.: **Interleukin-6:**		*Interleukin-6 unit: pg/ml, pg/dl, pg/l, pg%, ng/l*	*Interleukin-6 other unit:*
Creatinine stand.: **Creatinine:**		*Creatinine unit: g/l, g/dl, mg/dl, mmol/l, µmol/l*	*Creatinine other unit:*
BUN stand.: **Blood Urea nitrogen:**		*Blood Urea nitrogen unit: mg/dl, µg/ml, mmol/l, µmol/l*	*Blood Urea nitrogen other unit:*
Lactat stand.: **Lactat:**		*Lactat unit: mmol/l, µmol/l, mg/dl, mg/l, µg/ml, mg%*	*Lactat other unit:*

Albumin stand.: [ ]

Albumin: [ ]

Albumin unit:
g/l, g/dl, mg/dl, g%, mmol/l, µmol/l

Albumin
other unit:

Blood collection:
arterial, venous

pH Value: ◎ **arterial** ◎ **venous** [ ]

☞ ***If further tests were performed, please document these on the next page.***

Have any protocol or GCP deviations occurred on this eForm?

◎ **no**

◎ **yes**

If yes, please document all protocol or GCP deviations for this eForm here (max. 3 entries):

**Description:**	**(Possible) Reason:**

Sponsor Code: TTV GUIDE II
EudraCT-Number: 2021-002525-24

Visit:

Site-No.: Patient-No.: Arm: Database:

Only applicable in eCRF!

Infectious diseases part 3 - Further diagnostics

Was a blood culture performed?

◎ no
◎ yes

If yes, date: dd/mm/yyyy

Result:

If positive, please specify pathogen(s):

Was a Legionella pneumophilia urinary antigen determination performed?

◎ no
◎ yes

If yes, date: dd/mm/yyyy

Result:

Was a Pneumococcus urinary antigen determination performed?

◎ no
◎ yes

If yes, date: dd/mm/yyyy

Result:

ation performed?

◎ no
◎ yes

If yes, date: dd/mm/yyyy

Result:

Was a urinary dip stick performed?

◎ no
◎ yes

If yes, date: dd/mm/yyyy

Interpretation: ◎ normal ◎ abnormal

Nitrite:

Leukocytes:

Erythrocytes:

If other findings, please specify:

Was a urine culture performed?

◎ no
◎ yes

If yes, date: dd/mm/yyyy

Result:

If positive, please specify pathogen(s):

Was a stool culture performed?

◎ no
◎ yes ☞ **If yes, date:** dd/mm/yyyy

Result:

☞ **If positive, please specify pathogen(s):**

Was a stool multiplex PCR done?

◎ no
◎ yes ☞ **If yes, date:** dd/mm/yyyy

Result:

☞ **If positive, please specify pathogen(s):**

Was a sputum or nasopharyngeal swab multiplex PCR done?

◎ no
◎ yes ☞ **If yes, date:** dd/mm/yyyy

Result:

☞ **If positive, please specify pathogen(s):**

Was a CMV test been done?

◎ no
◎ yes ☞ **If yes, date:** dd/mm/yyyy

Table: CMV test, max. 5 rows

Material:	If other material, please specify:	Result:	Unit:	If other unit, please specify:	New row?
	...			...	
	...			...	
	...			...	
	...			...	
	...			...	

Was a BKV test been done?

◎ no
◎ yes ☞ **If yes, date:** dd/mm/yyyy

Table: BKV test, max. 5 rows

Material:	If other material, please specify:	Result:	Unit:	If other unit, please specify:	New row?
	...			...	
	...			...	
	...			...	
	...			...	
	...			...	

Was a imaging done?

◎ no

◎ yes

Table: Imaging, max. 5 rows

Date: dd/mm/yyyy	Imaging modality:	Localisation:	Please enter the exact findings: (max. 200 characters)	New row?

Other relevant findings and examinations:

Table: Relevant lab findings, max. 5 rows

Date: dd/mm/yyyy	Laboratory parameter:	Material:	Examination method:	Result:

Have any protocol or GCP deviations occurred on this eForm?

◎ no

◎ yes

If yes, please document all protocol or GCP deviations for this eForm here (max. 3 entries):

Description:	(Possible) Reason:

Sponsor Code: TTV GUIDE II
EudraCT-Number: 2021-002525-24

Visit:

Site-No.: Patient-No.: Arm: Database: *Only applicable in eCRF!*

Graft rejection

No. eCRF:
automatic entry

Description of corresponding AE (see eCRF page Adverse event)

Was the graft rejection biopsy secured?

◎ **no**
◎ **yes** ☞ **If yes, date of biopsy:** dd/mm/yyyy

Date of KTX: ***automatic entry***
dd/mm/yyyy

Donor-specific antibodies currently detectable:

◎ **no**
◎ **yes** ☞ **If yes, please specify:**

BKV PCR: ***(blood)***

BKV (blood) unit: IU/ml, Copies/µl
◎ **IU/ml**
◎ **Copies/µl**

SV40 stain:

BANFF Classification:

based on the Banff 2019 Kidney Meeting Report

Normal biopsy or nonspecific changes:
◎ **no**
◎ **yes**

Active antibody-mediated rejection:
◎ **no**
◎ **yes**

Chronic active antibody-mediated rejection:
◎ **no**
◎ **yes**

Chronic (inactive) antibody-mediated rejection:
◎ **no**
◎ **yes**

C4d staining without evidence of rejection:
◎ **no**
◎ **yes**

Borderline (suspicious) for acute T-cell-mediated rejection:
◎ **no**
◎ **yes**

Acute T-cell-mediated rejection:

Chronic active T-cell-mediated rejection:

Polyomavirus nephropathy:

BANFF single scores:

i: **t:** **v:** **g:** **ptc:** **C4d:**

ci: **ct:** **cv:** **cg:** **ptcml:**

ti: **i-IFTA:** **t-IFTA:** **pvl:**

Have any protocol or GCP deviations occurred on this eForm?

◎ no

◎ yes

If yes, please document all protocol or GCP deviations for this eForm here (max. 3 entries):

Description: **(Possible) Reason:**

EudraCT-Number: 2021-002525-24

Visit:

Site-No.: Patient-No.: Arm: Database: *Only applicable in eCRF!*

SAE report

(Further) Serious adverse event (SAE) occurred?

◎ no
◎ yes ☞ If yes, please provide further information.

All fields marked with a (!) are mandatory fields and must always be filled in.
This color indicates automatic entry fields. Please complete the appropriate eCRF page first to transfer for SAE reporting.

SAE eCRF No.: *automatic entry*

(!) Diagnosis, relevant symptoms:

Abbreviations are not allowed!

Corresponding AE No.: ***Please transfer the eCRF number of the corresponding adverse event from the label of the respective eCRF page.***

Description of corresponding AE (see eCRF page Adverse event):

Date of awareness: dd/mm/yyyy

SAE Criterion or reason for reporting:

◎ results in death
◎ life-threatening
◎ requires inpatient hospitalization or prolongation
◎ other medically important condition
◎ results in persistent or significant disability/ incapacity
◎ congenital anomaly/ birth defect

(!) Start: dd/mm/yyyy

Date of resolution or date of death: dd/mm/yyyy

Outcome of the SAE:

◎ recovered/resolved
◎ recovering/resolving
◎ not recovered/not resolved
◎ recovered/resolved with sequelae
◎ fatal
◎ unknown

Primary cause of death (see eCRF page End of study):

Age at time of onset of SAE: (years) *automatic entry*

Weight: (kg)

(!) Causality of the SAE:

◎ certain
◎ probable, likely
◎ possible
◎ unlikely
◎ not related
◎ not assessable, unclassificable

CTCAE grade (1-5):
CTCAE 5.0 – English Version, November 2017, National Cancer Institute

Did the SAE lead to unblinding of the patient?

◎ no
◎ yes

Have further therapeutic measures for the treatment of SAE been carried out?

◎ no
◎ yes

Medication name and dosage:

Date of 1st administration:
(see eCRF page Tacrolimus)
dd/mm/yyyy

Batch No.:

Date of last administration before onset of SAE:
dd/mm/yyyy

Daily dose (mg):

Frequency:

Action taken regarding with Tacrolimus:

◎ dose not changed
◎ dose reduced
◎ dose increased
◎ drug interrupted
◎ drug withdrawn
◎ unknown
◎ not applicable

Date of treatment interruption or discontinuation:
dd/mm/yyyy

Did the reaction recur when therapy was resumed?

◎ no
◎ yes

Relevant concomitant treatment

Please transfer here the eCRF number from the label of the respective eCRF page Concomitant treatment.

Table: Relevant concomitant treatment, max. 4 rows

eCRF No.:*	Description:	Daily dose/ Applications per day:	Unit:
	...		
	...		
	...		
	...		

Relevant pre-existing, concomitant diseases

Please transfer here the eCRF number from the label of the respective eCRF page Pre-existing, concomitant disease.

Table: Relevant pre-existing, concomitant diseases, max. 3 rows

eCRF No.:*	Description:

Relevant laboratory findings or investigations

Table: Relevant laboratory findings or investigations, max. 3 rows

Description, reference range, result:	Date (dd/mm/yyyy):

Comment:

SAE reporting

Please fill in all mandatory fields (!) before reporting of this event.
Reporting only by the investigator.
A second SAE reporting with the same report number is not possible.

Please note: If the e-mail with the SAE report has not been received after approx. 15 min, please send a feedback via e-mail to: sandra.koenig@ukdd.de

Table: SAE reporting, max. 5 rows

To report the SAE, please klick here and save the eCRF page!	SAE report No.:	Date of report (automatic entry):	Name of reporter (automatic entry):	Manual entry of report date by Data Management KKS Dresden
◎ SAE reporting				
◎ SAE reporting				
◎ SAE reporting				
◎ SAE reporting				
◎ SAE reporting				

TTV GUIDE

Sponsor Code: TTV GUIDE II
EudraCT-Number: 2021-002525-24

Visit:

Site-No.: Patient-No.: Arm: Database:

Only applicable in eCRF!

Concomitant medication

(Further) concomitant medication?

◎ no
◎ yes
◎ unknown

Please document all medications administered since the start of the study at visit 1.
For each change: Documentation in a new line or form.

In addition to the daily dose, please document the unit, frequency and application.
For combination products: one active substance per line / form.

No. eCRF:
automatic entry

Active ingredient / Trade name:

Indication of concomitant medication:

****Please transfer the eCRF number from the label of the respective eCRF page of the concomitant disease/adverse event.***

Table: Indication, max. 3 rows

administred due to:	eCRF No. of concomitant disease/ adverse event:*	Description concomitant disease/ adverse event: (automatic entry)	Period:

Start prior start of study?

◎ no
◎ yes

Start: dd/mm/yyyy

End: dd/mm/yyyy

End:
◎ **ongoing after end of study**

Daily dose:

Unit:

Frequency:

Application:

Have any protocol or GCP deviations occurred on this eForm?

◎ no
◎ yes

If yes, please document all protocol or GCP deviations for this eForm here (max. 3 entries):

Description:	(Possible) Reason:

Sponsor Code: TTV GUIDE IT
EudraCT-Number: 2021-002525-24

Visit:

Site-No.: Patient-No.: Arm: Database: *Only applicable in eCRF!*

Vaccinations

(Further) Vaccination?

◎ no
◎ yes
◎ unknown

Please document all vaccinations administered since the start of the study at visit 1 with date, indication and vaccine used.
For each indication of vaccination use a new form.

For COVID-19: Please also document all vaccinations before the start of the study.

For influenza: If received, please also document the vaccination of the previous year.

No. eCRF:

automatic entry

Indication:

If other indication, please specify:

Date of vaccination:

dd/mm/yyyy

Vaccine used:

Have any protocol or GCP deviations occurred on this eForm?

◎ no
◎ yes

If yes, please document all protocol or GCP deviations for this eForm here (max. 3 entries):

Description: **(Possible) Reason:**

Sponsor Code: TTV GUIDE IT
EudraCT-Number: 2021-002525-24

Site-No.: Patient- No.: Visit: Database:

Only applicable in eCRF!

Central assessement of infectious diseases

Corresponding AE No.: ☞ ***Please transfere the number of corresponding adverse event from the respective e-mail.***

Description of corresponding Infection disease:

Is this infectious disease confirmed?

◎ **no**
◎ **yes** ☞ **If no, other diagnosis:**

Comments:

Sponsor Code: TTV GUIDE II
EudraCT-Number: 2021-002525-24

Visit:

Site-No.: **Patient- No.:** **Database:** *Only applicable in eCRF!*

Data clarification

Please note: create the query for an infectious disease first and then click on "send e-mail" and save the eForm.

For a query on another infectious disease, please enter the corresponding AE-No. in the next line.

****Please transfere the number of corresponding adverse event from the label of the respective eCRF page.***

Table: Data clarification infectious diseases, max. 15 rows

***Corresponding AE No.:**	**Infectious disease term:**		**To send an e-Mail for this query, please click here and save the eCRF page!**	DC report no.:
		...	◎ **send e-mail**	
		...	◎ **send e-mail**	
		...	◎ **send e-mail**	
		...	◎ **send e-mail**	
		...	◎ **send e-mail**	
		...	◎ **send e-mail**	
		...	◎ **send e-mail**	
		...	◎ **send e-mail**	
		...	◎ **send e-mail**	
		...	◎ **send e-mail**	
		...	◎ **send e-mail**	
		...	◎ **send e-mail**	
		...	◎ **send e-mail**	
		...	◎ **send e-mail**	
		...	◎ **send e-mail**	

Sponsor Code: TTV GUIDE IT
EudraCT-Number: 2021-002525-24

Visit:

Site-No.: Patient- No.: Database: *Only applicable in eCRF!*

Central assessement of graft rejection

Corresponding AE No.: ☞ ***Please transfere the number of corresponding adverse event from the respective e-mail.***

Date of biopsy: ***automatic entry***
dd/mm/yyyy

Date of KTX: ***automatic entry***
dd/mm/yyyy

Donor-specific antibodies: ***automatic entry***

BKV PCR: ***automatic entry***

Has kidney biopsy material been received?

◎ no
◎ yes ☞ **If yes, date of receipt:**
dd/mm/yyyy

Has a graft rejection been confirmed?

☞ **If no, other diagnosis:**
◎ no
◎ yes ☞ **If yes, please provide further informations**

SV40 stain:

Arteries:

Glomeruli:

Scarred glomerulus:

BANFF Classification

based on the Banff 2019 Kidney Meeting Report

Normal biopsy or nonspecific changes:
◎ no
◎ yes

Active antibody-mediated rejection:
◎ no
◎ yes

Chronic active antibody-mediated rejection:
◎ no
◎ yes

Chronic (inactive) antibody-mediated rejection:
◎ no
◎ yes

C4d staining without evidence of rejection:
◎ no
◎ yes

Borderline (suspicious) for acute T-cell-mediated rejection:
◎ no
◎ yes

Acute T-cell-mediated rejection: [ ]

Chronic active T-cell-mediated rejection: [ ]

Polyomavirus nephropathy: [ ]

BANFF single scores:

i: [ ] **t:** [ ] **v:** [ ] **g:** [ ] **ptc:** [ ] **C4d:** [ ]

ci: [ ] **ct:** [ ] **cv:** [ ] **cg:** [ ] **ptcml:** [ ]

ti: [ ] **i-IFTA:** [ ] **t-IFTA:** [ ] **pvl:** [ ]

Comments:

---

eForm label:

Sponsor Code: TTV GUIDE IT
EudraCT-Number: 2021-002525-24

Site-No.: **Patient- No.:** Visit: **Database:** *Only applicable in eCRF!*

Data clarification

Please note: create the query for a graft rejection first an then click on "send e-mail" and save the eForm.

For a query on another graft rejection, please enter the corresponding AE-No. in the next line.

****Please transfere the number of corresponding adverse event from the label of the respective eCRF page.***

Table: Data clarification graft rejection, max. 15 rows

***Corresponding AE No.:**	**Graft rejection term:**	**To send an e-mail for this query, please click here and save the eCRF page!**	DC report no.:
	...	◎ **send e-mail**	
	...	◎ **send e-mail**	
	...	◎ **send e-mail**	
	...	◎ **send e-mail**	
	...	◎ **send e-mail**	
	...	◎ **send e-mail**	
	...	◎ **send e-mail**	
	...	◎ **send e-mail**	
	...	◎ **send e-mail**	
	...	◎ **send e-mail**	
	...	◎ **send e-mail**	
	...	◎ **send e-mail**	
	...	◎ **send e-mail**	
	...	◎ **send e-mail**	
	...	◎ **send e-mail**	

TTV GUIDE

Sponsor Code: TTV GUIDE IT
EudraCT-Number: 2021-002525-24

Site-No.: **Patient- No.:** **Arm:** Visit: **Database:** *Only applicable in eCRF!*

Potential Serious Breach - Site report

Report a (further) potential serious breach?

◎ no
◎ yes ☞ **If yes, please provide further information.**

Definition of Serious breach:
A "serious breach" means any deviation or the Regulation (EU) 536/2014 or of the approved version of the protocol applicable that is likely to affect to a significant degree

(A) the safety and rights of a subject and/or
(B) the reliability and robustness of the data generated in the clinical trial

eCRF No.:
automatic entry

Date of serious breach:
dd/mm/yyyy

Visit where the serious breach occurred:

Description of the serious breach:

(Possible) Reason for the serious breach:

Action taken by the reporting site to investigate and correct the serious breach?

◎ no
◎ yes

☞ **If yes, short description:**

Were preventive action taken?

◎ no
◎ yes

☞ **If yes, short description:**

Serious breach reporting:

Table: Serious breach reporting, max. 3 rows

To report the serious breach, please click here and save the eCRF page!	Serious breach report No.:	Date or report: (automatic entry)	Name of reporter: (automatic entry)
◎ **Serious breach reporting**			
◎ **Serious breach reporting**			
◎ **Serious breach reporting**			

Sponsor Code: TTV GUIDE IT
EudraCT-Number: 2021-002525-24

Visit:

Site-No.: Patient-No.: Arm: Database: *Only applicable in eCRF!*

End of study

Please specify the end of study:

- ◎ Regular end of study
- ◎ Screening failure
- ◎ Patient wants no further study participation
- ◎ Withdrawal, because patient is not available
- ◎ Withdrawal of study because of death
- ◎ Withdrawal of study due to other reasons

☞ **If other reasons, please specify:**

Date of last contact: dd/mm/yyyy

☞ **Date of death:** dd/mm/yyyy

Primary cause of death:

Secondary cause of death:

In case of End of study due to graft loss, please also complete the eform "Graft loss".

Go to eForm "Graft loss"

Have any protocol or GCP deviations occurred on this eForm?

- ◎ no
- ◎ yes

If yes, please document all protocol or GCP deviations for this eForm here (max. 3 entries):

Description: ...

(Possible) Reason: ...

Sponsor Code: TTV GUIDE II
EudraCT-Number: 2021-002525-24

Visit:

Site-No.: **Patient-No.:** **Arm:** **Database:** *Only applicable in eCRF!*

Signature Investigator

I confirm that all information provided are correct and complete.

Date:
dd/mm/yyyy

Name:
(After entering the date, the name is automatically adopted)

Sponsor Code: TTV GUIDE TT
EudraCT-Number: 2021-002525-24

Visit:

Site-No.: Patient-No.: Arm: Database: *Only applicable in eCRF!*

Signature Principal Investigator

I confirm that all information provided are correct and complete.

Date:
dd/mm/yyyy

Name:

TTV GUIDE

Sponsor Code: TTV GUIDE IT
EudraCT-Number: 2021-002525-24

Visit:

Site-No.: Patient-No.: Arm: Database:

Only applicable in eCRF!

Database training

Please complete all active fields in this eForm and save this eForm afterwards.
An automatic e-mail will be sent to the data management and your data will be processed.

Function (role):

◎ Investigator (INV)	*INV is authorized for: enter and change data, respond to queries, electronic signature*
◎ Data Entry Staff (DE)	*DE is authorized for: enter and change data, respond to queries*
◎ Monitor (MO)	*MO is authorized for: read data, create and close queries, create SDV marks, freeze data*
◎ Virology lab (DErestricted2)	*Virology lab is authorized for: enter and change data on the eForm "TTV analysis"*
◎ Central infectiologist (DErestrictedDC)	*Central infectiologist is authorized for: enter and change data on the eForm "Central assessement infectious diseases" and create and close queries on the eForm "Data clarification infectious diseases"*
◎ Central pathologist (DErestrictedDC2)	*Central pathologist is authorized for: enter and change data on the eForm "Central assessement graft rejection" and create and close queries on the eForm "Data clarification graft rejection"*

Training contents for role Investigators (INV) and Data Entry Staff (DE):

1. ◎ Registration of a new patient and enter the data for informed consent
2. ◎ Entry of inclusion and exclusion criteria, no enrolment into trial - Screening failure
3. ◎ Entry of inclusion and exclusion criteria, enrolment into trial
4. ◎ Randomisation
5. ◎ Entry of data in the eForm "Intervention"
6. ◎ Transferring entries form the patient diary on the eForms "Drug account" and "Tacrolimus"
7. ◎ Entry of data in the eForms "Infectious diseases Part 1 - Part 3", "Graft rejection" and "Graft loss"
8. ◎ SAE reporting
9. ◎ electronic signature on the eForm "Signature Investigator"

Training contents for role Monitors (MO):

1. ◎ Create and close queries
2. ◎ Create SDV marks
3. ◎ Freeze data

Training content for virology lab (DErestricted2):

1. ◎ Data entry on the eForm "TTV analysis"

Training content for central infectiologist (DErestrictedDC):

1. ◎ Data entry on the eForm "Central assessement infectious diseases"
2. ◎ Create and close queries on the eForm "Data clarification infectious diseases"

Training content for central pathologist (DErestrictedDC2):

1. ◎ Data entry on the eForm "Central assessement graft rejection"
2. ◎ Create and close queries on the eForm "Data clarification graft rejection"

With my signature I confirm that I have received training in the use of the study-specific database from qualified personnel or that I have familiarised myself with the database.
I agree to keep my password secret and to adhere to data protection.

With my signature I also agree to the use and storage of my personal data in the context of database access.
I have read the privacy policy (see link in login window).

User name: ____

Date: ____ *(automatic entry)*
dd/mm/yyyy
