# Supplementary material for: A multicentre, patient- and assessor-blinded, non-inferiority, randomised and controlled phase II trial to compare standard and torque teno virus-guided immunosuppression in kidney transplant recipients in the first year after transplantation: TTVguideIT
Source: Trials. 2023 Mar 22;24:213. doi: 10.1186/s13063-023-07216-0 (PMC10032258; doi:10.1186/s13063-023-07216-0)
Supplement: Supplementary file 5 — Additional file 5. [file 13063_2023_7216_MOESM5_ESM.pdf]

BAASIS
The Basel Assessment of Adherence to immunoSuppressive medIcations Scale©

The Basel Assessment of Adherence to immunoSuppressive medIcations Scale (BAASIS©) – *written questionnaire (self-report)*

Introduction to questionnaire

Anti-rejection medications are the tablets you take to prevent your body from rejecting your transplanted organ. Taking anti-rejection medications correctly *every day at the same time,* can be difficult for many patients.

We would like to learn how you manage these medications in daily life**.**

Please answer the following questions as correctly and honestly as you can.

© University of Basel, Leuven-Basel Research Group, Institute of Nursing Science, Department Public Health, University of Basel, Switzerland, 2005. Permission & conditions to use the BAASIS© can be obtained from: http://baasis.nursing.unibas.ch/ or sabina.degeest@unibas.ch

1A. Did you miss any doses, even one, of any of your anti-rejection medications in the past 4 weeks?

☐ Yes ☐ No

(If yes): **How often did this happen in the past 4 weeks?**

☐ Once
☐ Twice
☐ Three times
☐ Four times
☐ More than four times

1B. *If you answered yes to question 1A*, did you ever skip two or more doses in a row in the past 4 weeks?

☐ Yes ☐ No

(*If yes*): How often did this happen in the past 4 weeks?

☐ Once
☐ Twice
☐ Three times
☐ Four times
☐ More than four times

© University of Basel, Leuven-Basel Research Group, Institute of Nursing Science, Department Public Health, University of Basel, Switzerland, 2005. Permission & conditions to use the BAASIS© can be obtained from: http://baasis.nursing.unibas.ch/ or sabina.degeest@unibas.ch

2. Did you take any of your anti-rejection medications *more than 2 hours before or after the time you usually take them* in the past 4 weeks?

☐ Yes ☐ No

(*If yes*): How often did this happen in the past 4 weeks?

☐ Once
☐ Two to three times
☐ About once weekly
☐ A couple of times per week
☐ Almost every day

3. Have you changed the prescribed amount of any of your anti-rejection medications during the past 4 weeks, on your own initiative without your doctor telling you to? For example, have you taken more or fewer pills or changed the dose, maybe by cutting a pill in half?

☐ Yes ☐ No

4. Have you completely stopped taking any of your anti-rejection medications within the past year, on your own initiative without your doctor telling to do that?

☐ Yes ☐ No

© University of Basel, Leuven-Basel Research Group, Institute of Nursing Science, Department Public Health, University of Basel, Switzerland, 2005. Permission & conditions to use the BAASIS© can be obtained from: http://baasis.nursing.unibas.ch/ or sabina.degeest@unibas.ch

5. Did your family doctor or specialist give you any prescription for new medication in the past year (Maybe high blood pressure medication or cholesterol/lipid lowering drugs)?

☐ Yes ☐ No

(If yes): **Did you fill the prescription at the pharmacy *<u>and</u>* start taking this new medication?**

☐ Yes ☐ No

© University of Basel, Leuven-Basel Research Group, Institute of Nursing Science, Department Public Health, University of Basel, Switzerland, 2005. Permission & conditions to use the BAASIS© can be obtained from: http://baasis.nursing.unibas.ch/ or sabina.degeest@unibas.ch
