# Supplementary material for: A multicentre, patient- and assessor-blinded, non-inferiority, randomised and controlled phase II trial to compare standard and torque teno virus-guided immunosuppression in kidney transplant recipients in the first year after transplantation: TTVguideIT
Source: Trials. 2023 Mar 22;24:213. doi: 10.1186/s13063-023-07216-0 (PMC10032258; doi:10.1186/s13063-023-07216-0)
Supplement: Supplementary file 6 — Additional file 6. [file 13063_2023_7216_MOESM6_ESM.pdf]

Psychological evaluation

Patient-ID		TX-Date	
Site-No.		Trial-Site	
Protocol Code		PI	

Study-Visit		Date		Time	

Social Status

1. ☐ Single
2. ☐ Married
3. ☐ Divorced
4. ☐ Separated
5. ☐ Widowed
6. ☐ In a stable relationship

Children

☐ Yes - How many? ___________
☐ No

Level of education

1. ☐ No school degree
2. ☐ Primary education[a]
3. ☐ Secondary education[b]
4. ☐ High school degree[c]
5. ☐ Vocational school degree[d]
6. ☐ University degree [e]

Employment status

1. ☐ Employed
2. ☐ Self-employed
3. ☐ Sick leave
4. ☐ Pension/rehab
5. ☐ Pension/age
6. ☐ Unemployed

Social support/resources

Emotional and practical support from family

☐ Yes
☐ No

Emotional and practical support from friends

☐ Yes
☐ No

[a] elementary, basic education (ISCED level 1 - Int. Standard Classification of Education)
[b] lower secondary education e.g. middle school, junior high school, (ISCED level 2)
[c] Degree after upper secondary education e.g. senior high school, eligibility to university (ISCED level 3)
[d] Degree after vocational/ professional school (ISCED level 3-5)
[e] Degree after university education (Bachelor- or Master-programs, Magister programs (ISCED level 6-7)

©Dr. Bettina Gober, Mag Katharina Ebenberger, Dr. Frederik Haupenthal

Addiction

1. ☐ Alcohol (for women: >12 g/d = 0.3L beer, 0.13L wine or 4cL 38% alc.;
   for men: >24 g/d = 0.6L beer, 0.26L wine or 8cl 38% alc. [f])
2. ☐ Illegal substance abuse
3. ☐ Nicotine abuse
4. ☐ Medication abuse
5. ☐ None

Critical life events within the last year

(Loss, separation, experience of violence, abuse, traumatic experiences, previous illnesses)

☐ Yes

☐ No

If yes, WHICH EVENT:____

and WHEN :____

Psychiatric History

1. ☐ None
2. ☐ Post-traumatic stress disorder
3. ☐ Depression
4. ☐ Anxiety disorder
5. ☐ Personality disorder
6. ☐ Others____

Treatment:

☐ Yes
☐ No

Transplantation and Treatment

I received the best possible treatment.

☐ Yes
☐ No

I receive information about the procedures and examinations that are important to me.

☐ Yes
☐ No

I have the opportunity to participate in medical decisions concerning my care.

☐ Yes
☐ No

The team gives me the feeling that they are "taking care of me".

☐ Yes
☐ No

The team treats me with respect.

☐ Yes
☐ No

There is an overall positive atmosphere in the team.

☐ Yes
☐ No

[f] according to WHO; alc. = alcohol

©Dr. Bettina Gober, Mag Katharina Ebenberger, Dr. Frederik Haupenthal

Future perspective and resources

Goals, motives, resources can be named (family, job, leisure time)

- ☐ Yes
- ☐ No

Additional important information:

©Dr. Bettina Gober, Mag Katharina Ebenberger, Dr. Frederik Haupenthal
