# Supplementary material for: A multicentre, patient- and assessor-blinded, non-inferiority, randomised and controlled phase II trial to compare standard and torque teno virus-guided immunosuppression in kidney transplant recipients in the first year after transplantation: TTVguideIT
Source: Trials. 2023 Mar 22;24:213. doi: 10.1186/s13063-023-07216-0 (PMC10032258; doi:10.1186/s13063-023-07216-0)
Supplement: Supplementary file 7 — Additional file 7. [file 13063_2023_7216_MOESM7_ESM.pdf]

MTSOSD

MODIFIED TRANSPLANT SYMPTOM OCCURRENCE AND SYMPTOM DISTRESS SCALE

Instructions

Taking medication after transplantation is associated with certain side effects, which may or may not be distressing to you.

In this questionnaire, each question is divided into two columns, as shown in the example below.

Example:

1. I have had itching	My itching was
O Never *O 0ccasionally* *☒ Regularly* *O Almost always* *O Always*	*not distressing at all* ... *terribly distressing* *0____1____(2)____3____4*

The ***left*** column asks about the ***occurrence*** of the **side effects**. Please indicate how frequently or how severely you have experienced a given symptom **during the past 4 weeks** by checking off the appropriate answer.

In the ***right*** column you will find questions asking you whether these symptoms are **distressing** to you. Please circle the number that corresponds with how distressing this symptom is to you.

If a symptom **does not occur**, answer **"never"** or **"not"** in the left column and go directly to the **next question**.

At the end of the questionnaire, you can add additional side effects that you may have experienced during the **past 4 weeks**. Please check if you have completed all items and all pages. Please also be sure that you have completed both the right and left column of the questionnaire.

Contact information of the authors:

Philip Moons
Center for Health Services and Nursing Research
Katholieke Universiteit Leuven (Belgium)
Tel: +32 16 33 69 84
E-mail: Philip.Moons@med.kuleuven.ac.be

Fabienne Dobbels
Center for Health Services and Nursing Research
Katholieke Universiteit Leuven (Belgium)
Tel: +32 16 33 69 76
E-mail: Fabienne.Dobbels@med.kuleuven.ac.be

Sabina De Geest
Institute of Nursing Science
University of Basel (Switzerland)
Tel: +41 61 267 09 51
E-mail: Sabina.DeGeest@unibas.ch

Developed to assess occurrence and distress experienced by the side effects of Cyclosporine, Tacrolimus, Micophenolate Mofetil, Azathioprine, Prednisolone, Sirolimus, LEA29Y.

Partly based on the Transplant Symptom Frequency and Symptom Distress Scale: Lough ME, Lindsey AM, Shinn JA, Stotts NA. Life satisfaction following heart transplantation. J Heart Transplantation 1985; 4: 446-449 & Lough ME, Lindsey AM, Shinn JA, Stotts NA. Impact of symptom frequency and symptom distress on self-reported quality of life in heart transplant recipients. Heart Lung 1987; 16: 193-200.

During the past 4 weeks (including today):

1. I have had itching<br><br>O *Never*<br>O *0ccasionally*<br>O *Regularly*<br>O *Almost always*<br>O *Always*	My itching was<br><br>*not distressing at all* … *terribly distressing*<br><br>*0_____1_____2_____3_____4*
2. I have had chest pain<br><br>O *Never*<br>O *0ccasionally*<br>O *Regularly*<br>O *Almost always*<br>O *Always*	My chest pain was<br><br>*not distressing at all* … *terribly distressing*<br><br>*0_____1_____2_____3_____4*
3. I have had wind<br><br>O *Never*<br>O *0ccasionally*<br>O *Regularly*<br>O *Almost always*<br>O *Always*	My wind was<br><br>*not distressing at all* … *terribly distressing*<br><br>*0_____1_____2_____3_____4*
4. I have had increased thirst<br><br>O *Never*<br>O *0ccasionally*<br>O *Regularly*<br>O *Almost always*<br>O *Always*	My increased thirst was<br><br>*not distressing at all* … *terribly distressing*<br><br>*0_____1_____2_____3_____4*
5. I have felt restless or nervous<br><br>O *Never*<br>O *0ccasionally*<br>O *Regularly*<br>O *Almost always*<br>O *Always*	My restlessness or nervousness was<br><br>*not distressing at all* … *terribly distressing*<br><br>*0_____1_____2_____3_____4*
6. I have had hearing loss<br><br>O *Not*<br>O *A little*<br>O *Moderately*<br>O *Greatly*<br>O *Very greatly*	My hearing loss was<br><br>*not distressing at all* … *terribly distressing*<br><br>*0_____1_____2_____3_____4*

During the past 4 weeks (including today):

7. I have had an abnormal skin color<br><br>O *Not*<br>O *A little*<br>O *Moderately*<br>O *Greatly*<br>O *Very greatly*	My abnormal skin color was<br><br>*not distressing at all* — *terribly distressing*<br><br>*0_____1_____2_____3_____4*
8. I have had increased sweating<br><br>O *Never*<br>O *0ccasionally*<br>O *Regularly*<br>O *Almost always*<br>O *Always*	My increased sweating was<br><br>*not distressing at all* — *terribly distressing*<br><br>*0_____1_____2_____3_____4*
9. My face and neck have been red<br><br>O *Never*<br>O *0ccasionally*<br>O *Regularly*<br>O *Almost always*<br>O *Always*	The redness in my face and neck was<br><br>*not distressing at all* — *terribly distressing*<br><br>*0_____1_____2_____3_____4*
10. I have had brittle fingernails<br><br>O *Not*<br>O *A little*<br>O *Moderately*<br>O *Greatly*<br>O *Very greatly*	My brittle fingernails were<br><br>*not distressing at all* — *terribly distressing*<br><br>*0_____1_____2_____3_____4*
11. My breasts have been larger<br><br>O *Not*<br>O *A little*<br>O *Moderately*<br>O *Greatly*<br>O *Very greatly*	My breast enlargement was<br><br>*not distressing at all* — *terribly distressing*<br><br>*0_____1_____2_____3_____4*
12. I have had sores on my lips and/or in my mouth<br><br>O *Never*<br>O *0ccasionally*<br>O *Regularly*<br>O *Almost always*<br>O *Always*	My sores on lips and/or in mouth were<br><br>*not distressing at all* — *terribly distressing*<br><br>*0_____1_____2_____3_____4*

During the past 4 weeks (including today):

13. I have had an altered voice<br><br>O *Not*<br>O *A little*<br>O *Moderately*<br>O *Greatly*<br>O *Very greatly*	My altered voice was<br><br>*not distressing at all* ... *terribly distressing*<br><br>*0_____1_____2_____3_____4*
14. I have had oily skin<br><br>O *Never*<br>O *0ccasionally*<br>O *Regularly*<br>O *Almost always*<br>O *Always*	My oily skin was<br><br>*not distressing at all* ... *terribly distressing*<br><br>*0_____1_____2_____3_____4*
15. I have felt dizzy<br><br>O *Never*<br>O *0ccasionally*<br>O *Regularly*<br>O *Almost always*<br>O *Always*	My dizziness was<br><br>*not distressing at all* ... *terribly distressing*<br><br>*0_____1_____2_____3_____4*
16. My hands have trembled<br><br>O *Never*<br>O *0ccasionally*<br>O *Regularly*<br>O *Almost always*<br>O *Always*	My trembling hands were<br><br>*not distressing at all* ... *terribly distressing*<br><br>*0_____1_____2_____3_____4*
17. I have had an increased urge to urinate<br><br>O *Never*<br>O *0ccasionally*<br>O *Regularly*<br>O *Almost always*<br>O *Always*	My increased urge to urinate was<br><br>*not distressing at all* ... *terribly distressing*<br><br>*0_____1_____2_____3_____4*
18. I have had a feeling of warmth in my hands and feet<br><br>O *Never*<br>O *0ccasionally*<br>O *Regularly*<br>O *Almost always*<br>O *Always*	The feeling of warmth in my hands and feet was<br><br>*not distressing at all* ... *terribly distressing*<br><br>*0_____1_____2_____3_____4*

During the past 4 weeks (including today):

19. I have had <u>bruises</u> more easily<br><br>O *Never*<br>O *0ccasionally*<br>O *Regularly*<br>O *Almost always*<br>O *Always*	My <u>bruises</u> were<br><br>*not distressing at all* ... *terribly distressing*<br><br>*0_____1_____2_____3_____4*
20. I have had <u>sores or warts around my genitals</u><br><br>O *Never*<br>O *0ccasionally*<br>O *Regularly*<br>O *Almost always*<br>O *Always*	My <u>sores or warts around genitals</u> were<br><br>*not distressing at all* ... *terribly distressing*<br><br>*0_____1_____2_____3_____4*
21. I have had <u>spots on my face and/or my back</u><br><br>O *Never*<br>O *0ccasionally*<br>O *Regularly*<br>O *Almost always*<br>O *Always*	My <u>spots</u> on my face and/or back were<br><br>*not distressing at all* ... *terribly distressing*<br><br>*0_____1_____2_____3_____4*
22. I have had an <u>excessive appetite</u><br><br>O *Never*<br>O *0ccasionally*<br>O *Regularly*<br>O *Almost always*<br>O *Always*	My <u>excessive appetite</u> was<br><br>*not distressing at all* ... *terribly distressing*<br><br>*0_____1_____2_____3_____4*
23. I have felt <u>depressed</u><br><br>O *Never*<br>O *0ccasionally*<br>O *Regularly*<br>O *Almost always*<br>O *Always*	My <u>feelings of depression</u> were<br><br>*not distressing at all* ... *terribly distressing*<br><br>*0_____1_____2_____3_____4*
24. <u>My gums have swollen</u><br><br>O *Not*<br>O *A little*<br>O *Moderately*<br>O *Greatly*<br>O *Very greatly*	My <u>swollen gums</u> were<br><br>*not distressing at all* ... *terribly distressing*<br><br>*0_____1_____2_____3_____4*

During the past 4 weeks (including today):

25. I have had swollen glands in my neck, armpit or groin

- O *Never*
- O *0ccasionally*
- O *Regularly*
- O *Almost always*
- O *Always*

My swollen glands were

not distressing at all — *terribly distressing*

0_____1_____2_____3_____4

---

26. I have had thinning of hair or hair loss

- O *Not*
- O *A little*
- O *Moderately*
- O *Greatly*
- O *Very greatly*

My hair thinning or hair loss was

not distressing at all — *terribly distressing*

0_____1_____2_____3_____4

---

27 A. I have had menstrual problems ***(for females only)***

- O *Not*
- O *A little*
- O *Moderately*
- O *Greatly*
- O *Very greatly*

My menstrual problems were

not distressing at all — *terribly distressing*

0_____1_____2_____3_____4

---

27 B. I have had erectile problems ***(for males only)***

- O *Never*
- O *0ccasionally*
- O *Regularly*
- O *Almost always*
- O *Always*

My erectile problems were

not distressing at all — *terribly distressing*

0_____1_____2_____3_____4

---

28. I have had a puffy face (moon face)

- O *Not*
- O *A little*
- O *Moderately*
- O *Greatly*
- O *Very greatly*

My puffy face was

not distressing at all — *terribly distressing*

0_____1_____2_____3_____4

---

29. I have had swollen ankles or feet

- O *Never*
- O *0ccasionally*
- O *Regularly*
- O *Almost always*
- O *Always*

My swollen ankles or feet were

not distressing at all — *terribly distressing*

0_____1_____2_____3_____4

During the past 4 weeks (including today):

30. I have had diarrhea<br><br>O *Never*<br>O *0ccasionally*<br>O *Regularly*<br>O *Almost always*<br>O *Always*	My diarrhea was<br><br>*not distressing at all* … *terribly distressing*<br><br>*0_____1_____2_____3_____4*
31. I have had tingling or numbness in my hands or feet<br><br>O *Never*<br>O *0ccasionally*<br>O *Regularly*<br>O *Almost always*<br>O *Always*	My tingling or numbness in my hands or feet was<br><br>*not distressing at all* … *terribly distressing*<br><br>*0_____1_____2_____3_____4*
32. I have had back pain<br><br>O *Never*<br>O *0ccasionally*<br>O *Regularly*<br>O *Almost always*<br>O *Always*	My back pain was<br><br>*not distressing at all* … *terribly distressing*<br><br>*0_____1_____2_____3_____4*
33. I have had a brittle skin<br><br>O *Not*<br>O *A little*<br>O *Moderately*<br>O *Greatly*<br>O *Very greatly*	My brittle skin was<br><br>*not distressing at all* … *terribly distressing*<br><br>*0_____1_____2_____3_____4*
34. I have felt anxious<br><br>O *Never*<br>O *0ccasionally*<br>O *Regularly*<br>O *Almost always*<br>O *Always*	My feelings of anxiety were<br><br>*not distressing at all* … *terribly distressing*<br><br>*0_____1_____2_____3_____4*
35. I have been experiencing mood swings<br><br>O *Never*<br>O *0ccasionally*<br>O *Regularly*<br>O *Almost always*<br>O *Always*	My mood swings were<br><br>*not distressing at all* … *terribly distressing*<br><br>*0_____1_____2_____3_____4*

During the past 4 weeks (including today):

36. I have had headaches<br><br>O *Never*<br>O *0ccasionally*<br>O *Regularly*<br>O *Almost always*<br>O *Always*	My headaches were<br><br>*not distressing at all* … *terribly distressing*<br><br>*0_____1_____2_____3_____4*
37. My facial features have changed<br><br>O *Not*<br>O *A little*<br>O *Moderately*<br>O *Greatly*<br>O *Very greatly*	My changed facial features were<br><br>*not distressing at all* … *terribly distressing*<br><br>*0_____1_____2_____3_____4*
38. I have had fat deposits on my neck and back ("buffalo hump")<br><br>O *Not*<br>O *A little*<br>O *Moderately*<br>O *Greatly*<br>O *Very greatly*	My fat deposits on neck and back were<br><br>*not distressing at all* … *terribly distressing*<br><br>*0_____1_____2_____3_____4*
39. I have had difficulty concentrating and/or memory problems<br><br>O *Never*<br>O *0ccasionally*<br>O *Regularly*<br>O *Almost always*<br>O *Always*	My concentration difficulties and/or memory problems were<br><br>*not distressing at all* … *terribly distressing*<br><br>*0_____1_____2_____3_____4*
40. I have had warts on hands and feet<br><br>O *Never*<br>O *0ccasionally*<br>O *Regularly*<br>O *Almost always*<br>O *Always*	My warts on hands and feet were<br><br>*not distressing at all* … *terribly distressing*<br><br>*0_____1_____2_____3_____4*
41. I have had increased hair growth on face and body<br><br>O *Not*<br>O *A little*<br>O *Moderately*<br>O *Greatly*<br>O *Very greatly*	My increased hair growth on face and body were<br><br>*not distressing at all* … *terribly distressing*<br><br>*0_____1_____2_____3_____4*

During the past 4 weeks (including today):

42. I have had <u>sleep difficulties</u><br>O *Never*<br>O *0ccasionally*<br>O *Regularly*<br>O *Almost always*<br>O *Always*	My <u>sleep difficulties</u> were<br>*not distressing at all* — *terribly distressing*<br>*0_____1_____2_____3_____4*
43. I have had <u>muscle weakness</u><br>O *Not*<br>O *A little*<br>O *Moderately*<br>O *Greatly*<br>O *Very greatly*	My <u>muscle weakness</u> was<br>*not distressing at all* — *terribly distressing*<br>*0_____1_____2_____3_____4*
44. My <u>sense of taste</u> has changed<br>O *Never*<br>O *0ccasionally*<br>O *Regularly*<br>O *Almost always*<br>O *Always*	The <u>change in my sense of taste</u> was<br>*not distressing at all* — *terribly distressing*<br>*0_____1_____2_____3_____4*
45. I have had a <u>poor appetite</u><br>O *Never*<br>O *0ccasionally*<br>O *Regularly*<br>O *Almost always*<br>O *Always*	My <u>poor appetite</u> was<br>*not distressing at all* — *terribly distressing*<br>*0_____1_____2_____3_____4*
46. I have felt <u>tired</u><br>O *Never*<br>O *0ccasionally*<br>O *Regularly*<br>O *Almost always*<br>O *Always*	My <u>tiredness</u> was<br>*not distressing at all* — *terribly distressing*<br>*0_____1_____2_____3_____4*
47. I have had <u>lack of energy</u><br>O *Never*<br>O *0ccasionally*<br>O *Regularly*<br>O *Almost always*<br>O *Always*	My <u>lack of energy</u> was<br>*not distressing at all* — *terribly distressing*<br>*0_____1_____2_____3_____4*

During the past 4 weeks (including today):

48. I have had stomach complaints, I have felt nauseous and/or I had to vomit<br><br>*O Never*<br>*O 0ccasionally*<br>*O Regularly*<br>*O Almost always*<br>*O Always*	My stomach complaints, nausea or vomiting were<br><br>*not distressing at all* … *terribly distressing*<br><br>*0_____1_____2_____3_____4*
49. I have had pain in my joints<br><br>*O Never*<br>*O 0ccasionally*<br>*O Regularly*<br>*O Almost always*<br>*O Always*	My joint pain was<br><br>*not distressing at all* … *terribly distressing*<br><br>*0_____1_____2_____3_____4*
50. I have had a rash on my skin<br><br>*O Never*<br>*O 0ccasionally*<br>*O Regularly*<br>*O Almost always*<br>*O Always*	My skin rash was<br><br>*not distressing at all* … *terribly distressing*<br><br>*0_____1_____2_____3_____4*
51. I have had muscle cramps<br><br>*O Never*<br>*O 0ccasionally*<br>*O Regularly*<br>*O Almost always*<br>*O Always*	My muscle cramps were<br><br>*not distressing at all* … *terribly distressing*<br><br>*0_____1_____2_____3_____4*
52. I have had nightmares<br><br>*O Never*<br>*O 0ccasionally*<br>*O Regularly*<br>*O Almost always*<br>*O Always*	My nightmares were<br><br>*not distressing at all* … *terribly distressing*<br><br>*0_____1_____2_____3_____4*
53. I have been short of breath<br><br>*O Not*<br>*O A little*<br>*O Moderately*<br>*O Greatly*<br>*O Very greatly*	My shortness of breath was<br><br>*not distressing at all* … *terribly distressing*<br><br>*0_____1_____2_____3_____4*

During the past 4 weeks (including today):

54. I have had a dry skin<br><br>*O Never*<br>*O 0ccasionally*<br>*O Regularly*<br>*O Almost always*<br>*O Always*	My dry skin was<br><br>*not distressing at all* … *terribly distressing*<br><br>*0_____1_____2_____3_____4*
55. I have had palpitations<br><br>*O Never*<br>*O 0ccasionally*<br>*O Regularly*<br>*O Almost always*<br>*O Always*	My palpitations were<br><br>*not distressing at all* … *terribly distressing*<br><br>*0_____1_____2_____3_____4*
56. I have had constipation<br><br>*O Never*<br>*O 0ccasionally*<br>*O Regularly*<br>*O Almost always*<br>*O Always*	My constipation was<br><br>*not distressing at all* … *terribly distressing*<br><br>*0_____1_____2_____3_____4*
57. I have had difficulty seeing well<br><br>*O Not*<br>*O A little*<br>*O Moderately*<br>*O Greatly*<br>*O Very greatly*	My seeing difficulties were<br><br>*not distressing at all* … *terribly distressing*<br><br>*0_____1_____2_____3_____4*
58. I have had a reduced interest in sex<br><br>*O Never*<br>*O 0ccasionally*<br>*O Regularly*<br>*O Almost always*<br>*O Always*	My reduced interest in sex was<br><br>*not distressing at all* … *terribly distressing*<br><br>*0_____1_____2_____3_____4*
59. My eyes have been sensitive to light<br><br>*O Never*<br>*O 0ccasionally*<br>*O Regularly*<br>*O Almost always*<br>*O Always*	My sensitivity to light was<br><br>*not distressing at all* … *terribly distressing*<br><br>*0_____1_____2_____3_____4*

Please check if you have completed all items and all pages.

Please also be sure that you have completed both the right and left column of the questionnaire.

THANK YOU VERY MUCH FOR YOUR COOPERATION
