# Supplementary material for: A multicentre, patient- and assessor-blinded, non-inferiority, randomised and controlled phase II trial to compare standard and torque teno virus-guided immunosuppression in kidney transplant recipients in the first year after transplantation: TTVguideIT
Source: Trials. 2023 Mar 22;24:213. doi: 10.1186/s13063-023-07216-0 (PMC10032258; doi:10.1186/s13063-023-07216-0)
Supplement: Supplementary file 8 — Additional file 8. [file 13063_2023_7216_MOESM8_ESM.pdf]

Report for the Application Evaluation Decision

A non-inferiority, randomised and controlled trial to compare the safety, tolerability and preliminary efficacy between standard and Torque Teno virus-guided immunosuppression in stable adult kidney transplant recipients with low immunological risk in the first year after transplantation

2022-500024-30-00

Decision

MSC:

Austria

Decision:

Authorised

Reporting Date:

01/07/2022

Tacit decision:

No

Application Part:

Conditions:

Reason:

Justification:

Deferrals

Disagreement with Part I

Submission date:

01/07/2022

Bundesamt für
Sicherheit im
Gesundheitswesen
BASG

BASG/AGES
Institute Surveillance
Traisengasse 5, 1200 Vienna

Medical University of Vienna
Department for Nephrology and Dialysis
Gregor Bond
Spitalgasse 23
1090 Wien

date: 01.07.2022
department: Clinical Trials (CLTR)
phone: +43(0)5 0555 36827
e-mail: clinicaltrials@ages.at
reference: 100769812

Notification of single decision according to Article 8 of REG (EU) 536/2014

Dear ladies and gentlemen,

the Federal Office for Safety in Healthcare („Bundesamt für Sicherheit im Gesundheitswesen") as the competent authority for clinical trials in Austria herewith notifies you about the decision for clinical trial

2022-500024-30-00

A non-inferiority, randomised and controlled trial to compare the safety, tolerability and preliminary efficacy between standard and Torque Teno virus-guided immunosuppression in stable adult kidney transplant recipients with low immunological risk in the first year after transplantation

The clinical trial is approved.

This formal letter is to be considered as notification within the terms of Article 8, section 6. The formal legal decision will be issued within the next 14 days.

Additional comments:

During the assessment of Part I changes were required that affected Part II documents. These documents were provided for response assessment in Part I, but could not been updated in the respective sections in Part II since this procedure was already closed at that time. The sponsor is requested to update the Part II documents in the respective sections during the next substantial modification to Part II.

For the Federal Office

Strasser Stefan
am 1.7.2022

BUNDESAMT FÜR SICHERHEIT IM GESUNDHEITSWESEN
AMTSSIGNATUR

Dieses Dokument wurde amtssigniert.

Informationen zur Prüfung der elektronischen Signatur und des Ausdrucks finden Sie unter http://www.basg.gv.at/amtssignatur.

Bundesamt für Sicherheit im Gesundheitswesen
Traisengasse 5, 1200 Wien

Report for the Application Evaluation Decision

A non-inferiority, randomised and controlled trial to compare the safety, tolerability and preliminary efficacy between standard and Torque Teno virus-guided immunosuppression in stable adult kidney transplant recipients with low immunological risk in the first year after transplantation

2022-500024-30-00

Decision

MSC:

Czechia

Decision:

Authorised

Reporting Date:

01/07/2022

Tacit decision:

No

Application Part:

Conditions:

Reason:

Justification:

Deferrals

Disagreement with Part I

Submission date:

01/07/2022

STÁTNÍ ÚSTAV PRO KONTROLU LÉČIV
Šrobárova 48
100 41 Praha 10
Telefon: +420 272 185 111
Fax: +420 271 732 377
E-mail: posta@sukl.cz
Web: www.sukl.cz

ADRESÁT
Medical University Of Vienna
Währinger Gürtel 18-20 Alsergrund
1090 Vienna
Austria

Spisová zn.	Vyřizuje / linka	Datum
51138/22-ctis	Dr. Hrušková Reinová / 317	01.07.2022

ROZHODNUTÍ

Státní ústav pro kontrolu léčiv se sídlem v Praze 10, Šrobárova 48 (dále jen „Ústav"), jako orgán příslušný k rozhodnutí podle § 13 odst. 2 písm. b) č. 378/2007 Sb., o léčivech a o změnách některých souvisejících zákonů (zákon o léčivech), ve znění pozdějších předpisů (dále jen „zákon o léčivech"), **rozhodl** v souladu s § 67 a násl. zákona č. 500/2004 Sb., správní řád, ve znění pozdějších předpisů (dále jen „správní řád"), v řízení o povolení klinického hodnocení humánního léčivého přípravku vedeného podle § 51 zákona o léčivech ve spojení s čl. 4 a násl. Nařízení Evropského Parlamentu a Rady (EU) č. 536/2014, o klinických hodnoceních humánních léčivých přípravků a o zrušení směrnice 2001/20/ES (dále jen „nařízení o klinickém hodnocení"), o žádosti o povolení klinického hodnocení podané prostřednictví portálu EU dne 07. 03. 2022 ohledně klinického hodnocení léčivého přípravku s názvem **A non-inferiority, randomised and controlled trial to compare the safety, tolerability and preliminary efficacy between standard and Torque Teno virus-guided immunosuppression in stable adult kidney transplant recipients with low immunological risk in the first year after transplantation** (dále jen „předmětné klinické hodnocení"), společnosti **Medical University Of Vienna**, se sídlem Spitalgasse 23 Alsergrund, 1090 Vienna, Austria

t a k t o :

Ústav v souladu s § 51 odst. 4 zákona o léčivech ve spojení s čl. 8 odst. 1 nařízení o klinickém hodnocení **povoluje předmětné klinické hodnocení**.

O d ů v o d n ě n í

Dne 07. 03. 2022 byla předložena žádost účastníka řízení o povolení předmětného klinického hodnocení prostřednictvím portálu EU s vyznačením České republiky jako dotčeného členského státu ve smyslu čl. 2 odst. 2 bod 12. nařízení o klinickém hodnocení. Předložením této žádosti bylo zahájeno správní řízení vedené pod sp. zn. 51128/22-ctis.

Ústav na základě předložené žádosti provedl řízení v souladu s § 51 odst. 3 písm. b) a c) zákona o léčivech ve spojení s čl. 5 a následující nařízení o klinickém hodnocení, a konstatuje, že předložená žádost splňuje požadavky relevantních právních předpisů.

S ohledem na výše uvedené rozhodl Ústav tak, jak je uvedeno ve výroku tohoto rozhodnutí.

Seznam schválené dokumentace:

Part I:

- Trial Protocol Version 5.0F, 03.06.2022
- SmPC of Advagraf (prolonged release) as Reference safety Information

- Pharmaceutical data

Part II:

- Informace pro pacienta a formulář informovaného souhlasu, verze 4.0F, 23.05.2022
- Instrukce pro pacienty k použití dávkovače léků
- Deník pacienta prop klinické hodnoceí TTV GUIDE IT, verze 0,4D ze dne 17.02.2022
- The Basel Assessment of Adherence to immunoSuppressive medlcations Scale (BAASIS©) – písemný dotazník (self-report)
- MTSOSD-R 59, Modifikovaná stupnice výskytu příznaků a obtížnosti příznaků u transplantace – revize 59
- Klinická studie – TTV guide IT – leták pro pacienty
- SF-36 (Czech) for Health Related Quality of Life Assessment – TTV-GUIDE-IT
- Kartička účastníka studie TTV guide IT
- Template k náboru pacientů
- Template pro kompenzace pacientů
- Certifikát o pojištění klinického hodnocení, pojistná smlouva, všeobecné pojistné podmínky, význam pojistných podmínek a informační leták k pojištění
- Informace pro pacienta a formulář informovaného souhlasu k zacházení s osobními údaji a biologickými vzorky, verze 4.0F. 03.06.2022
- CV Prof. MUDr. Ondřej Viklický, CSc a prohlášení o střetu zájmů zkoušejícího
- Template k vhodnosti centra
- Prohlášení zadavatele k zpracování osobních údajů

Poučení o odvolání

Proti tomuto rozhodnutí je možno podat podle § 81 a násl. správního řádu u Ústavu odvolání, a to ve lhůtě 15 dnů ode dne jeho doručení. O odvolání rozhoduje Ministerstvo zdravotnictví ČR.

Otisk úředního razítka

MUDr. Alice Němcová
Ředitelka Odboru klinického hodnocení léčivých přípravků

Elektronicky podepsal:
MUDr. Alice Němcová
Státní ústav pro kontrolu léčiv
Dne: 19.07.2022 13:57

EUROPEAN MEDICINES AGENCY
SCIENCE MEDICINES HEALTH

Report for the Application Evaluation Decision

A non-inferiority, randomised and controlled trial to compare the safety, tolerability and preliminary efficacy between standard and Torque Teno virus-guided immunosuppression in stable adult kidney transplant recipients with low immunological risk in the first year after transplantation

2022-500024-30-00

Decision

MSC:

Germany

Decision:

Authorised

Reporting Date:

01/07/2022

Tacit decision:

No

Application Part:

Conditions:

Reason:

Justification:

Deferrals

Disagreement with Part I

Submission date:

01/07/2022

Bundesinstitut
für Arzneimittel
und Medizinprodukte

BfArM, Kurt-Georg-Kiesinger-Allee 3, 53175 Bonn

Medical University Of Vienna
Spitalgasse 23
1090 Vienna

ABTEILUNG Informationstechnik, Klinische Prüfung
FACHGRUPPE Klinische Prüfung
TEL +49 (0)228 99 307-4318
E-MAIL ct@bfarm.de

HAUS-ANSCHRIFT Kurt-Georg-Kiesinger-Allee 3
53175 Bonn
TEL +49 (0)228 99 307-4318
FAX +49 (0)228 99 307-5207
E-MAIL poststelle@bfarm.de
INTERNET www.bfarm.de

Bonn, 30.06.2022
GESCHZ 10.3-2022-500024-30-00-00005

Verfahren der Genehmigung einer klinischen Prüfung gemäß Artikel 5, 8 der Verordnung (EU) Nr. 536/2014 i.V.m. § 40 AMG

Antrag vom 07.03.2022 (Part I) / 07.03.2022 (Part II)

**EU-CT-Nummer**	**2022-500024-30-00**
**Verfahrensnummer**	**00005**
**Sponsor**	**Medical University Of Vienna, Vienna,**
**Gesetzl. Vertreter**	**, ,**
**Studientitel**	**A non-inferiority, randomised and controlled trial to compare the safety, tolerability and preliminary efficacy between standard and Torque Teno virus-guided immunosuppression in stable adult kidney transplant recipients with low immunological risk in the first year after transplantation**
**Prüfpräparat**	**-, TACROLIMUS**

Bescheid

Diese klinische Prüfung wird

☑ genehmigt.

☐ mit Auflagen genehmigt. Die Auflagen lauten wie folgt:
Auflagen

Bewertung:

A. Schlussfolgerung zu Teil I des Bewertungsberichts:

☐ Die Bundesrepublik Deutschland ist als berichterstattender Mitgliedstaat (rMS) beteiligt und kommt in Bezug auf Teil I des Bewertungsberichts zu dem Schluss, dass die Durchführung der klinischen Prüfung

☐ vertretbar ist.

☐ mit Auflagen gemäß Artikel 8 Absatz 1 Unterabsatz 3 Verordnung (EU) Nr. 536/2014 vertretbar ist, siehe oben
Begründung der Auflage(n), siehe Anlage

☑ Die Bundesrepublik Deutschland ist als betroffener Mitgliedstaat (MSc) beteiligt und kommt in Übereinstimmung mit der Schlussfolgerung des rMS zu dem Schluss, dass die Durchführung der klinischen Prüfung

☑ vertretbar ist gemäß Artikel 8 Absatz 2 Verordnung (EU) Nr. 536/2014

☐ mit Auflagen vertretbar ist gemäß Artikel 8 Absatz 2 Verordnung (EU) Nr. 536/2014, siehe oben
Begründung der Auflage(n), siehe Anlage

☐ Die Bundesoberbehörde weicht von der Stellungnahme der Ethik-Kommission ab, § 40 Absatz 8 Satz 3 AMG.
Bezeichnung der Ethik-Kommission: Ethik-Kommission der Ärztekammer Nordrhein
Wiedergabe der Stellungnahme und Begründung für das Abweichen, siehe Anlage

B. Schlussfolgerung zu Teil II des Bewertungsberichts:

Bezeichnung der Ethik-Kommission:
Ethik-Kommission der Ärztekammer Nordrhein
Tersteegenstr. 9
Düsseldorf

Die Ethikkommission kommt zu dem Schluss, dass die Durchführung der klinischen Prüfung

☑ vertretbar ist.

☐ mit Auflagen vertretbar ist, siehe oben
Begründung der Auflage(n), siehe Anlage

Rechtsbehelfsbelehrung:

Gegen diesen Bescheid kann innerhalb eines Monats nach Bekanntgabe Widerspruch erhoben werden. Der Widerspruch ist bei dem Bundesinstitut für Arzneimittel und Medizinprodukte (BfArM) in Bonn einzulegen.

Mit freundlichen Grüßen
Im Auftrag

Dr. C. Riedel

Dieser Bescheid enthält in Übereinstimmung mit § 37 Absatz 3 Satz 1 Verwaltungsverfahrensgesetz nur eine Namenswiedergabe und keine Unterschrift.

Anlage X (nur bei Bedarf!)

Begründung der Auflage(n):

EUROPEAN MEDICINES AGENCY
SCIENCE MEDICINES HEALTH

Report for the Application Evaluation Decision

A non-inferiority, randomised and controlled trial to compare the safety, tolerability and preliminary efficacy between standard and Torque Teno virus-guided immunosuppression in stable adult kidney transplant recipients with low immunological risk in the first year after transplantation

2022-500024-30-00

Decision

MSC:

Spain

Decision:

Authorised

Reporting Date:

08/07/2022

Tacit decision:

No

Application Part:

Conditions:

Reason:

Justification:

Deferrals

Disagreement with Part I

Submission date:

08/07/2022

DEPARTAMENTO
DE MEDICAMENTOS
DE USO HUMANO
Área de Ensayos Clínicos

REFERENCIA: MUH/CLIN/EC

ASUNTO: RESOLUCIÓN DE LA SOLICITUD DE AUTORIZACIÓN DE UN ENSAYO CLÍNICO

DATOS DE LA SOLICITUD

Promotor: Medical University Of Vienna
Spitalgasse 23Alsergrund, Vienna
Austria

Ensayo clínico: Nº EUCT **2022-500024-30-00** y título **A non-inferiority, randomised and controlled trial to compare the safety, tolerability and preliminary efficacy between standard and Torque Teno virus-guided immunosuppression in stable adult kidney transplant recipients with low immunological risk in the first year after transplantation**.

Fecha de solicitud: 07/03/2022

Una vez evaluada la solicitud de ensayo clínico previamente indicada, se considera que cumple con los requisitos indicados en el Reglamento (UE) n°536/2014 del Parlamento Europeo y del Consejo, de 16 de abril de 2014, sobre los ensayos clínicos de medicamentos de uso humano, y demás legislación aplicable *.

Por todo lo anteriormente expuesto la Directora de la Agencia de Medicamentos y Productos Sanitarios en el ejercicio de sus competencias RESUELVE:

AUTORIZAR el ensayo clínico solicitado.
Si existe discrepancias entre la fecha de CTIS y la de este documento, prevalecerá la fecha de autorización de CTIS.

* Real Decreto 1090/2015, de 4 de diciembre, por el que se regulan los ensayos clínicos con medicamentos, los Comités de Ética de la Investigación con medicamentos y el Registro Español de Estudios Clínicos.
Texto refundido de la Ley de Garantías y Uso Racional de los medicamentos y productos sanitarios, aprobado por Real Decreto Legislativo 1/2015, de 24 de julio.
Real Decreto 1275/2011, de 16 de septiembre, por el que se crea la Agencia estatal "Agencia Española de Medicamentos y Productos Sanitarios" y se aprueba su Estatuto.

Agencia Española de Medicamentos y Productos Sanitarios (AEMPS) **CSV: 4 Q C 5 2 F 8 3 F D**
Fecha de la firma: 21/07/2022
Puede comprobar la autenticidad del documento en la sede de la AEMPS:https://localizador.aemps.es

CORREO ELECTRÓNICO
smhaem@aemps.es

C/ Campezo, 1 - Edificio 8, 28022 MADRID
Tel.: 918225073
Fax: 918225043

Contra esta Resolución, que pone fin a la vía administrativa, puede interponerse potestativamente Recurso de Reposición ante el/la Director/a de la "Agencia Española de Medicamentos y Productos Sanitarios" en el plazo de un mes, conforme a lo dispuesto en los artículos 123 y 124 de la Ley 39/2015, de 1 de octubre, del Procedimiento Administrativo Común de las Administraciones Públicas, o interponerse Recurso Contencioso-Administrativo ante el Juzgado Central de lo Contencioso- Administrativo de Madrid, en el plazo de dos meses a contar desde el día siguiente a la recepción de la presente notificación, conforme a lo dispuesto en la Ley Reguladora de la Jurisdicción Contencioso-Administrativa de 13 de julio de 1998, y sin perjuicio de cualquier otro recurso que pudiera interponerse.

DIRECTORA DE LA AGENCIA ESPAÑOLA DE MEDICAMENTOS Y PRODUCTOS SANITARIOS

Fdo. Mª Jesús Lamas Díaz

CORREO ELECTRÓNICO
smhaem@aemps.es

C/ Campezo, 1 - Edificio 8, 28022 MADRID
Tel.: 918225073
Fax: 918225043

EUROPEAN MEDICINES AGENCY
SCIENCE MEDICINES HEALTH

Report for the Application Evaluation Decision

A non-inferiority, randomised and controlled trial to compare the safety, tolerability and preliminary efficacy between standard and Torque Teno virus-guided immunosuppression in stable adult kidney transplant recipients with low immunological risk in the first year after transplantation

2022-500024-30-00

Decision

MSC:

France

Decision:

Authorised

Reporting Date:

28/06/2022

Tacit decision:

No

Application Part:

Conditions:

Reason:

Justification:

Deferrals

Disagreement with Part I

Submission date:

28/06/2022

Report for the Application Evaluation Decision

A non-inferiority, randomised and controlled trial to compare the safety, tolerability and preliminary efficacy between standard and Torque Teno virus-guided immunosuppression in stable adult kidney transplant recipients with low immunological risk in the first year after transplantation

2022-500024-30-00

Decision

MSC:

Netherlands

Decision:

Authorised

Reporting Date:

30/06/2022

Tacit decision:

No

Application Part:

Conditions:

Reason:

Justification:

Deferrals

Disagreement with Part I

Submission date:

30/06/2022

BESLUIT NEDERLAND/LETTER OF APPROVAL, THE NETHERLANDS
Primaire beoordeling

**EU CT-nummer**	2022-500024-30-00
**Titel onderzoek**	**A randomised and controlled trial to compare the safety, tolerability and preliminary efficacy between standard and Torque Teno virus-guided immunosuppression in stable adult kidney transplant recipients with low immunological risk in the first year after transplantation**
**Verrichter**	**Medical University Of Vienna**
**Datum besluit**	30 Juni 2022
**MREC nr**	2022/154
**Kenmerk**	M22.299954

Besluit

Het bovenstaande aanvraagdossier betreft een klinische proef als bedoeld in artikel 1 van Verordening (EU) Nr. 536/2014 (hierna: de verordening).
Het aanvraagdossier is gevalideerd, zoals vermeld in CTIS. Op grond van artikel 17a, eerste lid, aanhef en onder c, van de WMO heeft de CCMO het dossier ter beoordeling toegewezen aan de medisch-ethische toetsingscommissie UMC Groningen (METc UMCG).
De medisch-ethische toetsingscommissie UMC Groningen (METc UMCG) heeft zich, op grond van artikel 4 juncto artikel 8 van de verordening juncto artikel 2, tweede lid, aanhef en onder a, van de Wet medisch-wetenschappelijk onderzoek met mensen (WMO), beraden over het dossier.

De commissie besluit dat de klinische proef wordt toegelaten om uit te voeren in de volgende centra:
- University Medical Center Groningen (hoofdonderzoeker prof. dr. S.J.L. Bakker)
- Leiden University Medical Center (hoofdonderzoeker dr. J.I. Rothmans)

Dit besluit verliest zijn geldigheid als de inclusie van de eerste proefpersoon niet heeft plaatsgevonden binnen twee jaar na de datum van dit besluit.

Documenten

Voor een overzicht van de documenten waarover de commissie bij haar beoordeling de beschikking heeft gehad, wordt verwezen naar bijlage 1.

Achtergrond

Het dossier is besproken in de plenaire vergadering(en) van de METC op 24 april 2022 (zie bijlage 2 voor de samenstelling van de commissie).

Overwegingen

De commissie heeft de aspecten van deel I en deel II van het aanvraagdossier beoordeeld zoals neergelegd in de artikelen 6 en 7 van de verordening.
Zij concludeert in deel I van het beoordelingsrapport dat de uitvoering van de klinische proef

aanvaardbaar is. Daarnaast oordeelt de commissie dat wordt voldaan aan de in deel II van het beoordelingsrapport bedoelde aspecten.
Voor de overwegingen die tot deze conclusie hebben geleid, wordt verwezen naar de beoordelingsrapporten deel I en deel II zoals die zijn gepubliceerd in CTIS. De inhoud van de rapporten maakt onderdeel uit van dit besluit.

Ten slotte wijst de METc UMCG u op de verplichtingen die volgen uit de verordening.

De voorzitter van de METc UMCG,

Prof. Dr. H.P.H. Kremer

Bijlage 1

Documenten Deel I

B. Cover letter
B1. Cover letter 2022-500024-30-00, 28 March 2022
B1. Cover letter 2022-500024-30-00, version 3, 3 June 2022

D. Protocol
D1. Protocol 2022-500024-30-00, Version 5.0 F, 03.06.2022
D1. Protocol synopsis ENG 2022-500024-30-00, Version 5.0 F, 03.06.2022
D1. Protocol synopsis NL 2022-500024-30-00, Version 5.0 F, 03.06.2022
D3. DSMB Charter 2022-500024-30-00, version 0.1D

G. Investigational Medicinal Product Dossier
G2. SmPC Advagraf, prolonged_release_Capsules_hard, 24-02-2022
G2. SmPC Prograf capsules_hard, 17-02-2022
G2. SmPC Prograf Concentrate_for_solution], 24-02-2022
G2. SmPC_Prograf-article-30-referral-annex-i-ii-iii_en, version 1.0

J. Labeling
J1. TTV Guide IT_No Content labelling_nonpublic_v_0.1F

Documenten Deel II

K. Recruitment arrangement
K1. Template recruitment arrangements NL, 01-06-2022

L. Subject information sheet, informed consent form, other subject information material
L1. SIS and ICF adults, version 2.0F, 31-05-2022

M. Suitability investigator
M1. CV Investigator S.J.L. Bakker, 20-01-2022
M1. CV Investigator J.I Rotmans, 28-01-2022
M2. DoI Investigator S.J.L. Bakker, 17-01-2022
M2. DoI Investigator J.I. Rotmans, 21-01-2022

N. Suitability facilities
N1. VGO [UMCG], 11-02-2022
N1. VGO [LUMC], 03-03-2022

O. Proof of Insurance or idemnification

O1. WMO trial participant insurance certificate, policy holder: Medical University Vienna, insurer: HDI, 30-november-2021
O2. Proof of liability coverage sponsor or investigator [AZG (UMCG)], [januari 2020]
O2. Proof of liability coverage sponsor or investigator LUMC], [januari 2022]

P. Financial and other arrangements
P1. Template compensation trial participants, investigator, funding and other arrangements, 28-03-2022

R. Compliance GDPR
R1. Template on the collection and use personal data NL, 28-03-2022
R1. Statement on Compliance with data protection, version 2.0, 22-02-2022

S. Biological samples
S1. Template on the collection, use and storage of biological samples NL, version 1.0F, 07-03-2022

Bijlage 2

Samenstelling Universitair Medisch Centrum Groningen
De volgende leden zijn lid van de METC ten tijde van het nemen van het besluit.

Artsen

Dr. S. Aluwini, radiotherapeut
Prof. dr. R. Bruggeman, hoogleraar neuropsychiatrie en psychotische stoornissen
Prof. dr. R.L. Diercks, orthopedisch chirurg
Mw. dr. M.L. Duiverman, longarts
Mw. prof. dr. G.A.P. Hospers, medisch oncoloog
Dr. M. de Jongste, cardioloog
Prof. dr. J.th.M. Plukker, chirurg

Kinderartsen

Prof. dr. A.F. Bos, kinderarts / neonatoloog
Dr. P.F. van Rheenen, kinderarts

Ethici

Dhr. dr. J.P.H. de Jong, ethicus
Mw. dr. A.N. Raat, ethicus
Mw. dr. L.A.M. van der Scheer, gezondheidsethicus / filosoof
Mw. dr. M.J. Siebelink, programmamanager transplantatiecentrum / onderzoeker

Methodologen

Dr. B.Z. Alizadeh, genetisch epidemioloog
Dr. H. Groen, universitair docent epidemiologie
Mw. dr. I.M. Nolte, universitair docent epidemiologie

Medical device specialist

Dr. M.J.W. Greuter, klinisch fysicus
Dr. ir. J. Sjollema, universitair docent / fysicus

Juristen

Mw. mr. W.B. Veen, jurist
Mr. J.W.P. de Vroedt MHA, jurist / stafmedewerker
Mw. mr. J. Zaal, jurist/stafmedewerker

Klinisch farmacologen

Mw. prof. dr. P.M.L.A. van den Bemt, klinisch farmacoloog
Dr. M.S. Bolhuis, klinisch farmacoloog
Prof. dr. H.J. Lambers Heerspink, klinisch farmacoloog / onderzoeker
Mw. dr. M .G.G. Sturkenboom, klinisch farmacoloog

Ziekenhuisapothekers

Mw. prof. dr. P.M.L.A. van den Bemt, ziekenhuisapotheker
Dr. M.S. Bolhuis, ziekenhuisapotheker
Prof. dr. E. van Roon, ziekenhuisapotheker
Mw. dr. M .G.G. Sturkenboom, ziekenhuisapotheker

Proefpersoon leden

Mw. W. Hoek, adviseur/begeleider wonen, zorg, hulpverlening en welzijn
Mw. C.M. Verlind, fysiotherapeut n.p.
Mw. drs. N. van Wijngaarden, bestuurssecretaris

Overige leden

Dr. G.W. van Imhoff, internist-hematoloog niet-praktiserend
Prof. dr. W. Helfrich, hoogleraar Translationele Chirurgische Oncologie
Prof. dr. C.G.M. Kallenberg, emeritus hoogleraar Interne Geneeskunde i.h.b. Klinische Immunologie
Mw. dr. A.C. Muller Kobold, klinisch chemicus
Mr. D. Renkema, jurist gezondheidsrecht
Dr. H.G.O.M. Smid, psycholoog
Mw. R. A.E. Tooten, Bsc, clinical trial specialist
Mw. dr. E.L. van der Veen, ziekenhuisapotheker i.o. / klinisch farmacoloog i.o.
Dr. ir. P.J.F. de Vries, consultant voedingswetenschappen
Mw. dr. F. Zwiers-Blokzijl, verpleegkundig specialist & postdoc onderzoeker
