# Supplementary material for: A multicentre, patient- and assessor-blinded, non-inferiority, randomised and controlled phase II trial to compare standard and torque teno virus-guided immunosuppression in kidney transplant recipients in the first year after transplantation: TTVguideIT
Source: Trials. 2023 Mar 22;24:213. doi: 10.1186/s13063-023-07216-0 (PMC10032258; doi:10.1186/s13063-023-07216-0)
Supplement: Supplementary file 9 — Additional file 9. [file 13063_2023_7216_MOESM9_ESM.pdf]

Report for the Application Evaluation Decision

A non-inferiority, randomised and controlled trial to compare the safety, tolerability and preliminary efficacy between standard and Torque Teno virus-guided immunosuppression in stable adult kidney transplant recipients with low immunological risk in the first year after transplantation

2022-500024-30-00

Decision

MSC:

Netherlands

Decision:

Authorised

Reporting Date:

17/10/2022

Tacit decision:

No

Application Part:

Conditions:

Reason:

Justification:

Deferrals

Disagreement with Part I

Submission date:

17/10/2022

BESLUIT NEDERLAND/LETTER OF APPROVAL, THE NETHERLANDS
Substantiële wijziging deel I en deel II

**EU CT-nummer**	2022-500024-30-00
**Titel onderzoek**	A randomised and controlled trial to compare the safety, tolerability and preliminary efficacy between standard and Torque Teno virus-guided immunosuppression in stable adult kidney transplant recipients with low immunological risk in the first year after transplantation. TTV-GUIDE
**Verrichter**	Medical University Of Vienna
**Datum nader besluit**	17 oktober 2022
**MREC nr**	2022/154
**Kenmerk**	M22.304828

Besluit
Het bovenstaande aanvraagdossier betreft een klinische proef als bedoeld in artikel 1 van Verordening (EU) Nr. 536/2014 (hierna: de verordening).
De medisch-ethische toetsingscommissie van het UMC Groningen (METc UMCG) heeft zich, op grond van artikel 4 juncto artikel 23 van de verordening juncto artikel 2, tweede lid, aanhef en onder a, van de Wet medisch-wetenschappelijk onderzoek met mensen (WMO), beraden over een substantiële wijziging van deel I en deel II van bovengenoemd aanvraagdossier.
De substantiële wijziging heeft betrekking op o.a. aanpassing eindpunt, reductie totale volume af te nemen biomateriaal en update ICF.

De commissie heeft eerder een positief besluit afgegeven over het aanvraagdossier.

De commissie besluit dat de substantiële wijziging van deel I en deel II wordt toegelaten.

Uitvoering van de klinische proef vindt plaats in de volgende centra:
- University Medical Center Groningen (hoofdonderzoeker prof. dr. S.J.L. Bakker)
- Leiden University Medical Center (hoofdonderzoeker dr. J.I. Rothmans)

Documenten
De volledige set van goedgekeurde documenten staat vermeld in CTIS, waarvan de volgende documenten deel uitmaken:

D1. Protocol 2022-500024-30-00, Version 6.0 F, 15.07.2022
D1. Synopsis 2022-500024-30-00, Version 6.0 F, 15.07.2022
L1. SIS and ICF adults, version 4.0F, 14.07.2022
L2. Other subject information material TTV GUIDE IT_patient-folder_NL, version
O1. WMO trial participant insurance certificate, policy holder: Medical University Vienna, insurer: HDI, 05-08-2022.

Achtergrond
De substantiële wijziging is besproken in de vergadering van de METC op 6 september 2022 (zie bijlage 1 voor de samenstelling van de commissie).

Overwegingen
De commissie heeft de substantiële wijziging van aspecten van deel I en deel II van het aanvraagdossier beoordeeld overeenkomstig de artikelen 18 en 22 van de verordening.

Zij concludeert in deel I en deel II van het beoordelingsrapport dat de substantiële wijziging van de klinische proef aanvaardbaar is. Voor de overwegingen die tot deze conclusie hebben geleid, wordt verwezen naar deel I en deel II van het beoordelingsrapport zoals dat is gepubliceerd in CTIS. De inhoud van het rapport maakt onderdeel uit van dit besluit.

Ten slotte wijst de METc UMCG u op de verplichtingen die volgen uit de verordening.

De voorzitter van de METc UMCG,

Prof. dr. H.P.H. Kremer

Dit besluit is met zorg digitaal vastgesteld. Hierdoor staat er geen fysieke handtekening in het besluit.

Bijlage 1

Samenstelling METc UMCG
De volgende leden zijn lid van de METC ten tijde van het nemen van het besluit.
Leden die bij een onderzoeksprotocol een belangenconflict hebben, zijn niet betrokken bij de beoordeling van het onderzoeksprotocol.

Artsen

Dr. S. Aluwini, radiotherapeut
Prof. dr. R. Bruggeman, hoogleraar neuropsychiatrie en psychotische stoornissen
Prof. dr. R.L. Diercks, orthopedisch chirurg
Mw. dr. M.L. Duiverman, longarts
Mw. prof. dr. G.A.P. Hospers, medisch oncoloog
Dr. M. de Jongste, cardioloog
Mw. prof. dr. B.L. van Leeuwen, oncologisch chirurg
Prof. dr. J.th.M. Plukker, chirurg

Kinderartsen

Prof. dr. A.F. Bos, kinderarts / neonatoloog
Dr. P.F. van Rheenen, kinderarts

Ethici

Dhr. dr. J.P.H. de Jong, ethicus
Mw. dr. A.N. Raat, ethicus
Mw. dr. L.A.M. van der Scheer, gezondheidsethicus / filosoof
Mw. dr. M.J. Siebelink, programmamanager transplantatiecentrum / onderzoeker

Methodologen

Dr. B.Z. Alizadeh, genetisch epidemioloog
Dr. H. Groen, universitair docent epidemiologie
Mw. dr. I.M. Nolte, universitair docent epidemiologie

Medical device specialist

Dr. M.J.W. Greuter, klinisch fysicus
Dr. ir. J. Sjollema, universitair docent / fysicus

Juristen

Mr. D. Renkema, jurist gezondheidsrecht
Mw. mr. W.B. Veen, jurist
Mr. J.W.P. de Vroedt MHA, jurist / stafmedewerker
Mw. mr. J. Zaal, jurist/stafmedewerker

Klinisch farmacologen

Mw. prof. dr. P.M.L.A. van den Bemt, klinisch farmacoloog
Dr. M.S. Bolhuis, klinisch farmacoloog
Prof. dr. H.J. Lambers Heerspink, klinisch farmacoloog / onderzoeker
Mw. dr. M .G.G. Sturkenboom, klinisch farmacoloog

Ziekenhuisapothekers

Mw. prof. dr. P.M.L.A. van den Bemt, ziekenhuisapotheker
Dr. M.S. Bolhuis, ziekenhuisapotheker
Mw. dr. M .G.G. Sturkenboom, ziekenhuisapotheker

Proefpersoon leden

Mw. W. Hoek, adviseur/begeleider wonen, zorg, hulpverlening en welzijn
Mw. C.M. Verlind, fysiotherapeut n.p.
Mw. drs. N. van Wijngaarden, bestuurssecretaris

Overige leden

Dr. G.W. van Imhoff, internist-hematoloog niet-praktiserend
Prof. dr. W. Helfrich, hoogleraar Translationele Chirurgische Oncologie
Prof. dr. C.G.M. Kallenberg, emeritus hoogleraar Interne Geneeskunde i.h.b. Klinische Immunologie
Mw. dr. A.C. Muller Kobold, klinisch chemicus
Dr. H.G.O.M. Smid, psycholoog
Mw. R. A.E. Tooten, Bsc, clinical trial specialist
Dr. E.L. van der Veen, ziekenhuisapotheker i.o.
Dr. ir. P.J.F. de Vries, consultant voedingswetenschappen
Mw. dr. F. Zwiers-Blokzijl, verpleegkundig specialist & postdoc onderzoeker

EUROPEAN MEDICINES AGENCY
SCIENCE MEDICINES HEALTH

Report for the Application Evaluation Decision

A non-inferiority, randomised and controlled trial to compare the safety, tolerability and preliminary efficacy between standard and Torque Teno virus-guided immunosuppression in stable adult kidney transplant recipients with low immunological risk in the first year after transplantation

2022-500024-30-00

Decision

MSC:

France

Decision:

Authorised

Reporting Date:

14/10/2022

Tacit decision:

No

Application Part:

Conditions:

Reason:

Justification:

Deferrals

Disagreement with Part I

Submission date:

14/10/2022

EUROPEAN MEDICINES AGENCY
SCIENCE MEDICINES HEALTH

Report for the Application Evaluation Decision

A non-inferiority, randomised and controlled trial to compare the safety, tolerability and preliminary efficacy between standard and Torque Teno virus-guided immunosuppression in stable adult kidney transplant recipients with low immunological risk in the first year after transplantation

2022-500024-30-00

Decision

MSC:

Spain

Decision:

Authorised

Reporting Date:

13/10/2022

Tacit decision:

No

Application Part:

Conditions:

Reason:

Justification:

Deferrals

Disagreement with Part I

Submission date:

13/10/2022

Report for the Application Evaluation Decision

A non-inferiority, randomised and controlled trial to compare the safety, tolerability and preliminary efficacy between standard and Torque Teno virus-guided immunosuppression in stable adult kidney transplant recipients with low immunological risk in the first year after transplantation

2022-500024-30-00

Decision

MSC:

Germany

Decision:

Authorised

Reporting Date:

13/10/2022

Tacit decision:

No

Application Part:

Conditions:

Reason:

Justification:

Deferrals

Disagreement with Part I

Submission date:

13/10/2022

Bundesinstitut
für Arzneimittel
und Medizinprodukte

BfArM, Kurt-Georg-Kiesinger-Allee 3, 53175 Bonn

Medical University of Vienna
Spitalgasse 23
1090 Vienna

ABTEILUNG Informationstechnik, Klinische Prüfung
FACHGRUPPE Klinische Prüfung
TEL +49 (0)228 99 307-4318
FAX +49 (0)228 99 307-4355
E-MAIL ct@bfarm.de

HAUS-ANSCHRIFT Kurt-Georg-Kiesinger-Allee 3
53175 Bonn
TEL +49 (0)228 99 307-0
INTERNET www.bfarm.de

Bonn, 11.10.2022
GESCHZ 10.3-2022-500024-30-00-B-00005-001

Verfahren der Genehmigung nachträglicher Änderungen zu einer klinischen Prüfung gemäß Artikel 16, 19, 20, 23 der Verordnung (EU) Nr. 536/2014 i.V.m. §§ 40, 40c AMG

Antrag vom 18.08.2022 (Part I) / 18.08.2022 (Part II)

**EU-CT-Nummer**	**2022-500024-30-00**
**Verfahrensnummer**	**B_00005-001**
**Sponsor**	**Medical University of Vienna, Vienna, Austria**
**Studientitel**	**A non-inferiority, randomised and controlled trial to compare the safety, tolerability and preliminary efficacy between standard and Torque Teno virus-guided immunosuppression in stable adult kidney transplant recipients with low immunological risk in the first year after transplantation**

Bescheid

Die beantragten nachträglichen Änderungen werden

☑ genehmigt.
☐ mit Auflagen genehmigt. Die Auflagen lauten wie folgt:
Auflagen

<u>Bewertung:</u>

A. Schlussfolgerung zu Teil I des Bewertungsberichts:

☐ Die Bundesrepublik Deutschland ist als berichterstattender Mitgliedstaat (rMS) beteiligt und kommt in Bezug auf Teil I des Bewertungsberichts zu dem Schluss, dass die wesentlichen Änderungen

☐ vertretbar sind.

☐ mit Auflagen gemäß Artikel 19 Absatz 1 Unterabsatz 3 Verordnung (EU) Nr. 536/2014 vertretbar sind, siehe oben. Die Begründung ist als Anlage Begründung I beigefügt.

☑ Die Bundesrepublik Deutschland ist als betroffener Mitgliedstaat (MSc) beteiligt und kommt in Übereinstimmung mit der Schlussfolgerung des rMS zu dem Schluss, dass die wesentlichen Änderungen

☑ vertretbar sind gemäß Artikel 19 Absatz 2 Verordnung (EU) Nr. 536/2014

☐ mit Auflagen vertretbar sind gemäß Artikel 19 Absatz 2 Verordnung (EU) Nr. 536/2014, siehe oben. Die Begründung ist als Anlage Begründung I beigefügt.

☐ Die Bundesoberbehörde weicht von der Stellungnahme der Ethik-Kommission ab, § 40c Absatz 1 AMG.

Bezeichnung der Ethik-Kommission: Ethik-Kommission der Ärztekammer Nordrhein

Wiedergabe der Stellungnahme und Begründung für das Abweichen, siehe Anlage Abweichung.

B. Schlussfolgerung zu Teil II des Bewertungsberichts:

Bezeichnung der Ethik-Kommission:
Ethik-Kommission der Ärztekammer Nordrhein
Tersteegenstr. 9
Düsseldorf

Die Ethik-Kommission kommt zu dem Schluss, dass die wesentlichen Änderungen

☑ vertretbar sind.

☐ mit Auflagen vertretbar sind, siehe oben. Die Begründung ist als Anlage Begründung II beigefügt.

C. Einhaltung nationaler Vorschriften

☑ Kein Verstoß gegen § 40a Satz 1 Nr. 1 bis 3 und Nr. 5, Satz 2 und 3 AMG sowie § 40b Absatz 2, 3 Satz 1, Absatz 4 Satz 1, 3 bis 9, Absatz 5 und 6 AMG

☐ Die Bundesoberbehörde weicht von der Stellungnahme der Ethik-Kommission ab, § 40c Absatz 1 AMG.

Wiedergabe der Stellungnahme und Begründung für das Abweichen, siehe Anlage Abweichung.

Rechtsbehelfsbelehrung:

Gegen diesen Bescheid kann innerhalb eines Monats nach Bekanntgabe Widerspruch erhoben werden. Der Widerspruch ist bei dem Bundesinstitut für Arzneimittel und Medizinprodukte (BfArM) in Bonn einzulegen.

Mit freundlichen Grüßen
Im Auftrag

Dr. C. Riedel

Dieser Bescheid enthält in Übereinstimmung mit § 37 Absatz 3 Satz 1 Verwaltungsverfahrensgesetz nur eine Namenswiedergabe und keine Unterschrift.

Anlagen

Anlage Begründung I

entfällt

Anlage Begründung II

entfällt

Anlage Abweichung

entfällt

EUROPEAN MEDICINES AGENCY
SCIENCE MEDICINES HEALTH

Report for the Application Evaluation Decision

A non-inferiority, randomised and controlled trial to compare the safety, tolerability and preliminary efficacy between standard and Torque Teno virus-guided immunosuppression in stable adult kidney transplant recipients with low immunological risk in the first year after transplantation

2022-500024-30-00

Decision

MSC:

Austria

Decision:

Authorised

Reporting Date:

12/10/2022

Tacit decision:

No

Application Part:

Conditions:

Reason:

Justification:

Deferrals

Disagreement with Part I

Submission date:

12/10/2022

Bundesamt für
Sicherheit im
Gesundheitswesen
BASG

BASG/AGES
Institute Surveillance
Traisengasse 5, 1200 Vienna

Medical University of Vienna
Department for Nephrology and Dialysis
Gregor Bond
Spitalgasse 23
1090 Wien

**date:**	12.10.2022
**department:**	Clinical Trials (CLTR)
**phone:**	+43(0)5 0555 36827
**e-mail:**	clinicaltrials@ages.at
**reference:**	101271932

Notification of single decision according to Article 23 of REG (EU) 536/2014

Dear ladies and gentlemen,

the Federal Office for Safety in Healthcare („Bundesamt für Sicherheit im Gesundheitswesen") as the competent authority for clinical trials in Austria herewith notifies you about the decision for the substantial modification SM-1 for the clinical trial.

2022-500024-30-00

A non-inferiority, randomised and controlled trial to compare the safety, tolerability and preliminary efficacy between standard and Torque Teno virus-guided immunosuppression in stable adult kidney transplant recipients with low immunological risk in the first year after transplantation

The substantial modification SM-1 is approved.

This formal letter is to be considered as notification within the terms of Article 23, section 6. The formal legal decision will be issued within the next 14 days.

For the Federal Office

Strasser Stefan
am 12.10.2022

BUNDESAMT FÜR SICHERHEIT IM GESUNDHEITSWESEN
AMTSSIGNATUR

Dieses Dokument wurde amtssigniert.

Informationen zur Prüfung der elektronischen Signatur und des Ausdrucks finden Sie unter http://www.basg.gv.at/amtssignatur.

Bundesamt für Sicherheit im Gesundheitswesen
Traisengasse 5, 1200 Wien

EUROPEAN MEDICINES AGENCY
SCIENCE MEDICINES HEALTH

Report for the Application Evaluation Decision

A non-inferiority, randomised and controlled trial to compare the safety, tolerability and preliminary efficacy between standard and Torque Teno virus-guided immunosuppression in stable adult kidney transplant recipients with low immunological risk in the first year after transplantation

2022-500024-30-00

Decision

MSC:

Czechia

Decision:

Authorised

Reporting Date:

17/10/2022

Tacit decision:

No

Application Part:

Conditions:

Reason:

Justification:

Deferrals

Disagreement with Part I

Submission date:

17/10/2022

STÁTNÍ ÚSTAV PRO KONTROLU LÉČIV
Šrobárova 48
100 41 Praha 10
Telefon: +420 272 185 111
Fax: +420 271 732 377
E-mail: posta@sukl.cz
Web: www.sukl.cz

ADRESÁT
Medical University Of Vienna
Währinger Gürtel 18-20 Alsergrund
1090 Vienna
Austria

Spisová zn.	Vyřizuje / linka	Datum
51138/22-ctis-I	Dr. E. Hrušková Reinová / 317	17. 10. 2022

ROZHODNUTÍ

Státní ústav pro kontrolu léčiv se sídlem v Praze 10, Šrobárova 48 (dále jen „Ústav"), jako orgán příslušný k rozhodnutí podle § 13 odst. 2 písm. b) č. 378/2007 Sb., o léčivech a o změnách některých souvisejících zákonů (zákon o léčivech), ve znění pozdějších předpisů (dále jen „zákon o léčivech"), **rozhodl** v souladu s § 67 a násl. zákona č. 500/2004 Sb., správní řád, ve znění pozdějších předpisů (dále jen „správní řád"), v řízení o povolení významné změny klinického hodnocení humánního léčivého přípravku vedeného podle § 51 zákona o léčivech ve spojení s čl. 17 a násl. nařízení Evropského Parlamentu a Rady (EU) č. 536/2014 ze dne 16. dubna 2014 o klinických hodnoceních humánních léčivých přípravků a o zrušení směrnice 2001/20/ES (dále jen „nařízení o klinickém hodnocení"), o žádosti o povolení významné změny části I a části II klinického hodnocení podané prostřednictví portálu EU dne **18. 08. 2022** ohledně významné změny klinického hodnocení léčivého přípravku s názvem **A non-inferiority, randomised and controlled trial to compare the safety, tolerability and preliminary efficacy between standard and Torque Teno virus-guided immunosuppression in stable adult kidney transplant recipients with low immunological risk in the first year after transplantation** (dále jen „předmětné klinické hodnocení"), společnosti **Medical University Of Vienna**, se sídlem Spitalgasse 23 Alsergrund, 1090 Vienna, Austria, EU číslo **2022-500024-30-00**

t a k t o :

Ústav v souladu s § 51 odst. 4 zákona o léčivech ve spojení s čl. 23 odst. 1 nařízení o klinickém hodnocení **povoluje významnou změnu předmětného klinického hodnocení**.

O d ů v o d n ě n í

Dne 18. 08. 2022 byla předložena žádost účastníka řízení o povolení významné změny předmětného klinického hodnocení prostřednictvím portálu EU s vyznačením České republiky jako dotčeného členského státu ve smyslu čl. 2 odst. 2 bod 12. nařízení o klinickém hodnocení. Předložením této žádosti bylo zahájeno správní řízení vedené pod sp. zn. 51138/22-ctis-I

Ústav na základě předložené žádosti provedl řízení v souladu s § 51 odst. 3 písm. b) a c) zákona o léčivech ve spojení s čl. 21 a 22 a následující nařízení o klinickém hodnocení, a konstatuje, že předložená žádost splňuje požadavky relevantních právních předpisů.

S ohledem na výše uvedené rozhodl Ústav tak, jak je uvedeno ve výroku tohoto rozhodnutí.

Seznam schválené dokumentace:

Part I:

1. Trial Protocol Version 6.0F, 15.07.2022

Part II:

1. Informace pro pacienta a formulář informovaného souhlasu, verze 6.0F, 15.07.2022
2. Informace pro pacienta a formulář informovaného souhlasu k zacházení s osobními údaji a biologickými vzorky, verze 5.0F, 15.07.2022
3. Deník pacienta pro klinické hodnocení TTV guide IT, verze 1.0 ze dne 27.05.2022/Rozložení upraveno 01.06.2022
4. Klinická studie TTV guide IT – Patient folder_CZ
5. Certificate of Clinical Trial Insurance, 09.08.2022
6. Pojistná smlouva, 09.08.2022

P o u č e n í o o d v o l á n í

Proti tomuto rozhodnutí je možno podat podle § 81 a násl. správního řádu u Ústavu odvolání, a to ve lhůtě 15 dnů ode dne jeho doručení. O odvolání rozhoduje Ministerstvo zdravotnictví ČR.

Otisk úředního razítka

MUDr. Alice Němcová

Digitálně podepsal MUDr. Alice Němcová
Datum: 2022.10.17 08:31:50 +02'00'

MUDr. Alice Němcová

Ředitelka Odboru klinického hodnocení léčivých přípravků
