# Supplementary material for: A multicentre, patient- and assessor-blinded, non-inferiority, randomised and controlled phase II trial to compare standard and torque teno virus-guided immunosuppression in kidney transplant recipients in the first year after transplantation: TTVguideIT
Source: Trials. 2023 Mar 22;24:213. doi: 10.1186/s13063-023-07216-0 (PMC10032258; doi:10.1186/s13063-023-07216-0)
Supplement: Supplementary file 10 — Additional file 10. [file 13063_2023_7216_MOESM10_ESM.pdf]

Ref. Ares(2021)712291 - 28/01/2021

EUROPEAN COMMISSION
Directorate-General for Research and Innovation

RTD.E – People
E.02 – Combatting Diseases

GRANT AGREEMENT DATA SHEET

Grant Agreement (GA) No: 896932

Acronym: TTV GUIDE TX

Title: PERSONALISATION OF IMMUNOSUPPRESSION BY MONITORING VIRAL LOAD POST KIDNEY TRANSPLANTATION - A RANDOMISED CONTROLLED PHASE II TRIAL

Call/Topic: New interventions for Non-Communicable Diseases

Type of action: Research and Innovation action

Granted by: European Union

Consortium agreement required: Yes

Collaboration agreement required: No

Coordination agreement required: No

Duration (in months): 60

Starting Date: 1 May 2021

Justification: Due to the COVID epidemic the Virology Labs are overrun at the time, and it is not possible to start implementing standardized TTV screenings until Spring. For this reason we request a delay until 1. Mai 2021

End date: 30 April 2026

Reimbursement rate: 100% of the action's eligible costs

Total costs (including non-EU funded)	Total costs	Maximum grant amount	Forms of costs
6 099 831.00	6 099 831.00	6 099 831.00	Unit, actual, flat rate, unit

Prefinancing and guarantees:

Prefinancing amount	Guarantee fund Amount
2 439 932.40	304 991.55

Bank account (IBAN, BIC) for payments: AT362011140410070700

Reporting periods:

No	Month From	Month To
1	1	18
2	19	30
3	31	42
4	43	60

Obligation to keep records (in years): 5

Right to carry out reviews (up to number of years: 2

Right to carry out audits (up to number of years): 2

Extension of audit findings from other grants to this grant (no later than number of years): 2

Evaluation of the impact of the action (up to number of years): 5

List of beneficiaries and linked third parties and international partners:

Beneficiary/ linked third party/ international partner	Role	PIC	Full official name	Country	Total costs (including non-EU funded)	Max. reimb. rate	Max Grant Amount
1 - MUW	CO	999989976	MEDIZINISCHE UNIVERSITAET WIEN	AT	973 306.25	100.00%	973 306.25
2 - BIOMERIEUX SA	BEN	973654088	BIOMERIEUX SA	FR	801 750.00	100.00%	801 750.00
3 - EUTEMA-RS	BEN	897856542	EUTEMA RESEARCH SERVICES GMBH	AT	435 750.00	100.00%	435 750.00
4 - ECRIN	BEN	948646712	ECRIN EUROPEAN CLINICAL RESEARCH INFRASTRUCTURE NETWORK	FR	108 136.00	100.00%	108 136.00

Beneficiary/ linked third party/ international partner	Role	PIC	Full official name	Country	Total costs (including non-EU funded)	Max. reimb. rate	Max Grant Amount
MU	LinkTP	999880657	Masarykova univerzita	CZ	27 860.00	100.00%	27 860.00
5 - MUI	BEN	999855437	MEDIZINISCHE UNIVERSITAT INNSBRUCK	AT	288 750.00	100.00%	288 750.00
6 - MUG	BEN	999836231	MEDIZINISCHE UNIVERSITAT GRAZ	AT	191 562.50	100.00%	191 562.50
7 - OKLinz	BEN	902282458	ORDENSKLINIKUM LINZ GMBH	AT	191 562.50	100.00%	191 562.50
8 - TUD	BEN	999897729	TECHNISCHE UNIVERSITAET DRESDEN	DE	722 268.75	100.00%	722 268.75
9 - UHREG	BEN	999886477	KLINIKUM DER UNIVERSITAET REGENSBURG	DE	206 562.50	100.00%	206 562.50
10 - CHARITE	BEN	999992692	CHARITE - UNIVERSITAETSMEDIZIN BERLIN	DE	191 562.50	100.00%	191 562.50
11 - IKEM	BEN	999609154	Institut klinické a experimentální mediciny	CZ	288 750.00	100.00%	288 750.00
12 - HULAFE	BEN	995991539	FUNDACION PARA LA INVESTIGACION DEL HOSPITAL UNIVERSITARIO LA FE DE LA COMUNIDAD VALENCIANA	ES	129 062.50	100.00%	129 062.50
13 - INCLIVA	BEN	999637187	FUNDACION PARA LA INVESTIGACION DEL HOSPITAL CLINICO DE LA COMUNITAT VALENCIANA, FUNDACION INCLIVA	ES	85 625.00	100.00%	85 625.00
14 - CHU	BEN	997220723	CENTRE HOSPITALIER UNIVERSITAIRE DE GRENOBLE	FR	236 760.00	100.00%	236 760.00
15 - HOPITAL CIVIL	BEN	951206930	HOPITAUX UNIVERSITAIRES DE STRASBOURG	FR	191 562.50	100.00%	191 562.50
16 - UNIPI	BEN	999862712	UNIVERSITA DI PISA	IT	137 125.00	100.00%	137 125.00
17 - LUMC	BEN	999990849	ACADEMISCH ZIEKENHUIS LEIDEN	NL	303 750.00	100.00%	303 750.00

Beneficiary/ linked third party/ international partner	Role	PIC	Full official name	Country	Total costs (including non-EU funded)	Max. reimb. rate	Max Grant Amount
18 - UMCG	BEN	999914801	ACADEMISCH ZIEKENHUIS GRONINGEN	NL	393 437.50	100.00%	393 437.50
19 - UGR	BEN	999882015	UNIVERSIDAD DE GRANADA	ES	194 687.50	100.00%	194 687.50

GA options:
Article 6.2.D.2 – Equipment costs – depreciation
Article 6.2.D.4 – Large research infrastructure (LRI) costs
Article 14 – Linked third parties
Article 29.3 – Actions that participate in the Open Research Data Pilot
Article 29.3 – Health actions that participate in the Open Research Data Pilot
Article 31.5 – Access rights to results for EU – EU grants
